# General and abdominal adiposity and hypertension in eight world regions: a pooled analysis of 837 population-based studies with 7·5 million participants

**DOI:** 10.1016/S0140-6736(24)01405-3

**Published:** 2024-08-31

**Authors:** NCD Risk Factor Collaboration (NCD-RisC), Pooled analysis and writing, Bin Zhou, James E Bennett, Aidan P Wickham, Rosie K Singleton, Anu Mishra, Rodrigo M Carrillo-Larco, Nayu Ikeda, Lakshya Jain, Ana Barradas-Pires, Rachel A Heap, Victor PF Lhoste, Kate E Sheffer, Nowell H Phelps, Archie W Rayner, Edward W Gregg, Mark Woodward, Gretchen A Stevens, Maria LC Iurilli, Goodarz Danaei, Mariachiara Di Cesare, Carlos A Aguilar-Salinas, Noor Ani Ahmad, Pascal Bovet, Zhengming Chen, Albertino Damasceno, Sarah L Filippi, Imre Janszky, Andre P Kengne, Young-Ho Khang, Kamlesh Khunti, Avula Laxmaiah, Lee-Ling Lim, Lauren Lissner, Paula Margozzini, Jean Claude Mbanya, Stephen McGarvey, Jonathan E Shaw, Stefan Söderberg, Luis Adrián Soto-Mota, Junyang Wang, Francesco Zaccardi, Majid Ezzati, Country and regional data, Leandra Abarca-Gómez, Mohsen Abbasi-Kangevari, Shynar Abdrakhmanova, Suhaila Abdul Ghaffar, Hanan F Abdul Rahim, Zulfiya Abdurrahmonova, Niveen M Abu-Rmeileh, Benjamin Acosta-Cazares, Ishag Adam, Marzena Adamczyk, Wichai Aekplakorn, Imelda A Agdeppa, Javad Aghazadeh-Attari, Charles Agyemang, Mohamad Hasnan Ahmad, Noor Ani Ahmad, Ali Ahmadi, Naser Ahmadi, Nastaran Ahmadi, Soheir H Ahmed, Wolfgang Ahrens, Gulmira Aitmurzaeva, Kamel Ajlouni, Hazzaa M Al-Hazzaa, Halima Al-Hinai, Jawad A Al-Lawati, Rajaa Al-Raddadi, Deena Al Asfoor, Huda M Al Hourani, Monira Alarouj, Fadia AlBuhairan, Shahla AlDhukair, Mohamed M Ali, Anna V Alieva, Abdullah Alkandari, Buthaina M Alkhatib, Eman Aly, Deepak N Amarapurkar, Pilar Amiano Etxezarreta, Norbert Amougou, Lars Bo Andersen, Sigmund A Anderssen, Odysseas Androutsos, Ranjit Mohan Anjana, Alireza Ansari-Moghaddam, Elena Anufrieva, Hajer Aounallah-Skhiri, Tahir Aris, Raphael E Arku, Nimmathota Arlappa, Krishna K Aryal, Felix K Assah, Batyrbek Assembekov, Maria Cecília F Assunção, Juha Auvinen, Mária Avdičová, Kishwar Azad, Ana Azevedo, Mohsen Azimi-Nezhad, Fereidoun Azizi, Flora Bacopoulou, Suhad Bahijri, Izet Bajramovic, Nagalla Balakrishna, Mohamed Bamoshmoosh, Maciej Banach, Piotr Bandosz, José R Banegas, Rafał Baran, Carlo M Barbagallo, Valter Barbosa Filho, Alberto Barceló, Maja Baretić, Joaquin Barnoya, Lena Barrera, Aluisio JD Barros, Mauro Virgílio Gomes Barros, Abdul Basit, Joao Luiz Bastos, Anwar M Batieha, Aline P Batista, Rosangela L Batista, Zhamilya Battakova, Louise A Baur, Pascal M Bayauli, Silvia Bel-Serrat, Antonisamy Belavendra, Habiba Ben Romdhane, Theodora Benedek, Judith Benedics, Mikhail Benet, Gilda Estela Benitez Rolandi, James E Bennett, Michaela Benzeval, Elling Bere, Nicolas Berger, Ingunn Holden Bergh, Salim Berkinbayev, Antonio Bernabe-Ortiz, Heloísa Bettiol, Augustin F Beybey, Jorge Bezerra, Aroor Bhagyalaxmi, Santosh K Bhargava, Elysée Claude Bika Lele, Mukharram M Bikbov, Bihungum Bista, Dusko J Bjelica, Peter Bjerregaard, Espen Bjertness, Marius B Bjertness, Cecilia Björkelund, Katia V Bloch, Anneke Blokstra, Simona Bo, Martin Bobak, Lynne M Boddy, Bernhard O Boehm, Jose G Boggia, Elena Bogova, Marialaura Bonaccio, Alice Bonilla-Vargas, Herman Borghs, Steve Botomba, Rupert Bourne, Pascal Bovet, Khadichamo Boymatova, Lutgart Braeckman, Tasanee Braithwaite, Imperia Brajkovich, Francesco Branca, Hermann Brenner, Lizzy M Brewster, Yajaira Briceño, Lacramioara Brinduse, Bettina Bringolf-Isler, Miguel Brito, Johannes Brug, Anna Bugge, Frank Buntinx, Marta Buoncristiano, Con Burns, Antonio Cabrera de León, Roberta B Caixeta, Tilema Cama, Günay Can, Ana Paula C Cândido, Felicia Cañete, Mario V Capanzana, Naděžda Čapková, Eduardo Capuano, Rocco Capuano, Vincenzo Capuano, Viviane C Cardoso, Axel C Carlsson, Rodrigo M Carrillo-Larco, Felipe F Casanueva, Maribel Casas, Laura Censi, Marvin Cervantes-Loaiza, Parinya Chamnan, Snehalatha Chamukuttan, Queenie Chan, Nish Chaturvedi, Fangfang Chen, Huashuai Chen, Long-Sheng Chen, Zhengming Chen, Yiling J Cheng, Bahman Cheraghian, Angela Chetrit, Ekaterina Chikova-Iscener, Mai JM Chinapaw, Anne Chinnock, Arnaud Chiolero, Adela Chirita-Emandi, María-Dolores Chirlaque, Chean Lin Chong, Diego G Christofaro, Jerzy Chudek, Renata Cifkova, Massimo Cirillo, Frank Claessens, Philip Clare, Emmanuel Cohen, Susana C Confortin, Tara C Coppinger, Lilia Yadira Cortés, Cojocaru R Cosmin, Simona Costanzo, Melanie J Cowan, Chris Cowell, Amelia C Crampin, Amanda J Cross, Ana B Crujeiras, Juan J Cruz, Alexandra M Cucu, Felipe V Cureau, Sarah Cuschieri, Graziella D’Arrigo, Eleonora d’Orsi, Haroldo da Silva-Ferreira, Christina C Dahm, Jean Dallongeville, Albertino Damasceno, Rachel Dankner, Kairat Davletov, Francisco de Assis Guedes de Vasconcelos, Maria Alice Altenburg de Assis, Dirk De Bacquer, Jaco De Bacquer, Jeroen de Bont, Amalia De Curtis, Patrícia Fragas Hinnig, Giovanni de Gaetano, Stefaan De Henauw, Pilar De Miguel-Etayo, Paula Duarte de Oliveira, Karina Mary de Paiva, Karin De Ridder, Marco Aurélio de Valois Correia Júnior, Mohan Deepa, Vincent Jr DeGennaro, Stefaan Demarest, Elaine Dennison, Valérie Deschamps, Meghnath Dhimal, María Pilar Díez Ripollés, Zivka Dika, Shirin Djalalinia, Liria Dominguez, Maria Benedetta Donati, Chiara Donfrancesco, Guanghui Dong, Silvana P Donoso, Maria Dorobantu, Marcus Dörr, Nico Dragano, Wojciech Drygas, Shufa Du, Charmaine A Duante, Priscilla Duboz, Rosemary B Duda, Vesselka L Duleva, Anar Dushpanova, Azhar Dyussupova, Elzbieta Dziankowska-Zaborszczyk, Narges Ebrahimi, Guadalupe Echeverría, Ricky Eddie, Ebrahim Eftekhar, Vasiliki Efthymiou, Eruke E Egbagbe, Sareh Eghtesad, Ulf Ekelund, Mohammad El-Khateeb, Jalila El Ati, Roberto Elosua, Ofem Enang, Rajiv T Erasmus, Cihangir Erem, Gul Ergor, Louise Eriksen, Johan G Eriksson, Jorge Escobedo-de la Peña, Ali Esmaeili, Roger G Evans, Ildar Fakhradiyev, Albina A Fakhretdinova, Caroline H Fall, Elnaz Faramarzi, Mojtaba Farjam, Farshad Farzadfar, Yosef Farzi, Mohammad Reza Fattahi, Asher Fawwad, Francisco J Felix-Redondo, Trevor S Ferguson, Romulo A Fernandes, Daniel Fernández-Bergés, Desha R Fernando, Daniel Ferrante, Gerson Ferrari, Marika Ferrari, Catterina Ferreccio, Eldridge Ferrer, Thamara Hubler Figueiró, Anna Fijalkowska, Günther Fink María Pilar, Mauro Fisberg, Maria Forsner, Edward F Fottrell, Heba M Fouad, Damian K Francis, Guillermo Frontera, Flavio D Fuchs, Sandra C Fuchs, Viktoriya Furdela, Takuro Furusawa, Stefan Adela Gabriela, Zbigniew Gaciong, Manuel Galán Cuesta, Andrzej Galbarczyk, Sonya V Galcheva, Myriam Galfo, Manoli Garcia-de-la-Hera, Pablo Garcia, Sarah P Garnett, Magda Gasull, Andrea Gazzinelli, Ulrike Gehring, Eva Gerdts, Ebrahim Ghaderi, Seyyed-Hadi Ghamari, Ali Ghanbari, Erfan Ghasemi, Oana-Florentina Gheorghe-Fronea, Anup Ghimire, Alessandro Gialluisi, Simona Giampaoli, Francesco Gianfagna, Glen Gironella, Aleksander Giwercman, Konstantinos Gkiouras, Natalya Glushkova, Ramesh Godara, Justyna Godos, Marcel Goldberg, Georgina Gómez, Jesús Humberto Gómez Gómez, Luis F Gomez, Santiago F Gómez, Aleksandra Gomula, Bruna Gonçalves Cordeiro da Silva, Helen Gonçalves, Mauer Gonçalves, Ana D González-Alvarez, David A Gonzalez-Chica, Esther M González-Gil, Marcela Gonzalez-Gross, Juan P González-Rivas, Angel R Gonzalez, Frederic Gottrand, Dušan Grafnetter, Aneta Grajda, Maria G Grammatikopoulou, Edward W Gregg, Tomasz Grodzicki, Else Karin Grøholt, Anders Grøntved, Viviana Guajardo, Pilar Guallar-Castillón, Maëlenn Guerchet, Ramiro Guerrero, Andre L Guimaraes, Unjali P Gujral, Martin C Gulliford, Marc J Gunter, Rajeev Gupta, Oye Gureje, Mirjana A Gurinović, Beata Gurzkowska, Laura Gutierrez, Xinyi Gwee, Rosa Haghshenas, Hamid Hakimi, Jytte Halkjær, Ian R Hambleton, Behrooz Hamzeh, Willem A Hanekom, Dominique Hange, Abu AM Hanif, Sari Hantunen, Jie Hao, Carla Menêses Hardman, Louise Hardy, Rachakulla Hari Kumar, Javad Harooni, Seyed Mohammad Hashemi-Shahri, Maria Hassapidou, Jun Hata, Teresa Haugsgjerd, Mirjam Heinen, Marleen Elisabeth Hendriks, Rafael dos Santos Henrique, Ana Henriques, Leticia Hernandez Cadena, Sauli Herrala, Marianella Herrera-Cuenca, Victor M Herrera, Isabelle Herter-Aeberli, Karl-Heinz Herzig, Ramin Heshmat, Allan G Hill, Sai Yin Ho, Michelle Holdsworth, Reza Homayounfar, Clara Homs, Emiel O Hoogendijk, Andrea RVR Horimoto, Claudia M Hormiga, Bernardo L Horta, Leila Houti, Christina Howitt, Thein Thein Htay, Aung Soe Htet, Maung Maung Than Htike, José María Huerta, Ilpo Tapani Huhtaniemi, Laetitia Huiart, Constanta Huidumac Petrescu, Martijn Huisman, Abdullatif Husseini, Inge Huybrechts, Nahla Hwalla, Licia Iacoviello, Ellina M Iakupova, Anna G Iannone, Jannicke Igland, Chinwuba Ijoma, Nayu Ikeda, Violeta Iotova, Vilma E Irazola, Takafumi Ishida, Godsent C Isiguzo, Muhammad Islam, Sheikh Mohammed Shariful Islam, Duygu Islek, Till Ittermann, Ivaila Y Ivanova-Pandourska, Masanori Iwasaki, Tuija Jääskeläinen, Rod T Jackson, Hashem Y Jaddou, Michel Jadoul, Tazeen Jafar, Nataša Jan, Imre Janszky, Edward Janus, Juel Jarani, Gerald Jarnig, Marjo-Riitta Jarvelin, Grazyna Jasienska, Ana Jelaković, Bojan Jelaković, Anjani Kumar Jha, Ramon O Jimenez, Karl-Heinz Jöckel, Michel Joffres, Jari J Jokelainen, Jost B Jonas, Pradeep Joshi, Rohina Joshi, Josipa Josipović, Farahnaz Joukar, Jacek J Jóźwiak, Anne Juolevi, Vesna Juresa, Vesna Jureša, Rudolf Kaaks, Felix O Kaducu, Agnes L Kadvan, Anthony Kafatos, Eero O Kajantie, Natia Kakutia, Daniela Kállayová, Zhanna Kalmatayeva, Ofra Kalter-Leibovici, Srinivasan Kannan, Efthymios Kapantais, Eva Karaglani, Argyro Karakosta, Khem B Karki, Adoubi Kassi Anicet, Marzieh Katibeh, Prasad Katulanda, Peter T Katzmarzyk, Jussi Kauhanen, Gyulli M Kazakbaeva, François F Kaze, Calvin Ke, Sirkka Keinänen-Kiukaanniemi, Roya Kelishadi, Cecily Kelleher, Han CG Kemper, Andre P Kengne, Maryam Keramati, Mathilde Kersting, Yousef Saleh Khader, Arsalan Khaledifar, Davood Khalili, Young-Ho Khang, Bahareh Kheiri, Motahareh Kheradmand, Alireza Khosravi, Ursula Kiechl-Kohlendorfer, Sophia J Kiechl, Stefan Kiechl, Hyeon Chang Kim, Heidi Klakk, Suntara Klanarong, Jana Klanova, Magdalena Klimek, Michael Knoflach, Susanne Kobel, Bhawesh Koirala, Elin Kolle, Patrick Kolsteren, Jürgen König, Raija Korpelainen, Paul Korrovits, Magdalena Korzycka, Jelena Kos, Seppo Koskinen, Malik Koussoh Simone, Éva Kovács, Irina Kovalskys, Sudhir Kowlessur, Slawomir Koziel, Jana Kratenova, Wolfgang Kratzer, Susi Kriemler, Peter Lund Kristensen, Helena Krizan, Maria F Kroker-Lobos, Steinar Krokstad, Herculina S Kruger, Ruan Kruger, Łukasz Kryst, Ruzena Kubinova, Urho M Kujala, Enisa Kujundzic, Zbigniew Kulaga, Mukhtar Kulimbet, Meena Kumari, Marie Kunešová, Pawel Kurjata, Catherine Kyobutungi, Quang Ngoc La, Demetre Labadarios, Carl Lachat, Daphne Lai, Youcef Laid, Lachmie Lall, Maritza Landaeta Jimenez, Edwige Landais, Tiina Lankila, Vera Lanska, Georg Lappas, Bagher Larijani, Mina P Lateva, Tint Swe Latt, Martino Laurenzi, Avula Laxmaiah, Maria Lazo-Porras, Gwenaëlle Le Coroller, Khanh Le Nguyen Bao, Terho Lehtimäki, Daniel Lemogoum, Elvynna Leong, Justyna Leszczak, Gabriel M Leung, Yanping Li, Merike Liivak, Charlie Lim, Wei-Yen Lim, M Fernanda Lima-Costa, Hsien-Ho Lin, Lars Lind, Lauren Lissner, Mieczyslaw Litwin, Liping Liu, Xiaotian Liu, Guadalupe Longo Abril, Oscar Lopes, Esther Lopez-Garcia, José Francisco López-Gil, Tania Lopez, José Eugenio Lozano, Janice L Lukrafka, Dalia Luksiene, Annamari Lundqvist, Nuno Lunet, Charles Lunogelo, Michala Lustigová, Jean-René M’Buyamba-Kabangu, George LL Machado-Coelho, Aristides M Machado-Rodrigues, Enguerran Macia, Ahmed A Madar, Gladys E Maestre, Stefania Maggi, Dianna J Magliano, Sara Magnacca, Emmanuella Magriplis, Gowri Mahasampath, Bernard Maire, Marcia Makdisse, Mohammad-Reza Malekpour, Fatemeh Malekzadeh, Reza Malekzadeh, Kodavanti Mallikharjuna Rao, Sofia Malyutina, Lynell V Maniego, Yannis Manios, Jim I Mann, Fariborz Mansour-Ghanaei, Enzo Manzato, Mala Ali Mapatano, Paula Margozzini, Rosu Maria-Magdalena, Joany Mariño, Anastasia Markaki, Larissa Pruner Marques, Jaume Marrugat, Reynaldo Martorell, Katharina Maruszczak, Giovanna Masala, Luis P Mascarenhas, Mannix Masimango Imani, Masoud Masinaei, Ellisiv B Mathiesen, Alicia Matijasevich, Piotr Matłosz, Tandi E Matsha, Victor Matsudo, Giletta Matteo, Pallab K Maulik, Christina Mavrogianni, Jean Claude N Mbanya, Anselmo J Mc Donald Posso, Shelly R McFarlane, Stephen T McGarvey, Rachael M McLean, Sounnia Mediene Benchekor, Kirsten Mehlig, Amir Houshang Mehrparvar, Jesus D Melgarejo, Fabián Méndez, Carlos O Mendivil, Carlos Mendoza Montano, Ana Maria B Menezes, Gert BM Mensink, Alibek Mereke, Indrapal I Meshram, Diane T Meto, Haakon E Meyer, Jie Mi, Karolina Miłkowska, Jody C Miller, Olga Milushkina, Cláudia S Minderico, GK Mini, Juan Francisco Miquel, J Jaime Miranda, Mohammad Reza Mirjalili, Marjeta Mišigoj-Duraković, Antonio Mistretta, Veronica Mocanu, Pietro A Modesti, Sahar Saeedi Moghaddam, Kazem Mohammad, Mohammad Reza Mohammadi, Zahra Mohammadi, Noushin Mohammadifard, Reza Mohammadpourhodki, Viswanathan Mohan, Muhammad Fadhli Mohd Yusoff, Iraj Mohebbi, Niels C Møller, Dénes Molnár, Amirabbas Momenan, Charles K Mondo, Michele M Monroy-Valle, Roger A Montenegro Mendoza, Eric Monterrubio-Flores, Kotsedi Daniel K Monyeki, Jin Soo Moon, Mahmood Moosazadeh, Farhad Moradpour, Leila B Moreira, Alain Morejon, Luis A Moreno, Karen Morgan, George Moschonis, Alireza Moslem, Mildrey Mosquera, Malgorzata Mossakowska, Aya Mostafa, Seyed-Ali Mostafavi, Mohammad Esmaeel Motlagh, Jorge Motta, Marcos André Moura-dos-Santos, Malay K Mridha, Kelias P Msyamboza, Thet Thet Mu, Florian Muca, Boban Mugoša, Patricia B Munroe, Jaakko Mursu, Kamarul Imran Musa, Sanja Musić Milanović, Vera Musil, Geofrey Musinguzi, Norlaila Mustafa, Muel Telo Marie-Claire Muyer, Iraj Nabipour, Balkish M Naidu, Farid Najafi, Hanna Nalecz, Jana Námešná, KM Venkat Narayan, Take Naseri, Michels Nathalie, Nareemarn Neelapaichit, Azim Nejatizadeh, Ilona Nenko, Flavio Nervi, Hannelore K Neuhauser, Tze Pin Ng, Chung T Nguyen, Quang V Nguyen, Quang Ngoc Nguyen, Michael Y Ni, Peng Nie, Ramfis E Nieto-Martínez, Teemu J Niiranen, Toshiharu Ninomiya, Nobuo Nishi, Sania Nishtar, Marianna Noale, Oscar A Noboa, Helena Nogueira, Kevin I Norton, Davide Noto, Natalia Nowak-Szczepanska, Mohannad Al Nsour, Irfan Nuhoğlu, Eha Nurk, Fred Nuwaha, Moffat Nyirenda, Terence W O’Neill, Caleb Ochimana, Angélica M Ochoa-Avilés, Eiji Oda, Augustine N Odili, Kyungwon Oh, Ryutaro Ohtsuka, Brian Oldenburg, Valérie Olié, Mohd Azahadi Omar, Saeed M Omar, Altan Onat, Sok King Ong, N Charlotte Onland-Moret, Lariane M Ono, Obinna Onodugo, Pedro Ordunez, Rui Ornelas, Ana P Ortiz, Pedro J Ortiz, Clive Osmond, Sergej M Ostojic, Afshin Ostovar, Johanna A Otero, Charlotte B Ottendahl, Akaninyene Otu, Kim Overvad, Ellis Owusu-Dabo, Cristina P Padez, Ioannis Pagkalos, Natalja Pajula, Alberto Palloni, Luigi Palmieri, Wen-Harn Pan, Francesco Panza, Mariela Paoli, Sousana K Papadopoulou, Rossina G Pareja, Soon-Woo Park, Suyeon Park, Winsome R Parnell, Mahboubeh Parsaeian, Ionela M Pascanu, Patrick Pasquet, Nikhil D Patel, Halyna Pavlyshyn, Raimund Pechlaner, Ivan Pećin, João M Pedro, Sergio Viana Peixoto, Markku Peltonen, Alexandre C Pereira, Karen GDA Peres, Marco A Peres, Agustín Perez-Londoño, Cynthia M Pérez, Valentina Peterkova, Olga Petrovna Kovtun, Niloofar Peykari, Son Thai Pham, Rafael N Pichardo, Preux Pierre-Marie, Hynek Pikhart, Aida Pilav, Pavel Piler, Aleksandra Piwonska, Andreia N Pizarro, Silvia Plata, Raluca M Pop, Barry M Popkin, Stevo R Popovic, Miquel Porta, Anil Poudyal, Farhad Pourfarzi, Akram Pourshams, Hossein Poustchi, Rajendra Pradeepa, Alison J Price, Antonio Prista, Rui Providencia, Jardena J Puder, Iveta Pudule, Soile Puhakka, Maria Puiu, Margus Punab, Mostafa Qorbani, Anna Quialheiro, Hedley K Quintana, Pedro J Quiroga-Padilla, Tran Quoc Bao, Stefan Rach, Salar Rahimikazerooni, Mahmudur Rahman, Olli Raitakari, Sherali Rakhmatulloev, Ivo Rakovac, Ambady Ramachandran, Otim PC Ramadan, Manuel Ramirez-Zea, Rafel Ramos, Lekhraj Rampal, Sanjay Rampal, Sheena E Ramsay, João FLB Rangel Junior, Daniel A Rangel Reina, Lalka S Rangelova, Vayia Rarra, Mohammad-Mahdi Rashidi, Cassiano Ricardo Rech, Josep Redon, Valéria Regecová, Jane DP Renner, Judit A Repasy, Cézane P Reuter, Luis Revilla, Andrew Reynolds, Negar Rezaei, Abbas Rezaianzadeh, Elio Riboli, Fernando Rigo, Attilio Rigotti, Leanne M Riley, Tobias F Rinke de Wit, Ulf Risérus, Raphael M Ritti-Dias, Reina G Roa, Romana Roccaldo, Fernando Rodríguez-Artalejo, María del Cristo Rodriguez-Perez, Laura A Rodríguez-Villamizar, Andrea Y Rodríguez, Ulla Roggenbuck, Peter Rohloff, Rosalba Rojas-Martinez, Elisabetta L Romeo, Rafaela V Rosario, Annika Rosengren, Ian Rouse, Adolfo Rubinstein, Blanca Sandra Ruiz-Betancourt, Maria Ruiz-Castell, Emma Ruiz Moreno, Iuliia A Rusakova, Wojciech Rusek, Petra Rust, Marcin Rutkowski, Marge Saamel, Hamideh Sabbaghi, Harshpal S Sachdev, Alireza Sadjadi, Ali Reza Safarpour, Sare Safi, Mohammad Hossien Saghi, Olfa Saidi, Calogero Saieva, Satoko Sakata, Nader Saki, Sanja Šalaj, Eduardo Salazar Martinez, Akkumis Salkhanova, Jukka T Salonen, Margarita Samoutian, Jose Sánchez-Abanto, Inés Sánchez Rodríguez, Diana A Santos, Ina S Santos, Maria Paula Santos, Tamara R Santos, Jouko L Saramies, Luis B Sardinha, Giselle Sarganas, Nizal Sarrafzadegan, Kai-Uwe Saum, Stefan Savin, Mariana Sbaraini, Marcia Scazufca, Beatriz D Schaan, Anja Schienkiewitz, Karin Schindler, Sabine Schipf, Amand Floriaan Schmidt, Börge Schmidt, Carsten O Schmidt, Ben Schöttker, Sara Schramm, Stine Schramm, Helmut Schröder, Constance Schultsz, Aletta E Schutte, Sylvain Sebert, Moslem Sedaghattalab, Aye Aye Sein, Abhijit Sen, Sadaf G Sepanlou, Guillermo Sequera, Ľudmila Ševčíková, Ronel Sewpaul, Teresa Shamah-Levy, Seyed Morteza Shamshirgaran, Maryam Sharafkhah, Sanjib K Sharma, Almaz Sharman, Jonathan E Shaw, Amaneh Shayanrad, Ali Akbar Shayesteh, Lela Shengelia, Kenji Shibuya, Hana Shimizu-Furusawa, Rahman Shiri, Marat Shoranov, Namuna Shrestha, Khairil Si-Ramlee, Abla M Sibai, Labros S Sidossis, Antonio M Silva, Caroline Ramos de Moura Silva, Diego Augusto Santos Silva, Kelly Samara Silva, Xueling Sim, Mary Simon, Michael Sjöström, Natalia A Skoblina, Jolanta Slowikowska-Hilczer, Przemysław Slusarczyk, Liam Smeeth, Lee Smith, Fernanda Cunha Soares, Grzegorz Sobek, Eugène Sobngwi, Morten Sodemann, Stefan Söderberg, Agustinus Soemantri, Vincenzo Solfrizzi, Mohammad Hossein Somi, Elin P Sørgjerd, Maroje Sorić, Victoria E Soto-Rojas, Aïcha Soumaré, Alfonso Sousa-Poza, Igor Spiroski, Jan A Staessen, Andreas Stang, Jostein Steene-Johannessen, Peter Stehle, Aryeh D Stein, George S Stergiou, Jakub Stokwiszewski, Ekaterina Stoyanova, Gareth Stratton, Karien Stronks, Lela Sturua, Milton F Suarez-Ortegón, Phalakorn Suebsamran, Gerhard Sulo, Johan Sundström, Paibul Suriyawongpaisal, Boyd A Swinburn, René Charles Sylva, Lucjan Szponar, E Shyong Tai, Konstantinos D Tambalis, Abdonas Tamosiunas, Baimakhan Tanabayev, Maya Tanrygulyyeva, Mohammed Rasoul Tarawneh, Jakob Tarp, Carolina B Tarqui-Mamani, Radka Taxová Braunerová, Saskia Te Velde, William R Tebar, Grethe S Tell, Tania Tello, KR Thankappan, Xenophon Theodoridis, Sathish Thirunavukkarasu, Nihal Thomas, Amanda G Thrift, Ľubica Tichá, Erik J Timmermans, Dwi Hapsari Tjandrarini, Anne Tjonneland, Janne S Tolstrup, Murat Topbas, Laura Torres-Collado, Giota Touloumi, Pierre Traissac, Areti Triantafyllou, Atul Trivedi, Lechaba Tshepo, Panagiotis Tsintavis, John Tuitele, Azaliia M Tuliakova, Marshall K Tulloch-Reid, Fikru Tullu, Tomi-Pekka Tuomainen, Maria L Turley, Evangelia Tzala, Themistoklis Tzotzas, Christophe Tzourio, Peter Ueda, Eunice Ugel, Flora AM Ukoli, Zhamyila Usupova, Hannu MT Uusitalo, Nalan Uysal, Gonzalo Valdivia, Damaskini Valvi, Rob M van Dam, Bert-Jan van den Born, Johan Van der Heyden, Yvonne T van der Schouw, Wendy Van Lippevelde, Hoang Van Minh, Natasja M Van Schoor, Irene GM van Valkengoed, Dirk Vanderschueren, Diego Vanuzzo, Gregorio Varela-Moreiras, Luz Nayibe Vargas, Senthil K Vasan, Daniel G Vasques, Tomas Vega, Gustavo Velasquez-Melendez, Biruta Velika, Charlotte Verdot, Maïté Verloigne, Giovanni Veronesi, WM Monique Verschuren, Roosmarijn Verstraeten, Lucie Viet, Frøydis N Vik, Monica Vilar, Salvador Villalpando, Jesus Vioque, Jyrki K Virtanen, Marjolein Visser, Bharathi Viswanathan, Mihaela Vladulescu, Henry Völzke, Ari Voutilainen, Martine Vrijheid, Alisha N Wade, Wan Mohamad Wan Bebakar, Wan Nazaimoon Wan Mohamud, Rildo de Souza Wanderley Júnior, Chongjian Wang, Huijun Wang, Ningli Wang, Qian Wang, Xiangjun Wang, Ya Xing Wang, Ying-Wei Wang, S Goya Wannamethee, Nicholas Wareham, Olivia Wartha, Adelheid Weber, Karen Webster-Kerr, Niels Wedderkopp, Daniel Weghuber, Wenbin Wei, Leo Westbury, Peter H Whincup, Kremlin Wickramasinghe, Kurt Widhalm, Indah S Widyahening, Andrzej Więcek, Rainford J Wilks, Karin Willeit, Peter Willeit, Julianne Williams, Tom Wilsgaard, Bogdan Wojtyniak, Roy A Wong-McClure, Andrew Wong, Emily B Wong, Mark Woodward, Frederick C Wu, Justyna Wyszyńska, Haiquan Xu, Liang Xu, Nor Azwany Yaacob, Li Yan, Weili Yan, Yang Yang, Martha Yépez García, Moein Yoosefi, Akihiro Yoshihara, Novie O Younger-Coleman, Yu-Ling Yu, Yunjiang Yu, Ahmad Faudzi Yusoff, Vassilis Zafiropulos, Ahmad A Zainuddin, Farhad Zamani, Sabina Zambon, Antonis Zampelas, Maria Elisa Zapata, Ko Ko Zaw, Tomasz Zdrojewski, Magdalena Żegleń, Kristyna Zejglicova, Tajana Zeljkovic Vrkic, Bing Zhang, Zhen-Yu Zhang, Yanitsa V Zhecheva, Bekbolat Zholdin, Paul Zimmet, Marie Zins, Julio Zuñiga Cisneros, Monika Zuziak

## Abstract

**Background:**

Adiposity can be measured using BMI (which is based on weight and height) as well as indices of abdominal adiposity. We examined the association between BMI and waist-to-height ratio (WHtR) within and across populations of different world regions and quantified how well these two metrics discriminate between people with and without hypertension.

**Methods:**

We used data from studies carried out from 1990 to 2023 on BMI, WHtR and hypertension in people aged 20–64 years in representative samples of the general population in eight world regions. We graphically compared the regional distributions of BMI and WHtR, and calculated Pearson’s correlation coefficients between BMI and WHtR within each region. We used mixed-effects linear regression to estimate the extent to which WHtR varies across regions at the same BMI. We graphically examined the prevalence of hypertension and the distribution of people who have hypertension both in relation to BMI and WHtR, and we assessed how closely BMI and WHtR discriminate between participants with and without hypertension using C-statistic and net reclassification improvement (NRI).

**Findings:**

The correlation between BMI and WHtR ranged from 0·76 to 0·89 within different regions. After adjusting for age and BMI, mean WHtR was highest in south Asia for both sexes, followed by Latin America and the Caribbean and the region of central Asia, Middle East and north Africa. Mean WHtR was lowest in central and eastern Europe for both sexes, in the high-income western region for women, and in Oceania for men. Conversely, to achieve an equivalent WHtR, the BMI of the population of south Asia would need to be, on average, 2·79 kg/m^2^ (95% CI 2·31–3·28) lower for women and 1·28 kg/m^2^ (1·02–1·54) lower for men than in the high-income western region. In every region, hypertension prevalence increased with both BMI and WHtR. Models with either of these two adiposity metrics had virtually identical C-statistics and NRIs for every region and sex, with C-statistics ranging from 0·72 to 0·81 and NRIs ranging from 0·34 to 0·57 in different region and sex combinations. When both BMI and WHtR were used, performance improved only slightly compared with using either adiposity measure alone.

**Interpretation:**

BMI can distinguish young and middle-aged adults with higher versus lower amounts of abdominal adiposity with moderate-to-high accuracy, and both BMI and WHtR distinguish people with or without hypertension. However, at the same BMI level, people in south Asia, Latin America and the Caribbean, and the region of central Asia, Middle East and north Africa, have higher WHtR than in the other regions.

**Funding:**

UK Medical Research Council and UK Research and Innovation (Innovate UK).

## Introduction

Adiposity can be measured using weight-to-height indices (eg, BMI) or indices of abdominal obesity such as waist circumference, waist-to-hip ratio (WHR), and waist-to-height ratio (WHtR). Some studies found that abdominal adiposity was a better predictor of cardio-metabolic outcomes than BMI, whereas others found similar associations. At any BMI level, the extent of abdominal adiposity can vary both within and across populations due to differences in fat and muscle mass and their distributions. For example, some studies have stated that, at any BMI level, Asian populations have higher levels of abdominal obesity than European and African populations.^1–5^ Current data on the relationship between BMI and abdominal obesity, and their association with health outcomes, typically cover one or a small number of countries or regions. Therefore, it has not been possible to systematically and consistently investigate how the relationship between BMI and abdominal obesity varies across global populations.^6^

We used a global database of population-based studies to quantify in a consistent way how BMI and WHtR are related within and across different world regions. We further examined the relationship of BMI and WHtR with hypertension and mean blood pressure, and measured how well the two adiposity indices predict prevalent hypertension. Through these analyses, we evaluated whether BMI can distinguish between those with lower versus higher WHtR, and predict the risk of having hypertension as well as WHtR.

## Methods

### Study design

We pooled and analysed population-based studies with data on height, weight, waist circumference, blood pressure, and use of antihypertensive medicines. We used BMI as an index of general adiposity and WHtR as an index of abdominal obesity. We used WHtR because it is a better predictor of cardiometabolic risk than waist circumference and WHR,^7–13^ due to accounting for height, which influences overall body size. This feature is particularly relevant in a global analysis, because height varies substantially across global populations.^14^ We show results using waist circumference in the appendix (pp 85–97, 104–109, 116–141) for comparison. We did not analyse WHR because some studies with data on waist circumference did not measure hip circumference.

We analysed hypertension as an outcome of obesity because it is a consequence of obesity and a leading global risk factor^15^ for cardiovascular disease, kidney disease, and dementia. Blood pressure is also more commonly measured in population-based studies^15^ than diabetes, cholesterol, and kidney disease, which require laboratory analysis. Furthermore, with data on systolic and diastolic blood pressure, hypertension could be defined consistently in all studies,^15^ whereas diabetes studies differ on whether they have data on fasting glucose or HbA_1c_.^16^ Pooled data were examined graphically and analysed using regression models as detailed below.

### Data

We used individual participant data from representative samples of the general population, collated by the NCD Risk Factor Collaboration (NCD-RisC), as detailed previously and in appendix (pp 35–36).^15,17^

We used data on adults aged 20–64 years from 837 studies with mid-year from 1990–2023. From each study, we used the data recorded in the sex variable. The studies might have varied in their protocol and terminology related to recording of sex or gender. To harmonise the data, we have used women and men throughout this paper. A list of data sources and their characteristics is provided in the appendix (pp 40–53). Studies from earlier years were not used because fewer studies had data on waist circumference. Older age groups were excluded because there were fewer participants in many regions. We had at least one data source for 181 countries (appendix pp 57–58), where 98·3% of the world population in 2023 lived. These countries were grouped into eight regions (appendix p 39). The number of studies per region ranged from 40 in Oceania to 189 in the high-income western region (appendix p 54). Of the 837 studies with data on BMI and WHtR, 710 (85%) also had data on hypertension. The other studies did not collect data on blood pressure as their primary intent and design. In particular, some population-based nutrition surveys collected anthropometric data but not blood pressure (appendix pp 40–53).

Details of data cleaning are provided in the appendix (pp 37, 59–60). After data cleaning, there remained 7·5 million participants aged 20–64 years with data on height, weight, and waist circumference; 5·4 million of these participants (72·2% of all participants with height, weight, and waist circumference data) also had data on blood pressure and hypertension treatment. In studies with data on blood pressure, the difference in mean BMI and WHtR of participants with and without blood pressure data was 0·49 kg/m^2^ and 0·01; the difference in mean age was 0·1 years.

When multiple measurements of blood pressure were taken, we removed the first measurement and averaged the remaining readings. Hypertension was defined as having systolic blood pressure (SBP) of 140 mmHg or greater, having diastolic blood pressure (DBP) of 90 mmHg or greater, or taking anti-hypertensive medication.^15^ We also used data on antihypertensive medicines to investigate whether the relationship between BMI or WHtR and blood pressure differed between participants who were treated for hypertension and those who were not. Treated hypertension was defined as current use of anti-hypertensive medication, regardless of blood pressure. Untreated hypertension was defined as having SBP 140 mmHg or greater or DBP 90 mm Hg or greater without the use of anti-hypertensive medication.

### Statistical analysis

**Figure 1:**
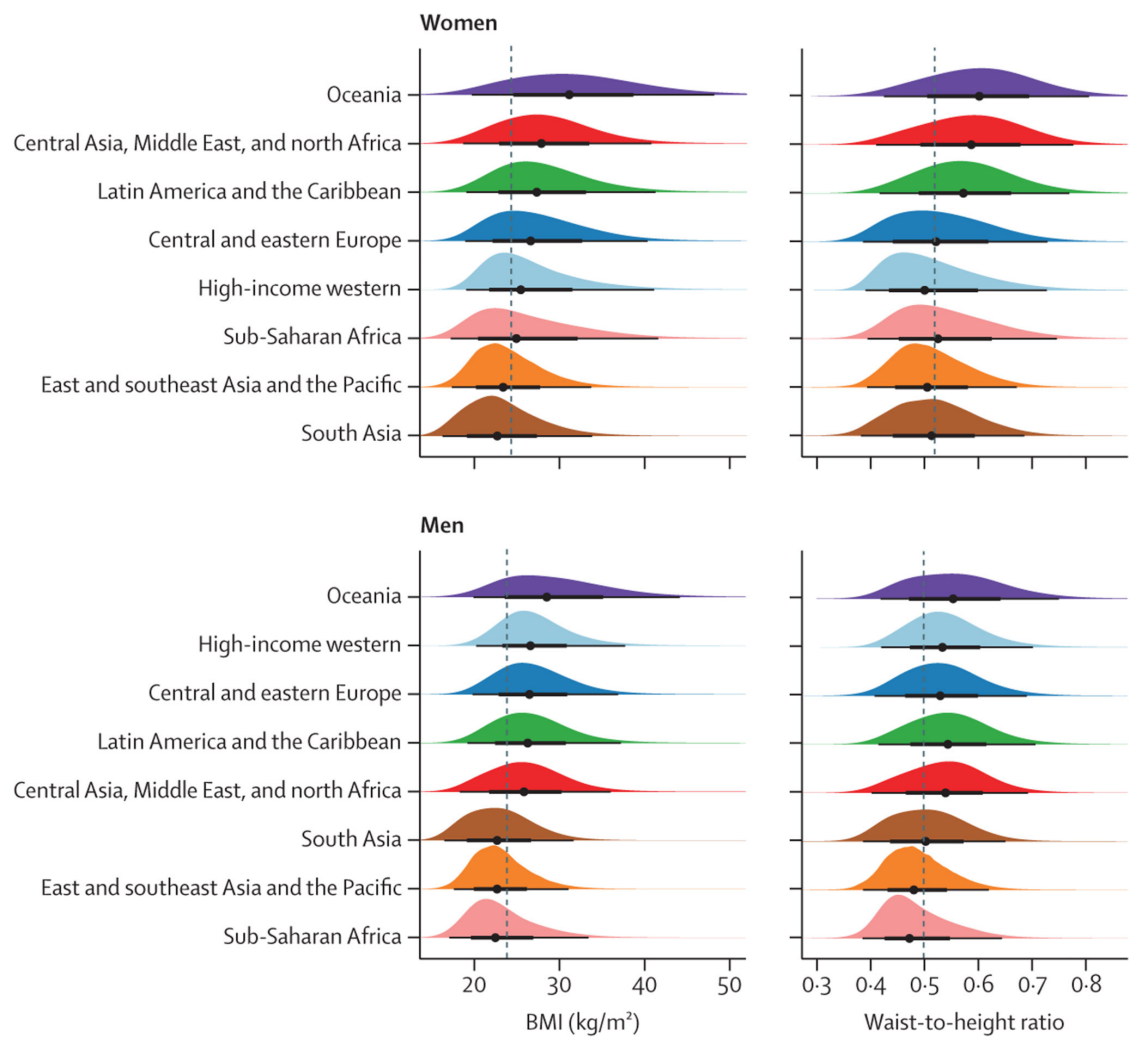
Distributions of BMI and waist-to-height ratio, by region The black lines below each distribution show the 2·5%, 25·0%, 75·0%, and 97·5% quantiles of the distributions and the points show the median. The dashed lines show medians across all participants. Regions are ordered by their sex-specific median BMI. See appendix (p 55) for numerical summaries.

**Figure 2:**
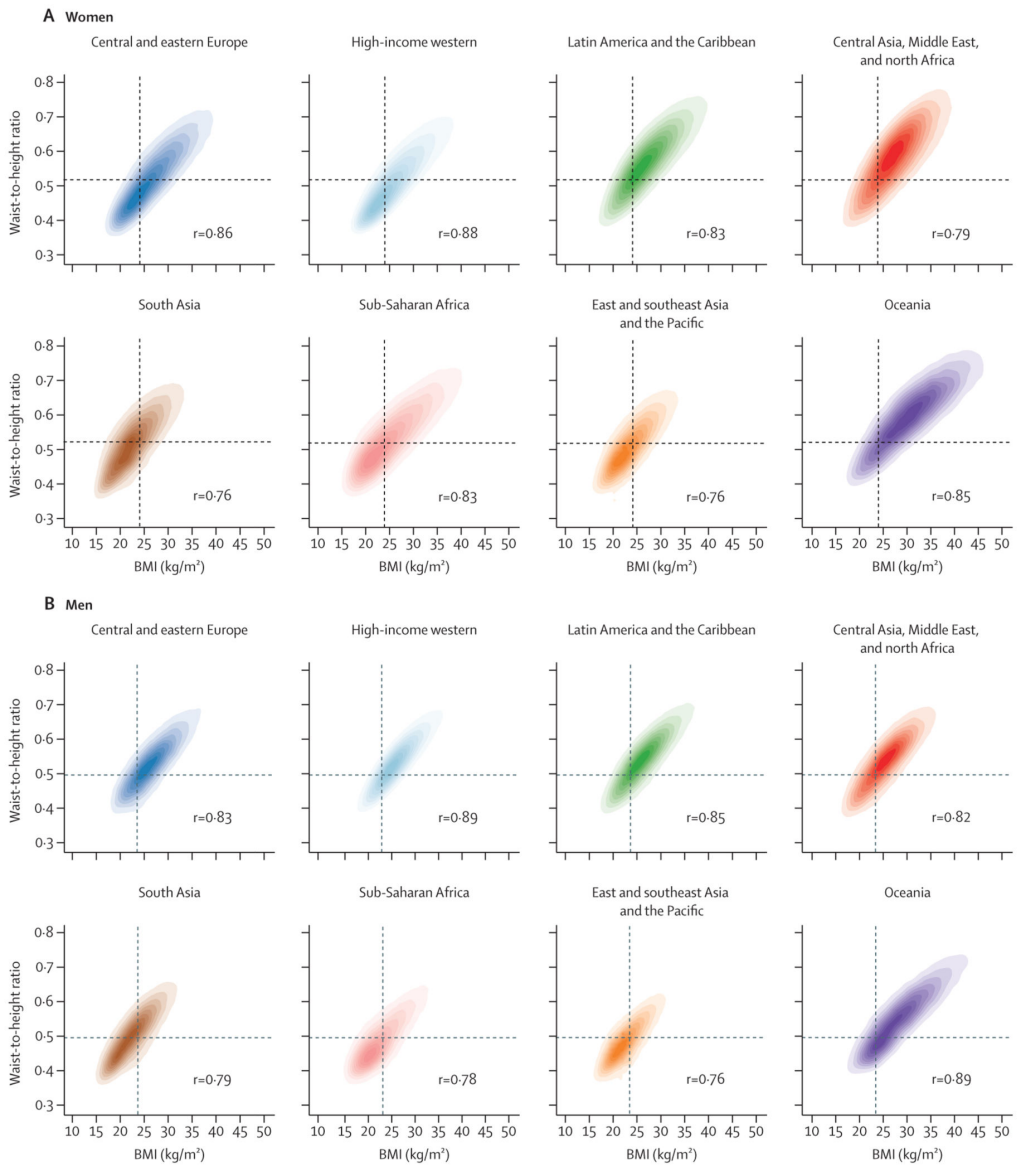
Relationship between waist-to-height ratio and BMI, by region The shading indicates the density of participants in each region, with darker shades corresponding to more participants and vice versa. Pearson correlation coefficients between BMI and waist-to-height ratio in each region are shown in the panels. The vertical dashed line shows median BMI for all participants globally, the horizontal dashed line shows median waist-to-height ratio for all participants globally. See appendix (pp 85–87) for results using waist circumference.

All analyses were done separately for men and women because there are differences in adiposity and hypertension between them.^15,17^ We graphically and numerically compared the regional distributions of BMI and WHtR to evaluate how they were distributed in each region relative to others (figure 1, appendix p 55). We calculated Pearson’s correlation coefficient between BMI and WHtR in each region to examine their relationship within regions (figure 2).

We used mixed-effects linear regression to estimate the extent to which WHtR varied across regions at any BMI level (appendix pp 61–62). The dependent variable was WHtR and the independent variables were BMI, age, and region. We adjusted for age because BMI and WHtR vary with age. We used age and BMI as linear terms, because their inclusion as categorical terms, with each category covering different age and BMI ranges, led to similar conclusions compared with linear terms for the BMI range that contained most participants (~17·5–47·5 kg/m^2^). The use of linear terms has the additional advantage of simplifying the presentation of results. Comparison of linear and categorical BMI terms at the global level indicates that at very high BMI levels, approximately 47·5 kg/m^2^ and higher, WHtR rises more slowly than predicted by linear BMI (appendix pp 63–64). The opposite happens at very low BMIs of around 17·5 kg/m^2^ and lower. We used random intercepts at the study level to account for unmeasured study level factors that might have influenced BMI or WHtR (these models are also known as multi-level, with individuals nested in studies).

**Figure 3:**
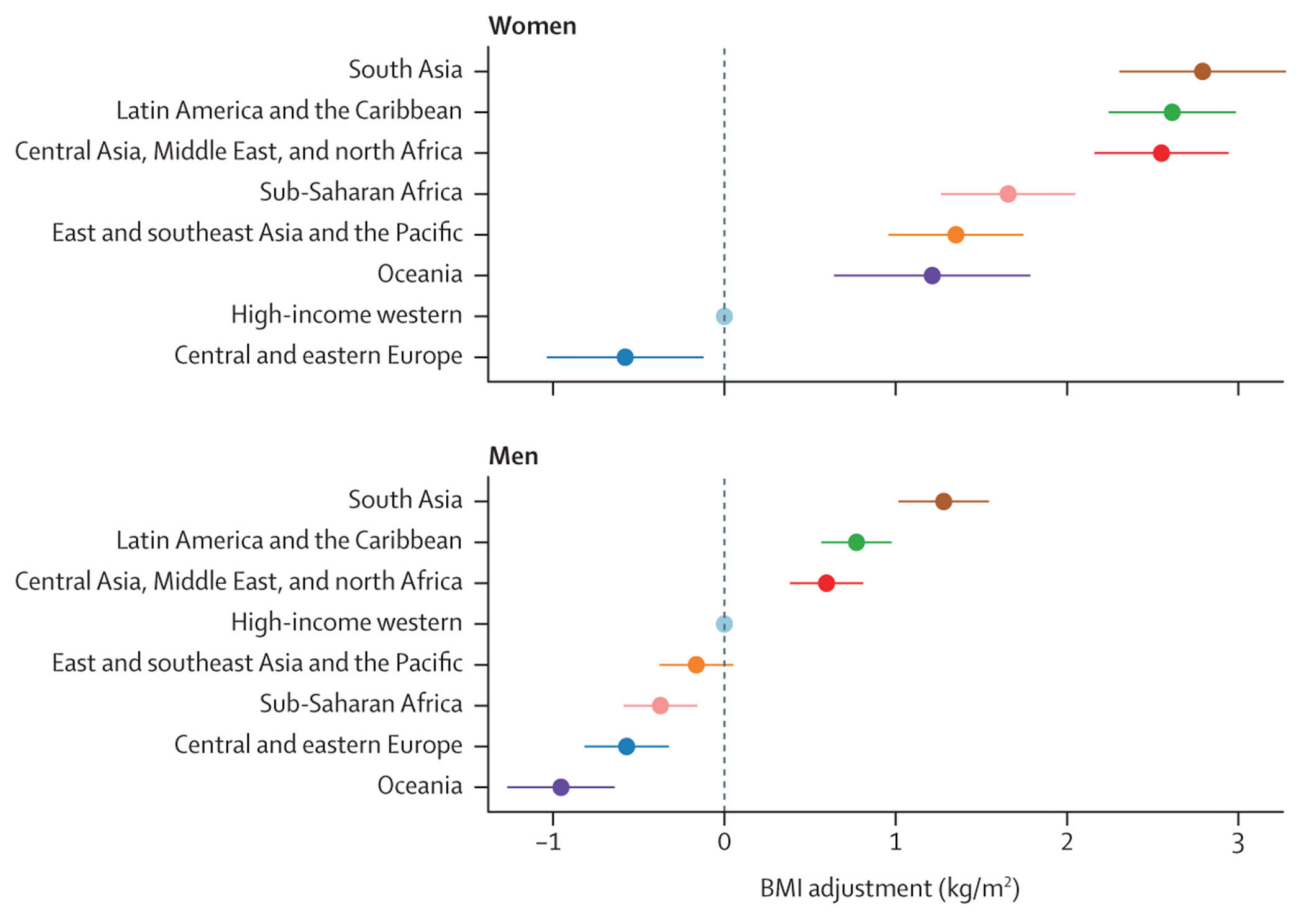
Regional BMI adjustment The BMI adjustment shows how much lower BMI in each region should be to achieve an equivalent waist-to-height ratio. The adjustment is shown relative to the population of the high-income western region where most current epidemiological studies have been done; regional ordering and differences across regions would be unchanged if a different reference were used. The bars show 95% CIs of the BMI adjustments. See appendix (pp 90–91) for results using waist circumference.

We report the difference across regions in WHtR at the sex-specific global mean BMI level. We used the regression coefficients to calculate how much lower or higher BMI would need to be for each region’s population to have the same WHtR; we refer to this quantity as regional BMI adjustment (figure 3). We estimated the 95% CIs of regional BMI adjustment based on the uncertainty of the model coefficients, accounting for the covariance matrix of the fixed effects.

**Figure 4:**
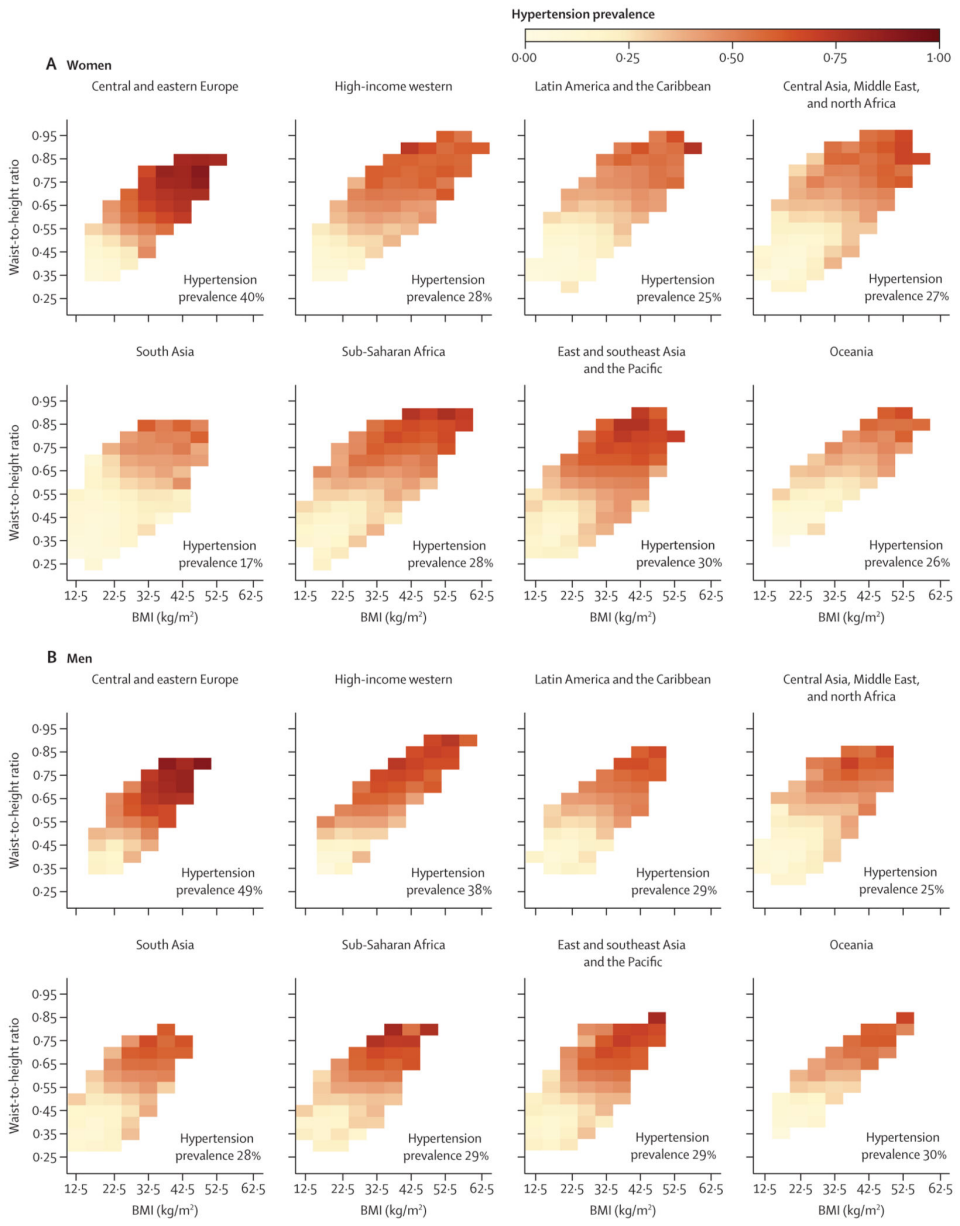
Prevalence of hypertension at different levels of waist-to-height ratio and BMI, by region Cells with 30 or fewer participants have been excluded from the figure because the results are less stable than at larger numbers. The number on each panel indicates the crude prevalence of hypertension among all participants in each region. See appendix for separate results for untreated and treated hypertension (pp 98–103) and for results using waist circumference (pp 92–94, 104–109).

**Figure 5:**
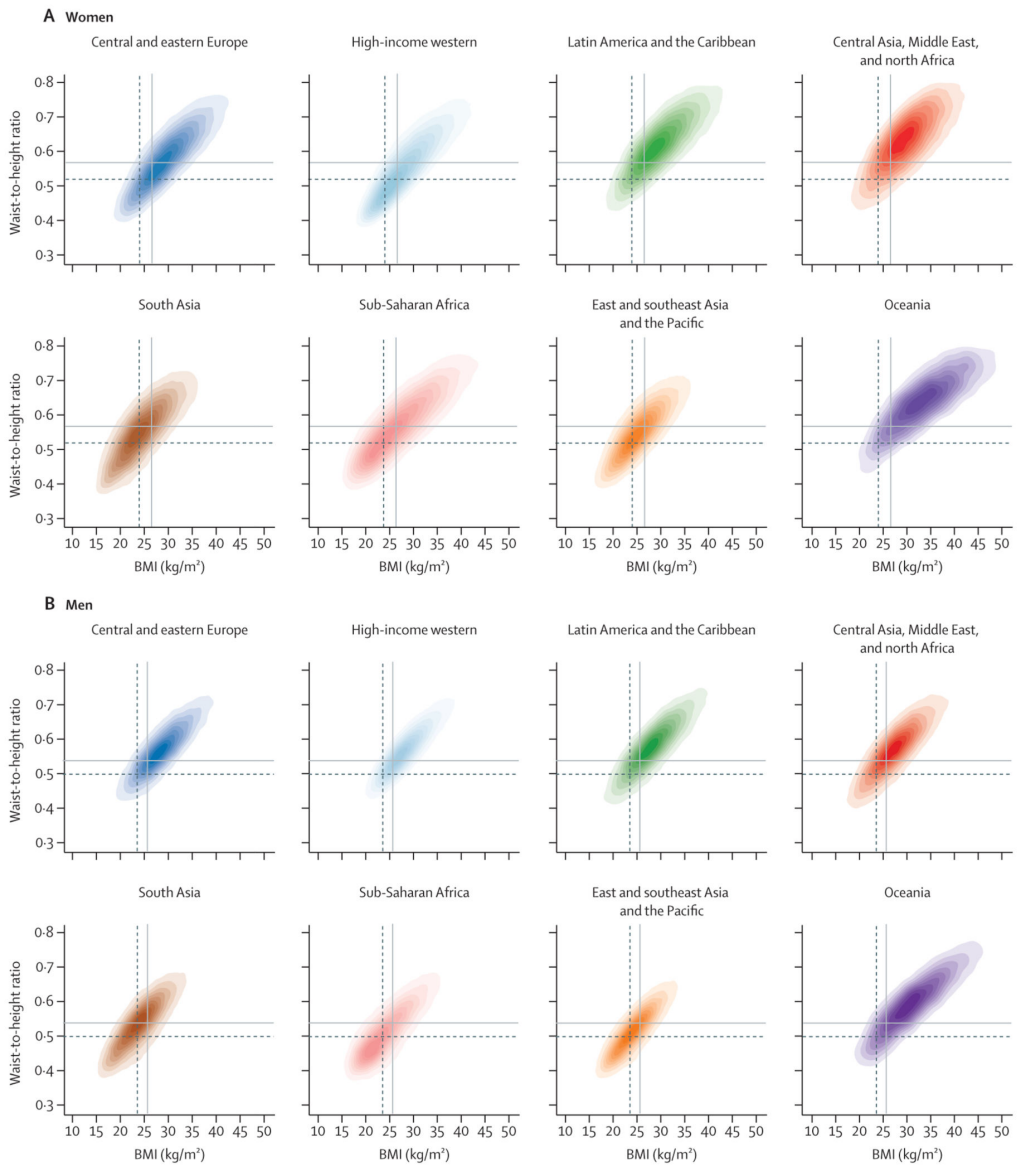
Distribution of participants with hypertension in relation to BMI and waist-to-height ratio, by region The shading indicates the density of participants with hypertension in each region, with darker shades corresponding to more participants. The vertical lines show median BMI and horizontal lines show median waist-to-height ratio for all participants (dashed lines) and those with hypertension (solid lines) globally. See appendix for separate results for untreated and treated hypertension (pp 110–115) and for results using waist circumference (pp 95–97, 116–121).

We graphically examined the prevalence of hypertension and the distribution of the number of people who have hypertension, both in relation to BMI and WHtR in each region. The first presentation quantifies the association of hypertension prevalence with BMI and WHtR, regardless of the distributions of BMI and WHtR in the regional population. The second presentation also takes into account the distributions of BMI and WHtR in the regional population (figures 4, 5).

We also assessed how well BMI and WHtR predict individuals’ hypertension status in each region using the C-statistic and the continuous net reclassification improvement (NRI) metrics. C-statistic measures the extent to which the use of BMI, WHtR, or both results in a participant with hypertension having a predicted probability higher than a participant without hypertension, whereas NRI measures the improvement made in the prediction of hypertension status by regression models with BMI, WHtR, or both, compared with a null model with neither. C-statistic and NRI were estimated from mixed effects logistic regressions that also included age, region, and study year, because hypertension prevalence varies by these factors.^15^ We included age as a linear term because exploratory analyses showed that the association with hypertension was linear for the age range of our analysis (20–64 years). For study year, we used categorical variables for 5-year intervals (1990–94, 1995–99, 2000–04, 2005–09, 2010–14, 2015–19, and 2020–23) because hypertension trends were nonlinear in some regions.^15^ We used interaction terms between region and age and between region and year to account for differences across regions in age associations and time trends. We tested but did not include an interaction between BMI and WHtR because its inclusion did not improve C-statistic or NRI. We also included study-level random intercepts to account for unobserved study level factors, such as sampling and measurement method, that might affect hypertension prevalence. In addition to C-statistic and NRI, we report the odds ratio (OR) for prevalent hypertension per SD of BMI and of WHtR without mutual adjustment, from regressions that had each metric alone; we also report ORs with mutual adjustment, from regressions that included both metrics.

All analyses were done using R version 4.3.2. The pooled analysis was approved by Imperial College London Research Ethics Committee and complies with all relevant ethical regulations. The participating studies followed their institutional approval and consent process.

### Role of the funding source

The funders of the study had no role in study design, data collection, data analysis, data interpretation, or writing of the paper.

## Results

The distributions of BMI and WHtR differed across regions and between sexes. For both sexes, BMI distributions in the three regions of south Asia, east and southeast Asia and the Pacific, and sub-Saharan Africa had lower medians and were shifted towards lower values compared with other regions (figure 1, appendix p 55). In contrast, WHtR distributions in these three regions overlapped more closely with those of other regions than was the case for their BMI distributions. For women, WHtR distribution in these three regions coincided closely with those of the high-income western region and central and eastern Europe. For men, the medians of the WHtR distributions of these three regions were ordered in the same way as for BMI, but the distribution in south Asia was more similar to other regions than was the case for BMI.

Oceania exhibited distributions of BMI with higher medians and quartiles compared with those in other regions. Oceania was followed by central Asia, Middle East and north Africa, and Latin America and the Caribbean for women, and by the high-income western region and central and eastern Europe for men. For women, the same regions also had the highest median WHtR, although Oceania’s WHtR distribution overlapped more closely with other regions than its BMI distribution. For men, the WHtR distributions were similar for the top five regions. In all regions, BMI and WHtR distributions of women had wider distributions than those of men.

The correlation coefficients between BMI and WHtR ranged from 0·76 to 0·89 in the two sexes across different regions—ie, within each region, BMI and WHtR were strongly, but not perfectly, correlated (figure 2). After adjusting for age and BMI, mean WHtR was highest in south Asia for both sexes, followed by Latin America and the Caribbean and the region of central Asia, Middle East, and north Africa (appendix pp 61–62). BMI-adjusted mean WHtR was lowest in central and eastern Europe for both sexes, high-income western region for women, and Oceania for men. The remaining regions showed no clear ordering for either sex. In all regions, after adjusting for age and BMI, mean WHtR was higher in women than in men.

To achieve an equivalent WHtR, those in south Asia need to have BMI that was, on average, 2·79 kg/m^2^ (95% CI 2·31–3·28) lower for women and 1·28 kg/m^2^ (1·02–1·54) lower for men than the population of the high-income western region (figure 3). The next largest BMI adjustments were for Latin America and the Caribbean (women: 2·61 [2·24–2·98] kg/m^2^, men: 0·77 [0·57–0·98] kg/m^2^) and the region of central Asia, Middle East, and north Africa (women: 2·55 [2·16–2·94] kg/m^2^, men: 0·60 [0·38–0·81] kg/m^2^). For both sexes, BMI adjustment for east and southeast Asia and the Pacific was smaller than that of south Asia, and for men its 95% CI contained the null value of zero. BMI adjustments to achieve the same WHtR were larger for women than men in all regions, although in central and eastern Europe the 95% CIs for the two sexes overlapped.

Although total hypertension prevalence varied across regions, in every region prevalence increased with both BMI and WHtR (figure 4). For low BMI (≤22·5 kg/m^2^) and WHtR (≤0·40), hypertension prevalence was 25% or less in all regions, except for men in sub-Saharan Africa where one BMI–WHtR combination within this range had a prevalence of 26%. In some BMI–WHtR region combinations in this range, prevalence was less than 15%. At high BMI (≥37·5 kg/m^2^) and WHtR (≥0·75), hypertension prevalence was 39% or higher for women and 49% or higher for men in all regions; in central and eastern Europe, it was 80% or higher for both men and women. The positive associations with BMI and WHtR were also seen for mean SBP and DBP among all participants (appendix pp 65–70) and in those who did not use anti-hypertensive medication (appendix pp 71–76). However, there was either no association or weak association between BMI or WHtR and mean SBP or DBP among participants on anti-hypertensive medication (appendix pp 77–82).

Models that included BMI alone and WHtR alone predicted hypertension status with similar C-statistics (0·74–0·78) and NRIs (0·41–0·44) when data from all regions were used (table). Regional models varied in their predictive performance, with higher C-statistics and NRIs in central and eastern Europe, the high-income western region, and Latin America and Caribbean for both sexes and in Oceania for men, and lower C-statistics and NRIs in south Asia and sub-Saharan Africa for both sexes. However, in every region, the ability to predict prevalent hypertension was virtually identical between models that included either adiposity measure (ie, similar C-statistic and NRI for models with either BMI or WHtR). Globally and within each region, including both BMI and WHtR led to little improvement in C-statistic and NRI compared with using either measure alone. Model C-statistics were consistently higher for women than men. ORs for both sexes for having hypertension per SD of BMI and WHtR were nearly identical (~1·7) without mutual adjustment (appendix pp 83–84). When both measures were included in the regression, BMI had a slightly larger OR per SD (1·39 [1·38–1·40] for BMI *vs* 1·33 [1·32–1·34] for WHtR for women; 1·38 [1·37–1·39] for BMI *vs* 1·32 [1·32–1·33] for WHtR for men).

The BMI and WHtR ranges where the density (ie, absolute number) of participants with hypertension was greatest varied across regions (figure 5). This variation occurred because the distributions of these two adiposity indices varied across regions (figures 1, 2). Specifically, in populations with lower BMI and WHtR distributions relative to other regions (both sexes in the east and southeast Asia and Pacific region; men in sub-Saharan Africa), more people with hypertension were concentrated in the lower range of BMI and WHtR, and vice versa (Oceania and the region of central Asia, Middle East, and north Africa). This finding is analogous to Rose’s phenomenon that most cases arise from ranges that contain the most people, not where the highest risk is. For example, 45% of men with hypertension in sub-Saharan Africa had a BMI of 25 kg/m^2^ or lower and a WHtR of 0·5 or lower. In contrast, in Oceania, this range accounted for only 10% of men with hypertension; rather, to reach a similar percentage of men with hypertension, BMI and WHtR would have to exceed around 30 kg/m^2^ and 0·65. In south Asia, people with hypertension were more concentrated towards lower BMI, but not WHtR, because in this region WHtR is higher at equivalent BMI levels compared with other regions (figure 2). In every region, density of those with treated hypertension was shifted towards higher BMI and WHtR compared with those who were not treated—ie, among participants with hypertension, those with higher BMI and WHtR were more likely to be treated than those with lower values (appendix pp 110–115).

## Discussion

In this global study, with data representative of the populations of all world regions, we found that BMI and WHtR were correlated at the individual level within all regions. In every region, BMI and WHtR predicted prevalent hypertension equally well. However, at any BMI level, WHtR was highest in south Asia, Latin America and the Caribbean, and central Asia, Middle East, and north Africa (especially for women), and lowest in central and eastern Europe and, for men, in Oceania.^18^

The main strength of our study is its global data and scope, which allowed comparison across regions. With a large number of studies and participants, we could robustly quantify how BMI and WHtR were associated both within and across regions, and how they were associated with hypertension prevalence. We maintained a high standard of data quality through careful checks of study sample and characteristics and, to avoid bias, we did not use self-reported data. Data were analysed according to a consistent protocol. Our study is also affected by limitations that apply to data pooling analyses, especially those that use data collected in different countries and time periods. Despite using substantially more data than previous studies, some regions, such as Oceania, had fewer studies, although even in this region we had data on 63 000 participants from 40 studies. Furthermore, we did not have sufficient data to include those older than 64 years, at which point BMI and WHtR might diverge due to age-related loss of muscle mass and decreases in height. We also did not have data on direct measures of body composition or fat distribution because such data require more complex measurements, which are not feasible in population surveillance, especially when health-care resources are limited. Although we only used studies with measurements of height, weight, and waist circumference following validated methods, unobserved differences might remain due to differing methods and implementation. We attempted to mitigate these differences by including study-level random effects in our models, which adjust for the influence of unobserved differences across studies. Finally, beyond hypertension, future research should investigate the similarities and differences in how BMI and abdominal adiposity are associated with conditions such as diabetes and kidney and liver disease in global populations.

That the populations of some regions had higher WHtR at similar BMI levels might be due to differences in genetics, smoking, diet, and exercise, which can differentially affect muscle and fat mass and their distributions.^19^ The differing WHtRs might also be due to phenotypic differences related to early-life nutrition, which affects body build, especially height and trunk height relative to leg height, which in turn influences the balance and distribution of lean and fatty body mass. In particular, populations in south Asia, where WHtR was higher than other regions at a similar BMI, have some of the lowest worldwide heights.^14^

The larger WHtR at any BMI level in women compared with men, and the variations in this sex difference across regions, could be due to at least three factors. First, body fat distribution is affected by sex hormones,^19^ which themselves vary across populations due to genetics, nutrition, environment, and social roles. Second, these regional sex differences could be due to differences in reproductive behaviours, including age at first child and total fertility rate, which might differentially affect waist circumference and weight.^20^ Finally, these differences might be due to sex differences in nutrition. The wider distributions of BMI and WHtR among women compared with men might additionally be because the social gradient and geographical variation in obesity is larger in women than men.^17,21,22^

The similarity of BMI and WHtR in predicting prevalent hypertension, which is consistent with studies in specific countries,^23–26^ could be due to two reasons. First, WHtR and BMI were correlated within regional populations. Second, the potential mechanisms for adiposity-induced hypertension, including insulin resistance, inflammation, and increased leptin leading to arterial stiffness; higher sympathetic nervous system activity; and activation of the renin-angiotensin-aldosterone system with increased sodium reabsorption might be similar for general and abdominal adiposity.^27,28^ The weak association of BMI and WHtR with blood pressure among those who used antihypertensive medicines could be because treatment weakens these mechanisms, and because patients with higher BMI and WHtR are more likely to be treated (appendix pp 110–115) and treated at higher doses or with a larger number of antihypertensive medicines.

The within-region correlations of BMI and WHtR indicate that, within regional populations, BMI can distinguish young and middle-aged adults with higher versus lower amounts of abdominal adiposity with moderate-to-high accuracy. Weight is also easier to measure than waist circumference in primary-care facilities and in population health surveys, and in some cultural settings it is more acceptable. Waist circumference might be measured with larger error. These features make BMI an appropriate, and in some settings preferred, metric for opportunistic risk screening and monitoring changes in obesity in primary care and in public health surveillance, especially if our results on the similarity of their performance in predicting hypertension are replicated for other conditions. Specialist obesity clinics will inevitably use a larger and more complex set of measures to decide on interventions, such as bariatric surgery and new obesity medicines to monitor patients’ outcomes. At the same time, the cross-region differences in the relationship indicate that some populations—especially those from south Asia—have higher amounts of abdominal adiposity at lower BMI levels, especially women. This finding might justify different thresholds for further screening if guideline committees consider that region-specific and sex-specific thresholds can be implemented in primary-care settings.

## Supplementary Material

Appendix

## Figures and Tables

**Table:** C-statistics and continuous NRIs for hypertension from three logistic models using BMI, waist-to-height ratio, and both

	Women								Men						
	C-statistic				NRI				C-statistic				NRI		
	BMI	Waist-to-height ratio	Both		BMI	Waist-to-height ratio	Both		BMI	Waist-to-height ratio	Both		BMI	Waist-to-height ratio	Both
Central and eastern Europe	0·810	0·810	0·813		0·568	0·560	0·581		0·765	0·763	0·767		0·467	0·458	0·481
High-income western	0·788	0·791	0·791		0·512	0·519	0·528		0·757	0·758	0·760		0·439	0·437	0·452
Latin America and the Caribbean	0·808	0·807	0·809		0·445	0·431	0·454		0·765	0·764	0·766		0·445	0·436	0·453
Central Asia, Middle East, and north Africa	0·791	0·791	0·793		0·395	0·400	0·414		0·755	0·753	0·757		0·405	0·405	0·420
South Asia	0·749	0·748	0·751		0·389	0·365	0·405		0·720	0·720	0·724		0·397	0·404	0·421
Sub-Saharan Africa	0·781	0·783	0·784		0·347	0·365	0·371		0·735	0·735	0·737		0·344	0·355	0·366
East and southeast Asia and the Pacific	0·755	0·752	0·757		0·456	0·437	0·471		0·718	0·715	0·721		0·433	0·420	0·446
Oceania	0·786	0·783	0·787		0·416	0·410	0·435		0·744	0·745	0·747		0·455	0·456	0·465
Global	0·780	0·779	0·782		0·440	0·427	0·454		0·741	0·740	0·744		0·421	0·414	0·434

NRI values were calculated relative to a logistic regression model using no adiposity measure. All models included terms for age and study year, and the global model included region. Regional models used only the data from that region. The appendix (p 56) shows results using waist circumference. NRI=net reclassification improvement.

## Data Availability

Data used in this research are governed by data-sharing protocols of participating studies. Contact information for data providers can be obtained from www.ncdrisc.org upon the publication of the paper. Computer code and meta-data for data sources used in the study are also available from the Zenodo repository (https://zenodo.org/records/12744326). Data governance and sharing agreements do not permit the sharing of individual participant data; contact information for data request is provided for all data sources used in the study.

## Research in context

### Evidence before this study

We searched PubMed for articles published from database inception up to March 27, 2024, with no language restrictions, using the following search terms: (“obesity”[mh:noexp] OR “overweight”[mh:noexp] OR “body size”[mh:noexp] OR “body height”[mh:noexp] OR “body weight”[mh:noexp] OR “thinness”[mh:noexp] OR “waist-hip ratio”[mh:noexp] or “waist circumference”[mh:noexp] or “body mass index” [mh:noexp]) AND (“humans”[mh]) AND (“health surveys”[mh] OR “epidemiological monitoring”[mh] OR “prevalence”[mh]) AND (“global^*^” OR “worldwide”) NOT “patient^*^”[Title] NOT comment[ptyp] NOT case reports[ptyp]. Articles were screened according to the inclusion and exclusion criteria described in the methods. We also reviewed the report of a WHO expert consultation on abdominal adiposity. We found studies either in one country or several countries that had compared BMI with different indices of abdominal adiposity, and had analysed their separate and combined associations, with incident or prevalent cardiometabolic outcomes. We also found one study that recruited patients from primary care facilities in 63 countries, and compared the associations of BMI and waist circumference with diagnosed cardiovascular disease and diabetes. However, in countries with limited health-care access and utilisation, a substantial proportion of people with these conditions might be undiagnosed. Some studies found that measures of abdominal adiposity were a better predictor of cardiometabolic outcomes than BMI, whereas others found similar associations. The classification of regions where the participants came from, or their ethnicity, varied across these studies, and many did not distinguish between different parts of Asia (eg, east, south, and west Asia). We did not find any population-based study that had participants from all world regions. A comparison of our findings with these studies is presented in the appendix (p 38).

### Added value of this study

To our knowledge, this is the first study that uses population-based data from all major regions of the world to evaluate how much BMI represents the extent of abdominal adiposity in different global populations, and measures how BMI and abdominal adiposity predict hypertension status, a major global cardiovascular risk. With numerous studies and millions of participants, we could consistently and robustly quantify these relationships in all world regions.

### Implications of all the available evidence

Within regional populations, BMI can distinguish individuals with higher versus lower amounts of abdominal adiposity, and is appropriate for opportunistic risk screening and monitoring changes in adiposity in surveillance and primary care. However, some populations, especially those from south Asia, have higher amounts of abdominal adiposity relative to other regions at equivalent BMI levels, especially women.
