# Supplementary material for: General and abdominal adiposity and hypertension in eight world regions: a pooled analysis of 837 population-based studies with 7·5 million participants
Source: Lancet. Author manuscript; Available in PMC 2024 Nov 5. (PMC7616775; doi:10.1016/S0140-6736(24)01405-3)
Supplement: Appendix [file EMS199576-supplement-Appendix.pdf]

THE LANCET

Supplementary appendix

This appendix formed part of the original submission and has been peer reviewed. We post it as supplied by the authors.

Supplement to: NCD Risk Factor Collaboration (NCD-RisC). General and abdominal adiposity and hypertension in eight world regions: a pooled analysis of 837 population-based studies with 7·5 million participants. *Lancet* 2024; **404:** 851–863.

Table of Contents

Appendix Text 1: NCD Risk Factor Collaboration (NCD-RisC) 4

Appendix Text 2: Data sources...35

Appendix Text 3: Data cleaning 37

Appendix Text 4: Comparison with previous studies..38

Appendix Table 1. List of analysis regions and countries in each region. 39

Appendix Table 2. Data sources used in the analysis...40

Appendix Table 3. Number of studies and participants aged 20-64 years, by region. 54

Appendix Table 4. Percentiles of waist-to-height ratio and body-mass index (BMI), by region. 55

Appendix Table 5. C-statistics and continuous net-reclassification improvement (NRI) for hypertension from three logistic models using body-mass index (BMI), waist circumference (WC), and both. 56

Appendix Figure 1: Number of studies with participants aged 20-64 years in each country. .57

Appendix Figure 2: Flowchart of data cleaning. 59

Appendix Figure 3: Waist-to-height ratio (WHtR) adjusted for body-mass index (BMI) and age, by region. 61

Appendix Figure 4: Comparison of waist-to-height ratio (WHtR) and BMI relationship using linear and categorical BMI terms. 63

Appendix Figure 5: Mean systolic blood pressure (SBP) at different levels of waist-to-height ratio (WHtR) and body-mass index (BMI), by region..65

Appendix Figure 6: Mean diastolic blood pressure (DBP) at different levels of waist-to-height ratio (WHtR) and body-mass index (BMI), by region..68

Appendix Figure 7: Mean systolic blood pressure (SBP) of participants who did not use anti-hypertensive medicines at different levels of waist-to-height ratio (WHtR) and body-mass index (BMI), by region. 71

Appendix Figure 8: Mean diastolic blood pressure (DBP) of participants who did not use anti-hypertensive medicines at different levels of waist-to-height ratio (WHtR) and body-mass index (BMI), by region. 74

Appendix Figure 9: Mean systolic blood pressure (SBP) of participants who used anti-hypertensive medicines at different levels of waist-to-height ratio (WHtR) and body-mass index (BMI), by region. 77

Appendix Figure 10: Mean diastolic blood pressure (DBP) of participants who used anti-hypertensive medicines at different levels of waist-to-height ratio (WHtR) and body-mass index (BMI), by region. 80

Appendix Figure 11: Odds ratio (OR) for prevalent hypertension per standard deviation (SD) of body-mass index (BMI) and of waist-to-height ratio (WHtR), with and without mutual adjustment....83

Appendix Figure 12: Relationship between waist circumference (WC) and body-mass index (BMI), by region....85

Appendix Figure 13: Waist circumference (WC) adjusted for body-mass index (BMI) and age, by region....88

Appendix Figure 14: Regional body-mass index (BMI) adjustment....90

Appendix Figure 15: Prevalence of hypertension at different levels of waist circumference (WC) and body-mass index (BMI), by region....92

Appendix Figure 16: Distribution of participants with hypertension in relation to body-mass index (BMI) and waist circumference (WC), by region....95

Appendix Figure 17: Prevalence of participants with hypertension who did not use anti-hypertensive medicines at different levels of waist-to-height ratio (WHtR) and body-mass index (BMI), by region....98

Appendix Figure 18: Prevalence of participants with hypertension who used anti-hypertensive medicines at different levels of waist-to-height ratio (WHtR) and body-mass index (BMI), by region....101

Appendix Figure 19: Prevalence of participants with hypertension who did not use anti-hypertensive medicines at different levels of waist circumference (WC) and body-mass index (BMI), by region....104

Appendix Figure 20: Prevalence of participants with hypertension who used anti-hypertensive medicines at different levels of waist circumference (WC) and body-mass index (BMI), by region....107

Appendix Figure 21: Distribution of participants with hypertension who did not use anti-hypertensive medicines in relation to body-mass index (BMI) and waist-to-height ratio (WHtR), by region....110

Appendix Figure 22: Distribution of participants with hypertension who used anti-hypertensive medicines in relation to body-mass index (BMI) and waist-to-height ratio (WHtR), by region....113

Appendix Figure 23: Distribution of participants with hypertension who did not use anti-hypertensive medicines in relation to body-mass index (BMI) and waist circumference (WC), by region....116

Appendix Figure 24: Distribution of participants with hypertension who used anti-hypertensive medicines in relation to body-mass index (BMI) and waist circumference (WC), by region. 119

Appendix Figure 25: Mean systolic blood pressure (SBP) at different levels of waist circumference (WC) and body-mass index (BMI), by region....122

Appendix Figure 26: Mean diastolic blood pressure (DBP) at different levels of waist circumference (WC) and body-mass index (BMI), by region....125

Appendix Figure 27: Mean systolic blood pressure (SBP) of participants who did not use anti-hypertensive medicines at different levels of waist circumference (WC) and body-mass index (BMI), by region. 128

Appendix Figure 28: Mean diastolic blood pressure (DBP) of participants who did not use anti-hypertensive medicines at different levels of waist circumference (WC) and body-mass index (BMI), by region. 131

Appendix Figure 29: Mean systolic blood pressure (SBP) of participants who used anti-hypertensive medicines at different levels of waist circumference (WC) and body-mass index (BMI), by region. 134

Appendix Figure 30: Mean diastolic blood pressure (DBP) of participants who used anti-hypertensive medicines at different levels of waist circumference (WC) and body-mass index (BMI), by region. 137

Appendix Figure 31: Odds ratio (OR) for prevalent hypertension per standard deviation (SD) of body-mass index (BMI) and of waist circumference (WC) with and without mutual adjustment. 140

References 142

Appendix Text 1: NCD Risk Factor Collaboration (NCD-RisC)

Pooled Analysis and Writing (* equal contribution)

Bin Zhou (PhD; Imperial College London, UK)*; James E Bennett (PhD; Imperial College London, UK)*; Aidan P Wickham (PhD; Imperial College London, UK)*; Rosie K Singleton (MSc; Imperial College London, UK); Anu Mishra (PhD; Bill & Melinda Gates Foundation, USA); Rodrigo M Carrillo-Larco (PhD; Emory University, USA); Nayu Ikeda (PhD; National Institutes of Biomedical Innovation, Health and Nutrition, Japan); Lakshya Jain (MSc; Imperial College London, UK); Ana Barradas-Pires (PhD; Imperial College London, UK); Rachel A Heap (MPH; Imperial College London, UK); Victor PF Lhoste (MSc; Imperial College London, UK); Kate E Sheffer (MSc; Imperial College London, UK); Nowell H Phelps (MMath; Imperial College London, UK); Archie W Rayner (MSci; Imperial College London, UK); Prof Edward W Gregg (PhD; Imperial College London, UK; RCSI University of Medicine and Health Sciences, Ireland); Prof Mark Woodward (PhD; University of New South Wales, Australia; Imperial College London, UK); Gretchen A Stevens (DSc; World Health Organization, Switzerland); Maria LC Iurilli (PhD; Imperial College London, UK); Prof Goodarz Danaei (ScD; Harvard TH Chan School of Public Health, USA); Prof Mariachiara Di Cesare (PhD; University of Essex, UK); Prof Carlos A Aguilar-Salinas (PhD; Instituto Nacional de Ciencias Médicas y Nutrición, Mexico); Noor Ani Ahmad (MPH; Ministry of Health, Malaysia); Prof Pascal Bovet (MD; Ministry of Health, Seychelles; Unisanté, Switzerland); Prof Zhengming Chen (DPhil; University of Oxford, UK); Prof Albertino Damasceno (PhD; Eduardo Mondlane University, Mozambique); Sarah L Filippi (PhD; Imperial College London, UK); Prof Imre Janszky (PhD; Norwegian University of Science and Technology, Norway); Prof Andre P Kengne (PhD; South African Medical Research Council, South Africa); Prof Young-Ho Khang (MD; Seoul National University College of Medicine, Republic of Korea); Prof Kamlesh Khunti (FMedSci; University of Leicester, UK); Prof Avula Laxmaiah (PhD; ICMR - National Institute of Nutrition, India); Lee-Ling Lim (PhD; University of Malaya, Malaysia); Prof Lauren Lissner (PhD; University of Gothenburg, Sweden); Paula Margozzini (MD; Pontificia Universidad Católica de Chile, Chile); Prof Jean Claude Mbanya (PhD; University of Yaoundé 1, Cameroon); Prof Stephen

McGarvey (PhD; Brown University, USA); Prof Jonathan E Shaw (MD; Baker Heart and Diabetes Institute, Australia); Prof Stefan Söderberg (PhD; Umeå University, Sweden); Luis Adrián Soto-Mota (PhD; Instituto Nacional de Ciencias Médicas y Nutrición, Mexico); Junyang Wang (PhD; Imperial College London, UK); Francesco Zaccardi (PhD; University of Leicester, UK); Prof Majid Ezzati (FMedSci; Imperial College London, UK; University of Ghana, Ghana)

Country and Regional Data (* equal contribution; listed alphabetically)

Leandra Abarca-Gómez (Caja Costarricense de Seguro Social, Costa Rica)*; Mohsen Abbasi-Kangevari (Non-Communicable Diseases Research Center, Iran)*; Shynar Abdrakhmanova (National Center of Public Health, Kazakhstan)*; Suhaila Abdul Ghaffar (Ministry of Health, Malaysia)*; Hanan F Abdul Rahim (Qatar University, Qatar)*; Zulfiya Abdurrahmonova (Ministry of Health and Social Protection, Tajikistan)*; Niveen M Abu-Rmeileh (Birzeit University, State of Palestine)*; Benjamin Acosta-Cazares (Instituto Mexicano del Seguro Social, Mexico)*; Ishag Adam (Qassim University, Saudi Arabia)*; Marzena Adamczyk (RehaKlinika, Poland)*; Wichai Aekplakorn (Mahidol University, Thailand)*; Imelda A Agdeppa (Food and Nutrition Research Institute, Philippines)*; Javad Aghazadeh-Attari (Urmia University of Medical Sciences, Iran)*; Charles Agyemang (University of Amsterdam, The Netherlands)*; Mohamad Hasnan Ahmad (Ministry of Health, Malaysia)*; Noor Ani Ahmad (Ministry of Health, Malaysia)*; Ali Ahmadi (Shahrekord University of Medical Sciences, Iran)*; Naser Ahmadi (Non-Communicable Diseases Research Center, Iran)*; Nastaran Ahmadi (Shahid Sadoughi University of Medical Sciences, Iran)*; Soheir H Ahmed (University of Hargeisa, Somaliland)*; Wolfgang Ahrens (Leibniz Institute for Prevention Research and Epidemiology - BIPS, Germany)*; Gulmira Aitmurzaeva (Republican Center for Health Promotion, Kyrgyzstan)*; Kamel Ajlouni (The National Center for Diabetes, Endocrinology and Genetics, Jordan)*; Hazzaa M Al-Hazzaa (Princess Nourah bint Abdulrahman University, Saudi Arabia)*; Halima Al-Hinai (Ministry of Health, Oman)*; Jawad A Al-Lawati (Ministry of Health, Oman)*; Rajaa Al-Raddadi (King Abdulaziz University, Saudi Arabia)*; Deena Al

Asfoor (World Health Organization Regional Office for the Eastern Mediterranean, Egypt)*; Huda M Al Hourani (The Hashemite University, Jordan)*; Monira Alarouj (Dasman Diabetes Institute, Kuwait)*; Fadia AlBuhairan (Aldara Hospital and Medical Center, Saudi Arabia)*; Shahla AlDhukair (King Abdullah International Medical Research Center, Saudi Arabia)*; Mohamed M Ali (World Health Organization, Switzerland)*; Anna V Alieva (Ministry of Health, Uzbekistan)*; Abdullah Alkandari (Dasman Diabetes Institute, Kuwait)*; Buthaina M Alkhatib (The Hashemite University, Jordan)*; Eman Aly (World Health Organization Regional Office for the Eastern Mediterranean, Egypt)*; Deepak N Amarapurkar (Bombay Hospital and Medical Research Centre, India)*; Pilar Amiano Etxezarreta (Departamento de Salud del Gobierno Vasco, Spain)*; Norbert Amougou (UMR CNRS-MNHN 7206, France)*; Lars Bo Andersen (Western Norway University of Applied Sciences, Norway)*; Sigmund A Anderssen (Norwegian School of Sport Sciences, Norway)*; Odysseas Androutsos (University of Thessaly, Greece)*; Ranjit Mohan Anjana (Madras Diabetes Research Foundation, India)*; Alireza Ansari-Moghaddam (Health Promotion Research Center, Iran)*; Elena Anufrieva (Yekaterinburg State Medical Academy, Russia)*; Hajer Aounallah-Skhiri (National Institute of Public Health, Tunisia)*; Tahir Aris (Ministry of Health, Malaysia)*; Raphael E Arku (University of Massachusetts Amherst, USA)*; Nimmathota Arlappa (ICMR - National Institute of Nutrition, India)*; Krishna K Aryal (University of Bergen, Norway)*; Felix K Assah (University of Yaoundé 1, Cameroon)*; Batyrbek Assembekov (Asfendiyarov Kazakh National Medical University, Kazakhstan)*; Maria Cecília F Assunção (Federal University of Pelotas, Brazil)*; Juha Auvinen (Oulu University Hospital, Finland; University of Oulu, Finland)*; Mária Avdičová (Regional Authority of Public Health, Slovakia)*; Kishwar Azad (Diabetic Association of Bangladesh, Bangladesh)*; Ana Azevedo (University of Porto, Portugal)*; Mohsen Azimi-Nezhad (Neyshabur University of Medical Sciences, Iran)*; Fereidoun Azizi (Research Institute for Endocrine Sciences, Iran)*; Flora Bacopoulou (National and Kapodistrian University of Athens, Greece)*; Suhad Bahijri (King Abdulaziz University, Saudi Arabia)*; Izet Bajramovic (University of Sarajevo, Bosnia and Herzegovina)*; Nagalla Balakrishna (ICMR - National Institute of Nutrition, India)*; Mohamed Bamoshmoosh (University of Science and Technology, Yemen)*;

Maciej Banach (Medical University of Lodz, Poland)*; Piotr Bandosz (Medical University of Gdańsk, Poland)*; José R Banegas (Universidad Autónoma de Madrid CIBERESP, Spain)*; Rafał Baran (University of Rzeszów, Poland)*; Carlo M Barbagallo (University of Palermo, Italy)*; Valter Barbosa Filho (Ceara State University, Brazil)*; Alberto Barceló (University of Miami, USA)*; Maja Baretić (University Hospital Centre Zagreb, Croatia)*; Joaquin Barnoya (Unidad de Cirugia Cardiovascular, Guatemala)*; Lena Barrera (Universidad del Valle, Colombia)*; Aluisio JD Barros (Federal University of Pelotas, Brazil)*; Mauro Virgílio Gomes Barros (University of Pernambuco, Brazil)*; Abdul Basit (Baqai Institute of Diabetology and Endocrinology, Pakistan)*; Joao Luiz Bastos (Simon Fraser University, Canada)*; Anwar M Batieha (Jordan University of Science and Technology, Jordan)*; Aline P Batista (Federal University of Ouro Preto, Brazil)*; Rosangela L Batista (Federal University of Maranhão, Brazil)*; Zhamilya Battakova (National Center of Public Health, Kazakhstan)*; Louise A Baur (University of Sydney, Australia)*; Pascal M Bayauli (Cliniques Universitaires de Kinshasa, DR Congo)*; Silvia Bel-Serrat (University College Dublin, Ireland)*; Antonisamy Belavendra (Christian Medical College Vellore, India)*; Habiba Ben Romdhane (University Tunis El Manar, Tunisia)*; Theodora Benedek (University of Medicine, Pharmacy, Sciences and Technology of Târgu-Mureș, Romania)*; Judith Benedics (Federal Ministry of Social Affairs, Health, Care and Consumer Protection, Austria)*; Mikhail Benet (Cafam University Foundation, Colombia)*; Gilda Estela Benitez Rolandi (Ministerio de Salud Pública y Bienestar Social, Paraguay)*; James E Bennett (Imperial College London, UK)*; Michaela Benzeval (University of Essex, UK)*; Elling Bere (University of Agder, Norway)*; Nicolas Berger (Sciensano, Belgium)*; Ingunn Holden Bergh (Norwegian Institute of Public Health, Norway)*; Salim Berkinbayev (Kazakh National Medical University, Kazakhstan)*; Antonio Bernabe-Ortiz (Universidad Científica del Sur, Peru)*; Heloísa Bettiol (University of São Paulo, Brazil)*; Augustin F Beybey (University of Yaoundé 1, Cameroon)*; Jorge Bezerra (University of Pernambuco, Brazil)*; Aroor Bhagyalaxmi (B J Medical College, India)*; Santosh K Bhargava (Sunder Lal Jain Hospital, India)*; Elysée Claude Bika Lele (Institute of Medical Research and Medicinal Plant Studies, Cameroon)*; Mukharram M Bikbov (Ufa Eye Research Institute, Russia)*; Bihungum

Bista (Nepal Health Research Council, Nepal)*; Dusko J Bjelica (University of Montenegro, Montenegro)*; Peter Bjerregaard (University of Southern Denmark, Denmark)*; Espen Bjertness (University of Oslo, Norway)*; Marius B Bjertness (University of Oslo, Norway)*; Cecilia Björkelund (University of Gothenburg, Sweden)*; Katia V Bloch (Federal University of Rio de Janeiro, Brazil)*; Anneke Blokstra (National Institute for Public Health and the Environment, The Netherlands)*; Simona Bo (University of Turin, Italy)*; Martin Bobak (University College London, UK)*; Lynne M Boddy (Liverpool John Moores University, UK)*; Bernhard O Boehm (Nanyang Technological University, Singapore)*; Jose G Boggia (Universidad de la República, Uruguay)*; Elena Bogova (National Medical Research Center for Endocrinology, Russia)*; Marialaura Bonaccio (IRCCS Neuromed, Italy)*; Alice Bonilla-Vargas (Caja Costarricense de Seguro Social, Costa Rica)*; Herman Borghs (KU Leuven, Belgium)*; Steve Botomba (Epidemiology Center of Diabetes, DR Congo)*; Rupert Bourne (Cambridge University Hospitals, UK)*; Pascal Bovet (Ministry of Health, Seychelles; Unisanté, Switzerland)*; Khadichamo Boymatova (World Health Organization Country Office, Tajikistan)*; Lutgart Braeckman (Ghent University, Belgium)*; Tasanee Braithwaite (King's College London, UK)*; Imperia Brajkovich (Universidad Central de Venezuela, Venezuela)*; Francesco Branca (World Health Organization, Switzerland)*; Hermann Brenner (German Cancer Research Center, Germany)*; Lizzy M Brewster (University of Amsterdam, The Netherlands)*; Yajaira Briceño (University of the Andes, Venezuela)*; Lacramioara Brinduse (Carol Davila University of Medicine and Pharmacy, Romania)*; Bettina Bringolf-Isler (Swiss Tropical and Public Health Institute, Switzerland; University of Basel, Switzerland)*; Miguel Brito (Instituto Politécnico de Lisboa, Portugal)*; Johannes Brug (National Institute for Public Health and the Environment, The Netherlands)*; Anna Bugge (University College Copenhagen, Denmark)*; Frank Buntinx (KU Leuven, Belgium)*; Marta Buoncristiano (World Health Organization Regional Office for Europe, Denmark)*; Con Burns (Munster Technological University, Ireland)*; Antonio Cabrera de León (Universidad de La Laguna, Spain)*; Roberta B Caixeta (Pan American Health Organization, USA)*; Tilema Cama (Ministry of Health, Tonga)*; Günay Can (Istanbul University - Cerrahpasa, Türkiye)*; Ana Paula C

Cândido (Federal University of Juiz de Fora, Brazil)*; Felicia Cañete (Ministry of Public Health, Paraguay)*; Mario V Capanzana (Food and Nutrition Research Institute, Philippines)*; Naděžda Čapková (National Institute of Public Health, Czech Republic)*; Eduardo Capuano (Canopo Study Center - Salerno, Italy)*; Rocco Capuano (Canopo Study Center - Salerno, Italy)*; Vincenzo Capuano (Canopo Study Center - Salerno, Italy)*; Viviane C Cardoso (University of São Paulo, Brazil)*; Axel C Carlsson (Karolinska Instituet, Sweden)*; Rodrigo M Carrillo-Larco (Emory University, USA)*; Felipe F Casanueva (Santiago de Compostela University, Spain)*; Maribel Casas (CIBERESP, Spain)*; Laura Censi (Council for Agricultural Research and Economics, Italy)*; Marvin Cervantes-Loaiza (Caja Costarricense de Seguro Social, Costa Rica)*; Parinya Chamnan (Sanpasitthiprasong Regional Hospital, Thailand)*; Snehalatha Chamukuttan (India Diabetes Research Foundation, India)*; Queenie Chan (Imperial College London, UK)*; Nish Chaturvedi (University College London, UK)*; Fangfang Chen (Capital Institute of Pediatrics, China)*; Huashuai Chen (Xiangtan University, China)*; Long-Sheng Chen (Ministry of Health and Welfare, Taiwan)*; Zhengming Chen (University of Oxford, UK)*; Yiling J Cheng (US Centers for Disease Control and Prevention, USA)*; Bahman Cheraghian (Ahvaz Jundishapur University of Medical Sciences, Iran)*; Angela Chetrit (The Gertner Institute for Epidemiology and Health Policy Research, Israel)*; Ekaterina Chikova-Iscener (National Center of Public Health and Analyses, Bulgaria)*; Mai JM Chinapaw (VU University Medical Center, The Netherlands)*; Anne Chinnock (Universidad de Costa Rica, Costa Rica)*; Arnaud Chiolero (University of Fribourg, Switzerland)*; Adela Chirita-Emandi (Victor Babes University of Medicine and Pharmacy Timisoara, Romania)*; María-Dolores Chirlaque (CIBERESP, Spain)*; Chean Lin Chong (RIPAS Hospital, Brunei)*; Diego G Christofaro (Universidade Estadual Paulista, Brazil)*; Jerzy Chudek (Medical University of Silesia, Poland)*; Renata Cifkova (Charles University, Czech Republic; Thomayer University Hospital, Czech Republic)*; Massimo Cirillo (University of Salerno, Italy)*; Frank Claessens (KU Leuven, Belgium)*; Philip Clare (University of Sydney, Australia)*; Emmanuel Cohen (UMR CNRS-MNHN 7206, France)*; Susana C Confortin (University of the Extreme South of Santa Catarina, Brazil)*; Tara C Coppinger (Munster Technological University, Ireland)*; Lilia

Yadira Cortés (Pontificia Universidad Javeriana, Colombia)*; Cojocaru R Cosmin (Carol Davila University of Medicine and Pharmacy, Romania)*; Simona Costanzo (IRCCS Neuromed, Italy)*; Melanie J Cowan (World Health Organization, Switzerland)*; Chris Cowell (University of Sydney, Australia)*; Amelia C Crampin (Malawi Epidemiology and Intervention Research Unit, Malawi)*; Amanda J Cross (Imperial College London, UK)*; Ana B Crujeiras (CIBEROBN, Spain)*; Juan J Cruz (Universidad Autónoma de Madrid CIBERESP, Spain)*; Alexandra M Cucu (National Institute of Public Health, Romania; University of Medicine and Pharmacy, Romania)*; Felipe V Cureau (Federal University of Rio Grande do Norte, Brazil)*; Sarah Cuschieri (University of Malta, Malta)*; Graziella D'Arrigo (National Research Council, Italy)*; Eleonora d'Orsi (Federal University of Santa Catarina, Brazil)*; Haroldo da Silva-Ferreira (Federal University of Alagoas, Brazil)*; Christina C Dahm (Aarhus University, Denmark)*; Jean Dallongeville (Institut Pasteur de Lille, France)*; Albertino Damasceno (Eduardo Mondlane University, Mozambique)*; Rachel Dankner (The Gertner Institute for Epidemiology and Health Policy Research, Israel)*; Kairat Davletov (Asfendiyarov Kazakh National Medical University, Kazakhstan)*; Francisco de Assis Guedes de Vasconcelos (Federal University of Santa Catarina, Brazil)*; Maria Alice Altenburg de Assis (Federal University of Santa Catarina, Brazil)*; Dirk De Bacquer (Ghent University, Belgium)*; Jaco De Bacquer (Ghent University, Belgium)*; Jeroen de Bont (Karolinska Institutet, Sweden)*; Amalia De Curtis (IRCCS Neuromed, Italy)*; Patrícia de Fragas Hinnig (Federal University of Santa Catarina, Brazil)*; Giovanni de Gaetano (IRCCS Neuromed, Italy)*; Stefaan De Henauw (Ghent University, Belgium)*; Pilar De Miguel-Etayo (CIBEROBN, Spain; University of Zaragoza, Spain)*; Paula Duarte de Oliveira (Federal University of Pelotas, Brazil)*; Karina Mary de Paiva (Federal University of Santa Catarina, Brazil)*; Karin De Ridder (Sciensano, Belgium)*; Marco Aurélio de Valois Correia Júnior (University of Pernambuco, Brazil)*; Mohan Deepa (Madras Diabetes Research Foundation, India)*; Vincent Jr DeGennaro (Innovating Health International, Haiti)*; Stefaan Demarest (Sciensano, Belgium)*; Elaine Dennison (University of Southampton, UK)*; Valérie Deschamps (The National Public Health Agency, France)*; Meghnath Dhimal (Nepal Health Research Council, Nepal)*; María Pilar Díez Ripollés (Consejería de Salud del

Gobierno de La Rioja, Spain)*; Zivka Dika (University of Zagreb, Croatia)*; Shirin Djalalinia (Ministry of Health and Medical Education, Iran)*; Liria Dominguez (Instituto de Investigación Nutricional, Peru)*; Maria Benedetta Donati (IRCCS Neuromed, Italy)*; Chiara Donfrancesco (Istituto Superiore di Sanità, Italy)*; Guanghui Dong (Sun Yat-sen University, China)*; Silvana P Donoso (Universidad de Cuenca, Ecuador)*; Maria Dorobantu (Carol Davila University of Medicine and Pharmacy, Romania)*; Marcus Dörr (University Medicine Greifswald, Germany)*; Nico Dragano (University Hospital Düsseldorf, Germany)*; Wojciech Drygas (Medical University of Lodz, Poland; Calisia University, Poland)*; Shufa Du (University of North Carolina at Chapel Hill, USA)*; Charmaine A Duante (Food and Nutrition Research Institute, Philippines)*; Priscilla Duboz (IRL 3189 ESS, France)*; Rosemary B Duda (Beth Israel Deaconess Medical Center, USA; Harvard Medical School, USA)*; Vesselka L Duleva (National Center of Public Health and Analyses, Bulgaria)*; Anar Dushpanova (Scuola Superiore Sant'Anna, Italy; Al-Farabi Kazakh National University, Kazakhstan)*; Azhar Dyussupova (Semey Medical University, Kazakhstan)*; Elzbieta Dziankowska-Zaborszczyk (Medical University of Lodz, Poland)*; Narges Ebrahimi (Non-Communicable Diseases Research Center, Iran)*; Guadalupe Echeverría (Pontificia Universidad Católica de Chile, Chile)*; Ricky Eddie (Ministry of Health and Medical Services, Solomon Islands)*; Ebrahim Eftekhar (Hormozgan University of Medical Sciences, Iran)*; Vasiliki Efthymiou (National and Kapodistrian University of Athens, Greece)*; Eruke E Egbagbe (University of Benin, Nigeria)*; Sareh Eghtesad (Tehran University of Medical Sciences, Iran)*; Ulf Ekelund (Norwegian School of Sport Sciences, Norway)*; Mohammad El-Khateeb (The National Center for Diabetes, Endocrinology and Genetics, Jordan)*; Jalila El Ati (National Institute of Nutrition and Food Technology, Tunisia)*; Roberto Elosua (Institut Hospital del Mar d'Investigacions Mèdiques, Spain; CIBERCV, Spain)*; Ofem Enang (University of Calabar, Nigeria)*; Rajiv T Erasmus (Stellenbosch University, South Africa)*; Cihangir Erem (Karadeniz Technical University, Türkiye)*; Gul Ergor (Dokuz Eylul University, Türkiye)*; Louise Eriksen (University of Southern Denmark, Denmark)*; Johan G Eriksson (University of Helsinki, Finland)*; Jorge Escobedo-de la Peña (Instituto Mexicano del Seguro Social, Mexico)*; Ali Esmaeili (Rafsanjan

University of Medical Sciences, Iran)*; Roger G Evans (Monash University, Australia)*; Ildar Fakhradiyev (Kazakh National Medical University, Kazakhstan)*; Albina A Fakhretdinova (Ufa Eye Research Institute, Russia)*; Caroline H Fall (University of Southampton, UK)*; Elnaz Faramarzi (Tabriz University of Medical Sciences, Iran)*; Mojtaba Farjam (Fasa University of Medical Sciences, Iran)*; Farshad Farzadfar (Non-Communicable Diseases Research Center, Iran)*; Yosef Farzi (Non-Communicable Diseases Research Center, Iran)*; Mohammad Reza Fattahi (Shiraz University of Medical Sciences, Iran)*; Asher Fawwad (Baqai Medical University, Pakistan)*; Francisco J Felix-Redondo (Centro de Salud Villanueva Norte, Spain)*; Trevor S Ferguson (The University of the West Indies, Jamaica)*; Romulo A Fernandes (Universidade Estadual Paulista, Brazil)*; Daniel Fernández-Bergés (Hospital Don Benito-Villanueva de la Serena, Spain)*; Desha R Fernando (University of Colombo, Sri Lanka)*; Daniel Ferrante (Ministry of Health, Argentina)*; Gerson Ferrari (Universidad de Santiago de Chile, Chile)*; Marika Ferrari (Council for Agricultural Research and Economics, Italy)*; Catterina Ferreccio (Pontificia Universidad Católica de Chile, Chile)*; Eldridge Ferrer (Food and Nutrition Research Institute, Philippines)*; Thamara Hubler Figueiró (Federal University of Santa Catarina, Brazil)*; Anna Fijalkowska (Institute of Mother and Child, Poland)*; Günther Fink (Swiss Tropical and Public Health Institute, Switzerland; University of Basel, Switzerland)*; Mauro Fisberg (Hospital Infantil Sabará, Brazil)*; Maria Forsner (Umeå University, Sweden)*; Edward F Fottrell (University College London, UK)*; Heba M Fouad (World Health Organization Regional Office for the Eastern Mediterranean, Egypt)*; Damian K Francis (The University of the West Indies, Jamaica)*; Guillermo Frontera (Hospital Universitario Son Espases, Spain)*; Flavio D Fuchs (Hospital de Clinicas de Porto Alegre, Brazil)*; Sandra C Fuchs (Federal University of Rio Grande do Sul, Brazil)*; Viktoriya Furdela (Ternopil National Medical University, Ukraine)*; Takuro Furusawa (Kyoto University, Japan)*; Stefan Adela Gabriela (Calafat Municipal Hospital, Romania)*; Zbigniew Gaciong (Medical University of Warsaw, Poland)*; Manuel Galán Cuesta (Consejería de Sanidad del Gobierno de Cantabria, Spain)*; Andrzej Galbarczyk (Jagiellonian University Medical College, Poland)*; Sonya V Galcheva (Medical University of Varna, Bulgaria)*; Myriam Galfo (Council for

Agricultural Research and Economics, Italy)*; Manoli Garcia-de-la-Hera (CIBERESP, Spain)*; Pablo Garcia (Wuqu' Kawoq, Guatemala)*; Sarah P Garnett (University of Sydney, Australia)*; Magda Gasull (CIBERESP, Spain)*; Andrea Gazzinelli (Federal University of Minas Gerais, Brazil)*; Ulrike Gehring (Utrecht University, The Netherlands)*; Eva Gerdts (University of Bergen, Norway)*; Ebrahim Ghaderi (Kurdistan University of Medical Sciences, Iran)*; Seyyed-Hadi Ghamari (Non-Communicable Diseases Research Center, Iran)*; Ali Ghanbari (Non-Communicable Diseases Research Center, Iran)*; Erfan Ghasemi (Non-Communicable Diseases Research Center, Iran)*; Oana-Florentina Gheorghe-Fronea (Carol Davila University of Medicine and Pharmacy, Romania)*; Anup Ghimire (B P Koirala Institute of Health Sciences, Nepal)*; Alessandro Gialluisi (IRCCS Neuromed, Italy; "Giuseppe Degennaro" LUM University, Italy)*; Simona Giampaoli (Istituto Superiore di Sanità, Italy)*; Francesco Gianfagna (University of Insubria, Italy; Mediterranea Cardiocentro, Italy)*; Glen Gironella (Food and Nutrition Research Institute, Philippines)*; Aleksander Giwercman (Lund University, Sweden)*; Konstantinos Gkiouras (Aristotle University of Thessaloniki, Greece)*; Natalya Glushkova (Al-Farabi Kazakh National University, Kazakhstan; Asfendiyarov Kazakh National Medical University, Kazakhstan)*; Ramesh Godara (Central University of Kerala, India)*; Justyna Godos (University of Catania, Italy)*; Marcel Goldberg (Institut National de la Santé et de la Recherche Médicale, France; Paris Cité University, France)*; Georgina Gómez (Universidad de Costa Rica, Costa Rica)*; Jesús Humberto Gómez Gómez (Instituto Murciano de Investigación Biosanitaria Virgen de la Arrixaca, Spain)*; Luis F Gomez (Pontificia Universidad Javeriana, Colombia)*; Santiago F Gómez (Gasol Foundation, Spain; University of Lleida, Spain)*; Aleksandra Gomula (PASs Hirszfeld Institute of Immunology and Experimental Therapy, Poland)*; Bruna Gonçalves Cordeiro da Silva (Federal University of Pelotas, Brazil)*; Helen Gonçalves (Federal University of Pelotas, Brazil)*; Mauer Gonçalves (University Agostinho Neto, Angola)*; Ana D González-Alvarez (Kansas State University, USA)*; David A Gonzalez-Chica (University of Adelaide, Australia)*; Esther M González-Gil (University of Zaragoza, Spain)*; Marcela Gonzalez-Gross (Universidad Politécnica de Madrid, Spain)*; Juan P González-Rivas (International Clinical Research Center, Czech Republic)*; Angel R

Gonzalez (Universidad Autónoma de Santo Domingo, Dominican Republic)*; Frederic Gottrand (University of Lille, France)*; Dušan Grafnetter (Institute for Clinical and Experimental Medicine, Czech Republic)*; Aneta Grajda (Children's Memorial Health Institute, Poland)*; Maria G Grammatikopoulou (University of Thessaly, Greece)*; Edward W Gregg (Imperial College London, UK; RCSI University of Medicine and Health Sciences, Ireland)*; Tomasz Grodzicki (Jagiellonian University Medical College, Poland)*; Else Karin Grøholt (Norwegian Institute of Public Health, Norway)*; Anders Grøntved (University of Southern Denmark, Denmark)*; Viviana Guajardo (International Life Science Institute, Argentina)*; Pilar Guallar-Castillón (Universidad Autónoma de Madrid CIBERESP, Spain)*; Maëlenn Guerchet (French National Research Institute for Sustainable Development, France)*; Ramiro Guerrero (Universidad Icesi, Colombia)*; Andre L Guimaraes (State University of Montes Claros, Brazil)*; Unjali P Gujral (Emory University, USA)*; Martin C Gulliford (King's College London, UK)*; Marc J Gunter (Imperial College London, UK)*; Rajeev Gupta (Eternal Heart Care Centre and Research Institute, India)*; Oye Gureje (University of Ibadan, Nigeria)*; Mirjana A Gurinović (Capacity Development Network in Nutrition in Central and Eastern Europe, Serbia)*; Beata Gurzkowska (Children's Memorial Health Institute, Poland)*; Laura Gutierrez (Institute for Clinical Effectiveness and Health Policy, Argentina)*; Xinyi Gwee (National University of Singapore, Singapore)*; Rosa Haghshenas (Non-Communicable Diseases Research Center, Iran)*; Hamid Hakimi (Rafsanjan University of Medical Sciences, Iran)*; Jytte Halkjær (Danish Cancer Institute, Denmark)*; Ian R Hambleton (The University of the West Indies, Barbados)*; Behrooz Hamzeh (Kermanshah University of Medical Sciences, Iran)*; Willem A Hanekom (Africa Health Research Institute, South Africa)*; Dominique Hange (University of Gothenburg, Sweden)*; Abu AM Hanif (BRAC James P Grant School of Public Health, Bangladesh)*; Sari Hantunen (University of Eastern Finland, Finland)*; Jie Hao (Capital Medical University, China)*; Carla Menêses Hardman (Federal University of Pernambuco, Brazil)*; Louise Hardy (University of Sydney, Australia)*; Rachakulla Hari Kumar (ICMR - National Institute of Nutrition, India)*; Javad Harooni (Yasuj University of Medical Sciences, Iran)*; Seyed Mohammad Hashemi-Shahri (Zahedan University Of Medical

Sciences, Iran)*; Maria Hassapidou (International Hellenic University, Greece)*; Jun Hata (Kyushu University, Japan)*; Teresa Haugsgjerd (University of Bergen, Norway)*; Mirjam Heinen (University College Dublin, Denmark)*; Marleen Elisabeth Hendriks (Joep Lange Institute, The Netherlands)*; Rafael dos Santos Henrique (Federal University of Pernambuco, Brazil)*; Ana Henriques (Institute of Public Health of the University of Porto, Portugal)*; Leticia Hernandez Cadena (National Institute of Public Health, Mexico)*; Sauli Herrala (Oulu University Hospital, Finland)*; Marianella Herrera-Cuenca (Universidad Central de Venezuela, Venezuela)*; Victor M Herrera (Universidad Autónoma de Bucaramanga, Colombia)*; Isabelle Herter-Aeberli (ETH Zurich, Switzerland)*; Karl-Heinz Herzig (University of Oulu, Finland; Oulu University Hospital, Finland)*; Ramin Heshmat (Chronic Diseases Research Center, Iran)*; Allan G Hill (University of Southampton, UK)*; Sai Yin Ho (University of Hong Kong, China)*; Michelle Holdsworth (French National Research Institute for Sustainable Development, France)*; Reza Homayounfar (Shahid Beheshti University of Medical Sciences, Iran)*; Clara Homs (Gasol Foundation, Spain; University Ramon Llull, Spain)*; Emiel O Hoogendijk (VU University Medical Center, The Netherlands)*; Andrea RVR Horimoto (University of São Paulo, Brazil)*; Claudia M Hormiga (Fundación Oftalmológica de Santander, Colombia)*; Bernardo L Horta (Federal University of Pelotas, Brazil)*; Leila Houti (University Oran 1, Algeria)*; Christina Howitt (The University of the West Indies, Barbados)*; Thein Thein Htay (Independent Public Health Specialist, Myanmar)*; Aung Soe Htet (University of Oslo, Norway)*; Maung Maung Than Htike (Ministry of Health and Sports, Myanmar)*; José María Huerta (CIBERESP, Spain)*; Ilpo Tapani Huhtaniemi (Imperial College London, UK)*; Laetitia Huiart (Santé publique France, France)*; Constanta Huidumac Petrescu (National Institute of Public Health, Romania)*; Martijn Huisman (Vrije Universiteit Amsterdam, The Netherlands)*; Abdullatif Husseini (Birzeit University, State of Palestine)*; Inge Huybrechts (International Agency for Research on Cancer, France)*; Nahla Hwalla (American University of Beirut, Lebanon)*; Licia Iacoviello (IRCCS Neuromed, Italy; "Giuseppe Degennaro" LUM University, Italy)*; Ellina M Iakupova (Ufa Eye Research Institute, Russia)*; Anna G Iannone (Gaetano Fucito Hospital, Italy)*; Jannicke Igland (University of Bergen, Norway)*; Chinwuba Ijoma

(University of Nigeria, Nigeria)*; Nayu Ikeda (National Institutes of Biomedical Innovation, Health and Nutrition, Japan)*; Violeta Iotova (Medical University of Varna, Bulgaria)*; Vilma E Irazola (Institute for Clinical Effectiveness and Health Policy, Argentina)*; Takafumi Ishida (The University of Tokyo, Japan)*; Godsent C Isiguzo (Alex Ekwueme Federal University Teaching Hospital, Nigeria)*; Muhammad Islam (The Hospital for Sick Children, Canada)*; Sheikh Mohammed Shariful Islam (Deakin University, Australia)*; Duygu Islek (Emory University, USA)*; Till Ittermann (University Medicine Greifswald, Germany)*; Ivaila Y Ivanova-Pandourska (Bulgarian Academy of Sciences, Bulgaria)*; Masanori Iwasaki (Hokkaido University, Japan)*; Tuija Jääskeläinen (Finnish Institute for Health and Welfare, Finland)*; Rod T Jackson (University of Auckland, New Zealand)*; Hashem Y Jaddou (Jordan University of Science and Technology, Jordan)*; Michel Jadoul (Université Catholique de Louvain, Belgium)*; Tazeen Jafar (Duke-NUS Medical School, Singapore)*; Nataša Jan (Slovenian Heart Foundation, Slovenia)*; Imre Janszky (Norwegian University of Science and Technology, Norway)*; Edward Janus (University of Melbourne, Australia)*; Juel Jarani (Sports University of Tirana, Albania)*; Gerald Jarnig (University of Graz, Austria)*; Marjo-Riitta Jarvelin (Imperial College London, UK; University of Oulu, Finland)*; Grazyna Jasienska (Jagiellonian University Medical College, Poland)*; Ana Jelaković (University Hospital Centre Zagreb, Croatia)*; Bojan Jelaković (University of Zagreb, Croatia)*; Anjani Kumar Jha (Nepal Health Research Council, Nepal)*; Ramon O Jimenez (Universidad Eugenio Maria de Hostos, Dominican Republic)*; Karl-Heinz Jöckel (University of Duisburg-Essen, Germany)*; Michel Joffres (Simon Fraser University, Canada)*; Jari J Jokelainen (Oulu University Hospital, Finland)*; Jost B Jonas (Institute of Molecular and Clinical Ophthalmology Basel, Switzerland)*; Pradeep Joshi (World Health Organization Country Office, India)*; Rohina Joshi (University of New South Wales, Australia)*; Josipa Josipović (University Hospital Centre Zagreb, Croatia)*; Farahnaz Joukar (Guilan University of Medical Sciences, Iran)*; Jacek J Jóźwiak (University of Opole, Poland)*; Anne Juolevi (Finnish Institute for Health and Welfare, Finland)*; Vesna Juresa (University of Zagreb, Croatia)*; Vesna Jureša (University of Zagreb, Croatia)*; Rudolf Kaaks (German Cancer Research Center, Germany)*; Felix O Kaducu (Gulu

University, Uganda)*; Agnes L Kadvan (Capacity Development Network in Nutrition in Central and Eastern Europe, Serbia)*; Anthony Kafatos (University of Crete, Greece)*; Eero O Kajantie (Finnish Institute for Health and Welfare, Finland)*; Natia Kakutia (National Center for Disease Control and Public Health, Georgia)*; Daniela Kállayová (Ministry of Health, Slovakia)*; Zhanna Kalmatayeva (Al-Farabi Kazakh National University, Kazakhstan)*; Ofra Kalter-Leibovici (The Gertner Institute for Epidemiology and Health Policy Research, Israel)*; Srinivasan Kannan (Sree Chitra Tirunal Institute for Medical Sciences and Technology, India)*; Efthymios Kapantais (Hellenic Medical Association for Obesity, Greece)*; Eva Karaglani (Harokopio University, Greece)*; Argyro Karakosta (National and Kapodistrian University of Athens, Greece)*; Khem B Karki (Maharajgunj Medical Campus, Nepal)*; Adoubi Kassi Anicet (University of Bouaké, Côte d'Ivoire)*; Marzieh Katibeh (Aarhus University, Denmark)*; Prasad Katulanda (University of Colombo, Sri Lanka)*; Peter T Katzmarzyk (Pennington Biomedical Research Center, USA)*; Jussi Kauhanen (University of Eastern Finland, Finland)*; Gyulli M Kazakbaeva (Ufa Eye Research Institute, Russia)*; François F Kaze (University of Yaoundé 1, Cameroon)*; Calvin Ke (University of Toronto, Canada)*; Sirkka Keinänen-Kiukaanniemi (Oulu University Hospital, Finland)*; Roya Kelishadi (Isfahan University of Medical Sciences, Iran)*; Cecily Kelleher (University College Dublin, Ireland)*; Han CG Kemper (VU University Medical Center, The Netherlands)*; Andre P Kengne (South African Medical Research Council, South Africa)*; Maryam Keramati (Mashhad University of Medical Sciences, Iran)*; Mathilde Kersting (Research Institute of Child Nutrition, Germany)*; Yousef Saleh Khader (Jordan University of Science and Technology, Jordan)*; Arsalan Khaledifar (Shahrekord University of Medical Sciences, Iran)*; Davood Khalili (Shahid Beheshti University of Medical Sciences, Iran)*; Young-Ho Khang (Seoul National University College of Medicine, Republic of Korea)*; Bahareh Kheiri (Shahid Beheshti University of Medical Sciences, Iran)*; Motahareh Kheradmand (Mazandaran University of Medical Sciences, Iran)*; Alireza Khosravi (Hypertension Research Center, Iran)*; Ursula Kiechl-Kohlendorfer (Medical University of Innsbruck, Austria)*; Sophia J Kiechl (VASCage – Research Centre on Vascular Ageing and Stroke, Austria)*; Stefan Kiechl (Medical University of Innsbruck, Austria; VASCage –

Research Centre on Vascular Ageing and Stroke, Austria)*; Hyeon Chang Kim (Yonsei University College of Medicine, Republic of Korea)*; Heidi Klakk (University College South Denmark, Denmark)*; Suntara Klanarong (Thaksin University, Thailand; deceased)*; Jana Klanova (Masaryk University, Czech Republic)*; Magdalena Klimek (Jagiellonian University Medical College, Poland)*; Michael Knoflach (Medical University of Innsbruck, Austria)*; Susanne Kobel (Ulm University Hospital, Germany)*; Bhawesh Koirala (B P Koirala Institute of Health Sciences, Nepal)*; Elin Kolle (Norwegian School of Sport Sciences, Norway)*; Patrick Kolsteren (Ghent University, Belgium)*; Jürgen König (University of Vienna, Austria)*; Raija Korpelainen (University of Oulu, Finland)*; Paul Korrovits (Tartu University Clinics, Estonia)*; Magdalena Korzycka (Institute of Mother and Child, Poland)*; Jelena Kos (University Hospital Centre Zagreb, Croatia)*; Seppo Koskinen (Finnish Institute for Health and Welfare, Finland)*; Malik Koussoh Simone (National Institute of Public Health, Côte d'Ivoire)*; Éva Kovács (Hildburghausen District Department of State Public Health Service, Germany)*; Irina Kovalskys (Pontificia Universidad Católica Argentina, Argentina)*; Sudhir Kowlessur (Ministry of Health and Wellness, Mauritius)*; Slawomir Koziel (PASs Hirszfeld Institute of Immunology and Experimental Therapy, Poland)*; Jana Kratenova (National Institute of Public Health, Czech Republic)*; Wolfgang Kratzer (University Hospital Ulm, Germany)*; Susi Kriemler (University of Zurich, Switzerland)*; Peter Lund Kristensen (University of Southern Denmark, Denmark)*; Helena Krizan (Croatian Institute of Public Health, Croatia)*; Maria F Kroker-Lobos (Institute of Nutrition of Central America and Panama, Guatemala)*; Steinar Krokstad (Norwegian University of Science and Technology, Norway)*; Herculina S Kruger (North-West University, South Africa; South African Medical Research Council, South Africa)*; Ruan Kruger (North-West University, South Africa)*; Łukasz Kryst (University of Physical Education, Poland)*; Ruzena Kubinova (National Institute of Public Health, Czech Republic)*; Urho M Kujala (University of Jyväskylä, Finland)*; Enisa Kujundzic (Institute of Public Health, Montenegro)*; Zbigniew Kulaga (Children's Memorial Health Institute, Poland)*; Mukhtar Kulimbet (Al-Farabi Kazakh National University, Kazakhstan; Asfendiyarov Kazakh National Medical University, Kazakhstan)*; Meena Kumari (University of Essex, UK)*; Marie Kunešová

(Institute of Endocrinology, Czech Republic)*; Pawel Kurjata (National Institute of Cardiology, Poland)*; Catherine Kyobutungi (African Population and Health Research Center, Kenya)*; Quang Ngoc La (Hanoi University of Public Health, Vietnam)*; Demetre Labadarios (Stellenbosch University, South Africa; University of Limpopo, South Africa)*; Carl Lachat (Ghent University, Belgium)*; Daphne Lai (Universiti Brunei Darussalam, Brunei)*; Youcef Laid (Ministry of Health, Algeria)*; Lachmie Lall (Ministry of Health, Guyana)*; Maritza Landaeta Jimenez (Universidad Central de Venezuela, Venezuela)*; Edwige Landais (French National Research Institute for Sustainable Development, France)*; Tiina Lankila (Oulu Deaconess Institute Foundation, Finland)*; Vera Lanska (Institute for Clinical and Experimental Medicine, Czech Republic)*; Georg Lappas (Sahlgrenska Academy, Sweden)*; Bagher Larijani (Endocrinology and Metabolism Research Center, Iran)*; Mina P Lateva (Medical University of Varna, Bulgaria)*; Tint Swe Latt (University of Public Health, Myanmar)*; Martino Laurenzi (Centro Studi Epidemiologici di Gubbio, Italy)*; Avula Laxmaiah (ICMR - National Institute of Nutrition, India)*; Maria Lazo-Porras (Universidad Peruana Cayetano Heredia, Peru)*; Gwenaëlle Le Coroller (Luxembourg Institute of Health, Luxembourg)*; Khanh Le Nguyen Bao (National Institute of Nutrition, Vietnam)*; Terho Lehtimäki (Tampere University Hospital, Finland; Tampere University, Finland)*; Daniel Lemogoum (University of Douala, Cameroon)*; Elvynna Leong (Universiti Brunei Darussalam, Brunei)*; Justyna Leszczak (University of Rzeszów, Poland)*; Gabriel M Leung (University of Hong Kong, China)*; Yanping Li (Harvard TH Chan School of Public Health, USA)*; Merike Liivak (National Institute for Health Development, Estonia)*; Charlie Lim (National University of Singapore, Singapore; National University Health System, Singapore)*; Wei-Yen Lim (National University of Singapore, Singapore; National University Health System, Singapore)*; M Fernanda Lima-Costa (Fundação Oswaldo Cruz, Brazil)*; Hsien-Ho Lin (National Taiwan University, Taiwan)*; Lars Lind (Uppsala University, Sweden)*; Lauren Lissner (University of Gothenburg, Sweden)*; Mieczyslaw Litwin (Children's Memorial Health Institute, Poland)*; Liping Liu (Beijing Center for Disease Prevention and Control, China)*; Xiaotian Liu (Zhengzhou University, China)*; Guadalupe Longo Abril (Servicio Andaluz de Salud, Spain)*; Oscar Lopes (Sports Medical

Center of Minho, Portugal)*; Esther Lopez-Garcia (Universidad Autónoma de Madrid CIBERESP, Spain)*; José Francisco López-Gil (Universidad de Las Américas, Ecuador)*; Tania Lopez (Universidad San Martín de Porres, Peru)*; José Eugenio Lozano (Consejería de Sanidad Junta de Castilla y León, Spain)*; Janice L Lukrafka (Federal University of Health Sciences of Porto Alegre, Brazil)*; Dalia Luksiene (Lithuanian University of Health Sciences, Lithuania)*; Annamari Lundqvist (Finnish Institute for Health and Welfare, Finland)*; Nuno Lunet (University of Porto, Portugal)*; Charles Lunogelo (Ilembula Lutheran Hospital, Tanzania)*; Michala Lustigová (Charles University, Czech Republic; National Institute of Public Health, Czech Republic)*; Jean-René M'Buyamba-Kabangu (University of Kinshasa Hospital, DR Congo)*; George LL Machado-Coelho (Federal University of Ouro Preto, Brazil)*; Aristides M Machado-Rodrigues (University of Coimbra, Portugal)*; Enguerran Macia (UMR 7268 ADES, France)*; Ahmed A Madar (University of Oslo, Norway)*; Gladys E Maestre (University of Texas Rio Grande Valley, USA)*; Stefania Maggi (Institute of Neuroscience of the National Research Council, Italy)*; Dianna J Magliano (Baker Heart and Diabetes Institute, Australia)*; Sara Magnacca (IRCCS Neuromed, Italy)*; Emmanuella Magriplis (Agricultural University of Athens, Greece)*; Gowri Mahasampath (Christian Medical College Vellore, India)*; Bernard Maire (French National Research Institute for Sustainable Development, France)*; Marcia Makdisse (Academia VBHC, Brazil)*; Mohammad-Reza Malekpour (Non-Communicable Diseases Research Center, Iran)*; Fatemeh Malekzadeh (Tehran University of Medical Sciences, Iran)*; Reza Malekzadeh (Shiraz University of Medical Sciences, Iran; Tehran University of Medical Sciences, Iran)*; Kodavanti Mallikharjuna Rao (ICMR - National Institute of Nutrition, India)*; Sofia Malyutina (Institute of Internal and Preventive Medicine, Russia)*; Lynell V Maniego (Food and Nutrition Research Institute, Philippines)*; Yannis Manios (Harokopio University, Greece)*; Jim I Mann (University of Otago, New Zealand)*; Fariborz Mansour-Ghanaei (Guilan University of Medical Sciences, Iran)*; Enzo Manzato (University of Padua, Italy)*; Mala Ali Mapatano (University of Kinshasa, DR Congo)*; Paula Margozzini (Pontificia Universidad Católica de Chile, Chile)*; Rosu Maria-Magdalena (Clinical County Emergency Hospital Craiova, Romania)*; Joany Mariño (University Medicine Greifswald,

Germany)*; Anastasia Markaki (Hellenic Mediterranean University, Greece)*; Larissa Pruner Marques (Fundação Oswaldo Cruz, Brazil)*; Jaume Marrugat (CIBERCV, Spain; Institut Hospital del Mar d'Investigacions Mèdiques, Spain)*; Reynaldo Martorell (Emory University, USA)*; Katharina Maruszczak (Paracelsus Medical University, Austria)*; Giovanna Masala (Institute for Cancer Reseach, Prevention and Clinical Network, Italy)*; Luis P Mascarenhas (Universidade Estadual do Centro-Oeste, Brazil)*; Mannix Masimango Imani (Université Catholique de Bukavu, DR Congo)*; Masoud Masinaei (Non-Communicable Diseases Research Center, Iran)*; Ellisiv B Mathiesen (UiT The Arctic University of Norway, Norway)*; Alicia Matijasevich (University of São Paulo, Brazil)*; Piotr Matłosz (University of Rzeszów, Poland)*; Tandi E Matsha (Sefako Makgatho Health Sciences University, South Africa)*; Victor Matsudo (Centro de Estudos do Laboratório de Aptidão Física de São Caetano do Sul, Brazil)*; Giletta Matteo (Ghent University, Belgium)*; Pallab K Maulik (George Institute for Global Health, India)*; Christina Mavrogianni (Harokopio University, Greece)*; Jean Claude N Mbanya (University of Yaoundé 1, Cameroon)*; Anselmo J Mc Donald Posso (Instituto Conmemorativo Gorgas de Estudios de la Salud, Panama)*; Shelly R McFarlane (The University of the West Indies, Jamaica)*; Stephen T McGarvey (Brown University, USA)*; Rachael M McLean (University of Otago, New Zealand)*; Sounnia Mediene Benchekor (University Oran 1, Algeria)*; Kirsten Mehlig (University of Gothenburg, Sweden)*; Amir Houshang Mehrparvar (Shahid Sadoughi University of Medical Sciences, Iran)*; Jesus D Melgarejo (University of Texas Rio Grande Valley, USA)*; Fabián Méndez (Universidad del Valle, Colombia)*; Carlos O Mendivil (Universidad de los Andes, Colombia)*; Carlos Mendoza Montano (Institute of Nutrition of Central America and Panama, Guatemala)*; Ana Maria B Menezes (Federal University of Pelotas, Brazil)*; Gert BM Mensink (Robert Koch Institute, Germany)*; Alibek Mereke (Al-Farabi Kazakh National University, Kazakhstan)*; Indrapal I Meshram (ICMR - National Institute of Nutrition, India)*; Diane T Meto (University of Abidjan, Côte d'Ivoire)*; Haakon E Meyer (University of Oslo, Norway)*; Jie Mi (Capital Institute of Pediatrics, China)*; Karolina Miłkowska (Jagiellonian University Medical College, Poland)*; Jody C Miller (University of Otago, New Zealand)*; Olga Milushkina (Pirogov Russian National

Research Medical University, Russia)*; Cláudia S Minderico (Universidade de Lisboa, Portugal)*; GK Mini (Saveetha Institute of Medical and Technical Sciences, India)*; Juan Francisco Miquel (Pontificia Universidad Católica de Chile, Chile)*; J Jaime Miranda (University of Sydney, Australia)*; Mohammad Reza Mirjalili (Shahid Sadoughi University of Medical Sciences, Iran)*; Marjeta Mišigoj-Duraković (University of Zagreb, Croatia)*; Antonio Mistretta (University of Catania, Italy)*; Veronica Mocanu (Grigore T Popa University of Medicine and Pharmacy, Romania)*; Pietro A Modesti (Università degli Studi di Firenze, Italy)*; Sahar Saeedi Moghaddam (Non-Communicable Diseases Research Center, Iran)*; Kazem Mohammad (Tehran University of Medical Sciences, Iran)*; Mohammad Reza Mohammadi (Psychiatry and Psychology Research Center, Iran)*; Zahra Mohammadi (Tehran University of Medical Sciences, Iran)*; Noushin Mohammadifard (Isfahan Cardiovascular Research Center, Iran)*; Reza Mohammadpourhodki (Mashhad University of Medical Sciences, Iran)*; Viswanathan Mohan (Madras Diabetes Research Foundation, India)*; Muhammad Fadhli Mohd Yusoff (Ministry of Health, Malaysia)*; Iraj Mohebbi (Urmia University of Medical Sciences, Iran)*; Niels C Møller (University of Southern Denmark, Denmark)*; Dénes Molnár (University Medical School of Pécs, Hungary)*; Amirabbas Momenan (Shahid Beheshti University of Medical Sciences, Iran)*; Charles K Mondo (Mulago Hospital, Uganda)*; Michele M Monroy-Valle (Universidad de San Carlos, Guatemala)*; Roger A Montenegro Mendoza (Gorgas Memorial Institute for Studies of Health, Panama)*; Eric Monterrubio-Flores (National Institute of Public Health, Mexico)*; Kotsedi Daniel K Monyeki (University of Limpopo, South Africa)*; Jin Soo Moon (Seoul National University, Republic of Korea)*; Mahmood Moosazadeh (Mazandaran University of Medical Sciences, Iran)*; Farhad Moradpour (Kurdistan University of Medical Sciences, Iran)*; Leila B Moreira (Federal University of Rio Grande do Sul, Brazil)*; Alain Morejon (University of Medical Sciences of Cienfuegos, Cuba)*; Luis A Moreno (University of Zaragoza, Spain; CIBEROBN, Spain)*; Karen Morgan (RCSI University of Medicine and Health Sciences, Ireland)*; George Moschonis (La Trobe University, Australia)*; Alireza Moslem (Sabzevar University Of Medical Sciences, Iran)*; Mildrey Mosquera (Universidad del Valle, Colombia)*; Malgorzata Mossakowska (International Institute of

Molecular and Cell Biology, Poland)*; Aya Mostafa (Ain Shams University, Egypt)*; Seyed-Ali Mostafavi (Tehran University of Medical Sciences, Iran)*; Mohammad Esmaeel Motlagh (Ahvaz Jundishapur University of Medical Sciences, Iran)*; Jorge Motta (Instituto Conmemorativo Gorgas de Estudios de la Salud, Panama)*; Marcos André Moura-dos-Santos (University of Pernambuco, Brazil)*; Malay K Mridha (BRAC James P Grant School of Public Health, Bangladesh)*; Kelias P Msyamboza (World Health Organization Country Office, Malawi)*; Thet Thet Mu (Department of Public Health, Myanmar)*; Florian Muca (Albanian Sport Science Association, Albania)*; Boban Mugoša (Institute of Public Health, Montenegro)*; Patricia B Munroe (Queen Mary University, UK)*; Jaakko Mursu (University of Eastern Finland, Finland)*; Kamarul Imran Musa (Universiti Sains Malaysia, Malaysia)*; Sanja Musić Milanović (Croatian Institute of Public Health, Croatia; University of Zagreb, Croatia)*; Vera Musil (University of Zagreb, Croatia)*; Geofrey Musinguzi (Makerere University School of Public Health, Uganda)*; Norlaila Mustafa (Universiti Kebangsaan Malaysia, Malaysia)*; Muel Telo Marie-Claire Muyer (University de Kinshasa, DR Congo)*; Iraj Nabipour (Bushehr University of Medical Sciences, Iran)*; Balkish M Naidu (Research and Methodology, Department of Statistics, Malaysia)*; Farid Najafi (Kermanshah University of Medical Sciences, Iran)*; Hanna Nalecz (Jozef Pilsudski University of Physical Education in Warsaw, Poland)*; Jana Námešná (Regional Authority of Public Health, Slovakia)*; KM Venkat Narayan (Emory University, USA)*; Take Naseri (Ministry of Health, Samoa)*; Michels Nathalie (Ghent University, Belgium)*; Nareemarn Neelapaichit (Mahidol University, Thailand)*; Azim Nejatizadeh (Hormozgan University of Medical Sciences, Iran)*; Ilona Nenko (Jagiellonian University Medical College, Poland)*; Flavio Nervi (Pontificia Universidad Católica de Chile, Chile)*; Hannelore K Neuhauser (Robert Koch Institute, Germany)*; Tze Pin Ng (National University of Singapore, Singapore)*; Chung T Nguyen (National Institute of Hygiene and Epidemiology, Vietnam)*; Quang V Nguyen (National Hospital of Endocrinology, Vietnam)*; Quang Ngoc Nguyen (Hanoi Medical University, Vietnam)*; Michael Y Ni (University of Hong Kong, China)*; Peng Nie (Xi'an Jiaotong University, China)*; Ramfis E Nieto-Martínez (Precision Care Clinic Corp, USA)*; Teemu J Niiranen (Finnish Institute for Health and Welfare, Finland; University

of Turku, Finland)*; Toshiharu Ninomiya (Kyushu University, Japan)*; Nobuo Nishi (National Institutes of Biomedical Innovation, Health and Nutrition, Japan)*; Sania Nishtar (Heartfile, Pakistan)*; Marianna Noale (Institute of Neuroscience of the National Research Council, Italy)*; Oscar A Noboa (Universidad de la República, Uruguay)*; Helena Nogueira (University of Coimbra, Portugal)*; Kevin I Norton (University of South Australia, Australia)*; Davide Noto (University of Palermo, Italy)*; Natalia Nowak-Szczepanska (PASs Hirszfeld Institute of Immunology and Experimental Therapy, Poland)*; Mohannad Al Nsour (Eastern Mediterranean Public Health Network, Jordan)*; Irfan Nuhoğlu (Karadeniz Technical University, Türkiye)*; Eha Nurk (National Institute for Health Development, Estonia)*; Fred Nuwaha (Makerere University School of Public Health, Uganda)*; Moffat Nyirenda (London School of Hygiene & Tropical Medicine, UK)*; Terence W O'Neill (University of Manchester, UK)*; Caleb Ochimana (Harvard TH Chan School of Public Health, USA)*; Angélica M Ochoa-Avilés (Universidad de Cuenca, Ecuador)*; Eiji Oda (Tachikawa General Hospital, Japan)*; Augustine N Odili (University of Abuja College of Health Sciences, Nigeria)*; Kyungwon Oh (Korea Disease Control and Prevention Agency, Republic of Korea)*; Ryutaro Ohtsuka (Japan Wildlife Research Center, Japan; deceased)*; Brian Oldenburg (Baker Heart and Diabetes Institute, Australia)*; Valérie Olié (The National Public Health Agency, France)*; Mohd Azahadi Omar (Ministry of Health, Malaysia)*; Saeed M Omar (Gadarif University, Sudan)*; Altan Onat (Istanbul University, Türkiye; deceased)*; Sok King Ong (Ministry of Health, Brunei)*; N Charlotte Onland-Moret (Utrecht University, The Netherlands)*; Lariane M Ono (Federal University of Paraná, Brazil)*; Obinna Onodugo (University of Nigeria, Nigeria)*; Pedro Ordunez (Pan American Health Organization, USA)*; Rui Ornelas (University of Madeira, Portugal)*; Ana P Ortiz (University of Puerto Rico, Puerto Rico)*; Pedro J Ortiz (Universidad Peruana Cayetano Heredia, Peru)*; Clive Osmond (University of Southampton, UK)*; Sergej M Ostojic (University of Novi Sad, Serbia)*; Afshin Ostovar (Osteoporosis Research Center, Iran)*; Johanna A Otero (Universidad de Santander, Colombia)*; Charlotte B Ottendahl (University of Southern Denmark, Denmark)*; Akaninyene Otu (University of Calabar, Nigeria)*; Kim Overvad (Aarhus University, Denmark)*; Ellis Owusu-Dabo (Kwame Nkrumah

University of Science and Technology, Ghana)*; Cristina P Padez (University of Coimbra, Portugal)*; Ioannis Pagkalos (International Hellenic University, Greece)*; Natalja Pajula (National Institute for Health Development, Estonia)*; Alberto Palloni (University of Wisconsin-Madison, USA)*; Luigi Palmieri (Istituto Superiore di Sanità, Italy)*; Wen-Harn Pan (Academia Sinica, Taiwan)*; Francesco Panza (University of Bari Aldo Moro, Italy)*; Mariela Paoli (University of the Andes, Venezuela)*; Sousana K Papadopoulou (International Hellenic University, Greece)*; Rossina G Pareja (Instituto de Investigación Nutricional, Peru)*; Soon-Woo Park (Catholic University of Daegu, Republic of Korea)*; Suyeon Park (Korea Disease Control and Prevention Agency, Republic of Korea)*; Winsome R Parnell (University of Otago, New Zealand)*; Mahboubeh Parsaeian (Tehran University of Medical Sciences, Iran)*; Ionela M Pascanu (University of Medicine, Pharmacy, Sciences and Technology of Târgu Mures, Romania)*; Patrick Pasquet (UMR CNRS-MNHN 7206, France)*; Nikhil D Patel (Jivandeep Hospital, India)*; Halyna Pavlyshyn (Ternopil National Medical University, Ukraine)*; Raimund Pechlaner (Medical University of Innsbruck, Austria)*; Ivan Pećin (University Hospital Centre Zagreb, Croatia)*; João M Pedro (Centro de Investigação em Saúde de Angola, Angola)*; Sergio Viana Peixoto (Fundação Oswaldo Cruz, Brazil)*; Markku Peltonen (Finnish Institute for Health and Welfare, Finland)*; Alexandre C Pereira (University of São Paulo, Brazil)*; Karen GDA Peres (Griffith University, Australia)*; Marco A Peres (National Dental Care Centre Singapore, Singapore)*; Agustín Perez-Londoño (Universidad de los Andes, Colombia)*; Cynthia M Pérez (University of Puerto Rico, Puerto Rico)*; Valentina Peterkova (National Medical Research Center for Endocrinology, Russia)*; Olga Petrovna Kovtun (Yekaterinburg State Medical Academy, Russia)*; Niloofar Peykari (Ministry of Health and Medical Education, Iran)*; Son Thai Pham (Vietnam National Heart Institute, Vietnam)*; Rafael N Pichardo (Clínica de Medicina Avanzada Dr. Abel González, Dominican Republic)*; Preux Pierre-Marie (Université de Limoges, France)*; Hynek Pikhart (University College London, UK)*; Aida Pilav (University of Sarajevo, Bosnia and Herzegovina)*; Pavel Piler (Masaryk University, Czech Republic)*; Aleksandra Piwonska (National Institute of Cardiology, Poland)*; Andreia N Pizarro (University of Porto, Portugal)*; Silvia Plata (Observatorio de Salud Pública de Santander,

Colombia)*; Raluca M Pop (University of Medicine, Pharmacy, Sciences and Technology of Târgu Mures, Romania)*; Barry M Popkin (University of North Carolina at Chapel Hill, USA)*; Stevo R Popovic (University of Montenegro, Montenegro)*; Miquel Porta (Institut Hospital del Mar d'Investigacions Mèdiques, Spain)*; Anil Poudyal (Nepal Health Research Council, Nepal)*; Farhad Pourfarzi (Ardabil University of Medical Sciences, Iran)*; Akram Pourshams (Tehran University of Medical Sciences, Iran)*; Hossein Poustchi (Tehran University of Medical Sciences, Iran)*; Rajendra Pradeepa (Madras Diabetes Research Foundation, India)*; Alison J Price (London School of Hygiene & Tropical Medicine, UK)*; Antonio Prista (Universidade Pedagógica, Mozambique)*; Rui Providencia (University College London, UK)*; Jardena J Puder (Lausanne University Hospital, Switzerland)*; Iveta Pudule (Centre for Disease Prevention and Control, Latvia)*; Soile Puhakka (Oulu Deaconess Institute Foundation, Finland)*; Maria Puiu (Victor Babes University of Medicine and Pharmacy Timisoara, Romania)*; Margus Punab (Tartu University Clinics, Estonia)*; Mostafa Qorbani (Alborz University of Medical Sciences, Iran)*; Anna Quialheiro (Cooperativa de Ensino Superior Politécnico e Universitário, Portugal)*; Hedley K Quintana (Gorgas Memorial Institute for Studies of Health, Panama)*; Pedro J Quiroga-Padilla (Universidad de los Andes, Colombia)*; Tran Quoc Bao (Ministry of Health, Vietnam)*; Stefan Rach (Leibniz Institute for Prevention Research and Epidemiology - BIPS, Germany)*; Salar Rahimikazerooni (Shiraz University of Medical Sciences, Iran)*; Mahmudur Rahman (Institute of Epidemiology Disease Control and Research, Bangladesh)*; Olli Raitakari (University of Turku, Finland)*; Sherali Rakhmatulloev (Ministry of Health and Social Protection, Tajikistan)*; Ivo Rakovac (World Health Organization Regional Office for Europe, Denmark)*; Ambady Ramachandran (India Diabetes Research Foundation, India)*; Otim PC Ramadan (World Health Organization Country Office, South Sudan)*; Manuel Ramirez-Zea (Institute of Nutrition of Central America and Panama, Guatemala)*; Rafel Ramos (Institut Universitari d'Investigació en Atenció Primària Jordi Gol, Spain)*; Lekhraj Rampal (Universiti Putra Malaysia, Malaysia)*; Sanjay Rampal (University of Malaya, Malaysia)*; Sheena E Ramsay (Newcastle University, UK)*; João FLB Rangel Junior (University of Pernambuco, Brazil)*; Daniel A Rangel Reina (Instituto Conmemorativo Gorgas

de Estudios de la Salud, Panama)*; Lalka S Rangelova (National Center of Public Health and Analyses, Bulgaria)*; Vayia Rarra (Sotiria Hospital, Greece)*; Mohammad-Mahdi Rashidi (Non-Communicable Diseases Research Center, Iran)*; Cassiano Ricardo Rech (Federal University of Santa Catarina, Brazil)*; Josep Redon (University of Valencia, Spain)*; Valéria Regecová (Slovak Academy of Sciences, Slovakia)*; Jane DP Renner (University of Santa Cruz do Sul, Brazil)*; Judit A Repasy (University of Pécs, Hungary)*; Cézane P Reuter (University of Santa Cruz do Sul, Brazil)*; Luis Revilla (Universidad San Martín de Porres, Peru)*; Andrew Reynolds (University of Otago, New Zealand)*; Negar Rezaei (Tehran University of Medical Sciences, Iran)*; Abbas Rezaianzadeh (Shiraz University of Medical Sciences, Iran)*; Elio Riboli (Imperial College London, UK)*; Fernando Rigo (CS S. Agustín Ibsalut, Spain)*; Attilio Rigotti (Pontificia Universidad Católica de Chile, Chile)*; Leanne M Riley (World Health Organization, Switzerland)*; Tobias F Rinke de Wit (Amsterdam Institute for Global Health and Development, The Netherlands)*; Ulf Risérus (Uppsala University, Sweden)*; Raphael M Ritti-Dias (Universidade Nove de Julho, Brazil)*; Reina G Roa (Ministerio de Salud, Panama)*; Romana Roccaldo (Council for Agricultural Research and Economics, Italy)*; Fernando Rodríguez-Artalejo (Universidad Autónoma de Madrid CIBERESP, Spain)*; María del Cristo Rodriguez-Perez (Canarian Health Service, Spain)*; Laura A Rodríguez-Villamizar (Universidad Industrial de Santander, Colombia)*; Andrea Y Rodríguez (Ministery of Health and Social Protection, Colombia)*; Ulla Roggenbuck (University of Duisburg-Essen, Germany)*; Peter Rohloff (Wuqu' Kawoq, Guatemala)*; Rosalba Rojas-Martinez (National Institute of Public Health, Mexico)*; Elisabetta L Romeo (Associazione Calabrese di Epatologia, Italy)*; Rafaela V Rosario (University of Minho, Portugal)*; Annika Rosengren (University of Gothenburg, Sweden; Sahlgrenska University Hospital, Sweden)*; Ian Rouse (Fiji National University, Fiji)*; Adolfo Rubinstein (Institute for Clinical Effectiveness and Health Policy, Argentina)*; Blanca Sandra Ruiz-Betancourt (Instituto Mexicano del Seguro Social, Mexico)*; Maria Ruiz-Castell (Luxembourg Institute of Health, Luxembourg)*; Emma Ruiz Moreno (National Center of Epidemiology ISCII CIBERESP, Spain)*; Iuliia A Rusakova (Ufa Eye Research Institute, Russia)*; Wojciech Rusek (Rehamed-Center, Poland)*; Petra

Rust (University of Vienna, Austria)*; Marcin Rutkowski (Medical University of Gdansk, Poland)*; Marge Saamel (National Institute for Health Development, Estonia)*; Hamideh Sabbaghi (Shahid Beheshti University of Medical Sciences, Iran)*; Harshpal S Sachdev (Sitaram Bhartia Institute of Science and Research, India)*; Alireza Sadjadi (Tehran University of Medical Sciences, Iran)*; Ali Reza Safarpour (Shiraz University of Medical Sciences, Iran)*; Sare Safi (Shahid Beheshti University of Medical Sciences, Iran)*; Mohammad Hossien Saghi (Sabzevar University of Medical Sciences, Iran)*; Olfa Saidi (University Tunis El Manar, Tunisia)*; Calogero Saieva (Institute for Cancer Research, Prevention and Clinical Network, Italy)*; Satoko Sakata (Kyushu University, Japan)*; Nader Saki (Ahvaz Jundishapur University of Medical Sciences, Iran)*; Sanja Šalaj (University of Zagreb, Croatia)*; Eduardo Salazar Martinez (National Institute of Public Health, Mexico)*; Akkumis Salkhanova (Academy of Preventive Medicine, Kazakhstan)*; Jukka T Salonen (University of Helsinki, Finland)*; Margarita Samoutian (Kindergarten of Avlonari, Greece)*; Jose Sánchez-Abanto (National Institute of Health, Peru)*; Inés Sánchez Rodríguez (Instituto Murciano de Investigación Biosanitaria Virgen de la Arrixaca, Spain)*; Diana A Santos (Universidade de Lisboa, Portugal)*; Ina S Santos (Federal University of Pelotas, Brazil)*; Maria Paula Santos (University of Porto, Portugal)*; Tamara R Santos (Federal University of Alagoas, Brazil)*; Jouko L Saramies (Wellbeing Services County of South Karelia, Finland)*; Luis B Sardinha (Universidade de Lisboa, Portugal)*; Giselle Sarganas (German Centre for Cardiovascular Research, Germany; Robert Koch Institute, Germany)*; Nizal Sarrafzadegan (Isfahan Cardiovascular Research Center, Iran)*; Kai-Uwe Saum (German Cancer Research Center, Germany)*; Stefan Savin (World Health Organization, Switzerland)*; Mariana Sbaraini (Federal University of Rio Grande do Sul, Brazil)*; Marcia Scazufca (University of São Paulo Clinics Hospital, Brazil)*; Beatriz D Schaan (Federal University of Rio Grande do Sul, Brazil)*; Anja Schienkiewitz (Robert Koch Institute, Germany)*; Karin Schindler (Medical University of Vienna, Austria)*; Sabine Schipf (University Medicine Greifswald, Germany)*; Amand Floriaan Schmidt (University College London, UK)*; Börge Schmidt (University of Duisburg-Essen, Germany)*; Carsten O Schmidt (University Medicine Greifswald, Germany)*; Ben Schöttker

(German Cancer Research Center, Germany)*; Sara Schramm (University of Duisburg-Essen, Germany)*; Stine Schramm (University of Southern Denmark, Denmark)*; Helmut Schröder (CIBERESP, Spain: Hospital Del Mar Medical Research Institute, Spain)*; Constance Schultsz (University of Amsterdam, The Netherlands)*; Aletta E Schutte (University of New South Wales, Australia; The George Institute for Global Health, Australia)*; Sylvain Sebert (University of Oulu, Finland)*; Moslem Sedaghattalab (Yasuj University of Medical Sciences, Iran)*; Aye Aye Sein (Ministry of Health and Sports, Myanmar)*; Abhijit Sen (Center for Oral Health Services and Research Mid-Norway, Norway)*; Sadaf G Sepanlou (Tehran University of Medical Sciences, Iran)*; Guillermo Sequera (Ministerio de Salud Pública y Bienestar Social, Paraguay)*; Ľudmila Ševčíková (Comenius University, Slovakia)*; Ronel Sewpaul (Human Sciences Research Council, South Africa)*; Teresa Shamah-Levy (National Institute of Public Health, Mexico)*; Seyed Morteza Shamshirgaran (Neyshabur University of Medical Sciences, Iran)*; Maryam Sharafkhah (Tehran University of Medical Sciences, Iran)*; Sanjib K Sharma (B P Koirala Institute of Health Sciences, Nepal)*; Almaz Sharman (Academy of Preventive Medicine, Kazakhstan)*; Jonathan E Shaw (Baker Heart and Diabetes Institute, Australia)*; Amaneh Shayanrad (Tehran University of Medical Sciences, Iran)*; Ali Akbar Shayesteh (Ahvaz Jundishapur University of Medical Sciences, Iran)*; Lela Shengelia (National Center for Disease Control and Public Health, Georgia)*; Kenji Shibuya (King's College London, UK)*; Hana Shimizu-Furusawa (Teikyo University, Japan)*; Rahman Shiri (Finnish Institute of Occupational Health, Finland)*; Marat Shoranov (Kazakh National Medical University, Kazakhstan)*; Namuna Shrestha (Public Health Promotion and Development Organization, Nepal)*; Khairil Si-Ramlee (Ministry of Health, Brunei)*; Abla M Sibai (American University of Beirut, Lebanon)*; Labros S Sidossis (Rutgers University, USA)*; Antonio M Silva (Federal University of Maranhão, Brazil)*; Caroline Ramos de Moura Silva (University of Pernambuco, Brazil)*; Diego Augusto Santos Silva (Federal University of Santa Catarina, Brazil)*; Kelly Samara Silva (Federal University of Santa Catarina, Brazil)*; Xueling Sim (National University of Singapore, Singapore; National University Health System, Singapore)*; Mary Simon (India Diabetes Research Foundation, India)*; Michael Sjöström (Karolinska Institutet, Sweden;

deceased)*; Natalia A Skoblina (Pirogov Russian National Research Medical University, Russia)*; Jolanta Slowikowska-Hilczer (Medical University of Lodz, Poland)*; Przemysław Slusarczyk (International Institute of Molecular and Cell Biology, Poland)*; Liam Smeeth (London School of Hygiene & Tropical Medicine, UK)*; Lee Smith (Anglia Ruskin University, UK)*; Fernanda Cunha Soares (University of Pernambuco, Brazil)*; Grzegorz Sobek (University of Rzeszów, Poland)*; Eugène Sobngwi (University of Yaoundé 1, Cameroon)*; Morten Sodemann (University of Southern Denmark, Denmark)*; Stefan Söderberg (Umeå University, Sweden)*; Agustinus Soemantri (Diponegoro University, Indonesia; deceased)*; Vincenzo Solfrizzi (University of Bari Aldo Moro, Italy)*; Mohammad Hossein Somi (Tabriz University of Medical Sciences, Iran)*; Elin P Sørgjerd (Norwegian University of Science and Technology, Norway)*; Maroje Sorić (University of Zagreb, Croatia; University of Ljubljana, Slovenia)*; Victoria E Soto-Rojas (Universidad Icesi, Colombia)*; Aïcha Soumaré (University of Bordeaux, France)*; Alfonso Sousa-Poza (University of Hohenheim, Germany)*; Igor Spiroski (Institute of Public Health, North Macedonia; Ss. Cyril and Methodius University, North Macedonia)*; Jan A Staessen (KU Leuven, Belgium)*; Andreas Stang (University of Duisburg-Essen, Germany)*; Jostein Steene-Johannessen (Norwegian School of Sport Sciences, Norway)*; Peter Stehle (Bonn University, Germany)*; Aryeh D Stein (Emory University, USA)*; George S Stergiou (National and Kapodistrian University of Athens, Greece)*; Jakub Stokwiszewski (National Institute of Public Health - National Institute of Hygiene, Poland)*; Ekaterina Stoyanova (Kalina Malina Kindergarten, Bulgaria)*; Gareth Stratton (Swansea University, UK)*; Karien Stronks (University of Amsterdam, The Netherlands)*; Lela Sturua (National Center for Disease Control and Public Health, Georgia)*; Milton F Suarez-Ortegón (Pontificia Universidad Javeriana Seccional Cali, Colombia)*; Phalakorn Suebsamran (Ubon Ratchathani University, Thailand)*; Gerhard Sulo (University of Bergen, Norway)*; Johan Sundström (Uppsala University, Sweden)*; Paibul Suriyawongpaisal (Mahidol University, Thailand)*; Boyd A Swinburn (University of Auckland, New Zealand)*; René Charles Sylva (National Statistical Office, Cabo Verde)*; Lucjan Szponar (National Institute of Public Health - National Institute of Hygiene, Poland)*; E Shyong Tai (National University of Singapore,

Singapore; National University Health System, Singapore)*; Konstantinos D Tambalis (National and Kapodistrian University of Athens, Greece)*; Abdonas Tamosiunas (Lithuanian University of Health Sciences, Lithuania)*; Baimakhan Tanabayev (South Kazakhstan Medical Academy, Kazakhstan)*; Maya Tanrygulyyeva (Scientific Research Institute of Maternal and Child Health, Turkmenistan)*; Mohammed Rasoul Tarawneh (Ministry of Health, Jordan)*; Jakob Tarp (Norwegian School of Sport Sciences, Norway)*; Carolina B Tarqui-Mamani (National Institute of Health, Peru)*; Radka Taxová Braunerová (Institute of Endocrinology, Czech Republic)*; Saskia Te Velde (University of Applied Sciences Utrecht, The Netherlands)*; William R Tebar (Universidade Estadual Paulista, Brazil)*; Grethe S Tell (University of Bergen, Norway)*; Tania Tello (Universidad Peruana Cayetano Heredia, Peru)*; KR Thankappan (Amrita Institute of Medical Sciences, India)*; Xenophon Theodoridis (Aristotle University of Thessaloniki, Greece)*; Sathish Thirunavukkarasu (Emory University, USA)*; Nihal Thomas (Christian Medical College Vellore, India)*; Amanda G Thrift (Monash University, Australia)*; Ľubica Tichá (Comenius University, Slovakia)*; Erik J Timmermans (University Medical Center Utrecht, The Netherlands)*; Dwi Hapsari Tjandrarini (National Research and Innovation Agency, Indonesia)*; Anne Tjonneland (Danish Cancer Institute, Denmark)*; Janne S Tolstrup (University of Southern Denmark, Denmark)*; Murat Topbas (Karadeniz Technical University, Türkiye)*; Laura Torres-Collado (Universidad Miguel Hernandez, Spain)*; Giota Touloumi (National and Kapodistrian University of Athens, Greece)*; Pierre Traissac (French National Research Institute for Sustainable Development, France)*; Areti Triantafyllou (Aristotle University of Thessaloniki, Greece)*; Atul Trivedi (Government Medical College, India)*; Lechaba Tshepo (Sefako Makgatho Health Sciences University, South Africa)*; Panagiotis Tsintavis (International Hellenic University, Greece)*; John Tuitele (Tafuna Family Health Center, American Samoa; LBJ Hospital, American Samoa)*; Azaliia M Tuliakova (Ufa Eye Research Institute, Russia)*; Marshall K Tulloch-Reid (The University of the West Indies, Jamaica)*; Fikru Tullu (Addis Ababa University, Ethiopia)*; Tomi-Pekka Tuomainen (University of Eastern Finland, Finland)*; Maria L Turley (Ministry of Health, New Zealand)*; Evangelia Tzala (Imperial College London, UK)*; Themistoklis Tzotzas

(Hellenic Medical Association for Obesity, Greece)*; Christophe Tzourio (University of Bordeaux, France)*; Peter Ueda (Karolinska Institutet, Sweden)*; Eunice Ugel (Universidad Centro-Occidental Lisandro Alvarado, Venezuela)*; Flora AM Ukoli (Meharry Medical College, USA)*; Zhamyila Usupova (Republican Center for Health Promotion, Kyrgyzstan)*; Hannu MT Uusitalo (University of Tampere Tays Eye Center, Finland)*; Nalan Uysal (Sabiha Gokcen Ilkokulu, Türkiye)*; Gonzalo Valdivia (Pontificia Universidad Católica de Chile, Chile)*; Damaskini Valvi (Icahn School of Medicine at Mount Sinai, USA)*; Rob M van Dam (George Washington University, USA)*; Bert-Jan van den Born (University of Amsterdam, The Netherlands)*; Johan Van der Heyden (Sciensano, Belgium)*; Yvonne T van der Schouw (Utrecht University, The Netherlands)*; Wendy Van Lippevelde (Ghent University, Belgium)*; Hoang Van Minh (Hanoi University of Public Health, Vietnam)*; Natasja M Van Schoor (Vrije Universiteit Amsterdam, The Netherlands)*; Irene GM van Valkengoed (University of Amsterdam, The Netherlands)*; Dirk Vanderschueren (KU Leuven, Belgium)*; Diego Vanuzzo (MONICA-FRIULI Study Group, Italy)*; Gregorio Varela-Moreiras (Universidad CEU San Pablo, Spain)*; Luz Nayibe Vargas (Pontificia Universidad Javeriana, Colombia)*; Senthil K Vasan (University of Southampton, UK)*; Daniel G Vasques (Federal University of Rio Grande do Sul, Brazil)*; Tomas Vega (Consejería de Sanidad Junta de Castilla y León, Spain)*; Gustavo Velasquez-Melendez (Federal University of Minas Gerais, Brazil)*; Biruta Velika (Centre for Disease Prevention and Control, Latvia)*; Charlotte Verdot (The National Public Health Agency, France)*; Maïté Verloigne (Ghent University, Belgium)*; Giovanni Veronesi (University of Insubria, Italy)*; WM Monique Verschuren (National Institute for Public Health and the Environment, The Netherlands)*; Roosmarijn Verstraeten (Institute of Tropical Medicine, Belgium)*; Lucie Viet (National Institute for Public Health and the Environment, The Netherlands)*; Frøydis N Vik (University of Agder, Norway)*; Monica Vilar (Universidad San Francisco de Quito, Ecuador)*; Salvador Villalpando (National Institute of Public Health, Mexico)*; Jesus Vioque (CIBERESP, Spain)*; Jyrki K Virtanen (University of Eastern Finland, Finland)*; Marjolein Visser (Vrije Universiteit Amsterdam, The Netherlands)*; Bharathi Viswanathan (Ministry of Health, Seychelles)*; Mihaela Vladulescu (Sunflower Nursery School,

Romania)*; Henry Völzke (University Medicine Greifswald, Germany)*; Ari Voutilainen (University of Eastern Finland, Finland)*; Martine Vrijheid (CIBERESP, Spain)*; Alisha N Wade (University of the Witwatersrand, South Africa)*; Wan Mohamad Wan Bebakar (Universiti Sains Malaysia, Malaysia)*; Wan Nazaimoon Wan Mohamud (Institute for Medical Research, Malaysia)*; Rildo de Souza Wanderley Júnior (University of Pernambuco, Brazil)*; Chongjian Wang (Zhengzhou University, China)*; Huijun Wang (Chinese Center for Disease Control and Prevention, China)*; Ningli Wang (Capital Medical University Beijing Tongren Hospital, China)*; Qian Wang (Xinjiang Medical University, China)*; Xiangjun Wang (Shanghai Educational Development Co. Ltd, China)*; Ya Xing Wang (Capital Medical University, China)*; Ying-Wei Wang (Ministry of Health and Welfare, Taiwan)*; S Goya Wannamethee (University College London, UK)*; Nicholas Wareham (University of Cambridge, UK)*; Olivia Wartha (Ulm University Hospital, Germany)*; Adelheid Weber (Federal Ministry of Social Affairs, Health, Care and Consumer Protection, Austria)*; Karen Webster-Kerr (The Ministry of Health and Wellness, Jamaica)*; Niels Wedderkopp (University of Southern Denmark, Denmark)*; Daniel Weghuber (Paracelsus Medical University, Austria)*; Wenbin Wei (Capital Medical University, China)*; Leo Westbury (University of Southampton, UK)*; Peter H Whincup (St George's, University of London, UK)*; Kremlin Wickramasinghe (World Health Organization Regional Office for Europe, Denmark)*; Kurt Widhalm (Medical University of Vienna, Austria)*; Indah S Widyahening (Universitas Indonesia, Indonesia)*; Andrzej Więcek (Medical University of Silesia, Poland)*; Rainford J Wilks (The University of the West Indies, Jamaica)*; Karin Willeit (Medical University of Innsbruck, Austria)*; Peter Willeit (Medical University of Innsbruck, Austria)*; Julianne Williams (World Health Organization Regional Office for Europe, Denmark)*; Tom Wilsgaard (UiT The Arctic University of Norway, Norway)*; Bogdan Wojtyniak (National Institute of Public Health NIH - National Research Institute, Poland)*; Roy A Wong-McClure (Caja Costarricense de Seguro Social, Costa Rica)*; Andrew Wong (University College London, UK)*; Emily B Wong (Africa Health Research Institute, South Africa)*; Mark Woodward (University of New South Wales, Australia; Imperial College London, UK)*; Frederick C Wu (University of Manchester, UK)*; Justyna Wyszyńska (University of Rzeszów,

Poland)*; Haiquan Xu (Institute of Food and Nutrition Development of Ministry of Agriculture and Rural Affairs, China)*; Liang Xu (Beijing Institute of Ophthalmology, China)*; Nor Azwany Yaacob (Universiti Sains Malaysia, Malaysia)*; Li Yan (Imperial College London, UK)*; Weili Yan (Children's Hospital of Fudan University, China)*; Yang Yang (Shanghai Educational Development Co. Ltd, China)*; Martha Yépez García (Universidad San Francisco de Quito, Ecuador)*; Moein Yoosefi (Non-Communicable Diseases Research Center, Iran)*; Akihiro Yoshihara (Niigata University, Japan)*; Novie O Younger-Coleman (The University of the West Indies, Jamaica)*; Yu-Ling Yu (KU Leuven, Belgium)*; Yunjiang Yu (South China Institute of Environmental Sciences, China)*; Ahmad Faudzi Yusoff (Ministry of Health, Malaysia)*; Vassilis Zafiropulos (Hellenic Mediterranean University, Greece)*; Ahmad A Zainuddin (Ministry of Health, Malaysia)*; Farhad Zamani (Iran University of Medical Sciences, Iran)*; Sabina Zambon (University of Padua, Italy)*; Antonis Zampelas (Agricultural University of Athens, Greece)*; Maria Elisa Zapata (Centro de Estudios Sobre Nutrición Infantil, Argentina)*; Ko Ko Zaw (University of Public Health, Myanmar)*; Tomasz Zdrojewski (Medical University of Gdańsk, Poland)*; Magdalena Żegleń (Jagiellonian University, Poland)*; Kristyna Zejglicova (National Institute of Public Health, Czech Republic)*; Tajana Zeljkovic Vrkic (University Hospital Centre Zagreb, Croatia)*; Bing Zhang (Chinese Center for Disease Control and Prevention, China)*; Zhen-Yu Zhang (KU Leuven, Belgium)*; Yanitsa V Zhecheva (Bulgarian Academy of Sciences, Bulgaria)*; Bekbolat Zholdin (West Kazakhstan Medical University, Kazakhstan)*; Paul Zimmet (Monash University, Australia)*; Marie Zins (Institut National de la Santé et de la Recherche Médicale, France; Paris Cité University, France)*; Julio Zuñiga Cisneros (Instituto Conmemorativo Gorgas de Estudios de la Salud, Panama)*; Monika Zuziak (Przedszkole No. 81, Poland)*

Appendix Text 2: Data sources

We used individual participant data from representative samples of the general population, collated by the NCD Risk Factor Collaboration (NCD-RisC), as detailed previously.[1,2] In summary, data were obtained from publicly available multi-country and national measurement surveys (e.g., Demographic and Health Surveys, WHO STEPwise approach to Surveillance (STEPS) surveys, and those identified via the Inter-University Consortium for Political and Social Research, European Health Interview and Health Examination Surveys Database, and the UK Data Service). With the help of the World Health Organization (WHO) regional and country offices, we identified and accessed population-based survey data from national health and statistical agencies. We searched and reviewed published studies as detailed previously,[3] and invited eligible studies to join NCD-RisC, as we did with data holders from a previous pooled analyses of cardiometabolic risk factors.[4-7] The NCD-RisC database is continuously updated through all the above routes as well as through periodic requests to NCD-RisC members, which consist of representatives of participating studies who are knowledgeable with the study's methods and measurements and often with other studies in their countries and regions, to suggest additional sources in their countries.

We carefully checked that each data source met our inclusion criteria, described below. Potential duplicate data sources were first identified by comparing studies from the same country and year, followed by checking with NCD-RisC members who had provided data whether sources from the same country and year, and with similar sample sizes and age ranges, were the same or distinct. If two sources were confirmed as duplicates, only one was maintained and used. All NCD-RisC members were also periodically asked to review the list of sources from their countries, to verify that the included data met the inclusion criteria, were not duplicates, and were appropriately classified in terms of the population that they had sampled from. For each data source, we recorded the study population, the sampling approach, the years of measurement and the measurement methods. This information was provided by participating studies together with available documentation on study design and methods. All

submitted data were checked by at least two persons independently. Questions and clarifications were discussed with NCD-RisC members and resolved before data were incorporated into the database.

The inclusion criteria were: (1) data were collected using a probabilistic sampling method with a defined sampling frame; (2) data were from general population samples from one of the 200 countries and territories listed in Appendix Table 1; and (3) height, weight and waist circumference were measured (as opposed to self-reported) with a validated protocol. When participants from a population-based study had been measured at multiple points in time, we used only the first instance of (re)measurement in 1990 or later. Studies were excluded if they had (1) included or excluded participants based on health status; (2) were conducted only among ethnic minorities or specific educational, occupational, or other socioeconomic subgroups; or (3) recruited participants through health facilities, with the exception of studies whose sampling frame was health insurance schemes in countries where at least 80% of the general population were insured, and studies in high-income and central European countries with universal insurance which had sampled via the primary care system.

Appendix Text 3: Data cleaning

We excluded women who were pregnant at the time of measurement because body weight and waist circumference change during pregnancy. We also excluded participants with missing sex (13,572 participants, 0.2% of all data). We excluded 8,477 participants (0.1% of all data) with weight <12 kg or >300 kg; height <100 cm or >250 cm; waist circumference <30 cm or >200 cm; BMI <10 kg/m^2 or >80 kg/m^2; WHtR <0.2 or >2.0 because such values are likely to be due to recording error. For analyses with data on blood pressure, we excluded participants whose blood pressure was not measured by design (because some studies only measured blood pressure in a random subset of participants), those who were missing blood pressure or hypertension treatment information, and those with SBP <70 mmHg or >270 mmHg, DBP <30 mmHg or >150 mmHg or with SBP ≤ DBP (a total of 171,082 participants, 3.1% of all data).

We also applied an outlier detection procedure using Mahalanobis distance to exclude risk factor pairs that had an implausible pairwise relationship relative to the overall data.[8] This method uses the empirical relationship between risk factor pairs to detect extreme combinations, for example a high SBP of 248 mmHg but low DBP of 40 mmHg, or a high BMI of 42 kg/m^2 but low waist circumference of 74 cm. We applied this technique separately to all pairs of anthropometric variables (height, weight, BMI, waist circumference and WHtR) and to the pair SBP and DBP. All variables, except height and DBP, were log transformed before outlier detection to account for their skewed distributions. We removed those pairs of measurements with a distance of more than six standard deviations (SDs) away from the joint distribution's mean (i.e. a distance greater than 40.08, the quantile of the chi-square distribution which is equivalent to six SDs of the normal distribution). Pairwise cleaning led to excluding 31,508 participants (0.4% of all data) from all analyses. A flowchart of data cleaning and use is given in Appendix Figure 2.

Appendix Text 4: Comparison with previous studies

All but one of the previous studies comparing BMI and abdominal adiposity, and their association with cardiometabolic conditions, were done in one or a small number of regions or ethnic groups.[9-36] Collectively, their results indicate that the relationship between BMI and measures of abdominal obesity varies across global population, and that those from Asia may have higher levels of abdominal obesity than predicted based on BMI compared to European and African populations. However, differences in design and reporting, including their classification of global populations (e.g., whether and how different parts of Asia were differentiated), do not allow consistent quantitative comparisons. Some of these studies found that BMI and indices of abdominal adiposity performed similarly in predicting incident or prevalent cardiometabolic conditions while others found different performance.[10,12,13,15,17-22,24-32,34,35,37-39] The only study with participants from different regions[38] did not quantitatively compare BMI and abdominal obesity. This study found a slightly larger increase in the risk of prevalent cardiovascular disease and diabetes per SD of waist circumference than BMI,[38] whereas we found similar associations for BMI and WHtR with hypertension as seen in Appendix Figure 11.

Appendix Table 1. List of analysis regions and countries in each region.

Region	Countries
Central and eastern Europe	Albania, Belarus, Bosnia and Herzegovina, Bulgaria, Croatia, Czechia, Estonia, Hungary, Lithuania, Moldova, Poland, Romania, Russian Federation, Slovakia, Ukraine
High-income western	Australia, Belgium, Denmark, Finland, France, Germany, Greece, Ireland, Israel, Italy, Luxembourg, Malta, Netherlands, New Zealand, Norway, Portugal, Spain, Sweden, United Kingdom, United States of America
Latin America and the Caribbean	Argentina, Bahamas, Barbados, Belize, Bolivia, Brazil, Chile, Colombia, Costa Rica, Cuba, Dominica, Dominican Republic, Ecuador, El Salvador, Grenada, Guatemala, Guyana, Haiti, Honduras, Jamaica, Mexico, Nicaragua, Panama, Paraguay, Peru, Saint Kitts and Nevis, Saint Lucia, Saint Vincent and the Grenadines, Suriname, Trinidad and Tobago, Uruguay, Venezuela
Central Asia, Middle East and north Africa	Algeria, Armenia, Azerbaijan, Egypt, Georgia, Iran, Iraq, Jordan, Kazakhstan, Kuwait, Kyrgyzstan, Lebanon, Libya, Mongolia, Morocco, Oman, Qatar, Saudi Arabia, State of Palestine, Tajikistan, Tunisia, Turkiye, Turkmenistan, United Arab Emirates, Uzbekistan, Yemen
South Asia	Afghanistan, Bangladesh, Bhutan, India, Nepal, Pakistan, Sri Lanka
Sub-Saharan Africa	Angola, Benin, Botswana, Burkina Faso, Cabo Verde, Cameroon, Central African Republic, Chad, Comoros, Congo, Cote d'Ivoire, DR Congo, Eritrea, Eswatini, Ethiopia, Gabon, Gambia, Ghana, Guinea, Kenya, Lesotho, Liberia, Madagascar, Malawi, Mali, Mauritania, Mauritius, Mozambique, Namibia, Niger, Nigeria, Rwanda, Sao Tome and Principe, Senegal, Seychelles, Sierra Leone, Somalia, South Africa, South Sudan, Sudan, Tanzania, Togo, Uganda, Zambia
East and southeast Asia and the Pacific	Brunei Darussalam, Cambodia, China, Indonesia, Japan, Lao PDR, Malaysia, Maldives, Myanmar, Philippines, Singapore, South Korea, Taiwan, Thailand, Timor-Leste, Viet Nam
Oceania	American Samoa, Cook Islands, Federated States of Micronesia, Fiji, French Polynesia, Kiribati, Marshall Islands, Nauru, Niue, Palau, Papua New Guinea, Samoa, Solomon Islands, Tokelau, Tonga, Tuvalu, Vanuatu

Appendix Table 2. Data sources used in the analysis.

	Country	Years	Survey/Study name/Citation	Age range used in anthropometric analyses		Age range used in hypertension analyses		Individuals used in anthropometric analyses		Individuals used in hypertension analyses		Region	Notes
				Women	Men	Women	Men	Women	Men	Women	Men		
1	Afghanistan	2018	STEPS	20-64	20-64	20-64	20-64	1,458	1,592	1,458	1,592	South Asia	
2	Albania	2017-2018	DHS	20-59	20-59	20-59	20-59	12,676	5,078	12,478	4,926	Central and eastern Europe	
3	Algeria	2003	STEPS	25-64	25-64	25-64	25-64	2,415	1,580	2,415	1,579	Central Asia, Middle East and north Africa	
4	Algeria	2005	Transition and Health Impact in North Africa	35-64	35-64			2,444	1,756			Central Asia, Middle East and north Africa	
5	Algeria	2007-2009	The ISOR (InSulino-resistance in ORan) Study	30-64	30-64	30-64	30-64	406	375	403	375	Central Asia, Middle East and north Africa	
6	Algeria	2016-2017	STEPS	20-64	20-64	20-64	20-64	3,285	2,705	3,275	2,697	Central Asia, Middle East and north Africa	
7	American Samoa	1994	McGarvey, Pac Health Dialog 8(1):157-62, 2001	29-64	29-64	29-64	29-64	230	155	212	139	Oceania	
8	American Samoa	2004	STEPS	25-64	25-64	25-64	25-64	1,049	912	1,047	908	Oceania	
9	Angola	2013-2014	CardioBengo - Population based cardiovascular longitudinal study in Bengo Province, Angola	20-64	20-64	20-64	20-64	1,272	686	1,223	665	Sub-Saharan Africa	
10	Argentina	2005	Encuesta Nacional de Nutrición y Salud 2005	20-49				4,391				Latin America and the Caribbean	
11	Argentina	2018	Encuesta Nacional de Factores de Riesgo 2018	20-64	20-64	20-64	20-64	7,225	5,398	7,059	5,226	Latin America and the Caribbean	
12	Argentina	2011-2012	CESCAS Study	35-64	35-64	35-64	35-64	1,916	1,262	1,716	1,167	Latin America and the Caribbean	
13	Argentina	2012-2013	Primer estudio sobre el estado nutricional y los hábitos alimentarios de la población adulta de Rosario	20-64	20-64	20-64	20-64	715	328	666	291	Latin America and the Caribbean	
14	Argentina	2014-2015	Latin American Study of Nutrition and Health (ELANS)	20-64	20-64			621	479			Latin America and the Caribbean	
15	Armenia	2016	STEPS	20-64	20-64	20-64	20-64	1,274	505	1,268	491	Central Asia, Middle East and north Africa	
16	Australia	1999-2000	The Australian Diabetes, Obesity and Lifestyle Study 1999-2000	25-64	25-64	25-64	25-64	4,792	3,904	4,765	3,887	High-income western	
17	Austria	2010-2012	Austrian Study on Nutritional Status 2012	20-64	20-64			176	116			High-income western	
18	Austria	2013-2017	Austrian Study on Nutrition (ASN)	20-64	20-64			1,304	757			High-income western	
19	Azerbaijan	2017	STEPS	20-64	20-64	20-64	20-64	1,416	1,006	1,412	1,006	Central Asia, Middle East and north Africa	
20	Bahamas	2019	STEPS	20-64	20-64	20-64	20-64	1,125	707	1,121	706	Latin America and the Caribbean	
21	Bahamas	2011-2012	STEPS	25-64	25-64	25-64	25-64	603	368	580	352	Latin America and the Caribbean	
22	Bangladesh	2002	STEPS	25-64	25-64			5,758	5,606			South Asia	
23	Bangladesh	2013	STEPS	25-64	25-64	25-64	25-64	2,027	1,551	2,026	1,550	South Asia	
24	Bangladesh	2016	Diabetes Mellitus: Action through community Groups or Health Information for better Control of population blood glucose, risk factors, knowledge and care seeking (DMagic)	30-64	30-64	30-64	30-64	5,576	4,689	5,576	4,689	South Asia	
25	Bangladesh	2018	STEPS	20-64	20-64	20-64	20-64	3,959	3,497	3,958	3,494	South Asia	
26	Bangladesh	2009-2010	STEPS	25-64	25-64	25-64	25-64	4,612	3,780	4,612	3,778	South Asia	
27	Bangladesh	2011-2012	Chronic Disease Risk Factor Study	20-64	20-64	20-64	20-64	378	239	369	234	South Asia	
28	Bangladesh	2018-2019	National Nutrition Surveillence	20-64	20-64			6,012	5,991			South Asia	
29	Barbados	1999-2000	The Survey on Health, Well-Being, and Aging in Latin America and the Caribbean (SABE)	60-64	60-64			173	94			Latin America and the Caribbean	1
30	Barbados	2011-2013	Health of the Nation (HotN)	25-64	25-64	25-64	25-64	555	354	553	353	Latin America and the Caribbean	
31	Belarus	2020	STEPS	20-64	20-64	20-64	20-64	2,606	2,044	2,606	2,044	Central and eastern Europe	
32	Belarus	2016-2017	STEPS	20-64	20-64	20-64	20-64	2,543	1,892	2,542	1,892	Central and eastern Europe	
33	Belgium	2003	The European Male Ageing Study		40-64		40-64		268		267	High-income western	
34	Belgium	1991-1994	Flemish Study on Environment, Genes and Health Outcomes	26-64	26-64	26-64	26-64	327	314	325	313	High-income western	
35	Belgium	2014-2015	Food Consumption Survey	20-64	20-64			589	560			High-income western	
36	Belgium	2018-2019	Belgian Health Examination Survey	20-64	20-64	20-64	20-64	483	417	479	413	High-income western	2
37	Belize	2005-2006	CAMDI	20-64	20-64	20-64	20-64	1,152	747	1,149	746	Latin America and the Caribbean	
38	Benin	2007	STEPS	25-64	25-64	25-64	25-64	1,504	951	1,464	950	Sub-Saharan Africa	
39	Benin	2008	STEPS	25-64	25-64	25-64	25-64	3,353	3,413	3,341	3,410	Sub-Saharan Africa	
40	Benin	2015	STEPS	20-64	20-64	20-64	20-64	2,336	2,118	2,333	2,116	Sub-Saharan Africa	
41	Bhutan	2007	STEPS	25-64	25-64	25-64	25-64	1,099	963	1,095	957	South Asia	
42	Bhutan	2014	STEPS	20-64	20-64	20-64	20-64	1,597	995	1,597	995	South Asia	
43	Bhutan	2019	STEPS	20-64	20-64	20-64	20-64	3,063	1,932	3,063	1,932	South Asia	
44	Bolivia	2019	STEPS	20-64	20-64	20-64	20-64	2,096	1,508	2,094	1,508	Latin America and the Caribbean	
45	Bosnia and Herzegovina	2002	Non-communicable disease risk factor survey, Federation of B&H	25-64	25-64	25-64	25-64	1,606	1,100	1,581	1,080	Central and eastern Europe	
46	Botswana	2007	STEPS	25-64	25-64	25-64	25-64	2,496	1,184	2,453	1,174	Sub-Saharan Africa	
47	Botswana	2014	STEPS	20-64	20-64	20-64	20-64	2,220	1,096	2,216	1,096	Sub-Saharan Africa	
48	Brazil	2004	Caju & Virgen das Gracas	20-64	20-64	20-64	20-64	224	229	224	229	Latin America and the Caribbean	
49	Brazil	2005	Syndrome of Obesity and Risk Factors for Cardiovascular Disease Study	20-64	20-64	20-64	20-64	721	522	714	521	Latin America and the Caribbean	
50	Brazil	2008	Caju & Virgen das Gracas	20-64	20-64	20-64	20-64	214	213	214	212	Latin America and the Caribbean	
51	Brazil	2010	San Pedro	20-64	20-64	20-64	20-64	164	115	159	107	Latin America and the Caribbean	
52	Brazil	2013	Pesquisas Nacional de Saude	20-64	20-64	20-64	20-64	26,756	20,876	26,269	19,915	Latin America and the Caribbean	
53	Brazil	2017	Effectiveness of interventions for health promotion in frail older adults with chronic non-communicable diseases in primary healthcare in Recife: a randomized community trial	60-64	60-64	60-64	60-64	139	34	139	34	Latin America and the Caribbean	
54	Brazil	2017	HealthRise Evaluation	30-64	30-64			891	431			Latin America and the Caribbean	
55	Brazil	1996-1997	The Bambui Cohort Study of Ageing	20-64	20-64	20-64	20-64	711	538	285	205	Latin America and the Caribbean	
56	Brazil	1999-2000	The Survey on Health, Well-Being, and Aging in Latin America and the Caribbean (SABE)	60-64	60-64			221	155			Latin America and the Caribbean	1
57	Brazil	2002-2004	1978-1979 Ribeira Preto Birth Cohort	22-25	22-25	22-25	22-25	1,082	1,012	1,082	1,012	Latin America and the Caribbean	
58	Brazil	2004-2006	Hearts of Brazil	20-64	20-64	20-64	20-64	548	497	543	496	Latin America and the Caribbean	
59	Brazil	2009-2010	EpiFloripa Cohort Study of Ageing - Wave 1	60-64	60-64	60-64	60-64	287	175	285	173	Latin America and the Caribbean	
60	Brazil	2009-2010	EpiFloripa Adults Cohort Study	20-59	20-59	20-59	20-59	934	751	921	749	Latin America and the Caribbean	
61	Brazil	2010-2015	Baependi Heart Study	20-64	20-64	20-64	20-64	1,126	789	1,081	769	Latin America and the Caribbean	
62	Brazil	2011-2014	Profile of Risk Factors for Coronary Arterial Disease in Rio Grade do Sul - Revaluation After 10 Years	20-64	20-64	20-64	20-64	471	369	452	331	Latin America and the Caribbean	
63	Brazil	2012-2013	Prevalence of Leptin Polymorphism Gln223Arg	20-64	20-64	20-64	20-64	425	222	425	222	Latin America and the Caribbean	
64	Brazil	2014-2015	II Diagnóstico de Saúde da População Materno-Infantil do Estado de Alagoas	20-49		20-49		3,119		3,077		Latin America and the Caribbean	
65	Brazil	2014-2015	Latin American Study of Nutrition and Health (ELANS)	20-64	20-64			944	790			Latin America and the Caribbean	
66	Brazil	2015-2016	The Ouro Preto Study	20-64	20-64	20-64	20-64	251	136	250	136	Latin America and the Caribbean	
67	Brazil	2015-2016	Brazilian Longitudinal Study of the Elderly Health and Wellness	50-64	50-64	50-64	50-64	2,865	2,516	2,850	2,502	Latin America and the Caribbean	
68	Brazil	2015-2016	The 1993 Pelotas (Brazil) Birth Cohort: 22 years follow-up	21-23	21-23	21-23	21-23	1,870	1,687	1,863	1,685	Latin America and the Caribbean	

	Country	Years	Survey/Study name/Citation	Age range used in anthropometric analyses		Age range used in hypertension analyses		Individuals used in anthropometric analyses		Individuals used in hypertension analyses		Region	Notes
				Women	Men	Women	Men	Women	Men	Women	Men		
69	Brazil	2016-2017	Study in Presidente Prudente	20-64	20-64	20-64	20-64	293	176	293	176	Latin America and the Caribbean	
70	Brazil	2018-2019	Epidemiology in the health (Santo Anastácio Edition)	20-64	20-64	20-64	20-64	116	89	116	89	Latin America and the Caribbean	
71	Brunei Darussalam	2010-2011	National Health And Nutritional Status Survey (NHANSS)	20-64	20-64	20-64	20-64	773	651	771	649	East and southeast Asia and the Pacific	
72	Brunei Darussalam	2015-2016	National Non-Communicable Diseases Survey (NNCDS)	20-64	20-64	20-64	20-64	972	694	972	694	East and southeast Asia and the Pacific	
73	Bulgaria	2014	Bulgarian National Monitoring of Dietary Intake	20-64	20-64	20-64	20-64	965	940	927	914	Central and eastern Europe	
74	Bulgaria	2020	National Survey on Risk Factors for Population's Health in Bulgaria 2020	20-64	20-64	20-64	20-64	748	502	733	496	Central and eastern Europe	
75	Burkina Faso	2013	STEPS	25-64	25-64	25-64	25-64	2,233	2,205	1,999	1,815	Sub-Saharan Africa	
76	Burkina Faso	2021	STEPS	20-64	20-64	20-64	20-64	1,817	1,346	1,816	1,346	Sub-Saharan Africa	
77	Cabo Verde	2007	STEPS	25-64	25-64	25-64	25-64	1,058	653	1,058	653	Sub-Saharan Africa	
78	Cabo Verde	2020	STEPS	20-64	20-64	20-64	20-64	2,381	1,678	2,380	1,677	Sub-Saharan Africa	
79	Cambodia	2010	STEPS	25-64	25-64	25-64	25-64	3,342	1,878	3,342	1,878	East and southeast Asia and the Pacific	
80	Cambodia	2023	STEPS	20-64	20-64	20-64	20-64	2,335	1,345	2,335	1,345	East and southeast Asia and the Pacific	
81	Cameroon	2003	STEPS	20-64	20-64	20-64	20-64	4,201	2,810	3,958	2,633	Sub-Saharan Africa	
82	Cameroon	2007	Cameroon Burden of Diabetes - Second Survey	20-64	20-64	20-64	20-64	4,271	3,055	4,087	2,933	Sub-Saharan Africa	
83	Cameroon	2014	Prevalence and determinants of chronic kidney disease in rural and urban Cameroonians: A cross-sectional study	20-64	20-64	20-64	20-64	197	149	197	149	Sub-Saharan Africa	
84	Cameroon	2018	Prevalence and determinants of chronic kidney disease in urban adults' populations of northern Cameroon	20-64	20-64	20-64	20-64	198	175	193	173	Sub-Saharan Africa	
85	Cameroon	1998-1999	Essential Non-communicable disease Health Intervention Project (ENHIP)	20-64	20-64	20-64	20-64	1,034	774	1,031	767	Sub-Saharan Africa	
86	Cameroon	2009-2012	Anthropologie nutritionnelle des migrants d'Afrique centrale à la ville et en France	20-64	20-64			533	473			Sub-Saharan Africa	
87	Cameroon	2014-2015	Cardiovascular risk factors screening in urban and rural areas in the Far-North Region Cameroon	20-64	20-64	20-64	20-64	331	482	331	482	Sub-Saharan Africa	
88	Central African Republic	2010	STEPS	25-64	25-64	25-64	25-64	1,960	1,833	1,958	1,832	Sub-Saharan Africa	
89	Chad	2008	STEPS	25-64	25-64	25-64	25-64	588	733	574	721	Sub-Saharan Africa	
90	Chile	2003	Encuesta Nacional de Salud	20-64	20-64	20-64	20-64	1,287	1,122	1,277	1,118	Latin America and the Caribbean	
91	Chile	1992-1993	Miquel et al., Gastroenterology 115(4):937-46, 1998	20-64	20-64			947	612			Latin America and the Caribbean	
92	Chile	1999-2000	The Survey on Health, Well-Being, and Aging in Latin America and the Caribbean (SABE)	60-64	60-64			178	95			Latin America and the Caribbean	1
93	Chile	2009-2010	Encuesta Nacional de Salud	20-64	20-64	20-64	20-64	2,121	1,410	2,120	1,402	Latin America and the Caribbean	
94	Chile	2010-2011	Encuesta Nacional de Consumo Alimentario	20-64	20-64			1,685	924			Latin America and the Caribbean	
95	Chile	2011-2012	CESCAS Study	35-64	35-64	35-64	35-64	774	702	698	612	Latin America and the Caribbean	
96	Chile	2014-2015	Latin American Study of Nutrition and Health (ELANS)	20-64	20-64			390	355			Latin America and the Caribbean	
97	Chile	2016-2017	Encuesta Nacional de Salud	20-64	20-64	20-64	20-64	2,411	1,354	2,410	1,354	Latin America and the Caribbean	
98	China	1993	China Health and Nutrition Study	20-64	20-64	20-64	20-64	3,477	3,171	3,379	3,004	East and southeast Asia and the Pacific	3
99	China	2008	China Health and Retirement Longitudinal Study (CHARLS), pilot survey	45-64	45-64			691	643			East and southeast Asia and the Pacific	
100	China	2009	The 33 Chinese Communities Health Study (33CCHS)	20-64	20-64	20-64	20-64	6,710	7,260	5,872	5,612	East and southeast Asia and the Pacific	
101	China	2011	Beijing Eye Study	50-64	50-64	50-64	50-64	1,054	721	1,053	721	East and southeast Asia and the Pacific	
102	China	1992-1993	Anzhen 02 Cohort Study	34-64	34-64			2,115	2,029			East and southeast Asia and the Pacific	
103	China	1995-1996	Hong Kong Cardiovascular Risk Factor Prevalence Study 1995-1996	25-64	25-64	25-64	25-64	1,315	1,229	1,314	1,228	East and southeast Asia and the Pacific	
104	China	2004-2008	China Kadoorie Biobank baseline survey	35-64	35-64	35-64	35-64	257,860	170,944	257,851	170,939	East and southeast Asia and the Pacific	
105	China	2006-2007	Handan Eye Study	30-64	30-64			2,953	2,548			East and southeast Asia and the Pacific	
106	China	2007-2010	WHO Study on global AGEing and adult health (SAGE)	50-64	50-64	50-64	50-64	3,980	3,403	3,935	3,359	East and southeast Asia and the Pacific	
107	China	2011-2014	The FAMILY Cohort	20-64	20-64	20-64	20-64	5,143	3,980	4,712	3,656	East and southeast Asia and the Pacific	
108	China	2015-2016	INTERMAP China Prospective (ICP)	40-64	40-64	40-64	40-64	236	185	236	185	East and southeast Asia and the Pacific	
109	China	2015-2017	Henan Rural Cohort	20-64	20-64	20-64	20-64	17,966	10,743	17,954	10,733	East and southeast Asia and the Pacific	
110	Colombia	2002	The Santa Fe Study (Santa Fe)	20-64	20-64	20-64	20-64	570	323	518	295	Latin America and the Caribbean	
111	Colombia	2002	The Santa Fe Study (Tunjuelito)	20-29	20-29	20-29	20-29	181	115	175	110	Latin America and the Caribbean	
112	Colombia	2010	STEPS	20-64	20-64	20-64	20-64	1,161	867	1,113	828	Latin America and the Caribbean	
113	Colombia	2015	Encuesta Nacional de Situacion Nutricional	20-64	20-64			39,011	31,316			Latin America and the Caribbean	
114	Colombia	2015	STEPS	20-64	20-64	20-64	20-64	1,104	804	1,103	803	Latin America and the Caribbean	
115	Colombia	2016	The Survey on Health, Well-Being, and Aging in Latin America and the Caribbean (SABE)	60-64	60-64	60-64	60-64	3,410	2,500	783	514	Latin America and the Caribbean	
116	Colombia	2018	COPEN: Estudio Colombiano de Perfiles Nutricionales	20-64	20-64			609	520			Latin America and the Caribbean	
117	Colombia	2022	COPEN: Estudio Colombiano de Perfiles Nutricionales	20-64	20-64			686	532			Latin America and the Caribbean	
118	Colombia	2014-2015	Latin American Study of Nutrition and Health (ELANS)	20-64	20-64			345	325			Latin America and the Caribbean	
119	Comoros	2011	STEPS	25-64	25-64	25-64	25-64	3,455	1,518	3,444	1,518	Sub-Saharan Africa	
120	Congo	2004	STEPS	25-64	25-64	25-64	25-64	943	999	820	855	Sub-Saharan Africa	
121	Congo	2019	Diabetes prevalence and risk factors	20-64	20-64			688	582			Sub-Saharan Africa	
122	Cook Islands	2003	STEPS	25-64	25-64	25-64	25-64	951	910	949	899	Oceania	
123	Cook Islands	2013-2015	STEPS	20-64	20-64	20-64	20-64	435	419	432	418	Oceania	
124	Costa Rica	2004	CAMDI	20-64	20-64	20-64	20-64	732	420	727	419	Latin America and the Caribbean	
125	Costa Rica	2010	Costa Rican National Cardiovascular Risk Factors Survey, 2010	20-64	20-64	20-64	20-64	1,515	526	1,453	494	Latin America and the Caribbean	
126	Costa Rica	2014	Costa Rican National Cardiovascular Risk Factors Survey, 2014	20-64	20-64	20-64	20-64	1,748	704	1,735	699	Latin America and the Caribbean	
127	Costa Rica	2004-2006	Costa Rican Longevity and Healthy Aging Study Pre-1945 Cohort Wave 1	60-64	60-64	60-64	60-64	183	145	183	144	Latin America and the Caribbean	
128	Costa Rica	2010-2011	Costa Rican Longevity and Healthy Aging Study 1945-1955 Cohort Wave 1	54-64	54-64	54-64	54-64	1,543	973	1,529	967	Latin America and the Caribbean	
129	Costa Rica	2014-2015	Latin American Study of Nutrition and Health (ELANS)	20-64	20-64			344	321			Latin America and the Caribbean	
130	Cote d'Ivoire	2005	STEPS	20-64	20-64	20-64	20-64	2,058	1,691	2,014	1,656	Sub-Saharan Africa	
131	Cote d'Ivoire	2014	Prevalence and factors associated with obesity in a periurban west African population	20-64	20-64			292	140			Sub-Saharan Africa	
132	Croatia	2008	Endemic Nephropathy and Arterial hypertension (ENAH)	20-64	20-64	20-64	20-64	381	247	298	157	Central and eastern Europe	
133	Croatia	2010	Endemic Nephropathy and Arterial hypertension (ENAH)	20-64	20-64	20-64	20-64	273	188	211	132	Central and eastern Europe	
134	Croatia	2015	Endemic Nephropathy and Arterial hypertension (ENAH) Follow-up Study	20-64	20-64	20-64	20-64	315	157	314	157	Central and eastern Europe	
135	Croatia	2018-2021	Epidemiology of arterial hypertension in Croatia (EH-UH)	20-64	20-64	20-64	20-64	321	185	321	184	Central and eastern Europe	
136	Cuba	1991	Non communicable disease risk factors in Cienfuegos	20-64	20-64			456	397			Latin America and the Caribbean	
137	Cuba	2001	Non communicable disease risk factors in Cienfuegos	20-64	20-64	20-64	20-64	757	630	757	630	Latin America and the Caribbean	
138	Cuba	2001	National Survey on Risk Factors and Chronic Diseases (NSRFCD)	20-64	20-64			8,667	7,759			Latin America and the Caribbean	

	Country	Years	Survey/Study name/Citation	Age range used in anthropometric analyses		Age range used in hypertension analyses		Individuals used in anthropometric analyses		Individuals used in hypertension analyses		Region	Notes
				Women	Men	Women	Men	Women	Men	Women	Men		
139	Cuba	2010	National Survey on Risk Factors and Chronic Diseases (NSRFCD)	20-64	20-64	20-64	20-64	2,896	2,499	2,883	2,490	Latin America and the Caribbean	
140	Cuba	1999-2000	The Survey on Health, Well-Being, and Aging in Latin America and the Caribbean (SABE)	60-64	60-64	60-64	60-64	234	195	234	195	Latin America and the Caribbean	1
141	Cuba	2010-2011	Non communicable disease risk factors in Cienfuegos	20-64	20-64	20-64	20-64	643	468	643	468	Latin America and the Caribbean	
142	Czechia	1992	Czech-MONICA	25-64	25-64	25-64	25-64	1,206	1,129	1,204	1,124	Central and eastern Europe	
143	Czechia	2009	Health, Lifestyle and the Environment	45-54	45-54	45-54	45-54	446	307	443	305	Central and eastern Europe	
144	Czechia	1997-1998	Czech post-MONICA	25-64	25-64	25-64	25-64	1,665	1,527	1,661	1,526	Central and eastern Europe	
145	Czechia	1998-2002	Health, Lifestyle and the Environment	45-54	45-54	45-54	45-54	2,044	1,539	2,033	1,526	Central and eastern Europe	
146	Czechia	2000-2001	Czech post-MONICA	25-64	25-64	25-64	25-64	1,682	1,617	1,679	1,615	Central and eastern Europe	
147	Czechia	2002-2005	Health, Alcohol and Psychosocial Factors In Eastern Europe	45-64	45-64	45-64	45-64	3,129	2,491	3,107	2,468	Central and eastern Europe	
148	Czechia	2004-2005	Health, Lifestyle and the Environment	45-54	45-54	45-54	45-54	1,065	761	1,060	757	Central and eastern Europe	
149	Czechia	2006-2009	Czech post-MONICA	25-64	25-64	25-64	25-64	1,855	1,715	1,852	1,712	Central and eastern Europe	
150	Czechia	2014-2015	European Heath Examination Survey	25-64	25-64	25-64	25-64	691	471	691	471	Central and eastern Europe	
151	Czechia	2015-2018	Czech post-MONICA	25-64	25-64	25-64	25-64	1,343	1,220	1,329	1,213	Central and eastern Europe	
152	Czechia	2019-2020	European Heath Examination Survey	25-64	25-64	25-64	25-64	626	425	626	424	Central and eastern Europe	
153	Czechia	2019-2022	CELSPAC: YA (The Central European Longitudinal Studies of Parents and Children: Young Adults)	27-30	27-30	27-30	27-30	151	140	150	139	Central and eastern Europe	
154	Denmark	1993-1997	EPIC Aarhus	50-64	50-64	50-64	50-64	8,597	8,318	8,469	8,131	High-income western	
155	Denmark	1993-1997	EPIC Copenhagen	50-64	50-64	50-64	50-64	20,854	18,502	20,599	18,153	High-income western	
156	Denmark	2007-2008	The Danish Health Examination Survey 2007-2008	20-64	20-64	20-64	20-64	8,556	5,467	8,552	5,466	High-income western	
157	Denmark	2009-2010	European Youth Heart Study	20-28	20-28			333	305			High-income western	
158	Dominica	2007-2008	STEPS	20-64	20-64	20-64	20-64	485	372	452	307	Latin America and the Caribbean	
159	Dominican Republic	2017	Prevalencia de hta y factores de riesgo en La Republica Dominicana al 2017 (ENPREFAR HAS 17)	20-64	20-64	20-64	20-64	822	766	822	766	Latin America and the Caribbean	
160	Dominican Republic	2010-2012	Estudio factores de riesgo cardiovascular y sindrome metabolico en la Republica Dominicana II (EFRICARD II)	20-64	20-64	20-64	20-64	2,682	1,321	2,674	1,317	Latin America and the Caribbean	
161	DR Congo	2005	STEPS	20-64	20-64	20-64	20-64	835	551	833	549	Sub-Saharan Africa	
162	DR Congo	2007	Diabetes and intermediate hyperglycaemia in Kisantu, DR Congo: a cross-sectional prevalence study	20-64	20-64	20-64	20-64	926	451	897	446	Sub-Saharan Africa	
163	DR Congo	2008	Visite de la Tension Artérielle et des Facteurs de Risque Associés en Afrique subsaharienne (VITARAA) - Kinshasa, RD Congo	20-64	20-64	20-64	20-64	665	509	647	496	Sub-Saharan Africa	
164	DR Congo	2016-2017	Prevalence and Risk Factors of CKD in South Kivu, Democratic Republic of Congo: A Large-Scale Population Study	20-64	20-64	20-64	20-64	652	412	647	411	Sub-Saharan Africa	
165	Ecuador	2018	STEPS	20-64	20-64	20-64	20-64	2,311	1,658	2,307	1,658	Latin America and the Caribbean	
166	Ecuador	2009-2010	The Survey on Health, Well-Being, and Aging in Latin America and the Caribbean (SABE)	60-64	60-64	60-64	60-64	671	622	658	612	Latin America and the Caribbean	
167	Ecuador	2011-2013	Encuesta Nacional de Salud y Nutrición (ENSANUT)	20-59	20-59	20-59	20-59	14,903	12,193	14,866	12,164	Latin America and the Caribbean	
168	Ecuador	2014-2015	Latin American Study of Nutrition and Health (ELANS)	20-64	20-64			509	426			Latin America and the Caribbean	
169	Egypt	2005	STEPS	20-64	20-64	20-64	20-64	3,159	3,138	3,001	2,975	Central Asia, Middle East and north Africa	
170	Egypt	2011	STEPS	20-64	20-64	20-64	20-64	2,562	1,442	2,557	1,440	Central Asia, Middle East and north Africa	
171	Egypt	2017	STEPS	20-64	20-64	20-64	20-64	3,030	1,719	3,028	1,718	Central Asia, Middle East and north Africa	
172	Egypt	2003-2004	Marzouk et al., Gut 56(8):1105-10, 2007	25-64	25-64	25-64	25-64	422	283	415	277	Central Asia, Middle East and north Africa	
173	Egypt	2007-2009	Mostafa et al., Gut 59(8):1135-40, 2010	35-64	35-64	35-64	35-64	761	557	749	548	Central Asia, Middle East and north Africa	
174	El Salvador	2004	CAMDI	20-64	20-64	20-64	20-64	722	363	722	363	Latin America and the Caribbean	
175	El Salvador	2014-2015	Encuesta Nacional de Enfermedades Crónicas (ENECA-ELS)	20-64	20-64	20-64	20-64	2,519	1,374	2,514	1,370	Latin America and the Caribbean	
176	Eritrea	2004	STEPS	20-64	20-64	20-64	20-64	942	930	924	903	Sub-Saharan Africa	
177	Eritrea	2010	STEPS	25-64	25-64	25-64	25-64	3,878	1,350	3,876	1,350	Sub-Saharan Africa	
178	Estonia	2003	The European Male Ageing Study		40-64		40-64		279		274	Central and eastern Europe	
179	Estonia	2013-2015	National Dietary Survey (RTU) 2014	20-64	20-64			1,358	696			Central and eastern Europe	
180	Eswatini	2014	STEPS	20-64	20-64	20-64	20-64	1,618	851	1,597	850	Sub-Saharan Africa	
181	Ethiopia	2006	STEPS	25-64	25-64	25-64	25-64	2,300	1,621	2,201	1,519	Sub-Saharan Africa	
182	Ethiopia	2015	STEPS	20-64	20-64	20-64	20-64	4,618	3,321	4,613	3,318	Sub-Saharan Africa	
183	Federated States of Micronesia	2002	STEPS	25-64	25-64	25-64	25-64	872	578	784	512	Oceania	
184	Federated States of Micronesia	2006	STEPS	20-64	20-64	20-64	20-64	1,313	746	1,297	733	Oceania	
185	Federated States of Micronesia	2008	STEPS	25-64	25-64	25-64	25-64	1,219	819	1,211	813	Oceania	
186	Federated States of Micronesia	2009	STEPS, Kosrae	20-64	20-64	20-64	20-64	394	187	382	184	Oceania	
187	Federated States of Micronesia	2009	STEPS, Yap	20-64	20-64	20-64	20-64	466	336	457	332	Oceania	
188	Federated States of Micronesia	2016	STEPS	20-64	20-64	20-64	20-64	769	466	768	466	Oceania	
189	Fiji	2002	STEPS	20-64	20-64	20-64	20-64	3,387	2,352	3,364	2,335	Oceania	
190	Fiji	2011	STEPS	25-64	25-64	25-64	25-64	1,373	1,051	1,336	1,017	Oceania	
191	Fiji	2007-2008	Pacific Obesity Prevention in Communities – Healthy Youth Health Communities Study	20-22	20-22			19	13			Oceania	
192	Finland	1997	Northern Finland Birth Cohort 1966	30-31	30-31	30-31	30-31	95	2,608	94	2,577	High-income western	
193	Finland	2001	Young Finns Study 2001	24-39	24-39	24-39	24-39	1,162	1,003	1,155	999	High-income western	
194	Finland	2008	Control group for Finnish male former elite athletes		61-64		61-64		18		18	High-income western	
195	Finland	2017	The FinHealth Survey	20-64	20-64	20-64	20-64	2,124	1,927	2,093	1,894	High-income western	
196	Finland	1991-1993	Kuopio Ischaemic Heart Disease Risk Factor Study		46-64		46-64		991		990	High-income western	
197	Finland	1996-1998	Oulu 35 Study	60-63	60-63	60-63	60-63	342	241	334	230	High-income western	
198	Finland	1996-1998	Savitaipale Study, Baseline	40-64	40-64	40-64	40-64	377	290	377	290	High-income western	
199	Finland	2000-2001	Health 2000 Survey	30-64	30-64	30-64	30-64	2,332	2,143	2,318	2,121	High-income western	
200	Finland	2001-2003	Oulu 45 Study	55-58	55-58	55-58	55-58	546	424	546	424	High-income western	
201	Finland	2001-2004	Helsinki Birth Cohort Study	56-64	56-64			894	791			High-income western	
202	Finland	2007-2008	Savitaipale Study, 10-year Follow-up	51-64	51-64	51-64	51-64	229	193	222	187	High-income western	
203	Finland	2011-2012	Health 2011 Survey	30-64	30-64	30-64	30-64	1,767	1,456	1,726	1,395	High-income western	
204	Finland	2019-2020	Northern Finland Birth Cohort 1986	33-35	33-35	33-35	33-35	900	579	892	579	High-income western	
205	France	2006-2007	Étude Nationale Nutrition Santé	20-64	20-64	20-64	20-64	1,114	669	1,086	661	High-income western	4
206	France	2012-2014	Cohorte des consultants des Centres d'examens de santé	20-64	20-64			23,266	19,517			High-income western	

	Country	Years	Survey/Study name/Citation	Age range used in anthropometric analyses		Age range used in hypertension analyses		Individuals used in anthropometric analyses		Individuals used in hypertension analyses		Region	Notes
				Women	Men	Women	Men	Women	Men	Women	Men		
207	France	2014-2016	L'Etude de Sante sur l'Environnement, la Biosurveillance, l'Activite physique et la Nutrition (Etude Esteban)	20-64	20-64	20-64	20-64	1,077	872	1,074	871	High-income western	5
208	France	2015-2017	Cohorte des consultants des Centres d'examens de santé	20-64	20-64			45,543	39,861			High-income western	
209	France	2018-2019	Cohorte des consultants des Centres d'examens de santé	20-64	20-64			24,879	21,420			High-income western	
210	France	2020-2021	Cohorte des consultants des Centres d'examens de santé	20-64	20-64			1,681	1,338			High-income western	
211	French Polynesia	2010	STEPS	20-64	20-64	20-64	20-64	1,794	1,355	1,202	904	Oceania	
212	Gabon	2009	STEPS	20-64	20-64	20-64	20-64	1,000	673	998	673	Sub-Saharan Africa	
213	Gambia	2010	STEPS	25-64	25-64	25-64	25-64	1,627	1,295	1,620	1,295	Sub-Saharan Africa	
214	Georgia	2010	STEPS	20-64	20-64	20-64	20-64	4,195	1,645	4,193	1,642	Central Asia, Middle East and north Africa	
215	Georgia	2016	STEPS	20-64	20-64	20-64	20-64	2,349	989	2,337	985	Central Asia, Middle East and north Africa	
216	Germany	2002	Echinoccoccus Multilocularis and Internal Diseases in Leutkirch	20-64	20-64			1,075	1,008			High-income western	
217	Germany	1994-1998	EPIC Heidelberg	35-64	40-64	35-64	40-64	13,443	11,670	5,357	4,390	High-income western	
218	Germany	1994-1998	EPIC Potsdam	35-64	40-64	35-64	40-64	15,987	10,222	15,215	9,530	High-income western	6
219	Germany	1997-1999	German National Health Interview and Examination Survey (GNHIES98)	20-64	20-64	20-64	20-64	2,917	2,852	2,899	2,841	High-income western	
220	Germany	1997-2001	Study of Health in Pomerania (SHIP-START-0) baseline study	20-64	20-64	20-64	20-64	1,733	1,564	1,728	1,559	High-income western	7
221	Germany	2000-2003	Heinz Nixdorf Recall Study	45-64	45-64	45-64	45-64	1,693	1,673	1,677	1,655	High-income western	8
222	Germany	2008-2011	Epidemiological study of the chances of prevention, early recognition and optimal treatment of chronic diseases in an elderly population (ESTHER)	58-64	58-64	58-64	58-64	473	352	408	303	High-income western	
223	Germany	2008-2011	German Health Interview and Examination Survey for adults 2008-11 (DEGS1)	20-64	20-64	20-64	20-64	2,635	2,341	2,614	2,330	High-income western	
224	Germany	2008-2012	Study of Health in Pomerania, second cohort (SHIP-TREND-0)	20-64	20-64	20-64	20-64	1,736	1,566	1,733	1,559	High-income western	7
225	Ghana	2003	Women's Health Study of Accra (WHSA-I)	20-64		20-64		845		840		Sub-Saharan Africa	
226	Ghana	2006	STEPS	25-64	25-64	25-64	25-64	1,609	827	1,602	824	Sub-Saharan Africa	
227	Ghana	2007-2008	WHO Study on global AGEing and adult health (SAGE)	50-64	50-64	50-64	50-64	995	1,236	987	1,225	Sub-Saharan Africa	
228	Ghana	2008-2010	Women's Health Study of Accra (WHSA-II)	20-64		20-64		2,072		2,057		Sub-Saharan Africa	
229	Ghana	2012-2014	Research on Obesity and Diabetes among African Migrants (RODAM), control group	25-64	25-64	25-64	25-64	1,565	751	1,563	750	Sub-Saharan Africa	
230	Greece	2016	SKG-Elderly	60-64	60-64	60-64	60-64	11	9	11	9	High-income western	
231	Greece	2018	STEPS-Thessaloniki	60-64	60-64	60-64	60-64	35	61	35	61	High-income western	
232	Greece	2013-2015	Hellenic National Nutrition and Health Survey (HNNHS)	20-64	20-64	20-64	20-64	566	355	565	354	High-income western	
233	Greece	2013-2016	National Survey of Morbidity and Risk Factors (EMENO)	20-64	20-64	20-64	20-64	1,875	1,356	1,852	1,344	High-income western	
234	Grenada	2011	STEPS	25-64	25-64	25-64	25-64	631	430	630	429	Latin America and the Caribbean	
235	Guatemala	2015	STEPS	20-64	20-64	20-64	20-64	1,156	325	1,148	321	Latin America and the Caribbean	
236	Guatemala	2016	Sistema de vigilancia Epidemiológica de Salud y Nutrición (SIVESNU)	20-49		20-49		1,270		1,230		Latin America and the Caribbean	
237	Guatemala	2001-2002	CAMDI	20-64	20-64	20-64	20-64	653	280	653	280	Latin America and the Caribbean	
238	Guatemala	2003-2005	The Institute of Nutrition of Central America and Panama Nutrition Supplementation Trial Cohort	25-41	25-41	25-41	25-41	287	259	271	204	Latin America and the Caribbean	
239	Guatemala	2008-2009	Encuesta Nacional de Salud Materno Infantil	20-49	20-59			12,349	5,368			Latin America and the Caribbean	
240	Guatemala	2015-2017	Nutrition on early childhood and metabolomic and cardiometabolic profile on adulthood (META)	37-55	37-55			311	213			Latin America and the Caribbean	
241	Guatemala	2017-2018	Sistema de vigilancia Epidemiológica de Salud y Nutrición (SIVESNU)	20-49		20-49		1,288		1,198		Latin America and the Caribbean	
242	Guatemala	2018-2019	Population-Based Survey of Chronic Kidney Disease in Guatemala	20-64	20-64			442	224			Latin America and the Caribbean	
243	Guatemala	2018-2019	Sistema de vigilancia Epidemiológica de Salud y Nutrición (SIVESNU)	20-49		20-49		1,322		1,268		Latin America and the Caribbean	
244	Guinea	2009	STEPS	20-64	20-64	20-64	20-64	1,024	967	1,008	955	Sub-Saharan Africa	
245	Guyana	2016	STEPS	20-64	20-64	20-64	20-64	1,348	904	1,348	903	Latin America and the Caribbean	
246	Haiti	2015-2016	Haiti Health Study (Carrefour)	25-64	25-64			763	529			Latin America and the Caribbean	
247	Haiti	2015-2016	Haiti Health Study (Thomonde)	25-64	25-64	25-64	25-64	398	241	397	241	Latin America and the Caribbean	
248	Honduras	2003-2004	CAMDI	20-64	20-64	20-64	20-64	678	359	677	359	Latin America and the Caribbean	
249	Hungary	2003	The European Male Ageing Study		40-64		40-64		292		285	Central and eastern Europe	
250	India	1995	Shobana et al., Diabetes Res Clin Pract 42(3):181–86, 1998	20-64	20-64			1,049	1,034			South Asia	
251	India	1997	Ramachandran et al., Diabetes Res Clin Pract 44(3):207–13, 1999	20-64	20-64			837	700			South Asia	
252	India	2000	Ramachandran et al., Diabet Med 20(3):220-24, 2003	20-64	20-64			4,866	4,281			South Asia	
253	India	2006	Ramachandran et al., Diabetes Care 31(5):893-98, 2008	20-64	20-64			3,690	3,258			South Asia	
254	India	2010	Kerala 2010 follow-up	21-64	21-64	21-64	21-64	210	185	210	185	South Asia	
255	India	2021	STEPS	20-64	20-64	20-64	20-64	2,289	2,282	2,239	2,237	South Asia	
256	India	1992-1994	Jaipur Heart Watch	20-64	20-64	20-64	20-64	192	523	103	184	South Asia	
257	India	1998-2002	Vellore Birth Cohort	25-31	25-31	25-31	25-31	1,050	1,158	1,049	1,158	South Asia	
258	India	1999-2001	Jaipur Heart Watch 2	20-64	20-64			459	393			South Asia	
259	India	1999-2002	New Delhi Birth Cohort	26-33	26-33	26-33	26-33	638	886	629	880	South Asia	
260	India	2001-2004	Chennai Urban Rural Epidemiology Study	20-64	20-64	20-64	20-64	1,188	984	1,187	983	South Asia	
261	India	2003-2004	Jaipur Heart Watch 3	20-64	20-64	20-64	20-64	210	182	209	180	South Asia	
262	India	2005-2006	Risk factor profile for chronic non-communicable diseases: Results of a community-based study in Kerala, India	20-64	20-64	20-64	20-64	2,714	2,522	2,714	2,522	South Asia	
263	India	2005-2006	National Nutrition Monitoring Bureau rural survey	20-64	20-64	20-64	20-64	12,415	10,428	12,415	10,426	South Asia	
264	India	2006-2007	Jaipur Heart Watch 4	20-64	20-64	20-64	20-64	511	443	489	415	South Asia	
265	India	2007-2008	Urban population in Hyderabad	20-60	20-60	20-60	20-60	1,555	1,513	1,536	1,499	South Asia	
266	India	2007-2008	WHO Study on global AGEing and adult health (SAGE)	50-64	50-64	50-64	50-64	2,063	1,909	2,059	1,906	South Asia	
267	India	2009-2010	Jaipur Heart Watch 5	20-64	20-64	20-64	20-64	234	356	229	350	South Asia	
268	India	2009-2010	Baseline Survey for the assessment of prevalence of risk factors of NCDs in Gandhinagar District	20-64	20-64	20-64	20-64	1,506	1,535	1,505	1,535	South Asia	
269	India	2010-2012	Centre for cArdiometabolic Risk Reduction in South-Asia (CARRS) - Surveillance Study	20-64	20-64	20-64	20-64	4,754	3,792	4,750	3,788	South Asia	9
270	India	2011-2012	National Nutrition Monitoring Bureau rural survey	20-64	20-64	20-64	20-64	23,886	18,792	23,836	18,749	South Asia	
271	India	2012-2013	Processed and non-processed foods	20-64	20-64			4,720	4,392			South Asia	
272	India	2012-2014	Jaipur Heart Watch 6	20-64	20-64			658	864			South Asia	
273	India	2014-2015	Control of Hypertension In Rural India (CHIRI) - Rishi Valley	20-64	20-64			2,954	1,986			South Asia	
274	India	2014-2015	Control of Hypertension In Rural India (CHIRI) - Trivandrum	20-64	20-64	20-64	20-64	1,514	1,450	1,513	1,450	South Asia	
275	India	2014-2015	Control of Hypertension In Rural India (CHIRI) - West Godavari	20-64	20-64	20-64	20-64	1,766	1,733	1,764	1,730	South Asia	

	Country	Years	Survey/Study name/Citation	Age range used in anthropometric analyses		Age range used in hypertension analyses		Individuals used in anthropometric analyses		Individuals used in hypertension analyses		Region	Notes
				Women	Men	Women	Men	Women	Men	Women	Men		
276	India	2015-2016	Diet and nutritional status of urban population and prevalence of hypertension	20-64	20-64	20-64	20-64	57,541	44,774	47,153	33,204	South Asia	
277	India	2016-2017	Secular TRends in DiabEtes in India (STRiDE-I) -Change in Prevalence in Ten Years among Urban and Rural Populations in Tamil Nadu	20-64	20-64			5,068	4,156			South Asia	
278	India	2017-2019	Longitudinal Aging Study in India	45-64	45-64	45-64	45-64	22,197	18,017	22,174	17,996	South Asia	
279	India	2019-2020	Prevalence, awareness, treatment and control of hypertension among adults aged 30 years and above in Barmer district, Rajasthan, India	30-64	30-64	30-64	30-64	124	137	124	137	South Asia	
280	India	2019-2021	DHS	20-49	20-54	20-49	20-54	554,801	79,721	553,278	79,510	South Asia	
281	Indonesia	2003	A genetic-ecological study of the risk factors for lifestyle-related diseases in Oceanian populations, Study A	20-64	20-64	20-64	20-64	87	94	87	94	East and southeast Asia and the Pacific	
282	Indonesia	2003	A genetic-ecological study of the risk factors for lifestyle-related diseases in Oceanian populations, Study B	20-64	20-64	20-64	20-64	118	81	118	81	East and southeast Asia and the Pacific	
283	Indonesia	2007	Indonesian Basic Health Survey (RISKESDAS) 2007	20-64	20-64			265,659	251,280			East and southeast Asia and the Pacific	
284	Indonesia	2013	Indonesian Basic Health Survey (RISKESDAS) 2013	20-64	20-64	20-64	20-64	286,480	266,891	286,026	266,540	East and southeast Asia and the Pacific	
285	Indonesia	2018	Indonesian Basic Health Survey (RISKESDAS) 2018	20-64	20-64	20-64	20-64	286,489	264,160	286,156	263,750	East and southeast Asia and the Pacific	
286	Indonesia	2000-2001	Indonesian Family Life Surveys	20-64	20-64	20-64	20-64	3,872	3,443	3,856	3,439	East and southeast Asia and the Pacific	
287	Indonesia	2007-2008	Indonesian Family Life Surveys	20-64	20-64	20-64	20-64	4,556	4,110	4,455	4,017	East and southeast Asia and the Pacific	
288	Indonesia	2014-2015	Indonesian Family Life Surveys	20-64	20-64	20-64	20-64	5,631	5,216	5,558	5,172	East and southeast Asia and the Pacific	
289	Iran	2001	Isfahan Healthy Heart Programme (IHHP), Arak	20-64	20-64	20-64	20-64	2,814	2,661	2,802	2,653	Central Asia, Middle East and north Africa	
290	Iran	2001	Isfahan Healthy Heart Programme (IHHP), Isfahan	20-64	20-64	20-64	20-64	1,882	1,721	1,874	1,713	Central Asia, Middle East and north Africa	
291	Iran	2001	Isfahan Healthy Heart Programme (IHHP), Najaf Abad	20-64	20-64	20-64	20-64	901	898	898	895	Central Asia, Middle East and north Africa	
292	Iran	2005	STEPS	20-64	20-64	20-64	20-64	35,369	35,725	35,113	35,463	Central Asia, Middle East and north Africa	
293	Iran	2006	STEPS	20-64	20-64	20-64	20-64	13,136	13,064	13,075	12,984	Central Asia, Middle East and north Africa	
294	Iran	2007	STEPS	20-64	20-64	20-64	20-64	2,155	2,151	2,147	2,138	Central Asia, Middle East and north Africa	
295	Iran	2007	STEPS	20-64	20-64	20-64	20-64	13,137	13,072	13,123	13,059	Central Asia, Middle East and north Africa	
296	Iran	2007	Isfahan Healthy Heart Programme (IHHP), Arak	20-64	20-64	20-64	20-64	1,565	1,521	1,558	1,509	Central Asia, Middle East and north Africa	
297	Iran	2007	Isfahan Healthy Heart Programme (IHHP), Isfahan	20-64	20-64	20-64	20-64	1,282	1,289	1,265	1,280	Central Asia, Middle East and north Africa	
298	Iran	2007	Isfahan Healthy Heart Programme (IHHP), Najaf Abad	20-64	20-64	20-64	20-64	717	670	705	662	Central Asia, Middle East and north Africa	
299	Iran	2008	STEPS	20-64	20-64	20-64	20-64	12,845	12,854	12,828	12,840	Central Asia, Middle East and north Africa	
300	Iran	2009	STEPS	20-64	20-64	20-64	20-64	13,074	13,156	13,026	13,138	Central Asia, Middle East and north Africa	
301	Iran	2011	STEPS	20-64	20-64	20-64	20-64	4,986	3,509	3,480	2,028	Central Asia, Middle East and north Africa	
302	Iran	2016	STEPS	20-64	20-64	20-64	20-64	12,866	11,847	12,849	11,826	Central Asia, Middle East and north Africa	
303	Iran	2003-2004	The Persian Gulf Healthy Heart Study	25-64	25-64	25-64	25-64	1,900	1,651	1,894	1,649	Central Asia, Middle East and north Africa	
304	Iran	2004-2008	Golestan Cohort Study Main Phase	40-64	40-64	40-64	40-64	26,190	18,265	26,158	18,247	Central Asia, Middle East and north Africa	
305	Iran	2008-2011	Tehran Lipid and Glucose Study	20-64	20-64	20-64	20-64	5,196	3,877	5,165	3,867	Central Asia, Middle East and north Africa	
306	Iran	2009-2010	The Persian Gulf Healthy Heart Study	31-64	31-64	31-64	31-64	964	764	962	763	Central Asia, Middle East and north Africa	
307	Iran	2011-2012	Amol county study	20-64	20-64	20-64	20-64	2,146	2,621	2,146	2,620	Central Asia, Middle East and north Africa	
308	Iran	2012-2013	Tehran City	20-64	20-64	20-64	20-64	457	340	457	339	Central Asia, Middle East and north Africa	
309	Iran	2012-2013	Zahedan City	20-64	20-64	20-64	20-64	947	984	946	984	Central Asia, Middle East and north Africa	
310	Iran	2012-2014	Pars Cohort Study	40-64	40-64	40-64	40-64	4,355	3,725	4,348	3,718	Central Asia, Middle East and north Africa	
311	Iran	2013-2014	Bushehr Elderly Health Program (BEH)	60-64	60-64	60-64	60-64	351	342	351	342	Central Asia, Middle East and north Africa	
312	Iran	2013-2014	Gilan Eye Study	50-64	50-64	50-64	50-64	976	573	932	545	Central Asia, Middle East and north Africa	
313	Iran	2014-2016	The PERSIAN Fasa Cohort Study	35-64	35-64	35-64	35-64	5,074	4,265	5,071	4,260	Central Asia, Middle East and north Africa	
314	Iran	2014-2016	The PERSIAN Guilan Cohort Study	35-64	35-64	35-64	35-64	5,305	4,562	5,305	4,562	Central Asia, Middle East and north Africa	
315	Iran	2014-2016	The PERSIAN Kermanshah Cohort Study	35-64	35-64	35-64	35-64	5,156	4,740	5,155	4,739	Central Asia, Middle East and north Africa	
316	Iran	2014-2016	The PERSIAN Kharameh Cohort Study	35-64	35-64	35-64	35-64	5,337	4,314	5,337	4,314	Central Asia, Middle East and north Africa	
317	Iran	2014-2016	The PERSIAN Tabriz Cohort Study	35-64	35-64	35-64	35-64	7,604	6,160	7,600	6,160	Central Asia, Middle East and north Africa	
318	Iran	2015-2017	The PERSIAN Yazd Cohort Study	30-64	30-64	30-64	30-64	4,566	4,647	4,478	4,495	Central Asia, Middle East and north Africa	
319	Iran	2015-2017	The PERSIAN Mazandaran Cohort Study	35-64	35-64	35-64	35-64	5,650	3,723	5,650	3,723	Central Asia, Middle East and north Africa	
320	Iran	2015-2017	The PERSIAN Rafsanjan Cohort Study	35-64	35-64	35-64	35-64	4,979	4,813	4,951	4,774	Central Asia, Middle East and north Africa	
321	Iran	2015-2018	The PERSIAN Zahedan Cohort Study	35-64	35-64	35-64	35-64	5,704	3,571	5,673	3,564	Central Asia, Middle East and north Africa	
322	Iran	2016-2018	The PERSIAN Ahvaz Cohort Study	35-64	35-64	35-64	35-64	5,427	3,723	5,426	3,723	Central Asia, Middle East and north Africa	
323	Iran	2016-2018	The PERSIAN BandarKong Cohort Study	35-64	35-64	35-64	35-64	2,132	1,602	2,121	1,592	Central Asia, Middle East and north Africa	
324	Iran	2016-2018	The PERSIAN Urmia Cohort Study	35-64	35-64	35-64	35-64	2,574	1,993	2,526	1,951	Central Asia, Middle East and north Africa	
325	Iran	2016-2019	The Khuzestan comprehensive health study: A platform for NCDs, blood borne and mental diseases research	20-64	20-64	20-64	20-64	19,350	10,749	19,332	10,737	Central Asia, Middle East and north Africa	
326	Iran	2016-2019	The PERSIAN Shahrekord Cohort Study	35-64	35-64	35-64	35-64	4,842	4,192	4,752	4,114	Central Asia, Middle East and north Africa	
327	Iran	2016-2020	The PERSIAN Ardabil Cohort Study	35-64	35-64	35-64	35-64	10,698	8,983	10,668	8,940	Central Asia, Middle East and north Africa	
328	Iran	2017-2018	The PERSIAN Kavar Cohort Study	35-64	35-64	35-64	35-64	2,428	2,308	2,428	2,308	Central Asia, Middle East and north Africa	
329	Iran	2017-2018	PERSIAN Elderly Component-Iranian Longitudinal Study on Ageing	50-64	50-64	50-64	50-64	2,951	2,342	2,691	2,165	Central Asia, Middle East and north Africa	
330	Iran	2017-2019	The PERSIAN Sabzevar Cohort Study	35-64	35-64	35-64	35-64	2,229	1,747	2,204	1,726	Central Asia, Middle East and north Africa	
331	Iran	2017-2019	The PERSIAN Dena (Yasouj) Cohort Study	35-64	35-64	35-64	35-64	1,714	1,317	1,683	1,294	Central Asia, Middle East and north Africa	
332	Iran	2018-2019	Prevalence of risk factors for cardiovascular disease among a rural population in eastern Iran	20-64	20-64	20-64	20-64	64	70	64	70	Central Asia, Middle East and north Africa	
333	Iran	2018-2019	The PERSIAN Dehgolan (Kordistan) Cohort Study	35-64	35-64	35-64	35-64	2,074	1,628	2,060	1,608	Central Asia, Middle East and north Africa	
334	Iran	2020-2021	STEPS	20-64	20-64	20-64	20-64	12,886	10,084	12,880	10,077	Central Asia, Middle East and north Africa	
335	Iraq	2015	STEPS	20-64	20-64	20-64	20-64	2,005	1,293	1,723	988	Central Asia, Middle East and north Africa	
336	Ireland	2002	Survey of Lifestyle, Attitudes and Nutritional in Ireland 2002	20-64	20-64			175	132			High-income western	
337	Ireland	2006-2007	Survey of Lifestyle, Attitudes and Nutritional in Ireland 2006-2007	20-64	20-64	45-64	45-64	978	714	470	326	High-income western	
338	Ireland	2009-2011	The Irish Longitudinal Study on Ageing	50-64	50-64	50-64	50-64	1,944	1,554	1,942	1,553	High-income western	
339	Israel	1999-2005	The Israel Glucose Intolerance, Obesity and Hypertension Study (GOH)	58-64	58-64	58-64	58-64	58	73	55	72	High-income western	
340	Israel	2002-2008	The Hadera District Study (HDS)	25-64	25-64	25-64	25-64	297	302	296	302	High-income western	
341	Italy	1990	Bruneck Study	40-64	40-64	40-64	40-64	285	317	285	317	High-income western	
342	Italy	2003	The European Male Ageing Study		40-64		40-64		273		269	High-income western	
343	Italy	1989-1992	Gubbio Study	20-64	20-64	20-64	20-64	1,288	1,099	1,287	1,099	High-income western	

	Country	Years	Survey/Study name/Citation	Age range used in anthropometric analyses		Age range used in hypertension analyses		Individuals used in anthropometric analyses		Individuals used in hypertension analyses		Region	Notes
				Women	Men	Women	Men	Women	Men	Women	Men		
344	Italy	1993-1996	Malattie cardiovascolari ATerosclerotiche Istituto Superiore di Sanità (MATISS)	20-64	20-64	20-64	20-64	938	900	938	900	High-income western	
345	Italy	1993-1998	EPIC Florence	24-64	24-64	24-64	24-64	9,713	2,634	9,106	2,555	High-income western	
346	Italy	1995-1996	Friuli Studio Emostatico	45-64	45-64	45-64	45-64	198	198	198	198	High-income western	
347	Italy	1998-1999	Progetto VIP	25-64	25-64	25-64	25-64	468	465	466	464	High-income western	
348	Italy	1998-2002	Osservatorio Epidemiologico Cardiovascolare (OEC)	35-64	35-64	35-64	35-64	3,623	3,655	3,621	3,652	High-income western	
349	Italy	2001-2003	The Study of Asti	45-64	45-64	45-64	45-64	877	779	877	779	High-income western	
350	Italy	2005-2007	Moli-family Study	20-64	20-64	20-64	20-64	203	174	203	174	High-income western	
351	Italy	2005-2010	Moli-sani Study	35-64	35-64	35-64	35-64	9,757	8,720	9,711	8,653	High-income western	
352	Italy	2008-2009	Progetto VIP	25-64	25-64	25-64	25-64	476	477	476	477	High-income western	
353	Italy	2008-2012	Osservatorio Epidemiologico Cardiovascolare/Health Examination Survey (OEC/HES)	35-64	35-64	35-64	35-64	2,979	2,939	2,972	2,928	High-income western	
354	Italy	2010-2012	CArdiovascular risk MEtabolic syndrome LIver and Autoimmunity diseases (CA.ME.LI.A)	20-64	20-64	20-64	20-64	411	382	395	352	High-income western	
355	Italy	2017-2020	Moli-sani Study	47-64	47-64	47-64	47-64	637	386	635	386	High-income western	
356	Italy	2018-2019	Progetto VIP	25-64	25-64	25-64	25-64	477	478	477	478	High-income western	
357	Italy	2018-2019	Health Examination Survey 2018-2019 - CUORE Project	35-64	35-64	35-64	35-64	787	775	783	773	High-income western	
358	Jamaica	2000-2001	Jamaica Health and Lifestyle Survey	20-64	20-64	20-64	20-64	1,030	478	1,014	465	Latin America and the Caribbean	
359	Jamaica	2007-2008	Jamaica Health and Lifestyle Survey	20-64	20-64	20-64	20-64	1,555	662	1,549	660	Latin America and the Caribbean	
360	Jamaica	2016-2017	Jamaica Health and Lifestyle Survey	20-64	20-64	20-64	20-64	1,084	633	1,070	625	Latin America and the Caribbean	
361	Japan	2003	The 2003 National Health and Nutrition Survey	20-64	20-64	20-64	20-64	2,499	1,840	2,221	1,384	East and southeast Asia and the Pacific	
362	Japan	2004	The 2004 National Health and Nutrition Survey	20-64	20-64	20-64	20-64	1,733	1,054	1,686	1,001	East and southeast Asia and the Pacific	
363	Japan	2005	The 2005 National Health and Nutrition Survey	20-64	20-64	20-64	20-64	1,533	975	1,492	936	East and southeast Asia and the Pacific	
364	Japan	2006	The 2006 National Health and Nutrition Survey	20-64	20-64	20-64	20-64	2,285	1,842	1,748	1,104	East and southeast Asia and the Pacific	
365	Japan	2007	The 2007 National Health and Nutrition Survey	20-64	20-64	20-64	20-64	2,250	1,795	1,668	1,036	East and southeast Asia and the Pacific	
366	Japan	2008	The 2008 National Health and Nutrition Survey	20-64	20-64	20-64	20-64	2,216	1,759	1,656	1,032	East and southeast Asia and the Pacific	
367	Japan	2009	The 2009 National Health and Nutrition Survey	20-64	20-64	20-64	20-64	2,214	1,819	1,629	1,067	East and southeast Asia and the Pacific	
368	Japan	2010	The 2010 National Health and Nutrition Survey	20-64	20-64	20-64	20-64	2,010	1,645	1,444	957	East and southeast Asia and the Pacific	
369	Japan	2011	The 2011 National Health and Nutrition Survey	20-64	20-64	20-64	20-64	1,847	1,532	1,336	863	East and southeast Asia and the Pacific	
370	Japan	2012	The 2012 National Health and Nutrition Survey	20-64	20-64	20-64	20-64	6,994	5,508	5,276	3,432	East and southeast Asia and the Pacific	
371	Japan	2013	The 2013 National Health and Nutrition Survey	20-64	20-64	20-64	20-64	1,727	1,457	1,148	739	East and southeast Asia and the Pacific	
372	Japan	2014	The 2014 National Health and Nutrition Survey	20-64	20-64	20-64	20-64	1,816	1,494	1,193	739	East and southeast Asia and the Pacific	
373	Japan	2015	The 2015 National Health and Nutrition Survey	20-64	20-64	20-64	20-64	1,719	1,331	1,202	690	East and southeast Asia and the Pacific	
374	Japan	2016	The 2016 National Health and Nutrition Survey	20-64	20-64	20-64	20-64	5,262	4,195	3,754	2,402	East and southeast Asia and the Pacific	
375	Japan	2017	The 2017 National Health and Nutrition Survey	20-64	20-64	20-64	20-64	1,395	1,168	950	595	East and southeast Asia and the Pacific	
376	Japan	2018	The 2018 National Health and Nutrition Survey	20-64	20-64	20-64	20-64	1,449	1,226	976	616	East and southeast Asia and the Pacific	
377	Japan	2019	The 2019 National Health and Nutrition Survey	20-64	20-64	20-64	20-64	1,166	944	750	464	East and southeast Asia and the Pacific	
378	Japan	2014-2015	Nagaoka Health Screening	20-64	20-64	20-64	20-64	3,376	3,954	3,010	3,658	East and southeast Asia and the Pacific	
379	Jordan	2004	Behavioural Risk Factor Surveillence Survey	20-64	20-64	20-64	20-64	394	181	393	181	Central Asia, Middle East and north Africa	
380	Jordan	2007	Behavioural Risk Factor Surveillence Survey	20-64	20-64	20-64	20-64	360	269	355	263	Central Asia, Middle East and north Africa	
381	Jordan	2009	Metablic abnomalities and vitamin D study	20-64	20-64	20-64	20-64	2,995	980	2,983	976	Central Asia, Middle East and north Africa	
382	Jordan	2019	STEPS	20-64	20-64	20-64	20-64	2,726	1,694	2,719	1,692	Central Asia, Middle East and north Africa	
383	Jordan	2016-2017	National Cardiovascular Diseases and Diabetes Study (NCDDS)	20-64	20-64	20-64	20-64	2,439	984	2,415	973	Central Asia, Middle East and north Africa	
384	Kazakhstan	2015	Almaty STEPS	20-64	20-64	20-64	20-64	1,022	332	1,018	330	Central Asia, Middle East and north Africa	
385	Kazakhstan	2015	Shymkent STEPS	20-64	20-64	20-64	20-64	630	306	577	290	Central Asia, Middle East and north Africa	
386	Kazakhstan	2019	A health status assessment of a population of Karaganda urban region	20-64	20-64	20-64	20-64	620	308	615	307	Central Asia, Middle East and north Africa	
387	Kazakhstan	2021	Prevalence of NCD Risk Factors in Kazakhstan	20-64	20-64	20-64	20-64	724	715	721	714	Central Asia, Middle East and north Africa	10
388	Kazakhstan	2011-2012	Household Health Survey	20-64	20-64	20-64	20-64	5,114	4,110	4,630	3,653	Central Asia, Middle East and north Africa	
389	Kazakhstan	2015-2016	Aktobe STEPS	20-64	20-64	20-64	20-64	1,045	296	1,032	293	Central Asia, Middle East and north Africa	
390	Kenya	2015	STEPS	20-64	20-64	20-64	20-64	2,206	1,568	2,203	1,567	Sub-Saharan Africa	
391	Kiribati	2004	STEPS	20-64	20-64	20-64	20-64	843	681	839	677	Oceania	
392	Kiribati	2015-2016	STEPS	20-64	20-64	20-64	20-64	647	524	635	515	Oceania	
393	Kuwait	2006	STEPS	20-64	20-64	20-64	20-64	1,294	915	1,293	915	Central Asia, Middle East and north Africa	
394	Kuwait	2014	STEPS	20-64	20-64	20-64	20-64	1,960	1,174	1,947	1,172	Central Asia, Middle East and north Africa	
395	Kuwait	2008-2010	Gulf Cooperation Council World Health Survey	20-64	20-64	20-64	20-64	1,431	1,234	1,390	1,199	Central Asia, Middle East and north Africa	
396	Kuwait	2011-2014	Kuwait Diabetes Epidemiology Program	20-64	20-64	20-64	20-64	2,082	2,671	2,077	2,651	Central Asia, Middle East and north Africa	
397	Kyrgyzstan	1993	Kyrgyzstan Multipurpose Poverty Survey	20-64	20-64			1,956	1,781			Central Asia, Middle East and north Africa	
398	Kyrgyzstan	2013	STEPS	25-64	25-64	25-64	25-64	1,598	939	1,597	939	Central Asia, Middle East and north Africa	
399	Lao PDR	2008	STEPS	25-64	25-64	25-64	25-64	1,013	618	1,006	613	East and southeast Asia and the Pacific	
400	Lao PDR	2013	STEPS	20-64	20-64	20-64	20-64	1,408	943	1,405	943	East and southeast Asia and the Pacific	
401	Lebanon	1997	Obesity in Lebanon: National Survey	20-64	20-64			645	421			Central Asia, Middle East and north Africa	
402	Lebanon	2017	STEPS	20-64	20-64	20-64	20-64	873	607	873	606	Central Asia, Middle East and north Africa	
403	Lebanon	2008-2009	STEPS	20-64	20-64	20-64	20-64	1,279	1,076	1,248	1,029	Central Asia, Middle East and north Africa	
404	Lesotho	2012	STEPS	25-64	25-64	25-64	25-64	1,412	705	1,405	704	Sub-Saharan Africa	
405	Liberia	2011	STEPS	25-64	25-64	25-64	25-64	1,062	749	1,059	747	Sub-Saharan Africa	
406	Liberia	2022	STEPS	20-64	20-64	20-64	20-64	1,891	1,225	1,888	1,223	Sub-Saharan Africa	
407	Libya	2009	STEPS	25-64	25-64	25-64	25-64	1,478	1,608	1,469	1,601	Central Asia, Middle East and north Africa	
408	Lithuania	2001-2002	MONICA4	35-64	35-64	35-64	35-64	776	624	704	533	Central and eastern Europe	
409	Lithuania	2006-2008	Health, Alcohol and Psychosocial Factors In Eastern Europe	45-64	45-64	45-64	45-64	2,533	2,060	2,521	2,042	Central and eastern Europe	
410	Luxembourg	2013-2015	European Health Examination Survey in Luxembourg	25-64	25-64	25-64	25-64	784	721	779	720	High-income western	
411	Luxembourg	2016-2018	Observation of cardiovascular risk factors in Luxembourg (ORISCAV-LUX2)	25-64	25-64	25-64	25-64	657	579	657	576	High-income western	
412	Madagascar	2005	STEPS	25-64	25-64	25-64	25-64	2,317	2,392	2,251	2,315	Sub-Saharan Africa	
413	Malawi	2009	STEPS	25-64	25-64	25-64	25-64	2,986	1,586	2,343	1,126	Sub-Saharan Africa	

	Country	Years	Survey/Study name/Citation	Age range used in anthropometric analyses		Age range used in hypertension analyses		Individuals used in anthropometric analyses		Individuals used in hypertension analyses		Region	Notes
				Women	Men	Women	Men	Women	Men	Women	Men		
414	Malawi	2017	STEPS	20-64	20-64	20-64	20-64	2,258	1,322	2,258	1,322	Sub-Saharan Africa	
415	Malawi	2013-2017	NCD Survey Malawi Epidemiology and Intervention Research Unit	20-64	20-64	20-64	20-64	15,235	9,720	15,228	9,719	Sub-Saharan Africa	
416	Malaysia	2004	Rampal et al., Public Health 122(1):11-8, 2008	20-64	20-64	20-64	20-64	7,419	5,250	7,338	5,174	East and southeast Asia and the Pacific	
417	Malaysia	2006	National Health and Morbidity Survey (NHMS)	20-64	20-64	20-64	20-64	15,298	12,775	15,197	12,688	East and southeast Asia and the Pacific	
418	Malaysia	2008	Metabolic Syndrome Study in Malaysia	20-64	20-64			2,407	1,241			East and southeast Asia and the Pacific	
419	Malaysia	2011	National Health and Morbidity Survey (NHMS)	20-64	20-64	20-64	20-64	7,338	6,717	7,278	6,638	East and southeast Asia and the Pacific	
420	Malaysia	2014	Malaysian Adult Nutrition Survey	20-59	20-59			1,476	1,266			East and southeast Asia and the Pacific	
421	Malaysia	2015	National Health and Morbidity Survey (NHMS)	20-64	20-64	20-64	20-64	8,016	7,417	7,959	7,335	East and southeast Asia and the Pacific	
422	Malaysia	2019	National Health and Morbidity Survey (NHMS)	20-64	20-64	20-64	20-64	4,308	3,687	4,260	3,636	East and southeast Asia and the Pacific	
423	Maldives	2011	STEPS	20-64	20-64	20-64	20-64	921	517	899	510	East and southeast Asia and the Pacific	
424	Maldives	2020-2021	STEPS	20-64	20-64	20-64	20-64	1,643	651	1,488	603	East and southeast Asia and the Pacific	
425	Mali	2007	STEPS	20-64	20-64	20-64	20-64	1,169	829	1,162	816	Sub-Saharan Africa	
426	Mali	2013	STEPS	20-64	20-64	20-64	20-64	744	421	566	312	Sub-Saharan Africa	
427	Malta	2014-2016	SAHHTEK - The University of Malta Health and Wellbeing Study	20-64	20-64	20-64	20-64	877	696	831	658	High-income western	
428	Marshall Islands	2002	STEPS	20-64	20-64	20-64	20-64	967	603	946	595	Oceania	
429	Mauritania	2006	STEPS	20-64	20-64			1,128	989			Sub-Saharan Africa	
430	Mauritius	1992	Mauritius Noncommunicable Disease Survey	25-64	25-64	25-64	25-64	3,160	2,786	3,119	2,749	Sub-Saharan Africa	
431	Mauritius	1992	Rodrigues, Mauritius (1992)	25-64	25-64	25-64	25-64	768	735	764	733	Sub-Saharan Africa	
432	Mauritius	1998	Mauritius Noncommunicable Disease Survey	25-64	25-64	25-64	25-64	2,866	2,274	2,861	2,268	Sub-Saharan Africa	
433	Mauritius	1999	Rodrigues, Mauritius (1999)	20-64	20-64	20-64	20-64	1,160	862	1,156	859	Sub-Saharan Africa	
434	Mauritius	2009	Mauritius Noncommunicable Disease Survey	20-64	20-64	20-64	20-64	3,025	2,606	3,000	2,587	Sub-Saharan Africa	
435	Mauritius	2015	Mauritius Noncommunicable Disease Survey	20-64	20-64	20-64	20-64	1,678	1,403	1,676	1,397	Sub-Saharan Africa	
436	Mexico	2001	The Mexican Health and Aging Study	50-64	50-64			806	639			Latin America and the Caribbean	
437	Mexico	2005	Encuesta Nacional Sobre Niveles de vida de los Hogares	20-64	20-64	20-64	20-64	7,370	5,925	7,241	5,844	Latin America and the Caribbean	
438	Mexico	2006	Encuesta Nacional de Salud y Nutrición	20-64	20-64	20-64	20-64	17,161	10,873	16,792	10,793	Latin America and the Caribbean	
439	Mexico	2006	PREVENIMSS National Coverage Surveys	20-64	20-64			9,345	7,147			Latin America and the Caribbean	
440	Mexico	2016	Encuesta Nacional de Salud y Nutrición	20-64	20-64	20-64	20-64	4,405	2,286	4,277	2,251	Latin America and the Caribbean	
441	Mexico	2021	Encuesta Nacional de Salud y Nutrición	20-64	20-64	20-64	20-64	4,457	2,803	4,086	2,289	Latin America and the Caribbean	
442	Mexico	2022	Encuesta Nacional de Salud y Nutrición	20-64	20-64	20-64	20-64	4,105	2,543	4,083	2,529	Latin America and the Caribbean	
443	Mexico	1999-2000	The Survey on Health, Well-Being, and Aging in Latin America and the Caribbean (SABE)	60-64	60-64	60-64	60-64	192	134	192	134	Latin America and the Caribbean	1
444	Mexico	2009-2010	WHO Study on global AGEing and adult health (SAGE)	50-64	50-64	50-64	50-64	527	324	519	316	Latin America and the Caribbean	
445	Mexico	2009-2012	Encuesta Nacional Sobre Niveles de vida de los Hogares	20-64	20-64	20-64	20-64	8,433	6,781	8,108	6,184	Latin America and the Caribbean	
446	Mexico	2011-2012	Encuesta Nacional de Salud y Nutrición	20-64	20-64	20-64	20-64	18,117	13,525	5,538	3,748	Latin America and the Caribbean	
447	Mexico	2018-2019	Encuesta Nacional de Salud y Nutrición	20-64	20-64	20-64	20-64	7,069	5,511	6,137	4,726	Latin America and the Caribbean	
448	Moldova	2013	STEPS	20-64	20-64	20-64	20-64	2,416	1,470	2,412	1,465	Central and eastern Europe	
449	Moldova	2021	STEPS	20-64	20-64	20-64	20-64	1,840	1,450	1,840	1,449	Central and eastern Europe	
450	Mongolia	2005	STEPS	20-64	20-64	20-64	20-64	1,517	1,436	1,511	1,429	Central Asia, Middle East and north Africa	
451	Mongolia	2009	STEPS	20-64	20-64	20-64	20-64	2,887	2,015	2,866	2,009	Central Asia, Middle East and north Africa	
452	Mongolia	2013	STEPS	20-64	20-64	20-64	20-64	2,682	2,186	2,671	2,183	Central Asia, Middle East and north Africa	
453	Mongolia	2019	STEPS	20-64	20-64	20-64	20-64	3,160	2,629	3,157	2,625	Central Asia, Middle East and north Africa	
454	Morocco	2017	STEPS	20-64	20-64	20-64	20-64	2,885	1,487	2,883	1,487	Central Asia, Middle East and north Africa	
455	Mozambique	2005	STEPS	25-64	25-64	25-64	25-64	1,652	1,258	1,645	1,256	Sub-Saharan Africa	
456	Mozambique	2014-2015	STEPS	20-64	20-64	20-64	20-64	1,447	968	1,443	967	Sub-Saharan Africa	
457	Myanmar	2009	STEPS	20-64	20-64	20-64	20-64	4,087	2,589	4,073	2,578	East and southeast Asia and the Pacific	
458	Myanmar	2014	STEPS	25-64	25-64	25-64	25-64	5,411	2,925	5,405	2,921	East and southeast Asia and the Pacific	
459	Myanmar	2003-2004	STEPS	25-64	25-64	25-64	25-64	2,103	1,628	2,086	1,617	East and southeast Asia and the Pacific	
460	Myanmar	2013-2014	STEPS, Yangon	25-64	25-64	25-64	25-64	669	646	669	646	East and southeast Asia and the Pacific	
461	Namibia	2005	STEPS	25-64	25-64	25-64	25-64	1,749	1,363	1,699	1,305	Sub-Saharan Africa	
462	Namibia	2009	Okambilimbili Survey	20-64	20-64			819	674			Sub-Saharan Africa	
463	Nauru	2004	STEPS	20-64	20-64	20-64	20-64	1,027	954	1,023	951	Oceania	
464	Nauru	2006	STEPS	20-64	20-64	20-64	20-64	218	226	218	226	Oceania	
465	Nauru	2015	STEPS	20-64	20-64	20-64	20-64	485	447	482	443	Oceania	
466	Nepal	2005	STEPS	20-64	20-64	20-64	20-64	3,598	3,226	3,508	3,162	South Asia	
467	Nepal	2015	Community based intervention for prevention and control of non-communicable diseases risk factors (CIPCON) baseline survey, Dhankuta	20-64	20-64	20-64	20-64	709	468	709	468	South Asia	
468	Nepal	2015	Community based intervention for prevention and control of non-communicable diseases risk factors (CIPCON) baseline survey, Ilam	20-64	20-64	20-64	20-64	660	495	660	495	South Asia	
469	Nepal	2019	STEPS	20-64	20-64	20-64	20-64	3,126	1,704	3,126	1,704	South Asia	
470	Nepal	2006-2011	Early detection and management of Kidney disease, Hypertension, Diabetes and Cardiovascular disease (KHDC Nepal), Tarahara	20-64	20-64	20-64	20-64	2,083	994	2,083	994	South Asia	
471	Nepal	2006-2011	Early detection and management of Kidney disease, Hypertension, Diabetes and Cardiovascular disease (KHDC Nepal), Damak	20-64	20-64	20-64	20-64	1,424	964	1,424	964	South Asia	
472	Nepal	2006-2011	Early detection and management of Kidney disease, Hypertension, Diabetes and Cardiovascular disease (KHDC Nepal), Dharan	20-64	20-64	20-64	20-64	5,291	3,395	5,286	3,394	South Asia	
473	Nepal	2007-2008	STEPS	20-64	20-64	20-64	20-64	2,090	1,620	2,087	1,614	South Asia	
474	Nepal	2012-2013	STEPS	20-64	20-64	20-64	20-64	2,509	1,132	2,509	1,132	South Asia	
475	Nepal	2016-2018	The Population Based Prevalence of Selected Non-Communicable Diseases In Nepal	20-64	20-64	20-64	20-64	6,480	3,784	6,480	3,784	South Asia	
476	Netherlands	1992-1993	The Longitudinal Aging Study Amsterdam (LASA)	55-64	55-64			429	406			High-income western	11
477	Netherlands	1993-1997	EPIC Bilthoven	20-59	20-59	20-59	20-59	11,994	9,923	11,373	9,590	High-income western	
478	Netherlands	1993-1997	EPIC Utrecht	49-64		49-64		14,599		14,188		High-income western	

	Country	Years	Survey/Study name/Citation	Age range used in anthropometric analyses		Age range used in hypertension analyses		Individuals used in anthropometric analyses		Individuals used in hypertension analyses		Region	Notes
				Women	Men	Women	Men	Women	Men	Women	Men		
479	Netherlands	1998-2001	Regenboog Project	20-64	20-64	20-64	20-64	2,065	2,037	2,037	2,011	High-income western	
480	Netherlands	2001-2003	Surinamese in the Netherlands: Study on Ethnicity and Health (SUNSET)	35-60	35-60	35-60	35-60	257	251	257	251	High-income western	
481	Netherlands	2002-2003	The Longitudinal Aging Study Amsterdam (LASA)	54-64	54-64	54-64	54-64	479	430	474	426	High-income western	11
482	Netherlands	2003-2007	Doetinchem Cohort Study	36-64	36-64	36-64	36-64	1,924	1,690	1,922	1,690	High-income western	
483	Netherlands	2012-2013	The Longitudinal Aging Study Amsterdam (LASA)	55-64	55-64	55-64	55-64	437	407	431	406	High-income western	11
484	New Zealand	2006-2007	New Zealand Health Survey	20-64	20-64			4,897	3,784			High-income western	
485	New Zealand	2008-2009	New Zealand Adult Nutrition Survey	20-64	20-64	20-64	20-64	1,376	1,082	1,328	1,056	High-income western	
486	New Zealand	2011-2012	New Zealand Health Survey	20-64	20-64			4,388	3,249			High-income western	
487	New Zealand	2012-2013	New Zealand Health Survey	20-64	20-64	20-64	20-64	4,769	3,593	4,568	3,415	High-income western	
488	New Zealand	2013-2014	New Zealand Health Survey	20-64	20-64	20-64	20-64	5,047	4,084	4,969	3,997	High-income western	
489	New Zealand	2014-2015	New Zealand Health Survey	20-64	20-64	20-64	20-64	5,101	4,074	5,003	3,989	High-income western	
490	New Zealand	2015-2016	New Zealand Health Survey	20-64	20-64	20-64	20-64	4,950	4,068	4,867	3,994	High-income western	
491	New Zealand	2016-2017	New Zealand Health Survey	20-64	20-64	20-64	20-64	4,928	3,865	4,805	3,786	High-income western	
492	New Zealand	2017-2018	New Zealand Health Survey	20-64	20-64			5,003	3,969			High-income western	
493	New Zealand	2018-2019	New Zealand Health Survey	20-64	20-64	20-64	20-64	4,995	3,765	4,866	3,659	High-income western	
494	New Zealand	2019-2020	New Zealand Health Survey	20-64	20-64	20-64	20-64	3,435	2,510	3,363	2,444	High-income western	
495	New Zealand	2020-2021	New Zealand Health Survey	20-64	20-64	20-64	20-64	2,879	2,208	2,792	2,133	High-income western	
496	Nicaragua	2003-2004	CAMDI	20-64	20-64	20-64	20-64	851	687	851	687	Latin America and the Caribbean	
497	Niger	2007	STEPS	20-64	20-64	20-64	20-64	1,065	1,289	1,063	1,265	Sub-Saharan Africa	
498	Niger	2021	STEPS	20-64	20-64	20-64	20-64	2,635	1,962	2,635	1,962	Sub-Saharan Africa	
499	Nigeria	2007	Ibadan Study of Ageing	60-64	60-64	60-64	60-64	117	9	112	9	Sub-Saharan Africa	
500	Nigeria	2007	Southeast Nigeria kidney disease study	25-64	25-64	25-64	25-64	442	168	439	168	Sub-Saharan Africa	
501	Nigeria	2009	Community Health Plan - Kwara Central Survey	20-64	20-64			1,067	891			Sub-Saharan Africa	
502	Nigeria	2011	Community Health Plan - Kwara Central Survey	20-64	20-64	20-64	20-64	402	294	401	294	Sub-Saharan Africa	
503	Nigeria	2013	Community Health Plan - Kwara Central Survey	20-64	20-64	20-64	20-64	346	264	345	264	Sub-Saharan Africa	
504	Nigeria	2018	Hypertension Prevalence, Awareness, Treatment and Control in Rural Area, Nigeria	20-64	20-64	20-64	20-64	149	145	149	143	Sub-Saharan Africa	
505	Nigeria	1999-2009	Prostate cancer dietary risk factors study		35-64				363			Sub-Saharan Africa	
506	Nigeria	2009-2011	Anthropometric indices in Calabar	20-64	20-64			309	369			Sub-Saharan Africa	
507	Nigeria	2017-2019	Removing the Mask on Hypertension (REMAH)	20-64	20-64	20-64	20-64	1,940	1,515	1,911	1,499	Sub-Saharan Africa	
508	Niue	2011	STEPS	20-64	20-64	20-64	20-64	345	293	342	290	Oceania	
509	Norway	1994-1995	The Tromsø Study: Tromsø 4	25-64	25-64	25-64	25-64	2,045	2,206	2,036	2,199	High-income western	
510	Norway	1995-1997	HUNT2 study	20-64	20-64	20-64	20-64	25,516	23,282	25,483	23,255	High-income western	
511	Norway	1997-1998	The Hordaland Health Study (HUSK) 1950-1951 birth cohort	46-49	46-49	46-49	46-49	2,058	1,656	2,042	1,642	High-income western	
512	Norway	1997-1998	The Hordaland Health Study (HUSK) 1953-1957 birth cohort	39-46	39-46	39-46	39-46	9,862	8,517	9,753	8,437	High-income western	
513	Oman	1991	The 1991 National Diabetes Survey of Oman	20-64	20-64			2,757	1,906			Central Asia, Middle East and north Africa	
514	Oman	2001	Nizwa Healthy Lifestyle Project	20-64	20-64	20-64	20-64	684	650	675	608	Central Asia, Middle East and north Africa	
515	Oman	2008	Gulf Cooperation Council World Health Survey	20-64	20-64	20-64	20-64	1,668	1,845	1,649	1,828	Central Asia, Middle East and north Africa	
516	Oman	2017	STEPS	20-64	20-64	20-64	20-64	2,629	3,024	2,627	3,024	Central Asia, Middle East and north Africa	
517	Pakistan	2005	STEPS	25-64	25-64	25-64	25-64	1,039	752	833	538	South Asia	
518	Pakistan	2014	STEPS	20-64	20-64	20-64	20-64	3,188	2,507	3,174	2,498	South Asia	
519	Pakistan	1990-1994	National Health Survey of Pakistan 1990-1994	20-64	20-64			3,293	3,008			South Asia	
520	Pakistan	2016-2017	National Diabetes Survey of Pakistan	20-64	20-64			3,365	2,535			South Asia	
521	Palau	2011-2013	STEPS	25-64	25-64	25-64	25-64	1,115	1,019	1,096	1,003	Oceania	
522	Panama	2019	Encuesta Nacional de Salud de Panama (ENSPA)	20-64	20-64	20-64	20-64	8,516	2,855	7,910	2,620	Latin America and the Caribbean	
523	Panama	2010-2011	Prevalencia de factores de riesgo asociados a enfermedad cardiovascular 2010-2011	20-64	20-64	20-64	20-64	2,044	804	2,035	803	Latin America and the Caribbean	
524	Papua New Guinea	1991	Dowse et al., Med J Aust 160:767-74, 1994	25-64	25-64	25-64	25-64	896	731	733	590	Oceania	
525	Paraguay	2011	Primera Encuesta Nacional de Factores de Riesgo de Enfermedades No Transmisibles en la Poblacion General	20-64	20-64	20-64	20-64	1,234	716	1,226	709	Latin America and the Caribbean	
526	Peru	2003	Factores de Riesgo de Enfermedades No Transmisibles	20-64	20-64	20-64	20-64	459	277	454	276	Latin America and the Caribbean	
527	Peru	2004	Factores de Riesgo de Enfermedades No Transmisibles	20-64	20-64	20-64	20-64	366	163	363	161	Latin America and the Caribbean	
528	Peru	2005	Factores de Riesgo de Enfermedades No Transmisibles	20-64	20-64	20-64	20-64	477	168	474	167	Latin America and the Caribbean	
529	Peru	2006	Factores de Riesgo de Enfermedades No Transmisibles	20-64	20-64	20-64	20-64	908	514	905	513	Latin America and the Caribbean	
530	Peru	2013	Clinical functional and sociofamilial profiles of the elderly from a community in a district of Lima, Peru	60-64	60-64			93	42			Latin America and the Caribbean	
531	Peru	2004-2005	Encuesta Nacional de Indicadores Nutricionales, Bioquímicos, Socioeconómicos y Culturales Relacionados con las Enfermedades Crónicas Degenerativas	20-64	20-64	20-64	20-64	1,930	1,807	1,899	1,790	Latin America and the Caribbean	
532	Peru	2007-2008	PERU MIGRANT Study	30-64	30-64	30-64	30-64	471	417	471	417	Latin America and the Caribbean	
533	Peru	2009-2011	Monitoreo de Indicadores Nutricionales en la ENAHO 2009-2010	20-64	20-64			17,191	14,035			Latin America and the Caribbean	
534	Peru	2009-2012	CRONICAS Cohort Study	35-64	35-64	35-64	35-64	1,247	1,157	1,247	1,156	Latin America and the Caribbean	
535	Peru	2011-2012	Monitoreo de Indicadores Nutricionales en la ENAHO 2011	20-64	20-64			4,640	3,760			Latin America and the Caribbean	
536	Peru	2014-2015	Latin American Study of Nutrition and Health (ELANS)	20-64	20-64			558	510			Latin America and the Caribbean	
537	Peru	2016-2017	Screening of T2DM	30-64	30-64	30-64	30-64	748	751	748	751	Latin America and the Caribbean	
538	Peru	2017-2018	Vigilancia Alimentario Nutricional por Etapas de Vida (VIANEV) 2017-2018	20-59	20-59	20-59	20-59	570	448	560	437	Latin America and the Caribbean	
539	Philippines	2003	6th National Nutrition Survey	20-64	20-64	20-64	20-64	1,532	1,564	1,522	1,559	East and southeast Asia and the Pacific	
540	Philippines	2005	Cebu Longitudinal Health and Nutrition Survey 2005 Child Follow-up	20-22	20-22			829	1,004			East and southeast Asia and the Pacific	
541	Philippines	2008	7th National Nutrition Survey	20-64	20-64	20-64	20-64	2,923	2,903	2,837	2,815	East and southeast Asia and the Pacific	
542	Philippines	2011	2011 Updating of Nutritional Status of Filipino Children and Other Population Groups	20-64	20-64			35,181	30,906			East and southeast Asia and the Pacific	
543	Philippines	2015	2015 Updating of Nutritional Status of Filipino Children and Other Population Groups	20-64	20-64	20-64	20-64	39,810	36,091	38,789	34,987	East and southeast Asia and the Pacific	
544	Philippines	1994-1995	Cebu Longitudinal Health and Nutrition Survey 1994-1995 Mother Follow-up	20-59				2,448				East and southeast Asia and the Pacific	
545	Philippines	2013-2014	8th National Nutrition Survey	20-64	20-64	20-64	20-64	33,668	29,398	8,386	8,028	East and southeast Asia and the Pacific	
546	Poland	2000	The health status, risk factors of chronic diseases and health behaviors of residents of Torun (CINDI Torun 2000)	20-64	20-64	20-64	20-64	935	843	927	840	Central and eastern Europe	

	Country	Years	Survey/Study name/Citation	Age range used in anthropometric analyses		Age range used in hypertension analyses		Individuals used in anthropometric analyses		Individuals used in hypertension analyses		Region	Notes
				Women	Men	Women	Men	Women	Men	Women	Men		
547	Poland	2002	NATPOL	20-64	20-64	20-64	20-64	1,005	811	1,004	808	Central and eastern Europe	
548	Poland	2003	The European Male Ageing Study		40-64		40-64		250		241	Central and eastern Europe	
549	Poland	2004	LIPIDOGRAM2004 Study - National epidemiological study of lipid disorders and selected risk factors of cardiovascular disease in primary health care in Poland	30-64	30-64			7,918	5,499			Central and eastern Europe	
550	Poland	2006	The health, risk factors for chronic diseases, attitudes and behaviors of health residents of Torun (CINDI Torun 2006)	20-64	20-64	20-64	20-64	1,066	714	1,065	713	Central and eastern Europe	
551	Poland	2006	LIPIDOGRAM2006 Study - National epidemiological study of lipid disorders and selected risk factors of cardiovascular disease in primary health care in Poland	32-64	32-64			7,832	4,921			Central and eastern Europe	
552	Poland	2011	NATPOL	20-64	20-64	20-64	20-64	985	972	985	972	Central and eastern Europe	
553	Poland	2018	Mogielica Human Ecology Study Site	20-64	20-64	20-64	20-64	71	25	71	24	Central and eastern Europe	
554	Poland	1995-1996	Polish Program CINDI (CINDI Lodz 1995)	20-64	20-64	20-64	20-64	1,140	725	1,132	710	Central and eastern Europe	
555	Poland	2000-2001	Household Food Consumption and Anthropometric Survey	20-64	20-64			1,251	989			Central and eastern Europe	
556	Poland	2001-2002	The health status, risk factors of chronic diseases and health behaviors of residents of Lodz (CINDI Lodz 2001)	20-64	20-64	20-64	20-64	800	936	797	929	Central and eastern Europe	
557	Poland	2002-2005	Health, Alcohol and Psychosocial Factors In Eastern Europe	45-64	45-64	45-64	45-64	3,857	3,542	3,815	3,502	Central and eastern Europe	
558	Poland	2003-2005	National Multicenter Health Survey in Poland. Project WOBASZ	20-64	20-64	20-64	20-64	6,027	5,426	5,986	5,389	Central and eastern Europe	
559	Poland	2003-2006	Mogielica Human Ecology Study Site	20-64	20-64	20-64	20-64	271	98	266	88	Central and eastern Europe	
560	Poland	2007-2010	Mogielica Human Ecology Study Site	20-64	20-64	20-64	20-64	223	91	217	82	Central and eastern Europe	
561	Poland	2007-2011	Medical, psychological and socioeconomic aspects of aging in Poland	55-64	55-64	55-64	55-64	379	325	377	311	Central and eastern Europe	
562	Poland	2011-2014	Mogielica Human Ecology Study Site	20-64	20-64	20-64	20-64	262	84	258	81	Central and eastern Europe	
563	Poland	2013-2014	National Multicenter Health Survey in Poland. Project WOBASZ II	20-64	20-64	20-64	20-64	2,507	2,114	2,494	2,108	Central and eastern Europe	
564	Poland	2015-2016	LIPIDOGRAM2015 & LIPIDOGEN2015 Study - National epidemiological study of lipid disorders and selected risk factors of cardiovascular disease in primary health care in Poland	20-64	20-64	20-64	20-64	6,309	3,574	6,309	3,574	Central and eastern Europe	
565	Portugal	1999-2003	EPIPorto study	20-64	20-64	20-64	20-64	1,113	664	815	512	High-income western	
566	Puerto Rico	2002-2003	Puerto Rican Elderly: Health Conditions	60-64	60-64			716	454			Latin America and the Caribbean	12
567	Puerto Rico	2010-2013	HPV Infection in a Population-Based Sample of Puerto Rican Women	20-64				538				Latin America and the Caribbean	
568	Qatar	2012	STEPS	20-64	20-64	20-64	20-64	1,222	942	1,218	939	Central Asia, Middle East and north Africa	
569	Romania	2011-2012	Study for the Evaluation of Prevalence of Hypertension and cArdiovascular Risk among the Adult Population of Romania - SEPHAR II	20-64	20-64	20-64	20-64	765	792	765	792	Central and eastern Europe	
570	Romania	2012-2014	PREvalence of DiAbetes mellitus, prediabetes, overweight, Obesity, dyslipidemia, hyperuricemia and chronic kidney disease in Romania (PREDATORR)	20-64	20-64	20-64	20-64	996	898	995	895	Central and eastern Europe	
571	Romania	2015-2016	Study for the Evaluation of Prevalence of Hypertension and cArdiovascular Risk among the Adult Population of Romania - SEPHAR III	20-64	20-64	20-64	20-64	777	688	777	688	Central and eastern Europe	
572	Russian Federation	1993	Russia Longitudinal Monitoring Survey- Higher School of Economics Round III	20-64	20-64			4,809	3,793			Central and eastern Europe	
573	Russian Federation	1994	Russia Longitudinal Monitoring Survey- Higher School of Economics Round V	20-64	20-64			3,583	3,017			Central and eastern Europe	
574	Russian Federation	1995	Russia Longitudinal Monitoring Survey- Higher School of Economics Round VI	20-64	20-64			3,319	2,778			Central and eastern Europe	
575	Russian Federation	1996	Russia Longitudinal Monitoring Survey- Higher School of Economics Round VII	20-64	20-64			3,353	2,729			Central and eastern Europe	
576	Russian Federation	2000	Russia Longitudinal Monitoring Survey- Higher School of Economics Round IX	20-64	20-64			3,599	2,905			Central and eastern Europe	
577	Russian Federation	2001	Russia Longitudinal Monitoring Survey- Higher School of Economics Round X	20-64	20-64			4,036	3,183			Central and eastern Europe	
578	Russian Federation	2002	Russia Longitudinal Monitoring Survey- Higher School of Economics Round XI	20-64	20-64			4,128	3,340			Central and eastern Europe	
579	Russian Federation	2003	Russia Longitudinal Monitoring Survey- Higher School of Economics Round XII	20-64	20-64			4,179	3,379			Central and eastern Europe	
580	Russian Federation	2004	Russia Longitudinal Monitoring Survey- Higher School of Economics Round XIII	20-64	20-64			4,197	3,390			Central and eastern Europe	
581	Russian Federation	2005	Russia Longitudinal Monitoring Survey- Higher School of Economics Round XIV	20-64	20-64			4,073	3,282			Central and eastern Europe	
582	Russian Federation	1992-1993	Russia Longitudinal Monitoring Survey- Higher School of Economics Round II	20-64	20-64			4,507	3,659			Central and eastern Europe	
583	Russian Federation	1993-1994	Russia Longitudinal Monitoring Survey- Higher School of Economics Round IV	20-64	20-64			4,241	3,397			Central and eastern Europe	
584	Russian Federation	1998-1999	Russia Longitudinal Monitoring Survey- Higher School of Economics Round VIII	20-64	20-64			3,446	2,829			Central and eastern Europe	
585	Russian Federation	2002-2005	Health, Alcohol and Psychosocial Factors In Eastern Europe	45-64	45-64	45-64	45-64	3,908	3,230	3,907	3,229	Central and eastern Europe	
586	Russian Federation	2007-2010	WHO Study on global AGEing and adult health (SAGE)	50-64	50-64	50-64	50-64	1,001	574	992	571	Central and eastern Europe	
587	Russian Federation	2015-2017	Ural Eye and Medical Study (UEMS)	40-64	40-64	40-64	40-64	2,224	1,894	2,223	1,893	Central and eastern Europe	
588	Rwanda	2012	STEPS	20-64	20-64	20-64	20-64	3,809	2,364	3,808	2,363	Sub-Saharan Africa	
589	Rwanda	2021-2022	STEPS	20-64	20-64	20-64	20-64	3,116	1,929	3,115	1,928	Sub-Saharan Africa	
590	Saint Kitts and Nevis	2007	STEPS	25-64	25-64	25-64	25-64	829	494	731	408	Latin America and the Caribbean	
591	Saint Lucia	2012	STEPS	25-64	25-64	25-64	25-64	1,045	631	1,044	631	Latin America and the Caribbean	
592	Saint Lucia	2019-2020	STEPS	20-64	20-64	20-64	20-64	1,259	995	1,259	994	Latin America and the Caribbean	
593	Saint Vincent and the Grenadines	2013-2014	STEPS	20-64	20-64	20-64	20-64	1,734	1,370	1,724	1,365	Latin America and the Caribbean	
594	Samoa	1991	Non-Communicable Disease Risk Factor (NCDRF)	25-64	25-64	25-64	25-64	834	694	828	684	Oceania	
595	Samoa	1995	McGarvey, Pac Health Dialog 8(1):157-62, 2001	29-64	29-64	29-64	29-64	134	134	134	134	Oceania	
596	Samoa	2002	STEPS	25-64	25-64	25-64	25-64	1,322	1,169	1,302	1,126	Oceania	
597	Samoa	2010	Samoan Genome-Wide Association Study	24-64	24-64	24-64	24-64	2,052	1,394	2,031	1,382	Oceania	13
598	Samoa	2013	STEPS	20-64	20-64	20-64	20-64	851	563	850	563	Oceania	
599	Sao Tome and Principe	2009	STEPS	25-64	25-64	25-64	25-64	1,155	861	1,145	855	Sub-Saharan Africa	
600	Sao Tome and Principe	2019	STEPS	20-64	20-64	20-64	20-64	1,205	871	1,204	871	Sub-Saharan Africa	
601	Saudi Arabia	2007	Gulf Cooperation Council World Health Survey	20-64	20-64	20-64	20-64	2,919	4,048	2,907	4,041	Central Asia, Middle East and north Africa	
602	Senegal	2003	Perceptions of healthy and desirable body size in urban Senegalese women	20-50				300				Sub-Saharan Africa	
603	Senegal	2015	Les maladies chroniques au Sénégal: Une écologie de la santé comparative entre Dakar et Widou Thiengoly	20-64	20-64	20-64	20-64	731	698	731	698	Sub-Saharan Africa	
604	Senegal	2015	STEPS	20-64	20-64	20-64	20-64	3,133	1,761	3,114	1,748	Sub-Saharan Africa	
605	Senegal	2010-2012	Biocultural determinants of overweight and obesity in the context of nutrition transition in Senegal: a holistic anthropological approach	20-64	20-64	20-64	20-64	252	240	195	183	Sub-Saharan Africa	
606	Serbia	2013-2014	Stay Fit for Lifelong Health; the Prevalence of Lifestyle Health Conditions in Serbian Population	20-64	20-64			297	1,327			Central and eastern Europe	
607	Seychelles	1994	Seychelles Heart Survey II	25-64	25-64	25-64	25-64	559	497	559	496	Sub-Saharan Africa	
608	Seychelles	2004	Seychelles Heart Survey III	25-64	25-64	25-64	25-64	686	567	686	567	Sub-Saharan Africa	

	Country	Years	Survey/Study name/Citation	Age range used in anthropometric analyses		Age range used in hypertension analyses		Individuals used in anthropometric analyses		Individuals used in hypertension analyses		Region	Notes
				Women	Men	Women	Men	Women	Men	Women	Men		
609	Seychelles	2013-2014	Seychelles Heart Survey IV	25-64	25-64	25-64	25-64	699	531	699	531	Sub-Saharan Africa	
610	Sierra Leone	2009	STEPS	25-64	25-64	25-64	25-64	2,085	1,910	2,068	1,904	Sub-Saharan Africa	
611	Singapore	2003-2005	Singapore Longitudinal Ageing Study - Cohort 1; SLAS-1	55-64	55-64	55-64	55-64	924	477	924	477	East and southeast Asia and the Pacific	
612	Singapore	2008-2013	Singapore Longitudinal Ageing Study - Cohort 2; SLAS-2	55-64	55-64	55-64	55-64	925	457	923	456	East and southeast Asia and the Pacific	
613	Singapore	2012-2013	Singapore Health Study	20-64	20-64			897	839			East and southeast Asia and the Pacific	14
614	Singapore	2014-2015	Singapore Health 2	20-64	20-64			855	666			East and southeast Asia and the Pacific	14
615	Slovakia	1993	Countrywide Integrated Noncommunicable Diseases Intervention Programme	20-64	20-64			1,155	671			Central and eastern Europe	
616	Slovakia	1998	Countrywide Integrated Noncommunicable Diseases Intervention Programme	20-64	20-64			997	810			Central and eastern Europe	
617	Slovakia	2003	Countrywide Integrated Noncommunicable Diseases Intervention Programme	20-64	20-64			845	574			Central and eastern Europe	
618	Slovakia	2008	Countrywide Integrated Noncommunicable Diseases Intervention Programme	20-64	20-64			547	376			Central and eastern Europe	
619	Slovakia	2011-2012	European Health Examination Survey	20-64	20-64	20-64	20-64	1,009	836	1,003	835	Central and eastern Europe	
620	Solomon Islands	2004	A genetic-ecological study of the risk factors for lifestyle-related diseases in Oceanian populations	20-64	20-64	20-64	20-64	185	175	183	175	Oceania	
621	Solomon Islands	2006	STEPS	20-64	20-64	20-64	20-64	1,226	880	1,220	875	Oceania	
622	Solomon Islands	2015	STEPS	20-64	20-64	20-64	20-64	904	743	903	740	Oceania	
623	Somalia	2016	The prevalence of selected risk factors for non-communicable diseases in Hargeisa, Somaliland: a cross-sectional study	20-64	20-64	20-64	20-64	886	127	884	127	Sub-Saharan Africa	
624	South Africa	1998	DHS	20-64	20-64	20-64	20-64	5,795	3,979	5,714	3,957	Sub-Saharan Africa	
625	South Africa	2003	DHS	20-64	20-64	20-64	20-64	3,392	2,279	3,350	2,261	Sub-Saharan Africa	
626	South Africa	2008	National Income Dynamics Study Wave I	20-64	20-64	20-64	20-64	5,861	3,762	5,735	3,688	Sub-Saharan Africa	
627	South Africa	2012	National Income Dynamics Study Wave III	20-64	20-64	20-64	20-64	7,908	5,276	7,851	5,245	Sub-Saharan Africa	
628	South Africa	2016	DHS	20-49	20-59	20-49	20-59	2,680	2,200	2,632	2,150	Sub-Saharan Africa	
629	South Africa	2017	National Income Dynamics Study Wave V	20-64	20-64	20-64	20-64	9,952	7,098	9,631	6,953	Sub-Saharan Africa	
630	South Africa	2007-2008	WHO Study on global AGEing and adult health (SAGE)	50-64	50-64	50-64	50-64	1,042	826	1,020	806	Sub-Saharan Africa	
631	South Africa	2008-2009	Cape Town Bellville South Cohort Study - Baseline evaluation I	35-64	35-64	35-64	35-64	471	134	469	134	Sub-Saharan Africa	
632	South Africa	2010-2011	National Income Dynamics Study Wave II	20-64	20-64	20-64	20-64	5,483	3,676	5,329	3,583	Sub-Saharan Africa	
633	South Africa	2011-2012	South Africa National Health and Nutrition Examination Survey	20-64	20-64	20-64	20-64	3,095	1,603	2,679	1,382	Sub-Saharan Africa	
634	South Africa	2014-2015	Health and Aging in Africa: A Longitudinal Study of an INDEPTH Community in South Africa (HAALSI)	40-64	40-64	40-64	40-64	1,485	1,209	1,477	1,197	Sub-Saharan Africa	
635	South Africa	2014-2015	National Income Dynamics Study Wave IV	20-64	20-64	20-64	20-64	9,453	6,876	9,163	6,756	Sub-Saharan Africa	
636	South Africa	2018-2020	Vukuzazi Study	20-64	20-64	20-64	20-64	8,524	3,682	8,519	3,681	Sub-Saharan Africa	
637	South Korea	1998	Korea National Health and Nutrition Examination Survey	20-64	20-64	20-64	20-64	3,678	3,201	3,666	3,188	East and southeast Asia and the Pacific	
638	South Korea	2001	Korea National Health and Nutrition Examination Survey	20-64	20-64	20-64	20-64	3,092	2,507	2,832	2,262	East and southeast Asia and the Pacific	
639	South Korea	2005	Korea National Health and Nutrition Examination Survey	20-64	20-64	20-64	20-64	2,558	1,960	2,544	1,948	East and southeast Asia and the Pacific	
640	South Korea	2007	Korea National Health and Nutrition Examination Survey	20-64	20-64	20-64	20-64	1,290	952	1,248	922	East and southeast Asia and the Pacific	
641	South Korea	2008	Korea National Health and Nutrition Examination Survey	20-64	20-64	20-64	20-64	2,935	2,211	2,923	2,199	East and southeast Asia and the Pacific	
642	South Korea	2009	Korea National Health and Nutrition Examination Survey	20-64	20-64	20-64	20-64	3,200	2,506	3,191	2,493	East and southeast Asia and the Pacific	
643	South Korea	2010	Korea National Health and Nutrition Examination Survey	20-64	20-64	20-64	20-64	2,702	2,046	2,675	2,019	East and southeast Asia and the Pacific	
644	South Korea	2011	Korea National Health and Nutrition Examination Survey	20-64	20-64	20-64	20-64	2,602	1,957	2,549	1,893	East and southeast Asia and the Pacific	
645	South Korea	2012	Korea National Health and Nutrition Examination Survey	20-64	20-64	20-64	20-64	2,484	1,798	2,366	1,656	East and southeast Asia and the Pacific	
646	South Korea	2013	Korea National Health and Nutrition Examination Survey	20-64	20-64	20-64	20-64	2,459	1,880	2,324	1,709	East and southeast Asia and the Pacific	
647	South Korea	2014	Korea National Health and Nutrition Examination Survey	20-64	20-64	20-64	20-64	2,343	1,686	2,127	1,466	East and southeast Asia and the Pacific	
648	South Korea	2015	Korea National Health and Nutrition Examination Survey	20-64	20-64	20-64	20-64	2,263	1,764	2,128	1,608	East and southeast Asia and the Pacific	
649	South Korea	2016	Korea National Health and Nutrition Examination Survey	20-64	20-64	20-64	20-64	2,522	1,912	2,516	1,903	East and southeast Asia and the Pacific	
650	South Korea	2017	Korea National Health and Nutrition Examination Survey	20-64	20-64	20-64	20-64	2,448	2,014	2,447	2,010	East and southeast Asia and the Pacific	
651	South Korea	2018	Korea National Health and Nutrition Examination Survey	20-64	20-64	20-64	20-64	2,515	2,009	2,502	1,995	East and southeast Asia and the Pacific	
652	South Korea	2019	Korea National Health and Nutrition Examination Survey	20-64	20-64	20-64	20-64	2,457	2,033	2,447	2,024	East and southeast Asia and the Pacific	
653	South Korea	2020	Korea National Health and Nutrition Examination Survey	20-64	20-64	20-64	20-64	2,243	1,890	2,214	1,858	East and southeast Asia and the Pacific	
654	South Korea	2021	Korea National Health and Nutrition Examination Survey	20-64	20-64	20-64	20-64	2,092	1,687	2,066	1,677	East and southeast Asia and the Pacific	
655	South Sudan	2017	Prevalence of hypertension and associated cardiovascular risk factors among adults aged 18-69 years in Juba City, South Sudan	20-64	20-64	20-64	20-64	445	354	445	354	Sub-Saharan Africa	
656	Spain	2003	The European Male Ageing Study		40-64		40-64		252		250	High-income western	
657	Spain	2004	Vioque J et al., Obesity 16(3):664-70, 2008	24-64	24-64	24-64	24-64	80	69	79	67	High-income western	
658	Spain	2004	Cardiovascular Risk Study in Castilla y León (RECCyL)	20-64	20-64	20-64	20-64	1,308	1,190	1,279	1,173	High-income western	
659	Spain	2009	Cardiovascular Risk Study in Castilla y León (RECCyL)	20-64	20-64	20-64	20-64	979	808	949	787	High-income western	
660	Spain	2013	ANIBES Study	20-64	20-64			828	757			High-income western	
661	Spain	2014	Cardiovascular Risk Study in Castilla y León (RECCyL)	20-64	20-64	20-64	20-64	836	707	807	680	High-income western	
662	Spain	1994-1995	Encuesta de Nutrición y Salud Comunidad Valenciana 1994-95 (ENCV)	20-64	20-64	20-64	20-64	649	592	646	588	High-income western	
663	Spain	1999-2000	Factores de riesgo en las islas Baleares: Estudio CORSAIB	35-64	35-64	35-64	35-64	678	615	678	615	High-income western	
664	Spain	2000-2005	CDC of the Canary Islands	20-64	20-64	20-64	20-64	3,786	2,767	3,782	2,764	High-income western	
665	Spain	2001-2002	Catalan Health Interview Survey	20-64	20-64	20-64	20-64	525	417	521	414	High-income western	
666	Spain	2001-2003	DIabetes, Nutrición y Obesidad en la población adulta de la Región de Murcia (DINO)	20-64	20-64	20-64	20-64	649	566	645	561	High-income western	
667	Spain	2003-2005	Registre Gironi del Cor (REGICOR)	35-64	35-64	35-64	35-64	642	612	637	611	High-income western	
668	Spain	2004-2006	PREVICTUS	60-64	60-64	60-64	60-64	597	568	597	568	High-income western	
669	Spain	2007-2009	Harmonizing Equation of Risk in Mediterraneon countries EXtremadura (HERMEX)	25-64	25-64	25-64	25-64	1,171	1,024	1,171	1,024	High-income western	
670	Spain	2008-2010	Study on Nutrition and Cardiovascular Risk in Spain	20-64	20-64	20-64	20-64	4,886	4,554	4,796	4,467	High-income western	
671	Sri Lanka	2006	STEPS	20-64	20-64	20-64	20-64	5,629	5,363	5,607	5,343	South Asia	
672	Sri Lanka	2014	STEPS	20-64	20-64	20-64	20-64	2,596	1,646	2,585	1,635	South Asia	
673	Sri Lanka	2021	STEPS	20-64	20-64	20-64	20-64	3,129	1,937	3,128	1,937	South Asia	
674	Sri Lanka	2005-2006	Sri Lanka Diabetes, Cardiovascular study (SLDCS)	20-64	20-64	20-64	20-64	2,351	1,470	2,150	1,381	South Asia	
675	State of Palestine	2010	STEPS	20-64	20-64	20-64	20-64	3,207	1,791	3,153	1,752	Central Asia, Middle East and north Africa	
676	State of Palestine	2022	STEPS	20-64	20-64	20-64	20-64	3,247	1,467	3,243	1,466	Central Asia, Middle East and north Africa	

	Country	Years	Survey/Study name/Citation	Age range used in anthropometric analyses		Age range used in hypertension analyses		Individuals used in anthropometric analyses		Individuals used in hypertension analyses		Region	Notes
				Women	Men	Women	Men	Women	Men	Women	Men		
677	State of Palestine	1996-1998	Ramallah study	20-64	20-64	20-64	20-64	829	388	822	387	Central Asia, Middle East and north Africa	
678	Sudan	2016	STEPS	20-64	20-64	20-64	20-64	4,088	2,280	4,087	2,280	Sub-Saharan Africa	
679	Sudan	2018	Prevalence and associated factors of hypertension among adults in Gadarif in eastern Sudan: a community-based study	20-64	20-64			333	139			Sub-Saharan Africa	
680	Suriname	2013-2015	The Healthy Life in Suriname Study (HELISUR)	20-64	20-64	20-64	20-64	676	383	676	383	Latin America and the Caribbean	
681	Sweden	1990	MONICA Gothenburg	25-64	25-64	25-64	25-64	772	775	763	766	High-income western	
682	Sweden	1995	MONICA Gothenburg	25-64	25-64	25-64	25-64	862	745	861	741	High-income western	
683	Sweden	2003	The European Male Ageing Study		40-64		40-64		260		257	High-income western	
684	Sweden	1992-1993	Population Study of Women in Gothenburg	62-64		62-64		247		244		High-income western	
685	Sweden	2001-2004	Swedish INTERGENE Cohort Study	24-64	24-64	24-64	24-64	1,463	1,335	1,461	1,332	High-income western	
686	Sweden	2004-2005	European Youth Heart Study (EYHS) II	20-21	20-21			111	70			High-income western	
687	Sweden	2004-2005	Population Study of Women in Gothenburg	38-50		38-50		493		455		High-income western	
688	Sweden	2016-2017	Population Study of Women in Gothenburg	38-50		38-50		570		569		High-income western	
689	Switzerland	2014-2015	National Nutrition Survey menuCH	20-64	20-64			919	756			High-income western	
690	Taiwan	2000	Social Environment and Biomarkers of Aging Study	50-64	50-64	50-64	50-64	175	207	175	207	East and southeast Asia and the Pacific	
691	Taiwan	1993-1996	Nutrition and Health Survey in Taiwan	20-64	20-64	20-64	20-64	1,272	1,085	1,242	1,064	East and southeast Asia and the Pacific	
692	Taiwan	2005-2008	Nutrition and Health Survey in Taiwan	20-64	20-64	20-64	20-64	897	797	855	772	East and southeast Asia and the Pacific	
693	Taiwan	2013-2016	Nutrition and Health Survey in Taiwan	20-64	20-64			1,063	950			East and southeast Asia and the Pacific	
694	Tajikistan	2016	STEPS	20-64	20-64	20-64	20-64	1,348	943	1,348	942	Central Asia, Middle East and north Africa	
695	Tanzania	2011	STEPS	25-64	25-64	25-64	25-64	1,512	999	1,491	996	Sub-Saharan Africa	
696	Tanzania	2012	STEPS	25-64	25-64	25-64	25-64	2,807	2,557	2,803	2,554	Sub-Saharan Africa	
697	Thailand	1997	Thailand National Health Examination Survey II	20-59	20-59			1,906	1,115			East and southeast Asia and the Pacific	
698	Thailand	2000	InterASIA	35-64	35-64	35-64	35-64	2,602	1,609	2,602	1,609	East and southeast Asia and the Pacific	
699	Thailand	2004	Thailand National Health Examination Survey III	20-64	20-64	20-64	20-64	12,529	11,362	12,493	11,333	East and southeast Asia and the Pacific	
700	Thailand	2009	Thailand National Health Examination Survey IV	20-64	20-64	20-64	20-64	6,815	5,970	6,813	5,960	East and southeast Asia and the Pacific	
701	Thailand	2014	Thailand National Health Examination Survey V	20-64	20-64	20-64	20-64	7,710	5,358	7,705	5,355	East and southeast Asia and the Pacific	
702	Thailand	2006-2007	Health Checks Ubon Ratchathani (HCUR) Study	20-64	20-64			304,797	285,596			East and southeast Asia and the Pacific	
703	Thailand	2019-2020	Thailand National Health Examination Survey VI	20-64	20-64	20-64	20-64	8,750	5,931	8,746	5,929	East and southeast Asia and the Pacific	
704	Timor-Leste	2014	STEPS	20-64	20-64	20-64	20-64	1,254	914	1,250	911	East and southeast Asia and the Pacific	
705	Togo	2010	STEPS	20-64	20-64	20-64	20-64	1,838	1,808	1,751	1,756	Sub-Saharan Africa	
706	Togo	2021-2022	STEPS	20-64	20-64	20-64	20-64	1,953	1,399	1,952	1,399	Sub-Saharan Africa	
707	Tokelau	2005	STEPS	20-64	20-64	20-64	20-64	253	223	252	223	Oceania	
708	Tokelau	2014	STEPS	20-64	20-64	20-64	20-64	257	240	256	234	Oceania	
709	Tonga	2004	STEPS	20-64	20-64	20-64	20-64	517	376	515	374	Oceania	
710	Tonga	2011	STEPS	20-64	20-64	20-64	20-64	1,388	861	1,384	859	Oceania	
711	Tonga	2017	STEPS	20-64	20-64	20-64	20-64	2,175	1,142	2,166	1,139	Oceania	
712	Tonga	2007-2008	Pacific Obesity Prevention in Communities - Ma'alahi Youth Project	20-22	20-22			50	27			Oceania	
713	Trinidad and Tobago	2011	STEPS	20-64	20-64	20-64	20-64	1,427	945	1,426	934	Latin America and the Caribbean	
714	Trinidad and Tobago	2013-2014	National Eye Survey	40-64	40-64	40-64	40-64	1,012	764	962	740	Latin America and the Caribbean	
715	Tunisia	2005	Tunisian National Survey 2005 (TAHINA)	35-64	35-64	35-64	35-64	3,981	2,966	3,941	2,945	Central Asia, Middle East and north Africa	
716	Tunisia	1996-1997	Ariana Healthy Project 1997	35-64	35-64	35-64	35-64	1,714	1,678	1,705	1,673	Central Asia, Middle East and north Africa	
717	Tunisia	1996-1997	Tunisian National Nutrition Survey 1996-1997	20-64	20-64	20-64	20-64	2,121	994	2,120	993	Central Asia, Middle East and north Africa	
718	Tunisia	2009-2010	ObeMaghreb	20-49	20-49	20-49	20-49	600	929	600	929	Central Asia, Middle East and north Africa	
719	Turkiye	1998	Turkish Adult Risk Factor Study	28-64	28-64	28-64	28-64	763	718	648	576	Central Asia, Middle East and north Africa	
720	Turkiye	2017	STEPS	20-64	20-64	20-64	20-64	2,608	1,674	2,608	1,672	Central Asia, Middle East and north Africa	
721	Turkiye	2003-2005	Prevalence of prehypertension and associated risk factors among Turkish adults: Trabzon Hypertension Study	20-64	20-64	20-64	20-64	2,353	2,027	2,352	2,027	Central Asia, Middle East and north Africa	
722	Turkiye	2007-2009	Balcova Heart Study	30-64	30-64	30-64	30-64	7,042	3,284	6,868	3,193	Central Asia, Middle East and north Africa	
723	Turkiye	2009-2012	Prevalence of diabetes and associated risk factors among adult population in Trabzon city	20-64	20-64			1,925	1,393			Central Asia, Middle East and north Africa	
724	Turkmenistan	2013	STEPS	20-64	20-64	20-64	20-64	2,721	1,859	2,717	1,849	Central Asia, Middle East and north Africa	
725	Turkmenistan	2018	STEPS	20-64	20-64	20-64	20-64	2,082	1,622	2,081	1,622	Central Asia, Middle East and north Africa	
726	Tuvalu	2015	STEPS	20-64	20-64	20-64	20-64	503	429	502	427	Oceania	
727	Uganda	2012	Prevalence, awareness and control of hypertension in Uganda	20-64	20-64			2,208	1,242			Sub-Saharan Africa	
728	Uganda	2014	STEPS	20-64	20-64	20-64	20-64	1,856	1,383	1,854	1,383	Sub-Saharan Africa	
729	Uganda	2023	STEPS	20-64	20-64	20-64	20-64	1,847	1,259	1,847	1,257	Sub-Saharan Africa	
730	Uganda	2011-2013	Gulu Health and Demographic Surveillance Site (HDSS)	20-64	20-64			2,125	1,461			Sub-Saharan Africa	
731	Uganda	2014-2015	Gulu Health and Demographic Surveillance Site (HDSS)	20-24	20-24			186	223			Sub-Saharan Africa	
732	Uganda	2018-2019	Scaling up Packages of Intervention for Cardiovascular disease prevention in selected sites in Europe and Sub-Saharan Africa (SPICES)	25-64	25-64	25-64	25-64	2,307	1,611	2,258	1,577	Sub-Saharan Africa	
733	Ukraine	2019	STEPS	20-64	20-64	20-64	20-64	2,064	1,297	2,047	1,274	Central and eastern Europe	
734	United Arab Emirates	2009	Gulf Cooperation Council World Health Survey	20-64	20-64	20-64	20-64	565	517	545	502	Central Asia, Middle East and north Africa	
735	United Arab Emirates	2017-2018	STEPS	20-64	20-64	20-64	20-64	2,199	1,989	2,195	1,986	Central Asia, Middle East and north Africa	
736	United Kingdom	1993	Health Survey for England (HSE)	20-64	20-64	20-64	20-64	5,362	5,057	5,346	5,040	High-income western	
737	United Kingdom	1994	Health Survey for England (HSE)	20-64	20-64	20-64	20-64	5,048	4,600	4,951	4,541	High-income western	
738	United Kingdom	1995	Scottish Health Survey (SHeS)	20-64	20-64	20-64	20-64	3,332	2,762	2,977	2,711	High-income western	
739	United Kingdom	1997	Health Survey for England (HSE)	20-64	20-64			2,824	2,557			High-income western	
740	United Kingdom	1998	Health Survey for England (HSE)	20-64	20-64	20-64	20-64	5,057	4,383	5,044	4,373	High-income western	
741	United Kingdom	1998	Scottish Health Survey (SHeS)	20-64	20-64	20-64	20-64	2,965	2,445	2,700	2,407	High-income western	
742	United Kingdom	1999	MRC National Survey of Health and Development	53-54	53-54	53-54	53-54	1,492	1,449	1,456	1,439	High-income western	
743	United Kingdom	2001	Health Survey for England (HSE)	20-64	20-64	20-64	20-64	4,589	3,931	4,575	3,918	High-income western	
744	United Kingdom	2002	Health Survey for England (HSE)	20-64	20-64	20-64	20-64	2,840	2,218	2,830	2,210	High-income western	

	Country	Years	Survey/Study name/Citation	Age range used in anthropometric analyses		Age range used in hypertension analyses		Individuals used in anthropometric analyses		Individuals used in hypertension analyses		Region	Notes
				Women	Men	Women	Men	Women	Men	Women	Men		
745	United Kingdom	2003	The European Male Ageing Study		40-64		40-64		239		237	High-income western	
746	United Kingdom	2003	Health Survey for England (HSE)	20-64	20-64	20-64	20-64	4,227	3,540	4,198	3,524	High-income western	
747	United Kingdom	2003	Scottish Health Survey (SHeS)	20-64	20-64	20-64	20-64	1,887	1,528	1,740	1,502	High-income western	
748	United Kingdom	2005	Health Survey for England (HSE)	20-64	20-64	20-64	20-64	2,024	1,625	2,005	1,613	High-income western	
749	United Kingdom	2006	Health Survey for England (HSE)	20-64	20-64	20-64	20-64	3,769	3,105	3,736	3,073	High-income western	
750	United Kingdom	2007	Health Survey for England (HSE)	20-64	20-64	20-64	20-64	1,772	1,467	1,752	1,450	High-income western	
751	United Kingdom	2008	Health Survey for England (HSE)	20-64	20-64	20-64	20-64	3,833	3,113	3,805	3,092	High-income western	
752	United Kingdom	2008	Scottish Health Survey (SHeS)	20-64	20-64	20-64	20-64	395	319	367	311	High-income western	
753	United Kingdom	2009	Health Survey for England (HSE)	20-64	20-64	20-64	20-64	1,147	949	1,133	938	High-income western	
754	United Kingdom	2009	Scottish Health Survey (SHeS)	20-64	20-64	20-64	20-64	425	310	401	309	High-income western	
755	United Kingdom	2010	Health Survey for England (HSE)	20-64	20-64	20-64	20-64	2,020	1,529	1,995	1,506	High-income western	
756	United Kingdom	2010	Scottish Health Survey (SHeS)	20-64	20-64	20-64	20-64	401	300	371	293	High-income western	
757	United Kingdom	2010	Understanding Society: the UK Household Longitudinal Study	20-64	20-64			6,175	4,529			High-income western	15
758	United Kingdom	2011	British Household Panel Survey	20-64	20-64			1,914	1,506			High-income western	15
759	United Kingdom	2011	Health Survey for England (HSE)	20-64	20-64	20-64	20-64	2,048	1,591	2,029	1,582	High-income western	
760	United Kingdom	2011	Scottish Health Survey (SHeS)	20-64	20-64	20-64	20-64	341	266	318	266	High-income western	
761	United Kingdom	2012	Health Survey for England (HSE)	20-64	20-64	20-64	20-64	1,907	1,448	1,887	1,433	High-income western	
762	United Kingdom	2012	Scottish Health Survey (SHeS)	20-64	20-64	20-64	20-64	353	286	325	274	High-income western	
763	United Kingdom	2013	Health Survey for England (HSE)	20-64	20-64	20-64	20-64	2,156	1,670	2,147	1,654	High-income western	
764	United Kingdom	2013	Scottish Health Survey (SHeS)	20-64	20-64	20-64	20-64	478	354	435	339	High-income western	
765	United Kingdom	2014	Health Survey for England (HSE)	20-64	20-64	20-64	20-64	1,873	1,523	1,866	1,515	High-income western	
766	United Kingdom	2014	Scottish Health Survey (SHeS)	20-64	20-64	20-64	20-64	486	364	452	361	High-income western	
767	United Kingdom	2015	Health Survey for England (HSE)	20-64	20-64	20-64	20-64	1,824	1,398	1,812	1,392	High-income western	
768	United Kingdom	2015	Scottish Health Survey (SHeS)	20-64	20-64	20-64	20-64	311	252	292	248	High-income western	
769	United Kingdom	2016	Health Survey for England (HSE)	20-64	20-64	20-64	20-64	1,700	1,280	1,691	1,275	High-income western	
770	United Kingdom	2016	Scottish Health Survey (SHeS)	20-64	20-64	20-64	20-64	349	267	317	253	High-income western	
771	United Kingdom	2017	Health Survey for England (HSE)	20-64	20-64	20-64	20-64	1,753	1,280	1,743	1,276	High-income western	
772	United Kingdom	2017	Scottish Health Survey	20-64	20-64	20-64	20-64	339	244	326	236	High-income western	
773	United Kingdom	2018	Health Survey for England (HSE)	20-64	20-64	20-64	20-64	1,637	1,214	1,618	1,199	High-income western	
774	United Kingdom	2018	Scottish Health Survey	20-64	20-64	20-64	20-64	410	283	397	273	High-income western	
775	United Kingdom	2019	Health Survey for England (HSE)	20-64	20-64	20-64	20-64	1,647	1,234	1,639	1,223	High-income western	
776	United Kingdom	2019	Scottish Health Survey	20-64	20-64	20-64	20-64	446	319	435	311	High-income western	
777	United Kingdom	1991-1992	Health Survey for England (HSE)	20-64	20-64	20-64	20-64	2,312	2,134	2,297	2,122	High-income western	
778	United Kingdom	1993-1997	EPIC Norfolk	40-64	40-64			9,744	7,731			High-income western	
779	United Kingdom	1998-2000	The British Regional Heart Study		60-64		60-64		1,233		1,231	High-income western	
780	United Kingdom	1999-2001	British Women's Heart and Health Study	60-64		60-64		1,011		1,006		High-income western	16
781	United Kingdom	1999-2004	Hertfordshire Cohort Study	59-64	59-64	59-64	59-64	425	652	423	651	High-income western	
782	United Kingdom	2000-2001	National Diet and Nutrition Survey (NDNS)	20-64	20-64	20-64	20-64	943	782	897	760	High-income western	
783	United Kingdom	2004-2005	English Longitudinal Study of Ageing Wave 2 2004-2005	52-64	52-64	52-64	52-64	1,887	1,562	1,504	1,212	High-income western	
784	United Kingdom	2008-2012	National Diet and Nutrition Survey (NDNS)	20-64	20-64	20-64	20-64	1,082	789	1,077	786	High-income western	
785	United Kingdom	2013-2014	National Diet and Nutrition Survey (NDNS)	20-64	20-64	20-64	20-64	452	279	452	278	High-income western	
786	United Kingdom	2015-2016	National Diet and Nutrition Survey (NDNS)	20-64	20-64	20-64	20-64	400	301	399	300	High-income western	
787	United Kingdom	2015-2018	British Cohort Study 1970	45-48	45-48			3,797	3,558			High-income western	
788	United Kingdom	2016-2019	National Diet and Nutrition Survey (NDNS)	20-64	20-64	20-64	20-64	525	377	330	234	High-income western	
789	United States of America	2006	Health and Retirement Study	53-64	53-64			1,382	985			High-income western	
790	United States of America	1988-1994	US NHANES III	20-64	20-64	20-64	20-64	6,132	5,565	5,923	5,360	High-income western	
791	United States of America	1990-1991	Coronary Artery Risk Development in Young Adults (CARDIA)	23-35	23-35	23-35	23-35	2,370	1,937	2,367	1,936	High-income western	
792	United States of America	1993-1998	Women's Health Initiative - Observational Study	50-64		50-64		49,292		48,738		High-income western	
793	United States of America	1996-1997	Study of Women's Health Across the Nation	40-55		40-55		3,163		3,160		High-income western	17
794	United States of America	1997-1999	Study of Women's Health Across the Nation	40-55		40-55		2,722		2,714		High-income western	17
795	United States of America	1998-2000	Study of Women's Health Across the Nation	40-55		40-55		2,560		2,556		High-income western	17
796	United States of America	1999-2000	US NHANES 1999-2000	20-64	20-64	20-64	20-64	1,465	1,434	1,400	1,403	High-income western	
797	United States of America	2001-2002	US NHANES 2001-2002	20-64	20-64	20-64	20-64	1,631	1,677	1,553	1,613	High-income western	
798	United States of America	2003-2004	US NHANES 2003-2004	20-64	20-64	20-64	20-64	1,482	1,528	1,383	1,459	High-income western	
799	United States of America	2005-2006	US NHANES 2005-2006	20-64	20-64	20-64	20-64	1,565	1,607	1,489	1,548	High-income western	
800	United States of America	2005-2006	National Social Life Health and Aging Project	57-64	57-64			450	490			High-income western	18
801	United States of America	2007-2008	US NHANES 2007-2008	20-64	20-64	20-64	20-64	2,003	1,977	1,928	1,904	High-income western	
802	United States of America	2009-2010	US NHANES 2009-2010	20-64	20-64	20-64	20-64	2,206	2,100	2,109	2,043	High-income western	
803	United States of America	2011-2012	US NHANES 2011-2012	20-64	20-64	20-64	20-64	1,933	1,955	1,837	1,901	High-income western	
804	United States of America	2013-2014	US NHANES 2013-2014	20-64	20-64	20-64	20-64	2,072	1,996	2,016	1,938	High-income western	
805	United States of America	2015-2016	US NHANES 2015-2016	20-64	20-64	20-64	20-64	2,000	1,890	1,950	1,852	High-income western	
806	United States of America	2017-2018	US NHANES 2017-2018	20-64	20-64	20-64	20-64	1,874	1,752	1,787	1,696	High-income western	
807	United States of America	2019-2020	US NHANES 2019-2020	20-64	20-64	20-64	20-64	1,208	1,152	1,143	1,110	High-income western	19
808	Uruguay	2006	STEPS	25-64	25-64	25-64	25-64	633	261	621	256	Latin America and the Caribbean	
809	Uruguay	2013	STEPS	20-64	20-64	20-64	20-64	1,304	744	1,298	743	Latin America and the Caribbean	
810	Uruguay	1999-2000	The Survey on Health, Well-Being, and Aging in Latin America and the Caribbean (SABE)	60-64	60-64			186	95			Latin America and the Caribbean	1
811	Uruguay	2011-2012	CESCAS Study	35-64	35-64	35-64	35-64	679	489	608	453	Latin America and the Caribbean	
812	Uruguay	2012-2016	Genotype, Phenotype and Environment of Hypertension in Uruguay (GEFA-HT-UY)	20-64	20-64	20-64	20-64	111	74	111	74	Latin America and the Caribbean	
813	Uzbekistan	2014	STEPS	20-64	20-64	20-64	20-64	2,080	1,443	2,078	1,440	Central Asia, Middle East and north Africa	
814	Uzbekistan	2019	STEPS	20-64	20-64	20-64	20-64	1,979	1,228	1,975	1,222	Central Asia, Middle East and north Africa	

	Country	Years	Survey/Study name/Citation	Age range used in anthropometric analyses		Age range used in hypertension analyses		Individuals used in anthropometric analyses		Individuals used in hypertension analyses		Region	Notes
				Women	Men	Women	Men	Women	Men	Women	Men		
815	Uzbekistan	2015-2016	Epidemiology of Diabetes and Prediabetes in Uzbekistan Screening Results	35-64	35-64			1,348	646			Central Asia, Middle East and north Africa	
816	Vanuatu	2005	STEPS	20-60	20-60	20-60	20-60	628	516	576	481	Oceania	
817	Vanuatu	2011	STEPS	25-64	25-64	25-64	25-64	1,731	1,686	1,722	1,674	Oceania	
818	Venezuela	2014-2015	Latin American Study of Nutrition and Health (ELANS)	20-64	20-64			495	458			Latin America and the Caribbean	
819	Venezuela	2014-2017	Maracaibo aging study Santa Rosa cohort	37-64	37-64	37-64	37-64	231	86	231	86	Latin America and the Caribbean	
820	Venezuela	2015-2017	Cardio-Metabolic Health Venezuelan Study (EVESCAM)	20-64	20-64	20-64	20-64	1,915	786	1,898	782	Latin America and the Caribbean	
821	Venezuela	2018-2020	Cardio-metabolic Health Venezuelan Study (EVESCAM) follow-up	22-64	22-64	22-64	22-64	630	224	609	220	Latin America and the Caribbean	
822	Viet Nam	2004	The Hypertension Management Programme in Rural Communes (Hanoi)	25-64	25-64	25-64	25-64	1,110	751	1,110	751	East and southeast Asia and the Pacific	
823	Viet Nam	2005	The Survey on Non-Communicable Disease Risk Factors	25-64	25-64	25-64	25-64	967	910	967	910	East and southeast Asia and the Pacific	
824	Viet Nam	2006	The Hypertension Management Programme in Rural Communes (Bavi)	25-64	25-64	25-64	25-64	551	317	551	317	East and southeast Asia and the Pacific	
825	Viet Nam	2007	The Hypertension Management Programme in Rural Communes (Phu Phuong)	25-64	25-64	25-64	25-64	539	314	539	314	East and southeast Asia and the Pacific	
826	Viet Nam	2009	The Hypertension Management Programme in Rural Communes (Phu Cuong)	25-64	25-64	25-64	25-64	595	309	595	309	East and southeast Asia and the Pacific	
827	Viet Nam	2009	STEPS	25-64	25-64	25-64	25-64	7,792	6,722	7,738	6,666	East and southeast Asia and the Pacific	
828	Viet Nam	2012	National Survey of Diabetes in Vietnam	30-64	30-64	30-64	30-64	5,335	4,797	5,332	4,797	East and southeast Asia and the Pacific	
829	Viet Nam	2015	STEPS	20-64	20-64	20-64	20-64	1,595	1,191	1,594	1,187	East and southeast Asia and the Pacific	
830	Viet Nam	2021	STEPS	20-64	20-64	20-64	20-64	1,694	1,682	1,690	1,675	East and southeast Asia and the Pacific	
831	Viet Nam	2001-2003	The National Epidemiological Survey on Hypertension and Its Risk Factors (North)	25-64	25-64	25-64	25-64	3,371	2,191	3,371	2,191	East and southeast Asia and the Pacific	
832	Viet Nam	2003-2004	The Survey on Heart Failure and Its Risk Factors	25-64	25-64	25-64	25-64	2,365	1,637	2,365	1,637	East and southeast Asia and the Pacific	
833	Viet Nam	2006-2008	The National Epidemiological Survey on Hypertension and Its Risk Factors (South)	25-64	25-64	25-64	25-64	1,831	1,168	1,831	1,168	East and southeast Asia and the Pacific	
834	Viet Nam	2008-2009	The Survey on Diabetes and Its Risk Factors	25-64	25-64	25-64	25-64	1,163	617	1,163	617	East and southeast Asia and the Pacific	
835	Yemen	2007-2009	Hypertension and Diabetes in Yemen (HYDY)	20-64	20-64	20-64	20-64	4,155	4,006	4,151	4,001	Central Asia, Middle East and north Africa	
836	Zambia	2008	STEPS	25-64	25-64	25-64	25-64	1,125	581	1,108	580	Sub-Saharan Africa	
837	Zambia	2017	STEPS	20-64	20-64	20-64	20-64	2,070	1,385	2,055	1,376	Sub-Saharan Africa	

1. The bibliographic citation for this data source is: Pelaez, Martha, Alberto Palloni, Cecilia Albala, Juan C. Alfonso, Roberto Ham-Chande, Anselm Hennis, Maria Lucia Lebrao, Esther Lesn-Diaz, Edith Pantelides, and Omar Prats. SABE - SURVEY ON HEALTH, WELL-BEING, AND AGING IN LATIN AMERICA AND THE CARIBBEAN, 2000 [Computer file]. ICPSR version. Washington, D.C.: Pan American Health Organization/World Health Organization (PAHO/WHO) [producers], 2004. Ann Arbor, MI: Inter-university Consortium for Political and Social Research [distributor], 2005.
2. Sciensano, OD Public health and surveillance (2020). Health Interview Survey 2018 [Data file and code book]. Conditionally obtainable from the Sciensano website: https://www.sciensano.be/en/node/55737/health-interview-survey-microdata-request-procedure.
3. This research uses data from China Health and Nutrition Survey (CHNS). We thank the National Institute for Health (NIH), the Eunice Kennedy Shriver National Institute of Child Health and Human Development (NICHD) for R01 HD30880, National Institute on Aging (NIA) for R01 AG065357, National Institute of Diabetes and Digestive and Kidney Diseases (NIDDK) for R01 DK104371 and P30 DK056350, National Heart, Lung, and Blood Institute (NHLBI) for R01 HL108427, the NIH Fogarty grant D43 TW009077, the Carolina Population Center for P2C HD050924 and P30 AG066615. We also thank the National Institute for Nutrition and Health, China Center for Disease Control and Prevention; Beijing Municipal Center for Disease Control and Prevention; the Chinese National Human Genome Center at Shanghai; and the China-Japan Friendship Hospital, National Health Commission of China.
4. Santé publique France, en tant qu'investigateur principal, promoteur et financeur de l'étude ENNS.; Aux Centres d'examen de santé de la Caisse nationale d'assurance maladie des travailleurs salariés (CnamTS) et leurs laboratoires.
5. Santé publique France, en tant qu'investigateur principal, promoteur et financeur de l'étude Esteban.; Aux Centres d'examen de santé de la Caisse nationale d'assurance maladie des travailleurs salariés (CnamTS) et leurs laboratoires.
6. Dr Matthias Schulze contributed the data from the EPIC Potsdam study.
7. Data have been provided by the Study of Health in Pomerania (SHIP) from the University Medicine Greifswald.
8. The authors thank the Heinz Nixdorf Foundation [Chairman: Martin Nixdorf; Past Chairman: Dr jur. Gerhard Schmidt], for their generous support of this study. Parts of the study were also supported by the German Research Council (DFG) [DFG project: EI 969/2-3, ER 155/6-1;6-2, HO 3314/2-1;2-2;2-3;4-3, INST 58219/32-1, JO 170/8-1, KN 885/3-1, PE 2309/2-1, SI 236/8-1;9-1;10-1,], the German Ministry of Education and Science [BMBF project: 01EG0401, 01GI0856, 01GI0860, 01GS0820_WB2-C, 01ER1001D, 01GI0205], the Ministry of Innovation, Science, Research and Technology, North Rhine-Westphalia (MIWFT-NRW), the Else Kröner-Fresenius-Stiftung [project: 2015_A119] and the German Social Accident Insurance [DGUV project: FF-FP295]. Furthermore the study was supported by the Competence Network for HIV/AIDS, the deanship of the University Hospital and IFORES of the University Duisburg-Essen, the European Union, the German Competence Network Heart Failure, Kulturstiftung Essen, the Protein Research Unit within Europe (PURE), the Dr. Werner-Jackstädt Stiftung and the following companies: Celgene GmbH München, Imatron/GE-Imatron, Janssen, Merck KG, Philips, ResMed Foundation, Roche Diagnostics, Sarstedt AG&Co, Siemens HealthCare Diagnostics, Volkswagen Foundation. The authors express their gratitude to all study participants of the Heinz Nixdorf Recall (HNR) Study, the personnel of the HNR study center and the EBT-scanner facilities, the investigative group and all former employees of the HNR study. The authors also thank the Advisory Board of the HNR Study: T. Meinertz, Hamburg, Germany (Chair); C. Bode, Freiburg, Germany; P.J. de Feyter, Rotterdam, Netherlands; B. Güntert, Hall i.T., Austria; F. Gutzwiller, Bern, Switzerland; H. Heinen, Bonn, Germany;O. Hess (†), Bern, Switzerland; B. Klein (†), Essen, Germany; H. Löwel, Neuherberg, Germany; M. Reiser, Munich, Germany; G. Schmidt (†), Essen, Germany; M. Schwaiger, Munich, Germany; C. Steinmüller, Bonn, Germany; T. Theorell, Stockholm, Sweden; and S.N Willich, Berlin, Germany.
9. The CARRS Study was funded in part by the National Heart, Lung, and Blood Institute (NHLBI), National Institutes of Health (NIH), Department of Health and Human Services, under Contract No. HHSN268200900026C, the United Health Group, Minneapolis, MN, USA, and by the by the National Heart, Lung, And Blood Institute of the National Institutes of Health under Award Number P01HL154996.
10. The study was supported by the grant of the Ministry of Healthcare of the Republic of Kazakhstan "National Programme for the Introduction of Personalized and Preventive Medicine in The Republic of Kazakhstan (2021–2023)" (Grant number OR12165486).
11. The Longitudinal Aging Study Amsterdam is supported by a grant from the Netherlands Ministry of Health Welfare and Sports, Directorate of Long-Term Care.
12. The bibliographic citation for this data source is: Palloni, Alberto, Ana Luisa Davila, and Melba Sanchez-Ayendez. Puerto Rican Elderly: Health Conditions (PREHCO) Project, 2002-2003, 2006-2007. ICPSR34596-v1. Ann Arbor, MI: Inter-university Consortium for Political and Social Research[distributor], 2013-09-13. doi:10.3886/ICPSR34596.v1.
13. Dr Take Naseri (Ministry of Health, Samoa), and Muagututia Sefuiva Reupena (Lutia I Puava Ae Mapu I Fagalele) contributed to the GWAS studies in Samoa.
14. The SH2012 and SH2 studies are supported by infrastructure funding from the Singapore Ministry of Health (Population Health Metrics Population Health Metrics and Analytics PHMA), National University of Singapore and National University Health System, Singapore.
15. University of Essex. Institute for Social and Economic Research and National Centre for Social Research, Understanding Society: Waves 2 and 3 Nurse Health Assessment, 2010- 2012 [data collection]. 5th Edition. UK Data Service. SN:7251. http://doi.org/10.5255/UKDA-SN-7251-5".
16. The British Women's Heart and Health Study is supported by the British Heart Foundation (PG/13/66/30442). British Women's Heart and Health Study data are available to bona fide researchers for research purposes. Please refer to the BWHHS data sharing policy at http://www.ucl.ac.uk/british-womens-heart-health-study.
17. The bibliographic citation for this data source is: Sutton-Tyrrell, Kim, Faith Selzer, MaryFran Sowers, Robert Neer, Lynda Powell, Ellen Gold, Gail Greendale, Gerson Weiss, Karen Matthews, and Sonja McKinlay. Study of Women's Health Across the Nation (SWAN), 1996-1997: Baseline Dataset. ICPSR28762-v2. Ann Arbor, MI: Inter-university Consortium for Political and Social Research[distributor], 2014-02-04. http://doi.org/10.3886/ICPSR28762.v2.
18. The bibliographic citation for this data source is: Waite, Linda J., Kathleen Cagney, William Dale, Elbert Huang, Edward O. Laumann, Martha McClintock, Colm A. O'Muircheartaigh, L. Phillip Schumm, and Benjamin Cornwell. National Social Life, Health, and Aging Project (NSHAP): Wave 2 and Partner Data Collection. ICPSR34921-v1. Ann Arbor, MI: Inter-university Consortium for Political and Social Research [distributor], 2014-04-29. https://doi.org/10.3886/ICPSR34921.v1.
19. Due to the COVID-19 pandemic the NHANES 2019-2020 cycle was not completed. As a result the data are not nationally representative and considered subnational.

Appendix Table 3. Number of studies and participants aged 20-64 years, by region.

Region[1]	Number of studies[2]	Number of participants[2]	% women	Mean age (years)
**Central and eastern Europe**	74 (52)	278,286 (145,594)	56.8	45.1
**High-income western**	189 (164)	1,005,702 (712,474)	57.5	46.3
**Latin America and the Caribbean**	136 (104)	562,766 (337,171)	59.2	40.4
**Central Asia, Middle East and north Africa**	113 (107)	751,357 (723,612)	55.3	43.4
**South Asia**	60 (47)	1,087,979 (981,556)	74.1	36.8
**Sub-Saharan Africa**	114 (101)	402,081 (377,346)	59.3	38.9
**East and southeast Asia and the Pacific**	111 (97)	3,390,300 (2,105,601)	53.5	41.7
**Oceania**	40 (38)	62,853 (60,619)	57.0	41.0
**Global**	**837 (710)**	**7,541,324 (5,443,973)**	**58.1**	**41.6**

[1]See Appendix Table 1 for list of countries in each region.

[2]Numbers in brackets show the subset of studies and participants with blood pressure data.

Appendix Table 4. Percentiles of waist-to-height ratio and body-mass index (BMI), by region. See Figure 1 for graphical presentation.

	Waist-to-height-ratio					BMI				
	**2.5%**	**25%**	**Median**	**75%**	**97.5%**	**2.5%**	**25%**	**Median**	**75%**	**97.5%**
	**Female**									
**High-income western**	0.39	0.45	0.50	0.57	0.73	18.77	22.39	25.17	29.14	40.83
**Central and eastern Europe**	0.39	0.46	0.52	0.59	0.73	18.65	23.00	26.33	30.47	40.06
**Latin America and the Caribbean**	0.42	0.51	0.57	0.63	0.77	18.78	23.76	27.05	30.98	40.99
**Central Asia, Middle East and north Africa**	0.41	0.52	0.59	0.65	0.78	18.43	24.01	27.59	31.47	40.51
**South Asia**	0.38	0.46	0.51	0.57	0.69	15.98	19.83	22.41	25.54	33.55
**Sub-Saharan Africa**	0.40	0.47	0.53	0.59	0.75	16.94	21.25	24.65	29.51	41.31
**East and southeast Asia and the Pacific**	0.39	0.46	0.51	0.56	0.67	17.07	20.74	23.10	26.06	33.46
**Oceania**	0.43	0.53	0.60	0.67	0.81	19.44	26.05	30.86	36.06	47.88
**Global**	**0.39**	**0.47**	**0.52**	**0.58**	**0.72**	**17.06**	**21.23**	**24.04**	**27.77**	**37.79**
	**Male**									
**High-income western**	0.42	0.49	0.53	0.58	0.70	19.93	23.94	26.33	29.19	37.53
**Central and eastern Europe**	0.41	0.48	0.53	0.58	0.69	19.49	23.59	26.21	29.27	36.68
**Latin America and the Caribbean**	0.41	0.49	0.54	0.59	0.70	18.91	23.23	26.01	29.09	37.02
**Central Asia, Middle East and north Africa**	0.40	0.49	0.54	0.59	0.69	18.00	22.63	25.56	28.64	35.78
**South Asia**	0.38	0.45	0.50	0.55	0.65	16.16	19.76	22.39	25.15	31.42
**Sub-Saharan Africa**	0.38	0.44	0.47	0.52	0.64	16.77	20.06	22.19	25.08	33.18
**East and southeast Asia and the Pacific**	0.38	0.44	0.48	0.52	0.62	17.29	20.41	22.39	24.74	30.81
**Oceania**	0.42	0.49	0.55	0.61	0.75	19.61	24.49	28.26	32.79	43.98
**Global**	**0.39**	**0.45**	**0.50**	**0.55**	**0.66**	**17.38**	**21.06**	**23.53**	**26.62**	**34.30**

Appendix Table 5. C-statistics and continuous net-reclassification improvement (NRI) for hypertension from three logistic models using body-mass index (BMI), waist circumference (WC), and both. NRI values were calculated relative to a logistic regression model using no adiposity measure. All models included terms for age and study year, and the global model included region. Regional models used only the data from that region.

	Women						Men					
	C-statistic			NRI			C-statistic			NRI		
	BMI	WC	Both	BMI	WC	Both	BMI	WC	Both	BMI	WC	Both
**Central and eastern Europe**	0.810	0.808	0.813	0.568	0.549	0.579	0.765	0.763	0.767	0.467	0.453	0.481
**High-income western**	0.788	0.788	0.790	0.512	0.506	0.525	0.757	0.757	0.759	0.439	0.428	0.449
**Latin America and the Caribbean**	0.808	0.808	0.810	0.445	0.446	0.462	0.765	0.766	0.768	0.445	0.458	0.468
**Central Asia, Middle East and north Africa**	0.791	0.791	0.793	0.395	0.403	0.418	0.755	0.754	0.757	0.405	0.411	0.426
**South Asia**	0.749	0.747	0.751	0.389	0.359	0.405	0.720	0.722	0.725	0.397	0.416	0.431
**Sub-Saharan Africa**	0.781	0.782	0.784	0.347	0.367	0.372	0.735	0.735	0.738	0.344	0.363	0.371
**East and southeast Asia and the Pacific**	0.755	0.750	0.756	0.456	0.425	0.468	0.718	0.715	0.721	0.433	0.417	0.448
**Oceania**	0.786	0.784	0.787	0.416	0.413	0.430	0.744	0.748	0.748	0.455	0.478	0.487
**Global**	**0.780**	**0.778**	**0.781**	**0.440**	**0.420**	**0.453**	**0.741**	**0.741**	**0.744**	**0.421**	**0.416**	**0.438**

Appendix Figure 1: Number of studies with participants aged 20-64 years in each country.

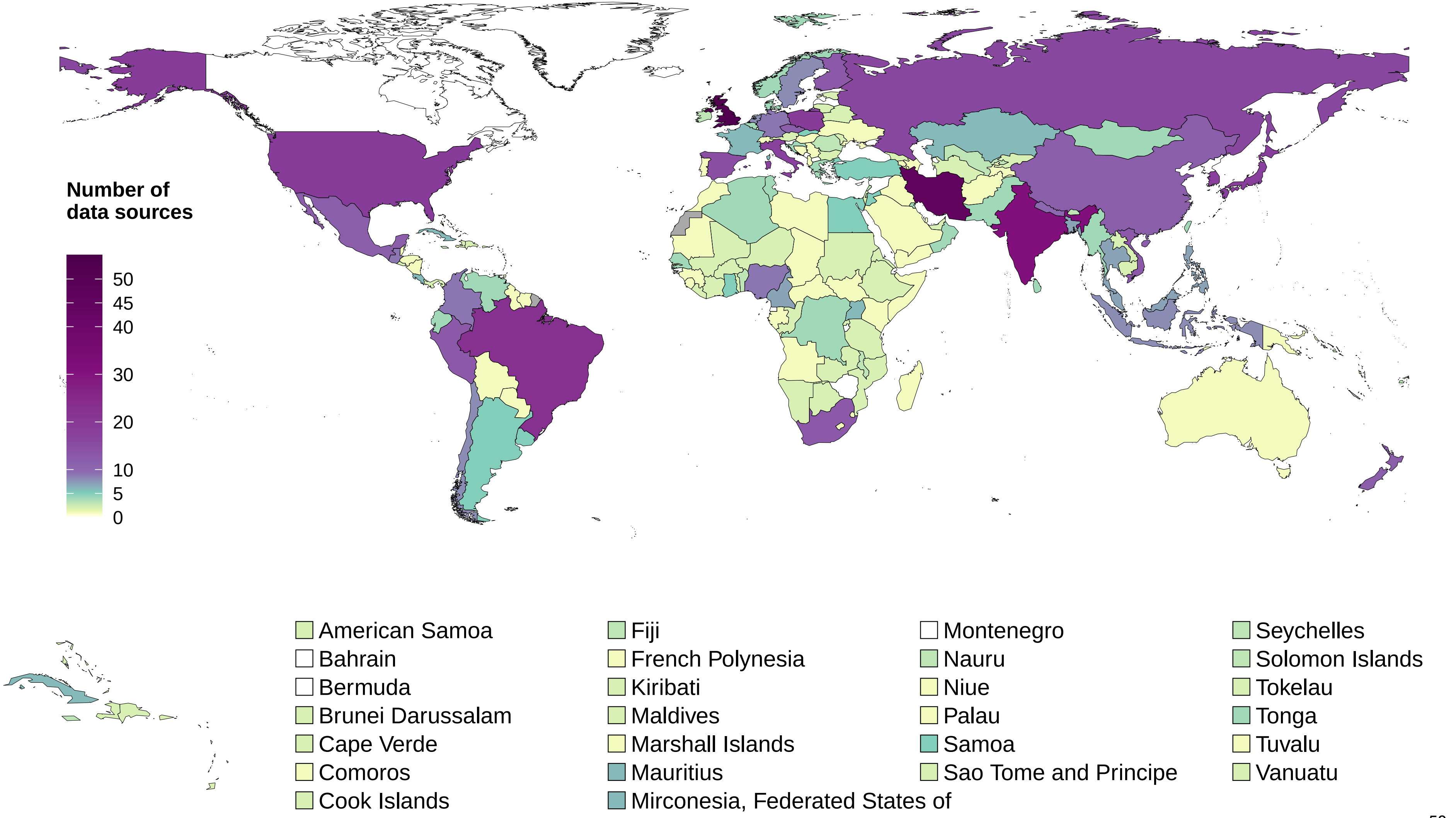
Number of data sources
50
45
40
30
20
10
5
0
American Samoa
Bahrain
Bermuda
Brunei Darussalam
Cape Verde
Comoros
Cook Islands
Fiji
French Polynesia
Kiribati
Maldives
Marshall Islands
Mauritius
Mirconesia, Federated States of
Montenegro
Nauru
Niue
Palau
Samoa
Sao Tome and Principe
Seychelles
Solomon Islands
Tokelau
Tonga
Tuvalu
Vanuatu

Appendix Figure 2: Flowchart of data cleaning.

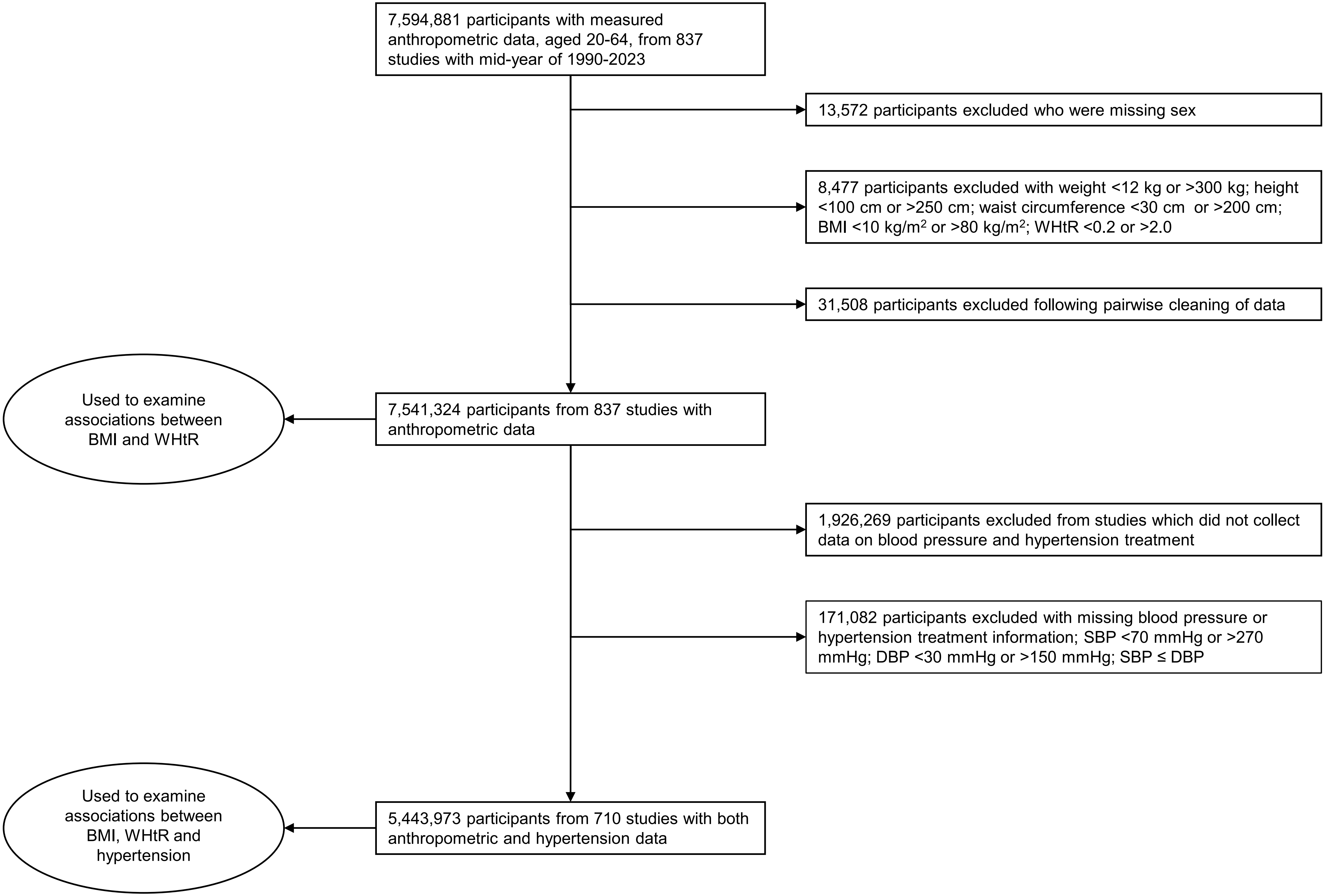
7,594,881 participants with measured anthropometric data, aged 20-64, from 837 studies with mid-year of 1990-2023
13,572 participants excluded who were missing sex
8,477 participants excluded with weight <12 kg or >300 kg; height <100 cm or >250 cm; waist circumference <30 cm or >200 cm; BMI <10 kg/m^2 or >80 kg/m^2; WHtR <0.2 or >2.0
31,508 participants excluded following pairwise cleaning of data
Used to examine associations between BMI and WHtR
7,541,324 participants from 837 studies with anthropometric data
1,926,269 participants excluded from studies which did not collect data on blood pressure and hypertension treatment
171,082 participants excluded with missing blood pressure or hypertension treatment information; SBP <70 mmHg or >270 mmHg; DBP <30 mmHg or >150 mmHg; SBP ≤ DBP
Used to examine associations between BMI, WHtR and hypertension
5,443,973 participants from 710 studies with both anthropometric and hypertension data

Appendix Figure 3: Waist-to-height ratio (WHtR) adjusted for body-mass index (BMI) and age, by region.

The graph shows mean regional WHtR for the global mean (across all participants) of BMI (25.0 kg/m^2 for women and 24.2 kg/m^2 for men) and age (41.3 years for women and 42.0 years for men). See Appendix Figure 13 for results using waist circumference (WC).

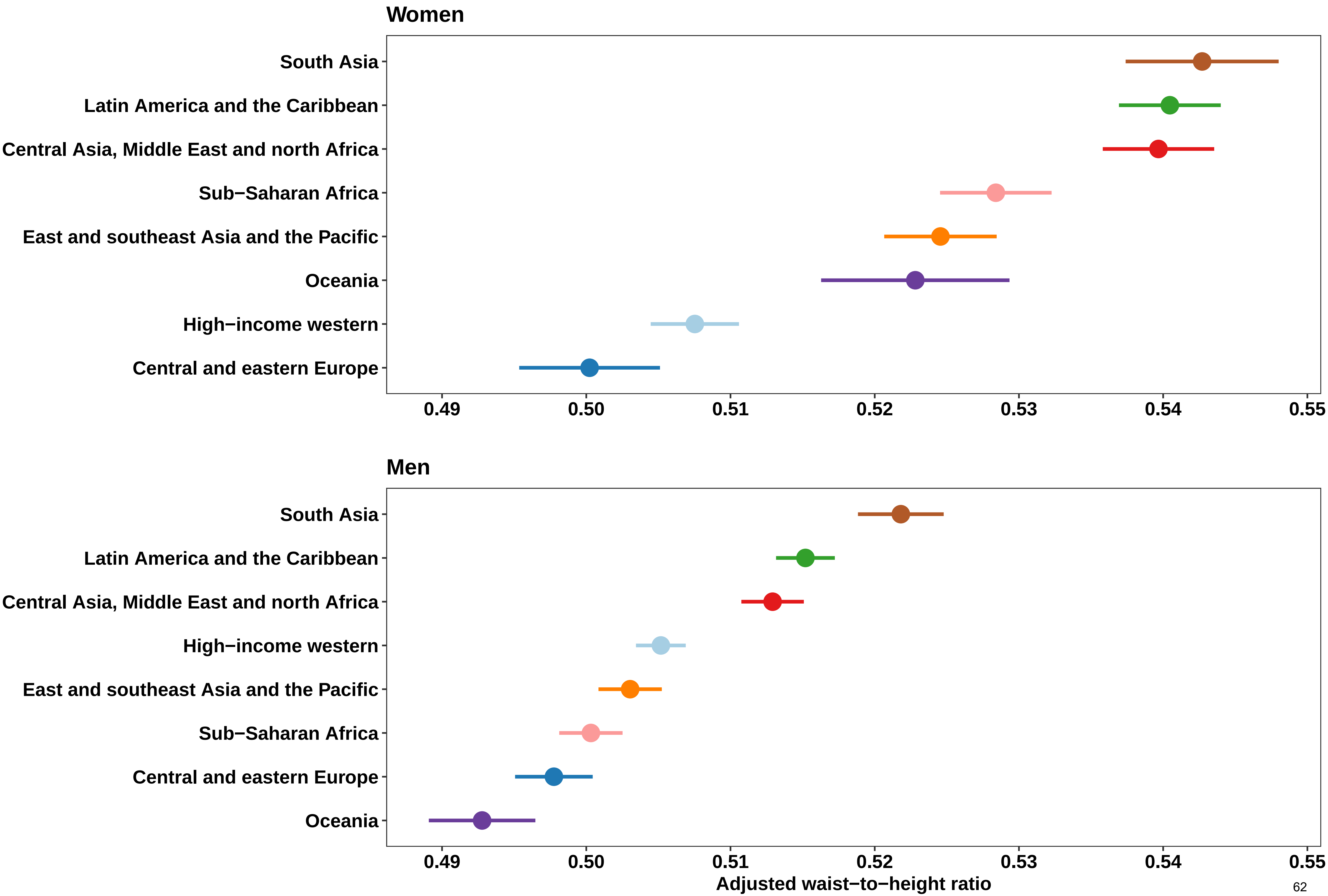

Women
South Asia
Latin America and the Caribbean
Central Asia, Middle East and north Africa
Sub−Saharan Africa
East and southeast Asia and the Pacific
Oceania
High−income western
Central and eastern Europe
0.49
0.50
0.51
0.52
0.53
0.54
0.55
Men
South Asia
Latin America and the Caribbean
Central Asia, Middle East and north Africa
High−income western
East and southeast Asia and the Pacific
Sub−Saharan Africa
Central and eastern Europe
Oceania
0.49
0.50
0.51
0.52
0.53
0.54
0.55
Adjusted waist−to−height ratio

Appendix Figure 4: Comparison of waist-to-height ratio (WHtR) and BMI relationship using linear and categorical BMI terms. The graph shows predicted WHtR at different BMI levels from the same regression model used in Appendix Figure 3, using data from all regions, and adjusted for age. The graph shows the WHtR for the global mean (across all participants) age (41.3 years for women and 42.0 years for men).

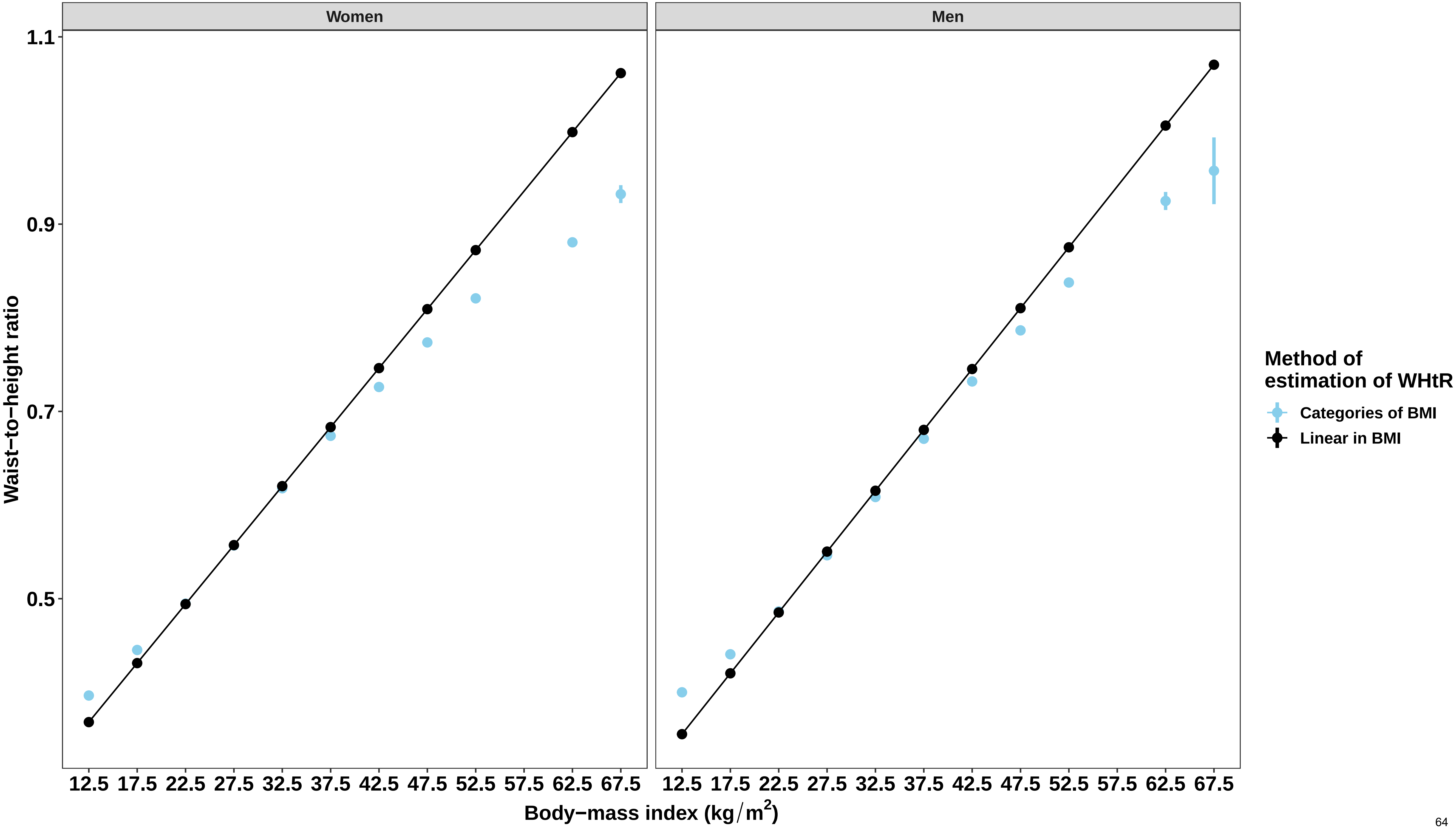

Women
Men
Waist−to−height ratio
1.1
0.9
0.7
0.5
12.5
17.5
22.5
27.5
32.5
37.5
42.5
47.5
52.5
57.5
62.5
67.5
Body−mass index (kg/m2)
Method of
estimation of WHtR
Categories of BMI
Linear in BMI

Appendix Figure 5: Mean systolic blood pressure (SBP) at different levels of waist-to-height ratio (WHtR) and body-mass index (BMI), by region.

Cells with ≤30 participants have been excluded from the figure because the results are less stable than at larger numbers. The number on each panel indicates the mean SBP among all participants in each region. See Appendix Figure 25 for results using waist circumference (WC).

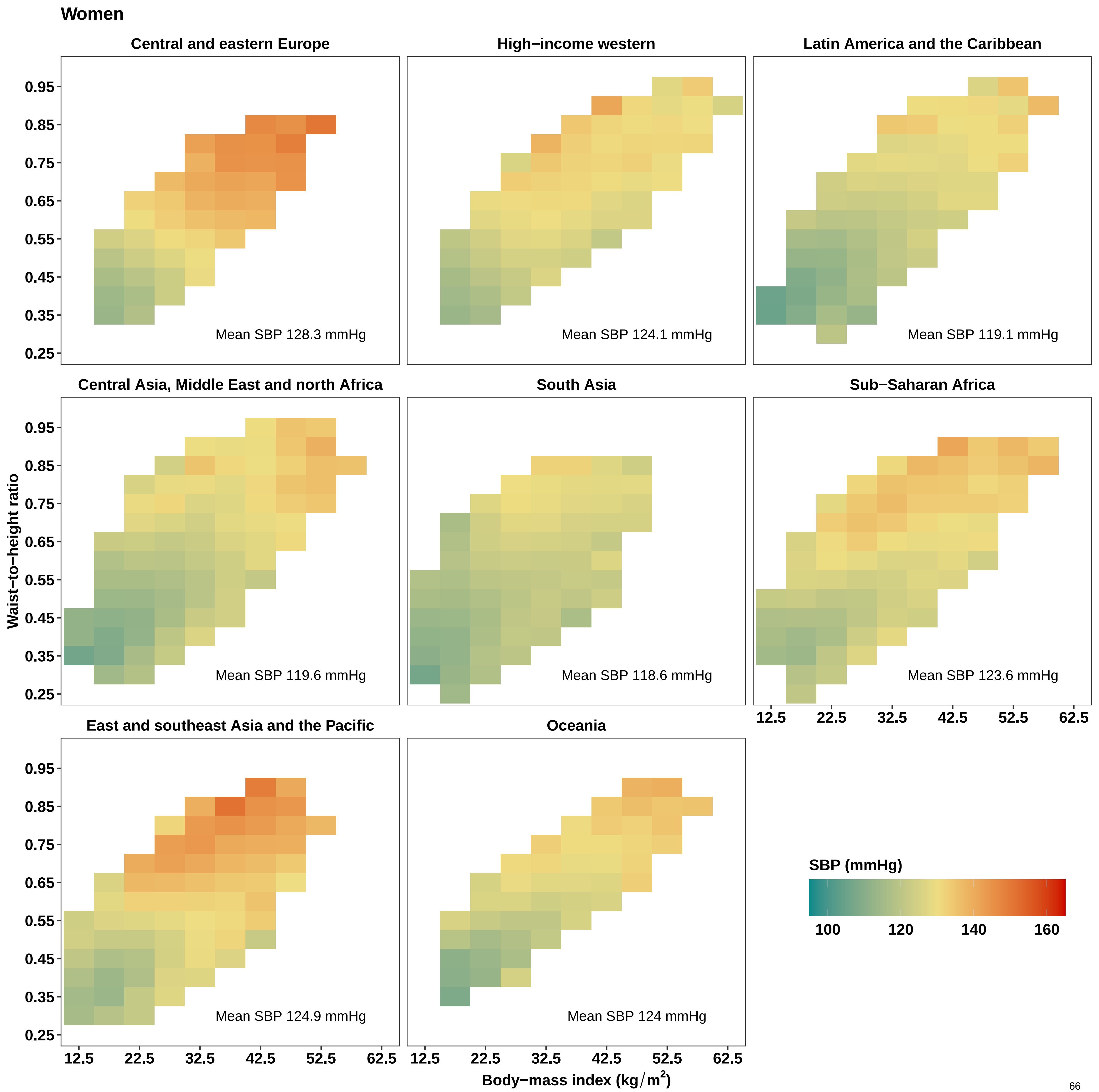

Women
Central and eastern Europe
High−income western
Latin America and the Caribbean
Central Asia, Middle East and north Africa
South Asia
Sub−Saharan Africa
East and southeast Asia and the Pacific
Oceania
Mean SBP 128.3 mmHg
Mean SBP 124.1 mmHg
Mean SBP 119.1 mmHg
Mean SBP 119.6 mmHg
Mean SBP 118.6 mmHg
Mean SBP 123.6 mmHg
Mean SBP 124.9 mmHg
Mean SBP 124 mmHg
Waist−to−height ratio
0.95
0.85
0.75
0.65
0.55
0.45
0.35
0.25
12.5
22.5
32.5
42.5
52.5
62.5
Body−mass index (kg/m²)
SBP (mmHg)
100
120
140
160

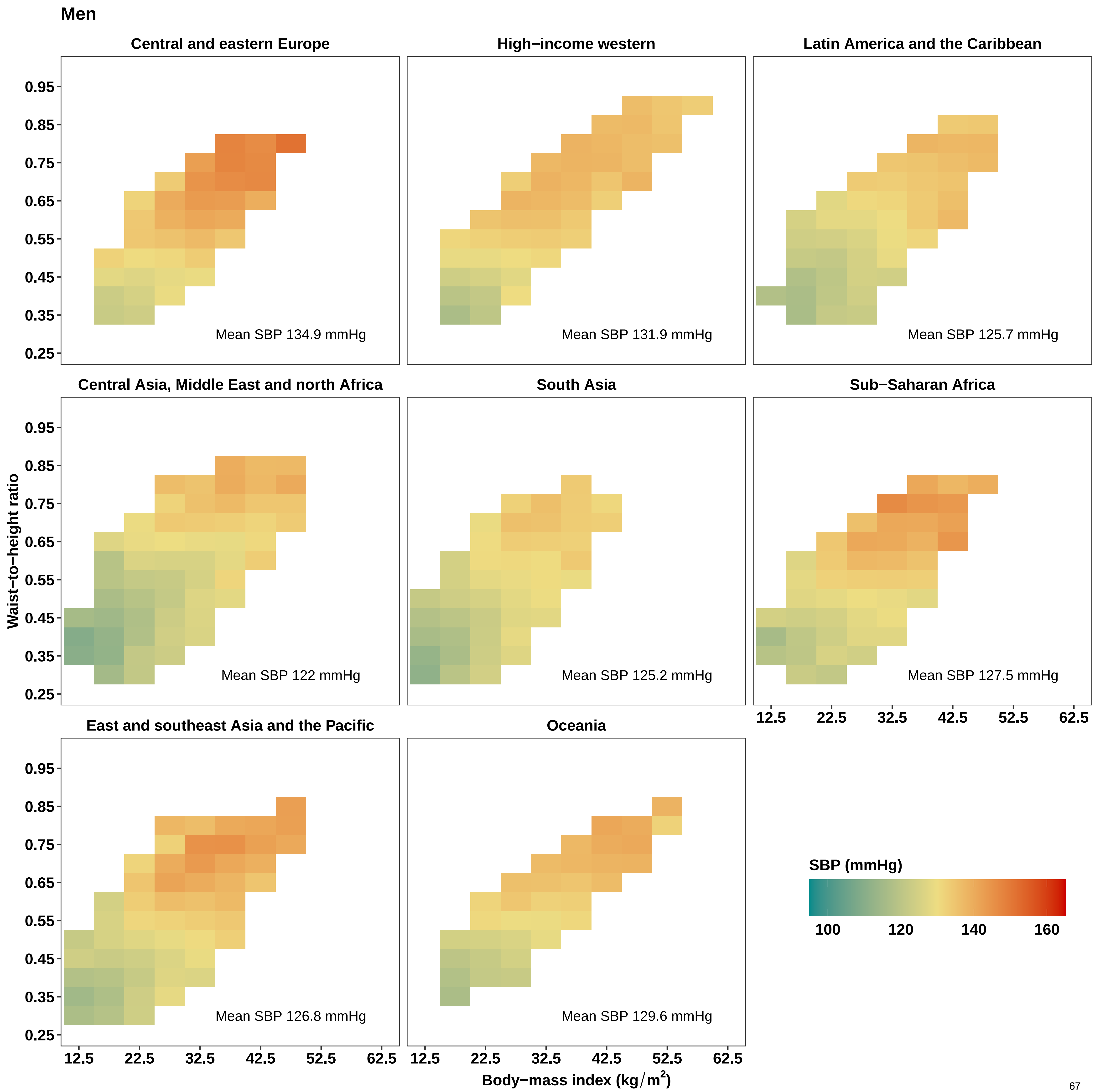

Men
Central and eastern Europe
High−income western
Latin America and the Caribbean
Central Asia, Middle East and north Africa
South Asia
Sub−Saharan Africa
East and southeast Asia and the Pacific
Oceania
Mean SBP 134.9 mmHg
Mean SBP 131.9 mmHg
Mean SBP 125.7 mmHg
Mean SBP 122 mmHg
Mean SBP 125.2 mmHg
Mean SBP 127.5 mmHg
Mean SBP 126.8 mmHg
Mean SBP 129.6 mmHg
Waist−to−height ratio
0.95
0.85
0.75
0.65
0.55
0.45
0.35
0.25
Body−mass index (kg/m2)
12.5
22.5
32.5
42.5
52.5
62.5
SBP (mmHg)
100
120
140
160

Appendix Figure 6: Mean diastolic blood pressure (DBP) at different levels of waist-to-height ratio (WHtR) and body-mass index (BMI), by region.

Cells with ≤30 participants have been excluded from the figure because the results are less stable than at larger numbers. The number on each panel indicates the mean DBP among all participants in each region. See Appendix Figure 26 for results using waist circumference (WC).

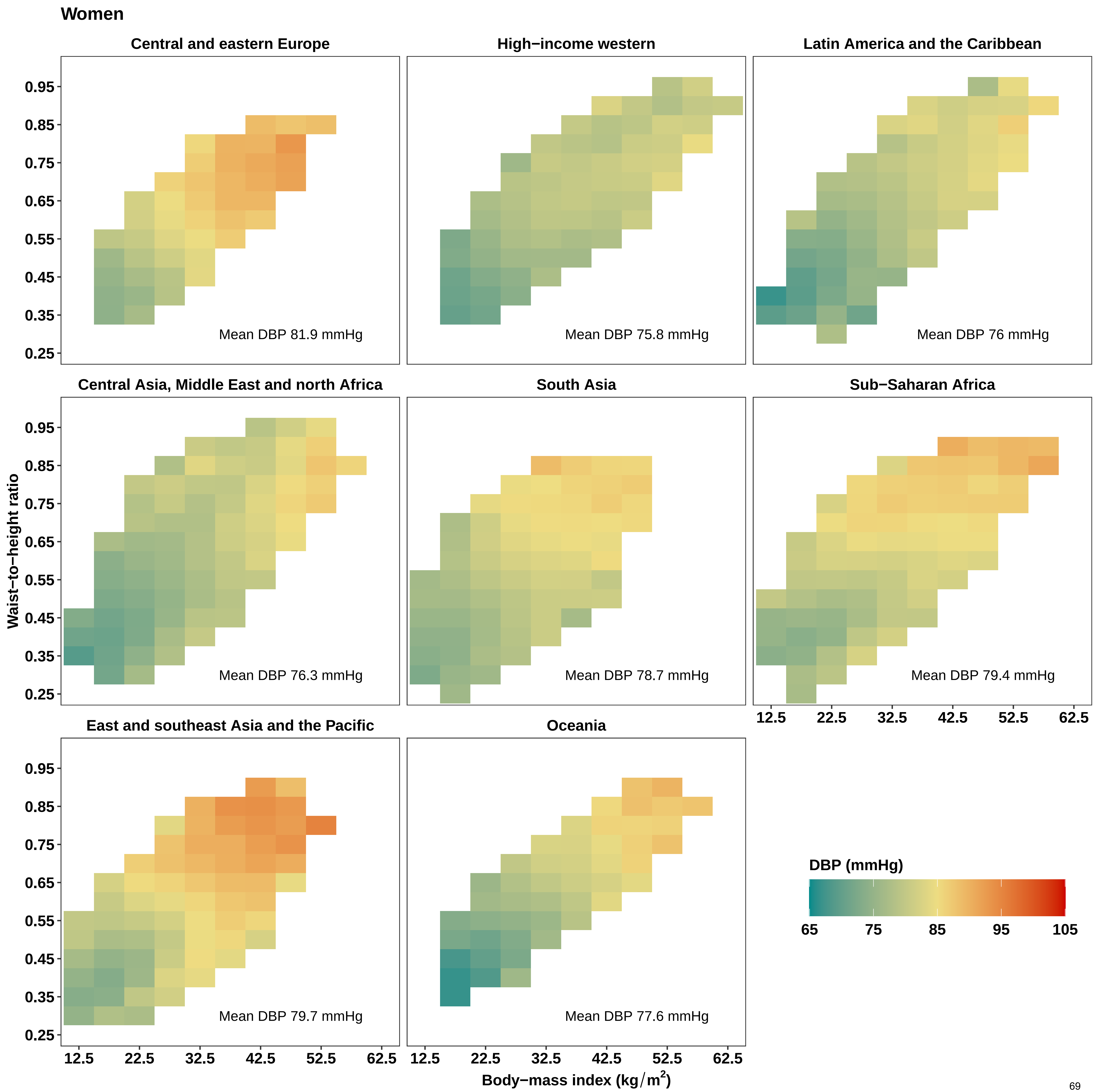
Women
Central and eastern Europe
Mean DBP 81.9 mmHg
High−income western
Mean DBP 75.8 mmHg
Latin America and the Caribbean
Mean DBP 76 mmHg
Central Asia, Middle East and north Africa
Mean DBP 76.3 mmHg
South Asia
Mean DBP 78.7 mmHg
Sub−Saharan Africa
Mean DBP 79.4 mmHg
East and southeast Asia and the Pacific
Mean DBP 79.7 mmHg
Oceania
Mean DBP 77.6 mmHg
Waist−to−height ratio
0.95
0.85
0.75
0.65
0.55
0.45
0.35
0.25
Body−mass index (kg/m^2)
12.5
22.5
32.5
42.5
52.5
62.5
DBP (mmHg)
65
75
85
95
105

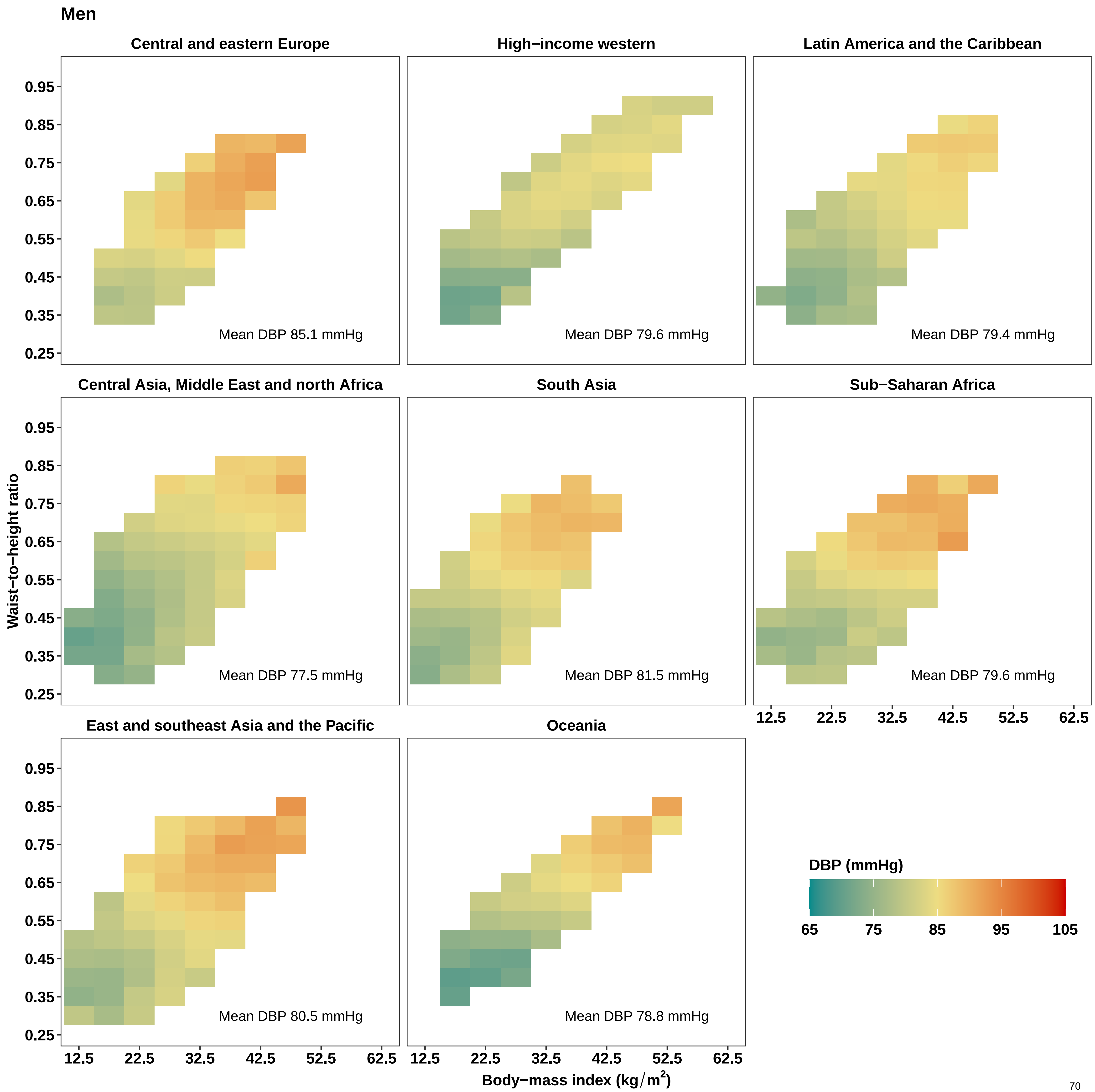

Men
Central and eastern Europe
High−income western
Latin America and the Caribbean
Central Asia, Middle East and north Africa
South Asia
Sub−Saharan Africa
East and southeast Asia and the Pacific
Oceania
Mean DBP 85.1 mmHg
Mean DBP 79.6 mmHg
Mean DBP 79.4 mmHg
Mean DBP 77.5 mmHg
Mean DBP 81.5 mmHg
Mean DBP 79.6 mmHg
Mean DBP 80.5 mmHg
Mean DBP 78.8 mmHg
Waist−to−height ratio
0.95
0.85
0.75
0.65
0.55
0.45
0.35
0.25
12.5
22.5
32.5
42.5
52.5
62.5
Body−mass index (kg/m2)
DBP (mmHg)
65
75
85
95
105

Appendix Figure 7: Mean systolic blood pressure (SBP) of participants who did not use anti-hypertensive medicines at different levels of waist-to-height ratio (WHtR) and body-mass index (BMI), by region.

Cells with ≤30 participants have been excluded from the figure because the results are less stable than at larger numbers. The number on each panel indicates the mean SBP among all participants who did not use anti-hypertensive medicines in each region. See Appendix Figure 27 for results using waist circumference (WC).

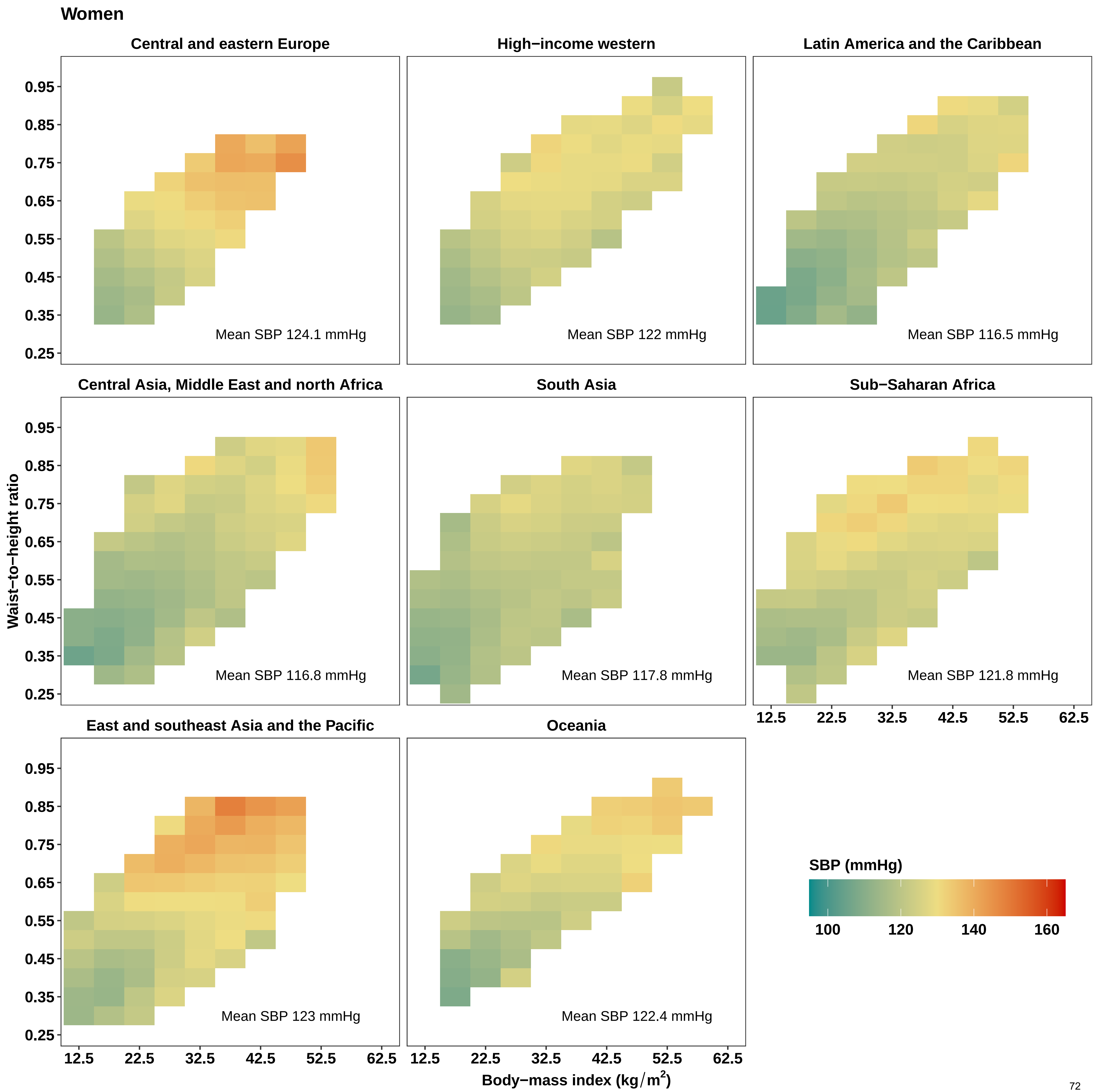
Women
Central and eastern Europe
High−income western
Latin America and the Caribbean
Central Asia, Middle East and north Africa
South Asia
Sub−Saharan Africa
East and southeast Asia and the Pacific
Oceania
Mean SBP 124.1 mmHg
Mean SBP 122 mmHg
Mean SBP 116.5 mmHg
Mean SBP 116.8 mmHg
Mean SBP 117.8 mmHg
Mean SBP 121.8 mmHg
Mean SBP 123 mmHg
Mean SBP 122.4 mmHg
Waist−to−height ratio
0.95
0.85
0.75
0.65
0.55
0.45
0.35
0.25
12.5
22.5
32.5
42.5
52.5
62.5
Body−mass index (kg/m²)
SBP (mmHg)
100
120
140
160

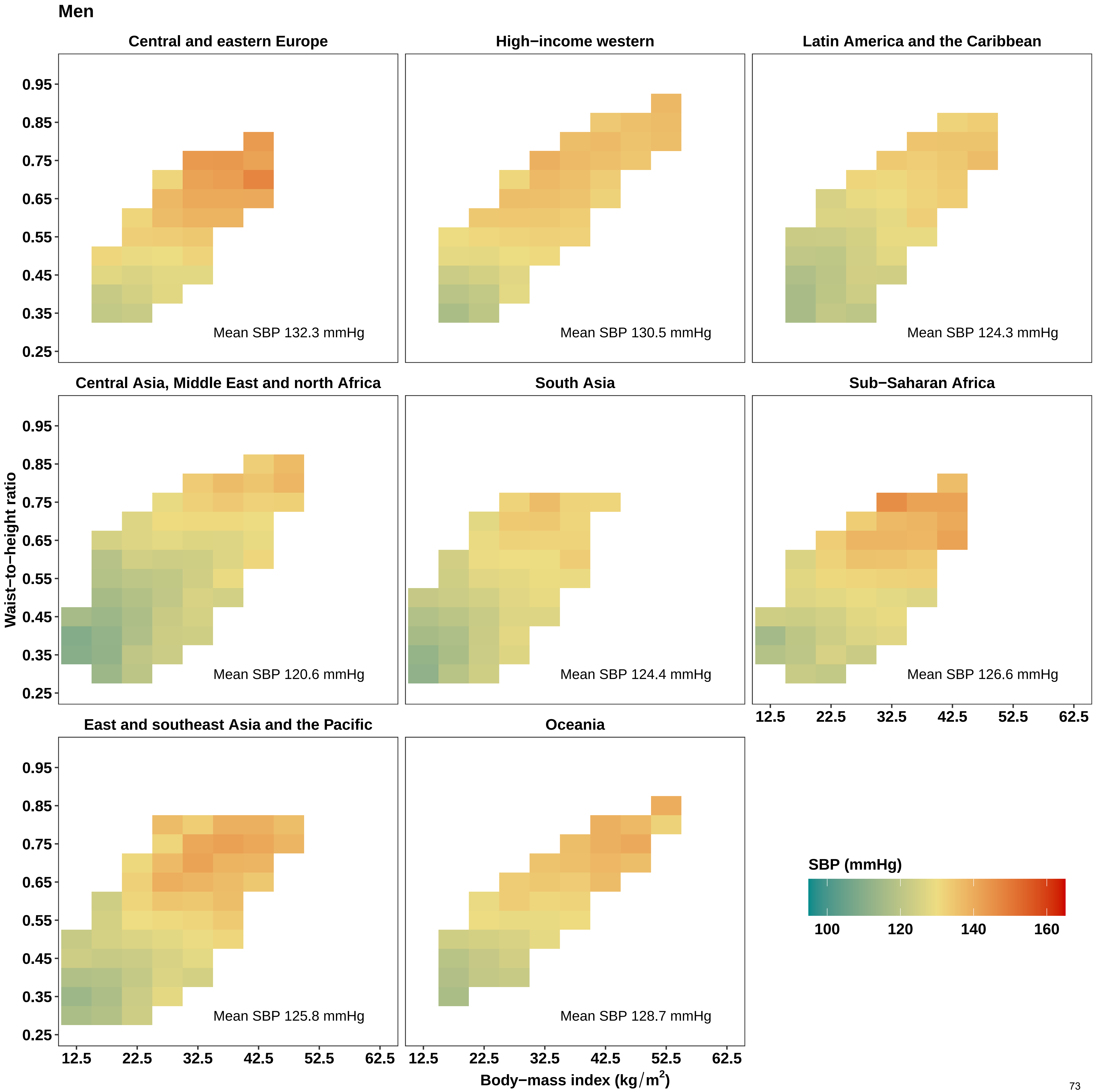
Men
Central and eastern Europe
Mean SBP 132.3 mmHg
High−income western
Mean SBP 130.5 mmHg
Latin America and the Caribbean
Mean SBP 124.3 mmHg
Central Asia, Middle East and north Africa
Mean SBP 120.6 mmHg
South Asia
Mean SBP 124.4 mmHg
Sub−Saharan Africa
Mean SBP 126.6 mmHg
East and southeast Asia and the Pacific
Mean SBP 125.8 mmHg
Oceania
Mean SBP 128.7 mmHg
Waist−to−height ratio
0.95
0.85
0.75
0.65
0.55
0.45
0.35
0.25
12.5
22.5
32.5
42.5
52.5
62.5
Body−mass index (kg/m2)
SBP (mmHg)
100
120
140
160

Appendix Figure 8: Mean diastolic blood pressure (DBP) of participants who did not use anti-hypertensive medicines at different levels of waist-to-height ratio (WHtR) and body-mass index (BMI), by region.

Cells with ≤30 participants have been excluded from the figure because the results are less stable than at larger numbers. The number on each panel indicates the mean DBP among all participants who did not use anti-hypertensive medicines in each region. See Appendix Figure 28 for results using waist circumference (WC).

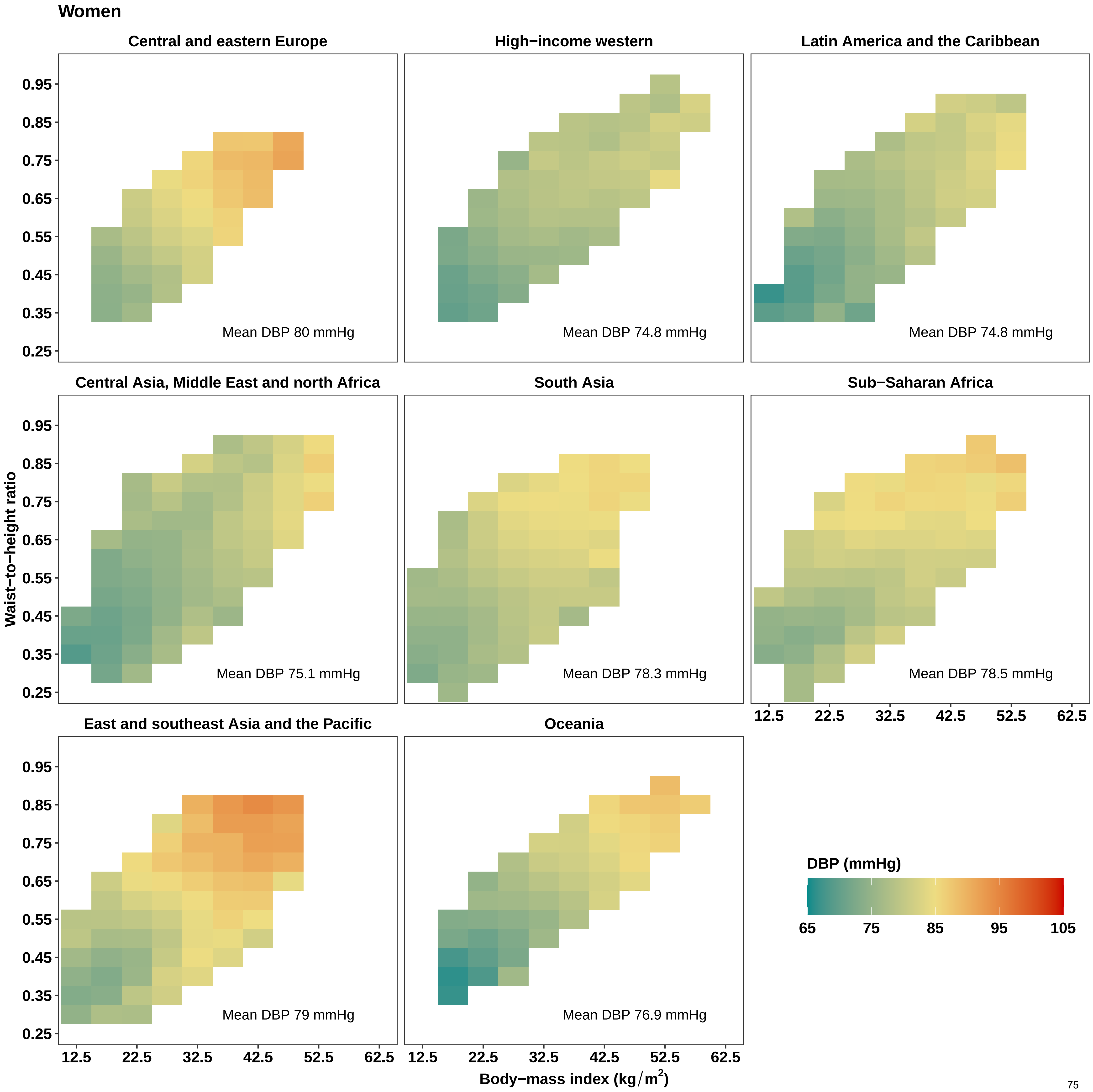
Women
Central and eastern Europe
High−income western
Latin America and the Caribbean
Central Asia, Middle East and north Africa
South Asia
Sub−Saharan Africa
East and southeast Asia and the Pacific
Oceania
Mean DBP 80 mmHg
Mean DBP 74.8 mmHg
Mean DBP 74.8 mmHg
Mean DBP 75.1 mmHg
Mean DBP 78.3 mmHg
Mean DBP 78.5 mmHg
Mean DBP 79 mmHg
Mean DBP 76.9 mmHg
Waist−to−height ratio
0.95
0.85
0.75
0.65
0.55
0.45
0.35
0.25
12.5
22.5
32.5
42.5
52.5
62.5
Body−mass index (kg/m2)
DBP (mmHg)
65
75
85
95
105

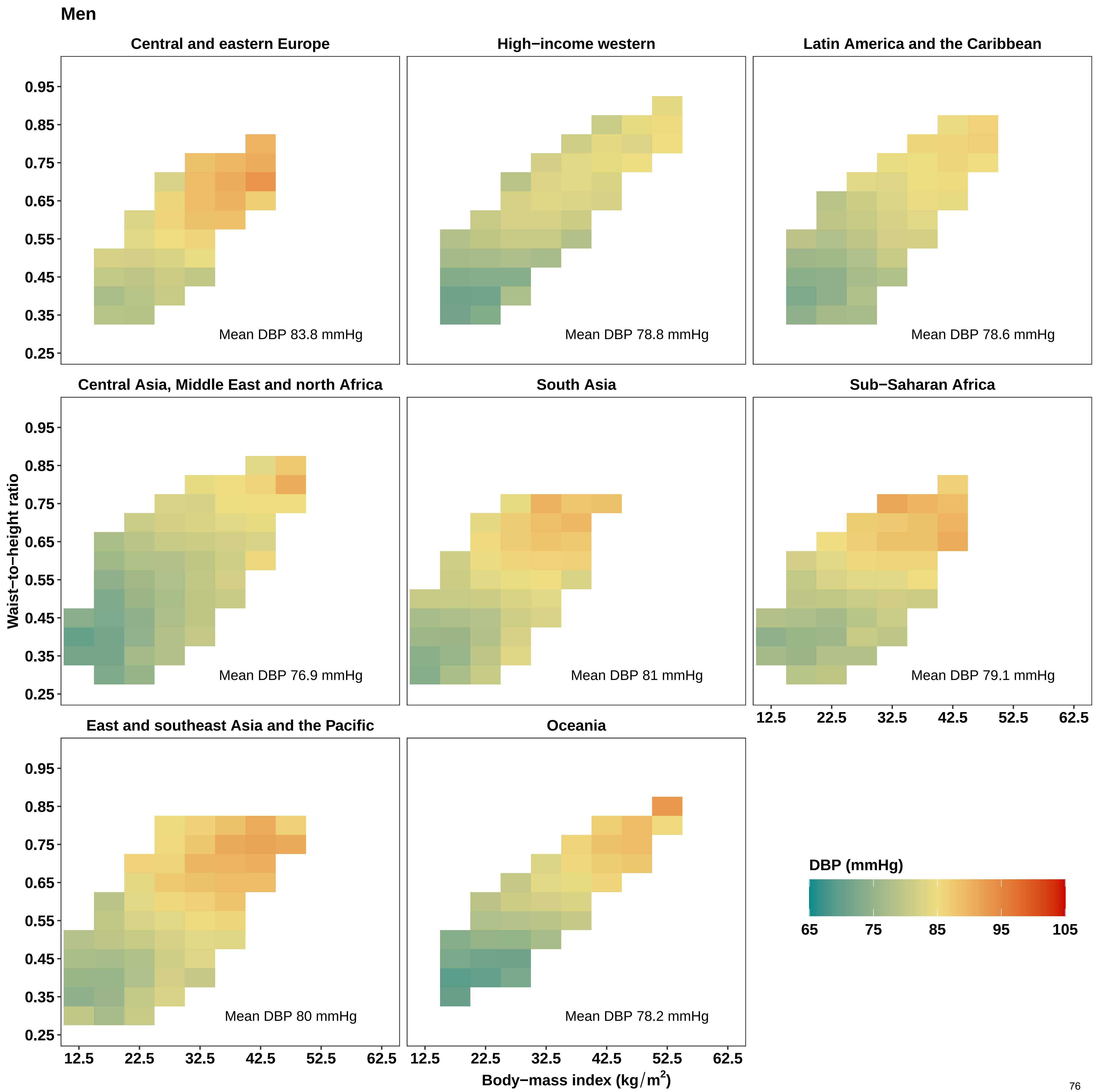

Men
Central and eastern Europe
Mean DBP 83.8 mmHg
High−income western
Mean DBP 78.8 mmHg
Latin America and the Caribbean
Mean DBP 78.6 mmHg
Central Asia, Middle East and north Africa
Mean DBP 76.9 mmHg
South Asia
Mean DBP 81 mmHg
Sub−Saharan Africa
Mean DBP 79.1 mmHg
East and southeast Asia and the Pacific
Mean DBP 80 mmHg
Oceania
Mean DBP 78.2 mmHg
Waist−to−height ratio
0.95
0.85
0.75
0.65
0.55
0.45
0.35
0.25
Body−mass index (kg/m²)
12.5
22.5
32.5
42.5
52.5
62.5
DBP (mmHg)
65
75
85
95
105

Appendix Figure 9: Mean systolic blood pressure (SBP) of participants who used anti-hypertensive medicines at different levels of waist-to-height ratio (WHtR) and body-mass index (BMI), by region.

Cells with ≤30 participants have been excluded from the figure because the results are less stable than at larger numbers. The number on each panel indicates the mean SBP among all participants who used anti-hypertensive medicines in each region. See Appendix Figure 29 for results using waist circumference (WC).

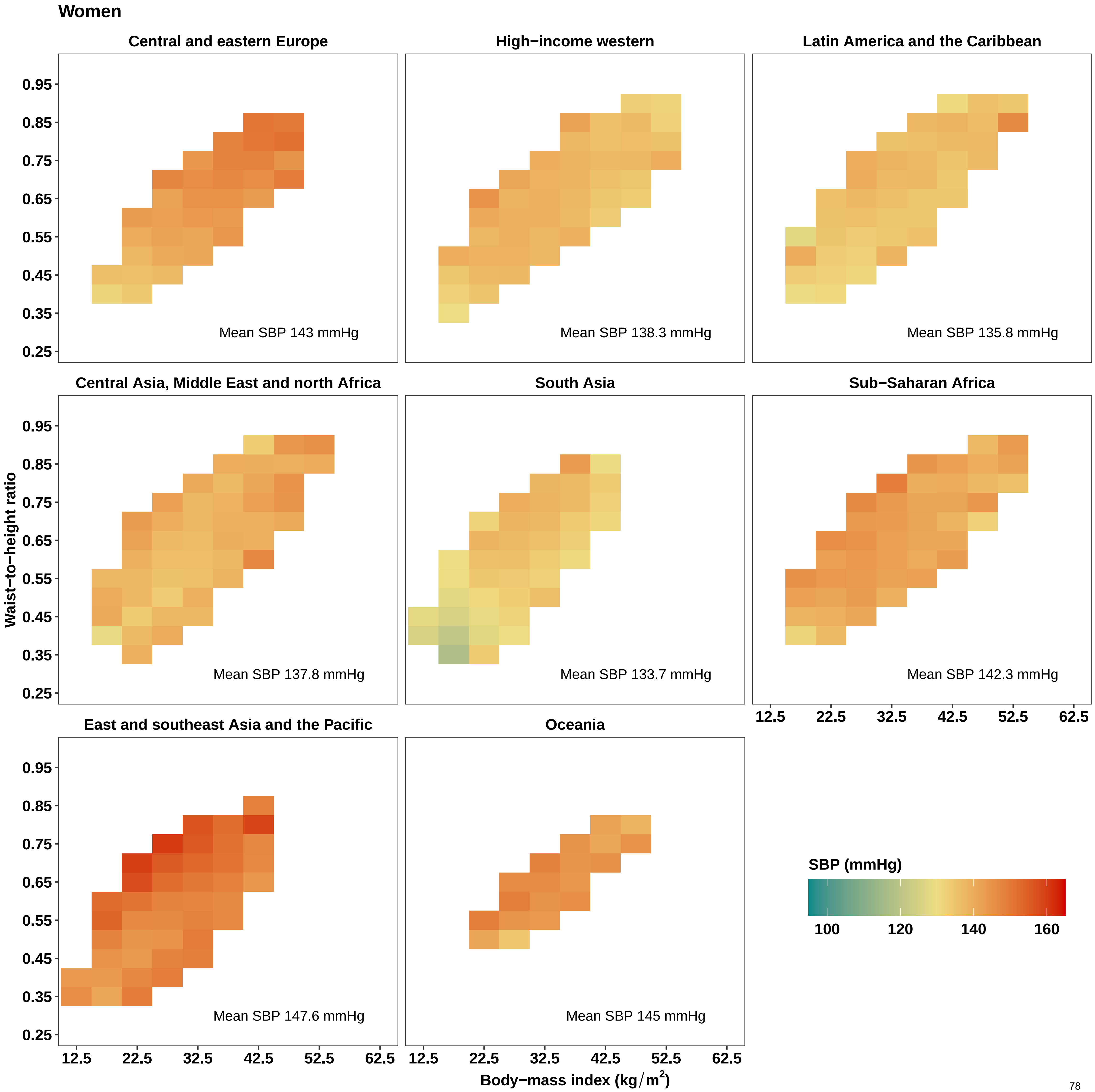

Women
Central and eastern Europe
Mean SBP 143 mmHg
High−income western
Mean SBP 138.3 mmHg
Latin America and the Caribbean
Mean SBP 135.8 mmHg
Central Asia, Middle East and north Africa
Mean SBP 137.8 mmHg
South Asia
Mean SBP 133.7 mmHg
Sub−Saharan Africa
Mean SBP 142.3 mmHg
East and southeast Asia and the Pacific
Mean SBP 147.6 mmHg
Oceania
Mean SBP 145 mmHg
Waist−to−height ratio
0.95
0.85
0.75
0.65
0.55
0.45
0.35
0.25
Body−mass index (kg/m2)
12.5
22.5
32.5
42.5
52.5
62.5
SBP (mmHg)
100
120
140
160

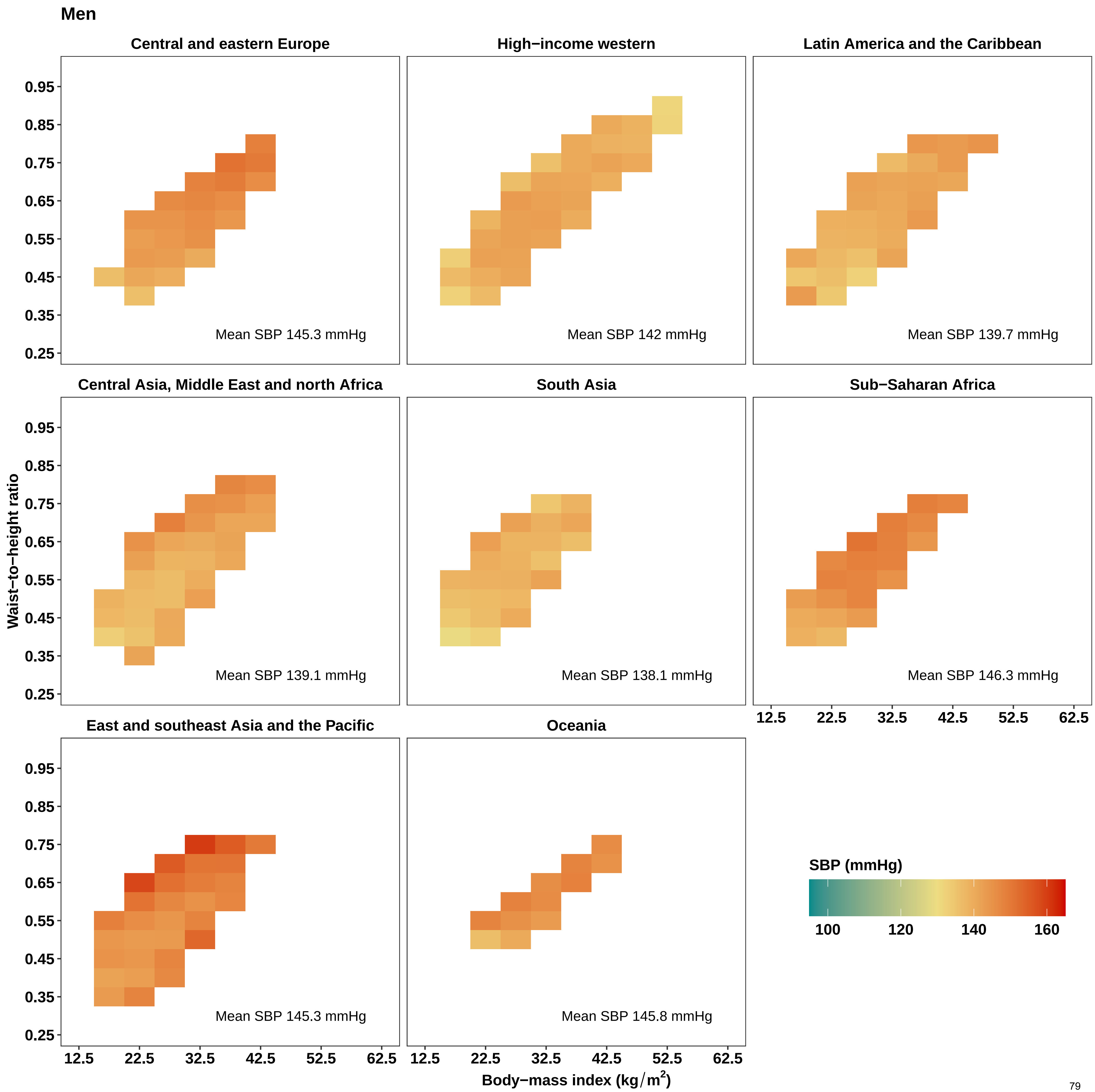
Men
Central and eastern Europe
High−income western
Latin America and the Caribbean
Central Asia, Middle East and north Africa
South Asia
Sub−Saharan Africa
East and southeast Asia and the Pacific
Oceania
Mean SBP 145.3 mmHg
Mean SBP 142 mmHg
Mean SBP 139.7 mmHg
Mean SBP 139.1 mmHg
Mean SBP 138.1 mmHg
Mean SBP 146.3 mmHg
Mean SBP 145.3 mmHg
Mean SBP 145.8 mmHg
Waist−to−height ratio
0.95
0.85
0.75
0.65
0.55
0.45
0.35
0.25
12.5
22.5
32.5
42.5
52.5
62.5
Body−mass index (kg/m2)
SBP (mmHg)
100
120
140
160

Appendix Figure 10: Mean diastolic blood pressure (DBP) of participants who used anti-hypertensive medicines at different levels of waist-to-height ratio (WHtR) and body-mass index (BMI), by region.

Cells with ≤30 participants have been excluded from the figure because the results are less stable than at larger numbers. The number on each panel indicates the mean DBP among all participants who used anti-hypertensive medicines in each region. See Appendix Figure 30 for results using waist circumference (WC).

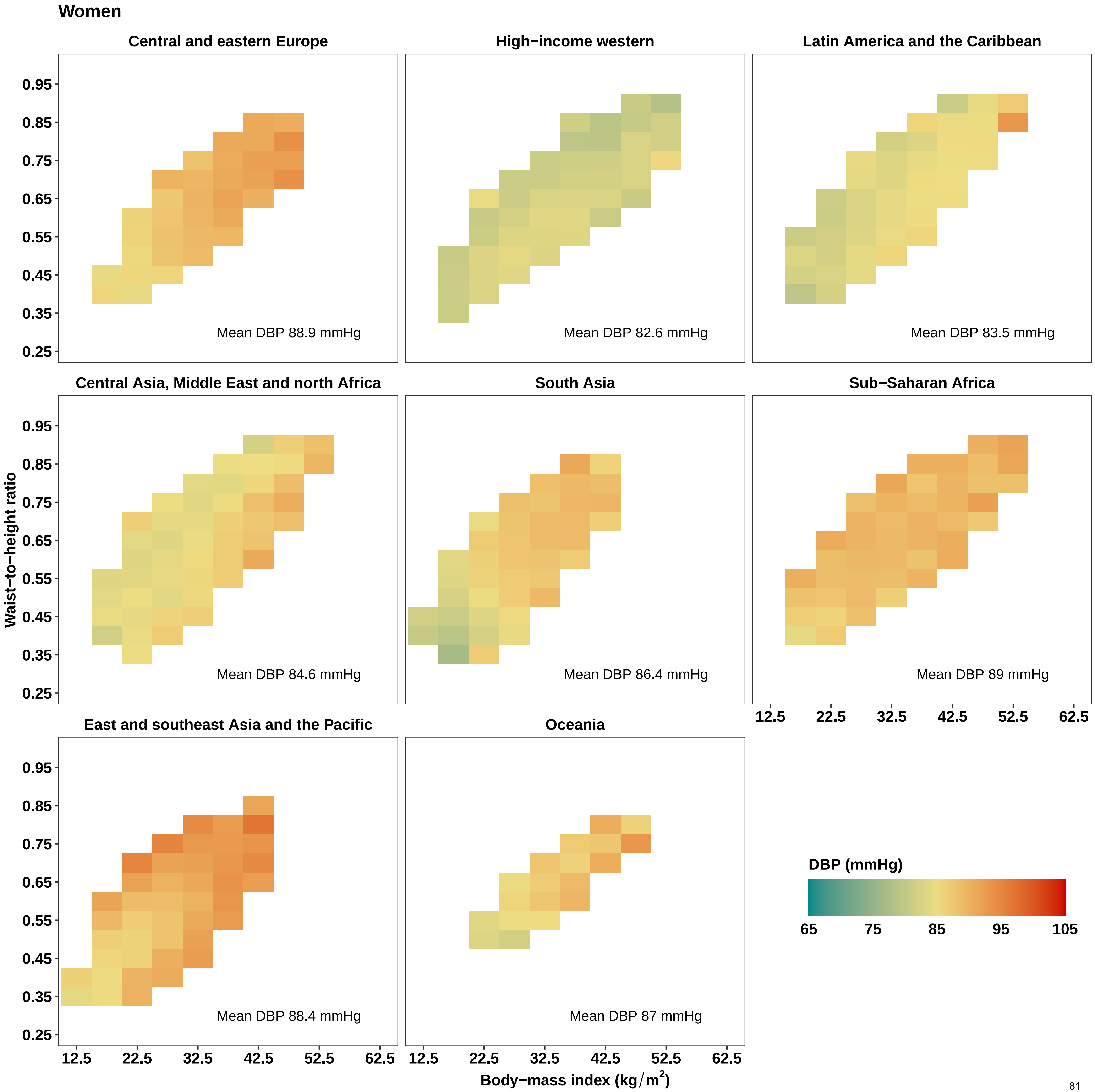
Women
Central and eastern Europe
Mean DBP 88.9 mmHg
High−income western
Mean DBP 82.6 mmHg
Latin America and the Caribbean
Mean DBP 83.5 mmHg
Central Asia, Middle East and north Africa
Mean DBP 84.6 mmHg
South Asia
Mean DBP 86.4 mmHg
Sub−Saharan Africa
Mean DBP 89 mmHg
East and southeast Asia and the Pacific
Mean DBP 88.4 mmHg
Oceania
Mean DBP 87 mmHg
Waist−to−height ratio
0.95
0.85
0.75
0.65
0.55
0.45
0.35
0.25
Body−mass index (kg/m2)
12.5
22.5
32.5
42.5
52.5
62.5
DBP (mmHg)
65
75
85
95
105

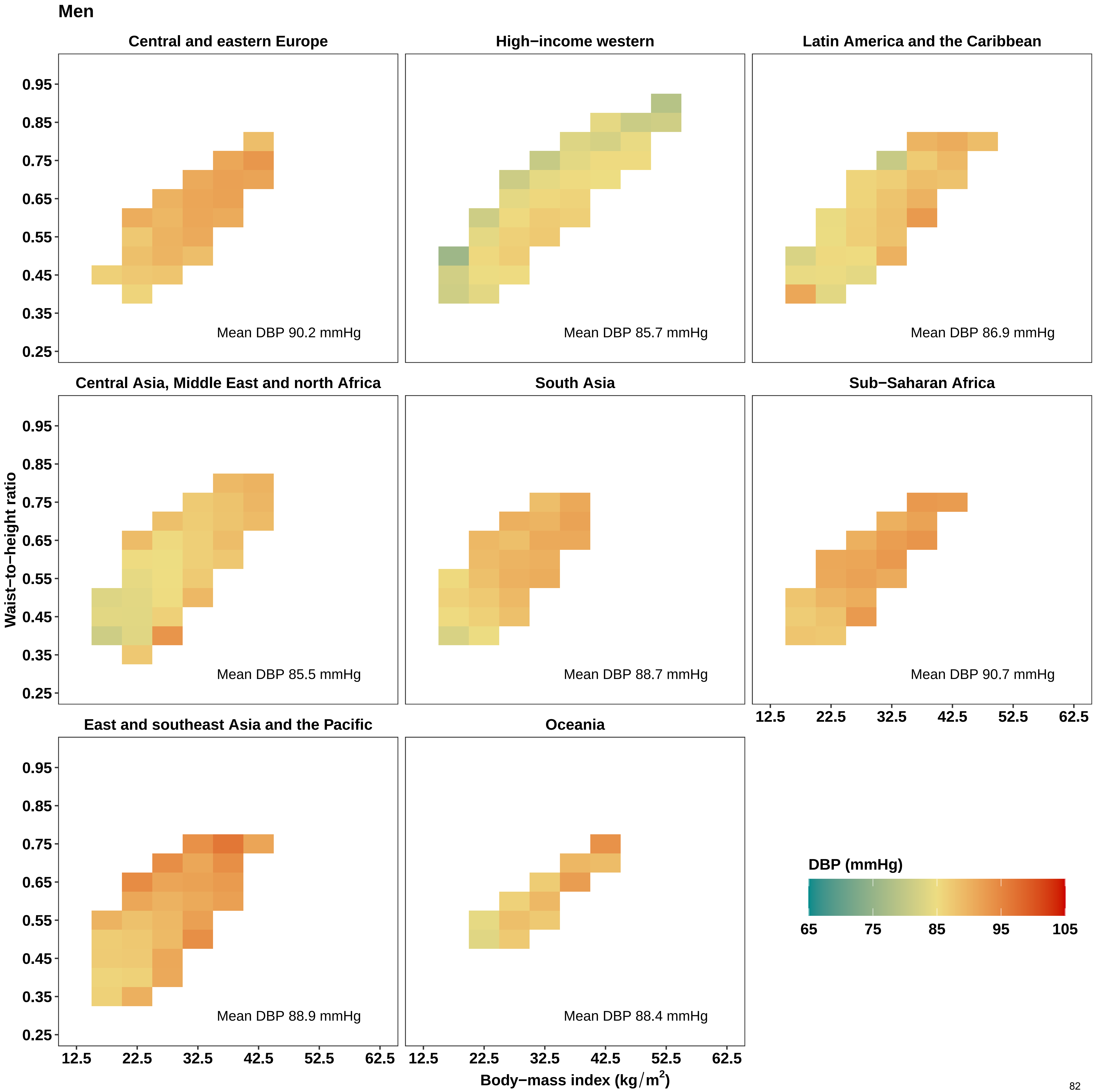
Men
Central and eastern Europe
High−income western
Latin America and the Caribbean
Central Asia, Middle East and north Africa
South Asia
Sub−Saharan Africa
East and southeast Asia and the Pacific
Oceania
Mean DBP 90.2 mmHg
Mean DBP 85.7 mmHg
Mean DBP 86.9 mmHg
Mean DBP 85.5 mmHg
Mean DBP 88.7 mmHg
Mean DBP 90.7 mmHg
Mean DBP 88.9 mmHg
Mean DBP 88.4 mmHg
Waist−to−height ratio
0.95 0.85 0.75 0.65 0.55 0.45 0.35 0.25
Body−mass index (kg/m²)
12.5 22.5 32.5 42.5 52.5 62.5
DBP (mmHg)
65 75 85 95 105

Appendix Figure 11: Odds ratio (OR) for prevalent hypertension per standard deviation (SD) of body-mass index (BMI) and of waist-to-height ratio (WHtR), with and without mutual adjustment.

In each panel, the upper point shows OR without adjustment for the 2nd adiposity index and the lower point shows OR with adjustment for the 2nd adiposity index. See Appendix Figure 31 for results using waist circumference (WC).

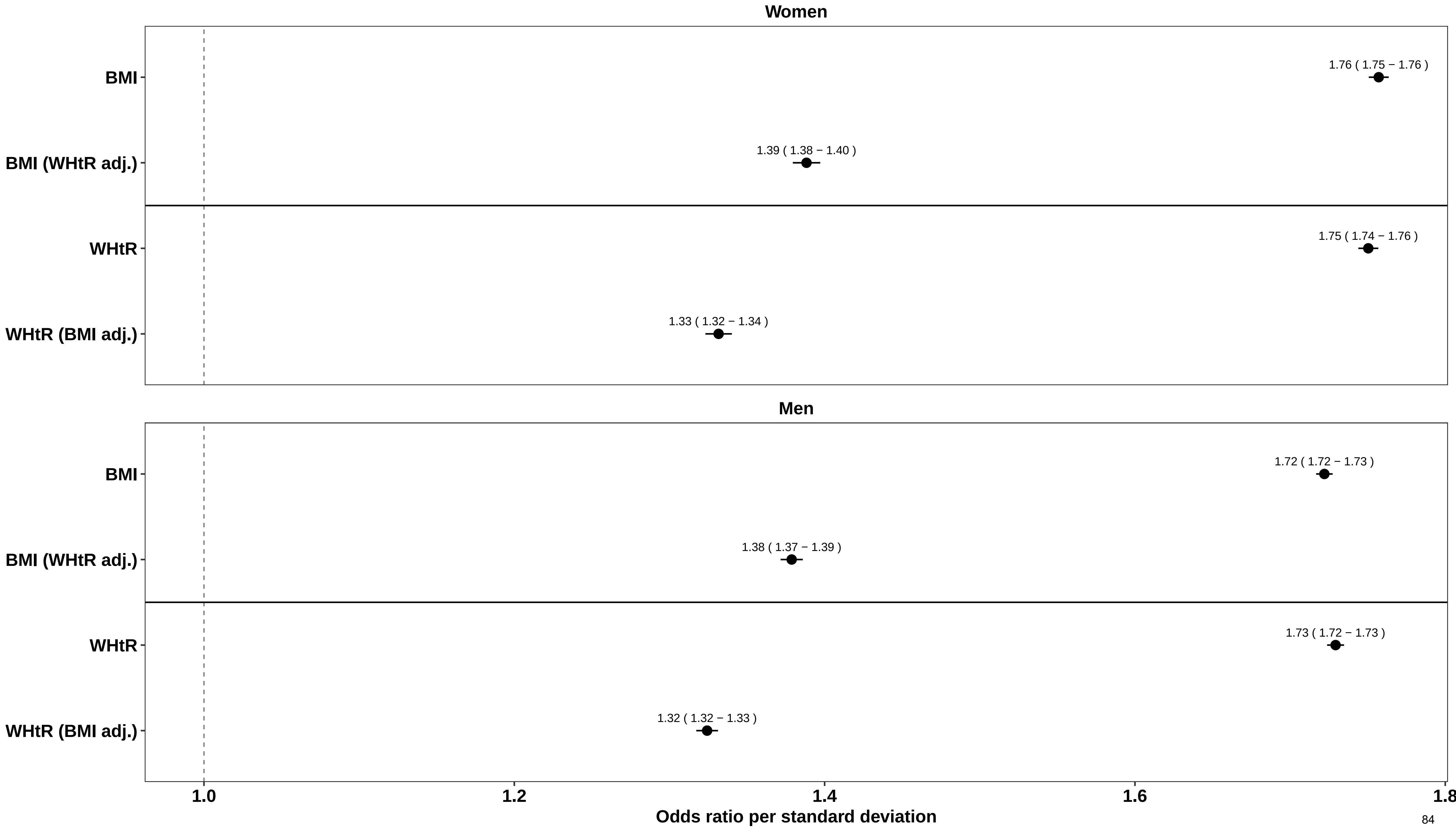
Women
BMI
BMI (WHtR adj.)
WHtR
WHtR (BMI adj.)
1.76 (1.75 − 1.76)
1.39 (1.38 − 1.40)
1.75 (1.74 − 1.76)
1.33 (1.32 − 1.34)
Men
BMI
BMI (WHtR adj.)
WHtR
WHtR (BMI adj.)
1.72 (1.72 − 1.73)
1.38 (1.37 − 1.39)
1.73 (1.72 − 1.73)
1.32 (1.32 − 1.33)
1.0
1.2
1.4
1.6
1.8
Odds ratio per standard deviation

Appendix Figure 12: Relationship between waist circumference (WC) and body-mass index (BMI), by region.

The shading indicates the density of participants in each region, with darker shades corresponding to more participants and vice versa. The numbers on the panels indicate the Pearson correlation coefficient between BMI and WC in each region. The vertical and horizontal lines show median BMI and WC, respectively, for all participants.

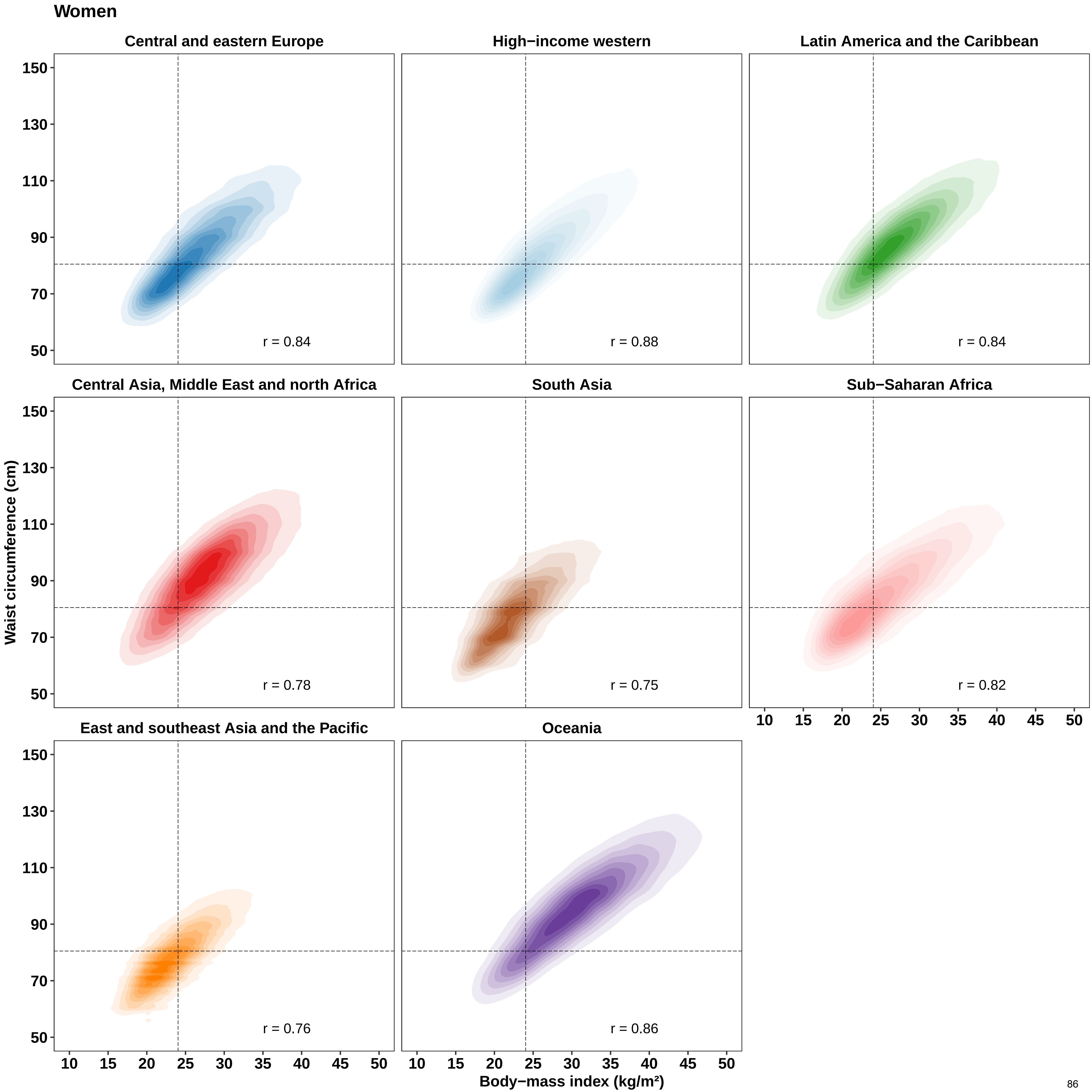

Women
Central and eastern Europe
r = 0.84
High−income western
r = 0.88
Latin America and the Caribbean
r = 0.84
Central Asia, Middle East and north Africa
r = 0.78
South Asia
r = 0.75
Sub−Saharan Africa
r = 0.82
East and southeast Asia and the Pacific
r = 0.76
Oceania
r = 0.86
Waist circumference (cm)
Body−mass index (kg/m²)

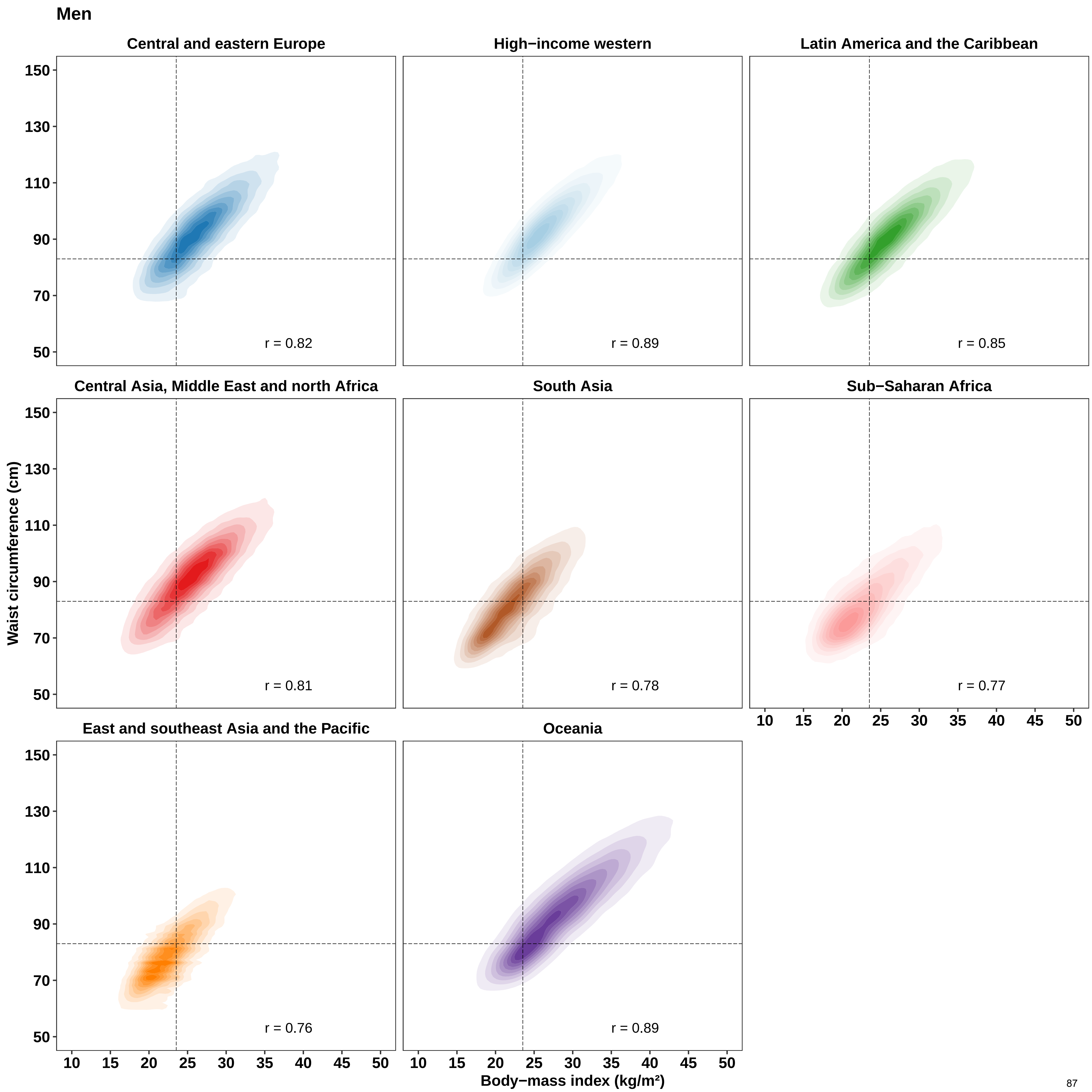
Men
Central and eastern Europe
High−income western
Latin America and the Caribbean
Central Asia, Middle East and north Africa
South Asia
Sub−Saharan Africa
East and southeast Asia and the Pacific
Oceania
r = 0.82
r = 0.89
r = 0.85
r = 0.81
r = 0.78
r = 0.77
r = 0.76
r = 0.89
Waist circumference (cm)
Body−mass index (kg/m²)
50
70
90
110
130
150
10
15
20
25
30
35
40
45
50

Appendix Figure 13: Waist circumference (WC) adjusted for body-mass index (BMI) and age, by region.

The graph shows mean regional WC for the global mean (across all participants) of BMI (25.0 kg/m^2 for women and 24.2 kg/m^2 for men) and age (41.3 years for women and 42.0 years for men).

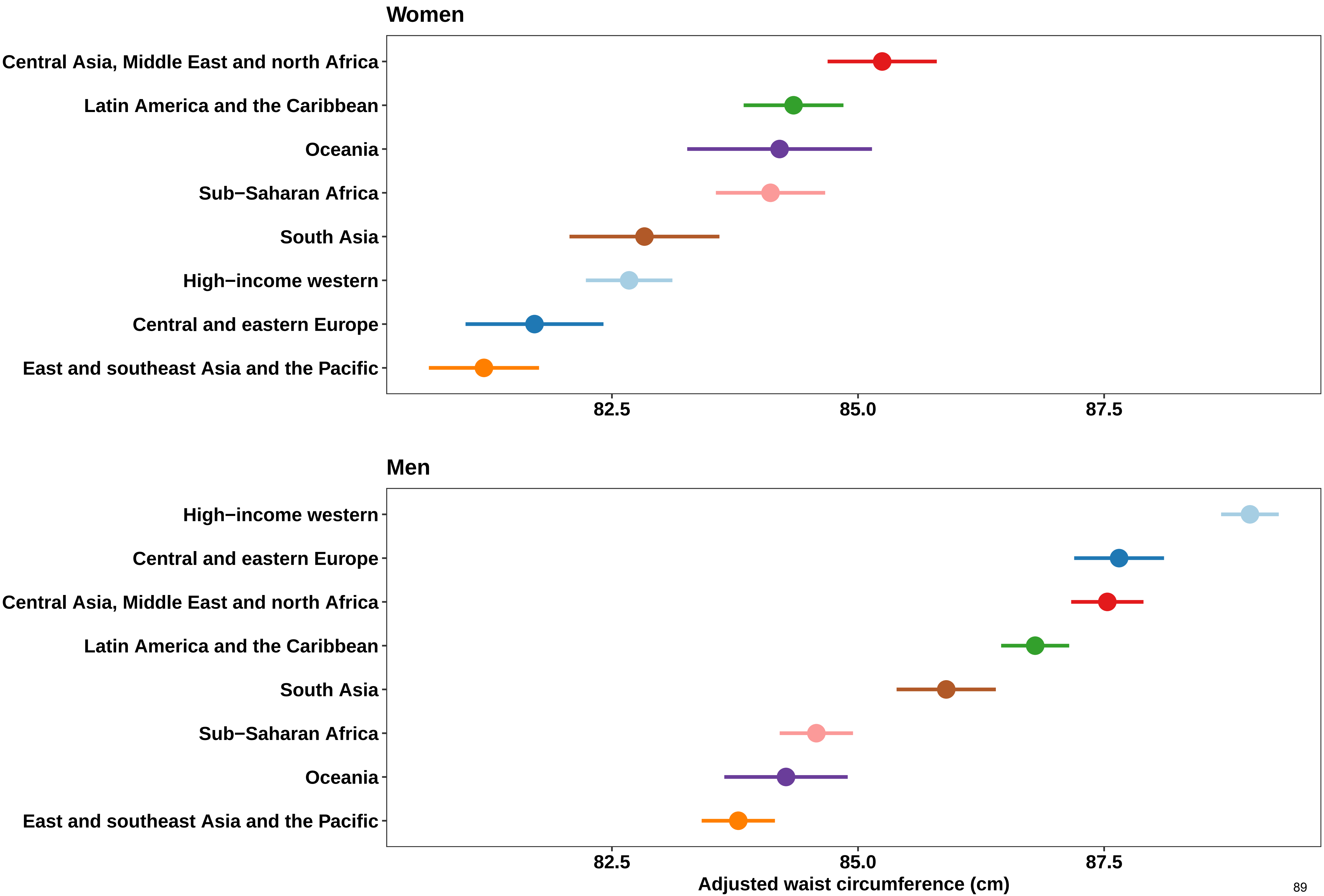
Women
Central Asia, Middle East and north Africa
Latin America and the Caribbean
Oceania
Sub−Saharan Africa
South Asia
High−income western
Central and eastern Europe
East and southeast Asia and the Pacific
82.5
85.0
87.5
Men
High−income western
Central and eastern Europe
Central Asia, Middle East and north Africa
Latin America and the Caribbean
South Asia
Sub−Saharan Africa
Oceania
East and southeast Asia and the Pacific
82.5
85.0
87.5
Adjusted waist circumference (cm)

Appendix Figure 14: Regional body-mass index (BMI) adjustment.

The BMI adjustment shows how much lower BMI in each region should be to achieve an equivalent waist circumference (WC). The adjustment is shown relative to the population of the high-income western region where most current epidemiological studies have been done; regional ordering and differences across regions would be unchanged if a different reference were used.

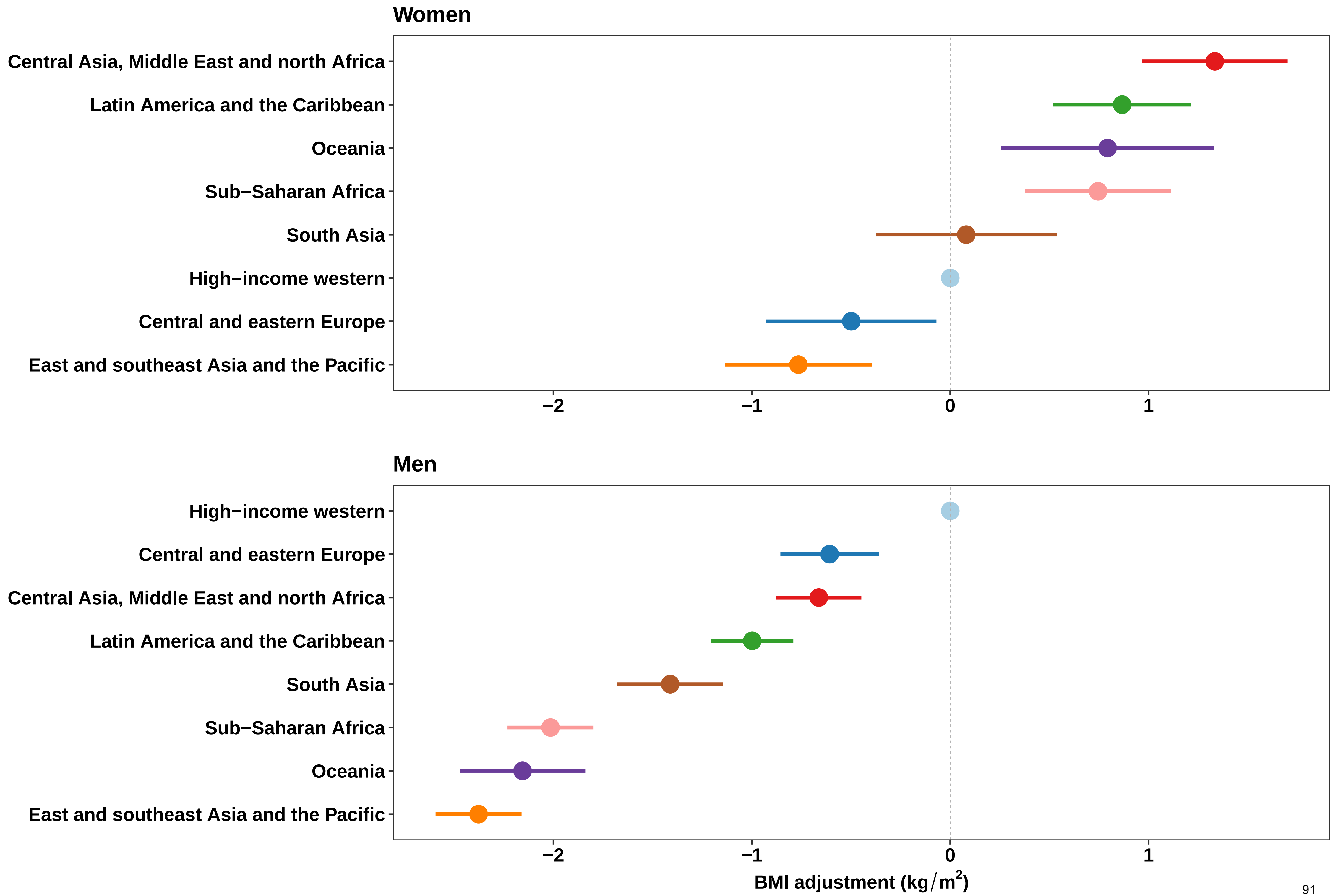
Women
Central Asia, Middle East and north Africa
Latin America and the Caribbean
Oceania
Sub−Saharan Africa
South Asia
High−income western
Central and eastern Europe
East and southeast Asia and the Pacific
−2
−1
0
1
Men
High−income western
Central and eastern Europe
Central Asia, Middle East and north Africa
Latin America and the Caribbean
South Asia
Sub−Saharan Africa
Oceania
East and southeast Asia and the Pacific
−2
−1
0
1
BMI adjustment (kg/m2)

Appendix Figure 15: Prevalence of hypertension at different levels of waist circumference (WC) and body-mass index (BMI), by region.

Cells with ≤30 participants have been excluded from the figure because the results are less stable than at larger numbers. The number on each panel indicates the crude prevalence of hypertension among all participants in each region. See Appendix Figures 19-20 for separate results for untreated and treated hypertension.

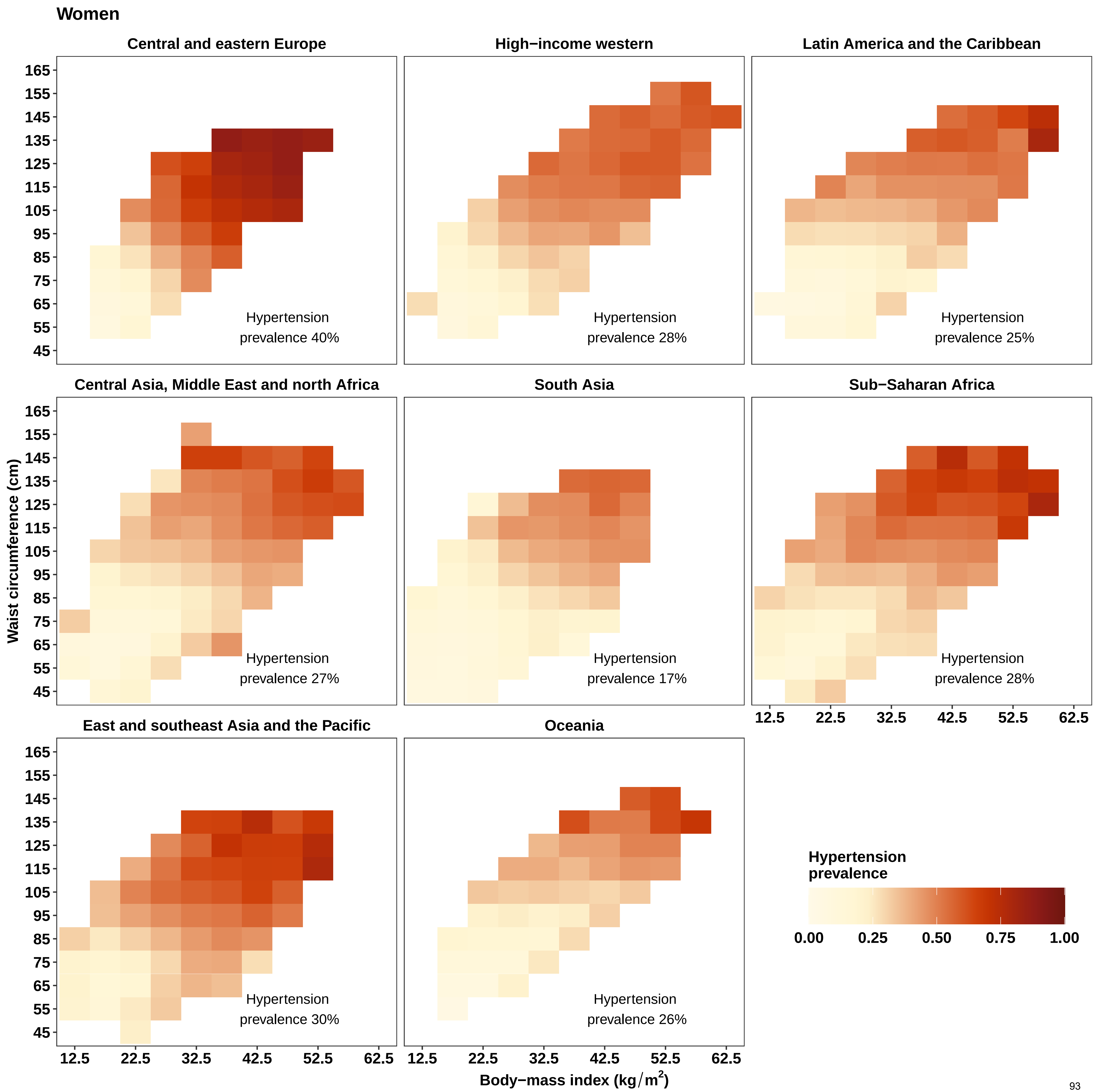

Women
Central and eastern Europe
High−income western
Latin America and the Caribbean
Central Asia, Middle East and north Africa
South Asia
Sub−Saharan Africa
East and southeast Asia and the Pacific
Oceania
Hypertension prevalence 40%
Hypertension prevalence 28%
Hypertension prevalence 25%
Hypertension prevalence 27%
Hypertension prevalence 17%
Hypertension prevalence 28%
Hypertension prevalence 30%
Hypertension prevalence 26%
Waist circumference (cm)
165 155 145 135 125 115 105 95 85 75 65 55 45
12.5 22.5 32.5 42.5 52.5 62.5
Body−mass index (kg/m2)
Hypertension prevalence
0.00 0.25 0.50 0.75 1.00

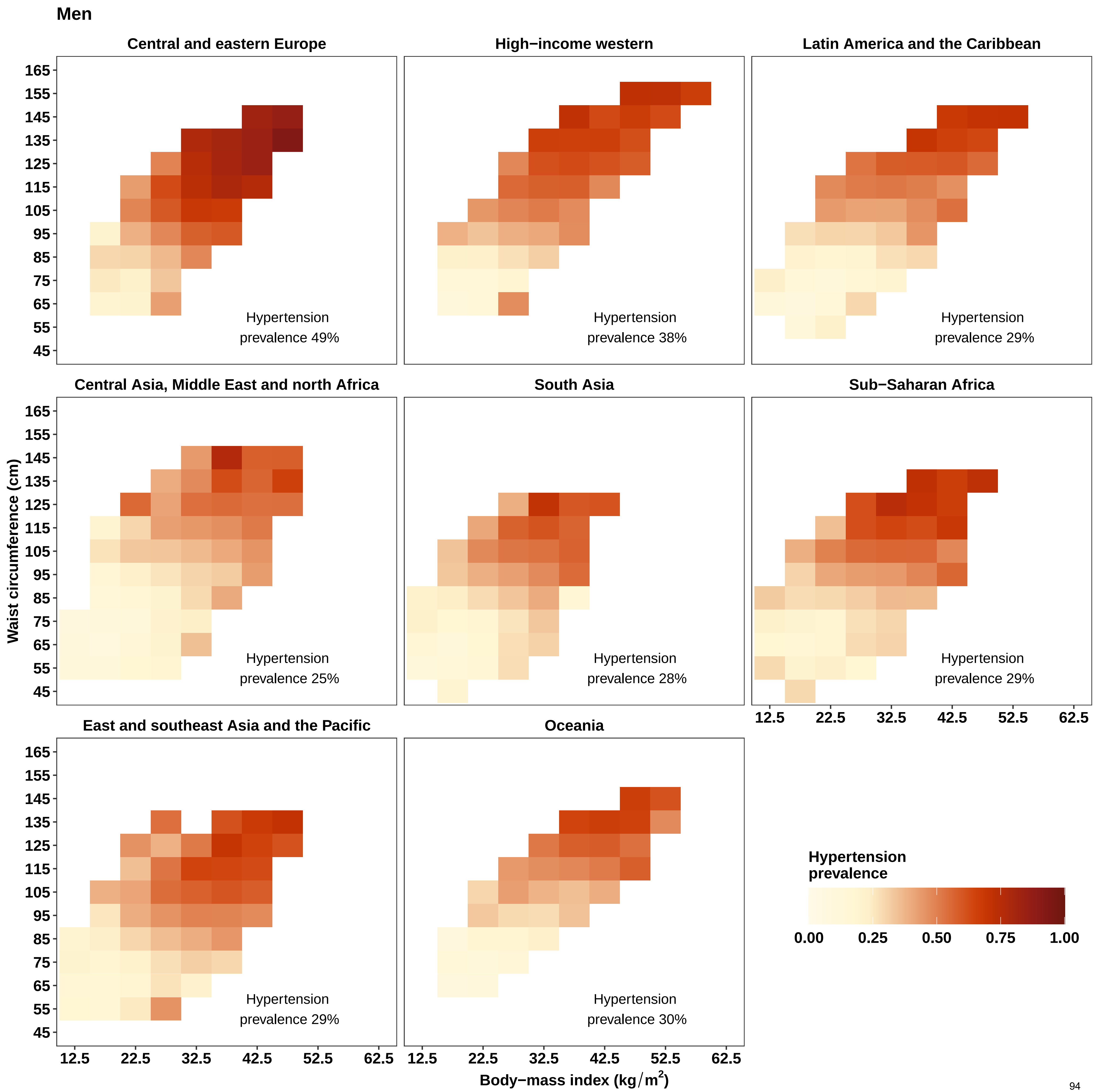

Men
Central and eastern Europe
High−income western
Latin America and the Caribbean
Central Asia, Middle East and north Africa
South Asia
Sub−Saharan Africa
East and southeast Asia and the Pacific
Oceania
Hypertension prevalence 49%
Hypertension prevalence 38%
Hypertension prevalence 29%
Hypertension prevalence 25%
Hypertension prevalence 28%
Hypertension prevalence 29%
Hypertension prevalence 29%
Hypertension prevalence 30%
Waist circumference (cm)
45 55 65 75 85 95 105 115 125 135 145 155 165
12.5 22.5 32.5 42.5 52.5 62.5
Body−mass index (kg/m²)
Hypertension prevalence
0.00 0.25 0.50 0.75 1.00

Appendix Figure 16: Distribution of participants with hypertension in relation to body-mass index (BMI) and waist circumference (WC), by region.

The shading indicates the density of participants with hypertension in each region, with darker shades corresponding to more participants. The vertical and horizontal lines show median BMI and WC, respectively, for all participants (black-dashed) and those with hypertension (grey-solid) globally. See Appendix Figures 23-24 for separate results for untreated and treated hypertension.

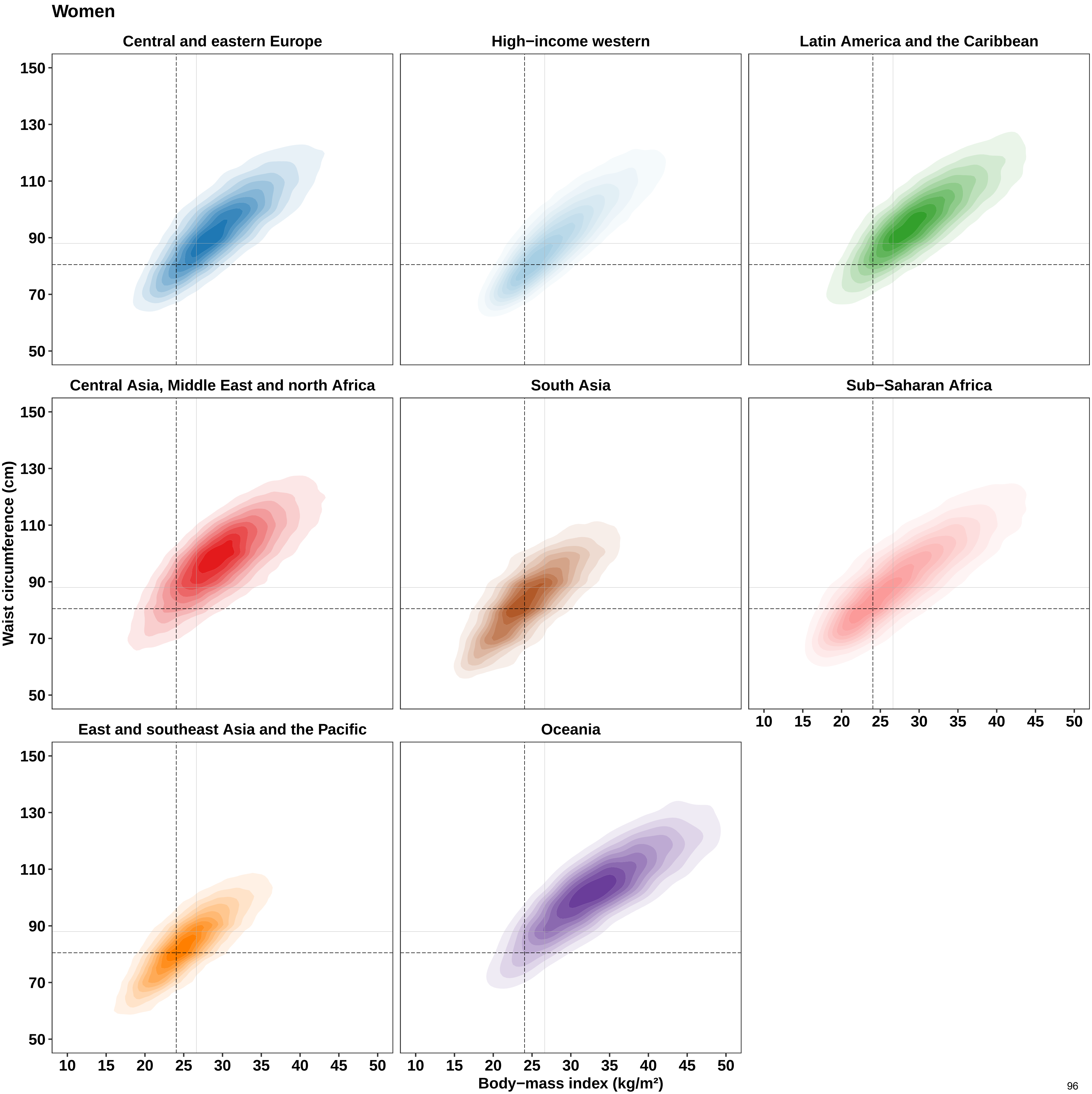

Women
Central and eastern Europe
High−income western
Latin America and the Caribbean
Central Asia, Middle East and north Africa
South Asia
Sub−Saharan Africa
East and southeast Asia and the Pacific
Oceania
Waist circumference (cm)
Body−mass index (kg/m²)
150
130
110
90
70
50
10
15
20
25
30
35
40
45
50

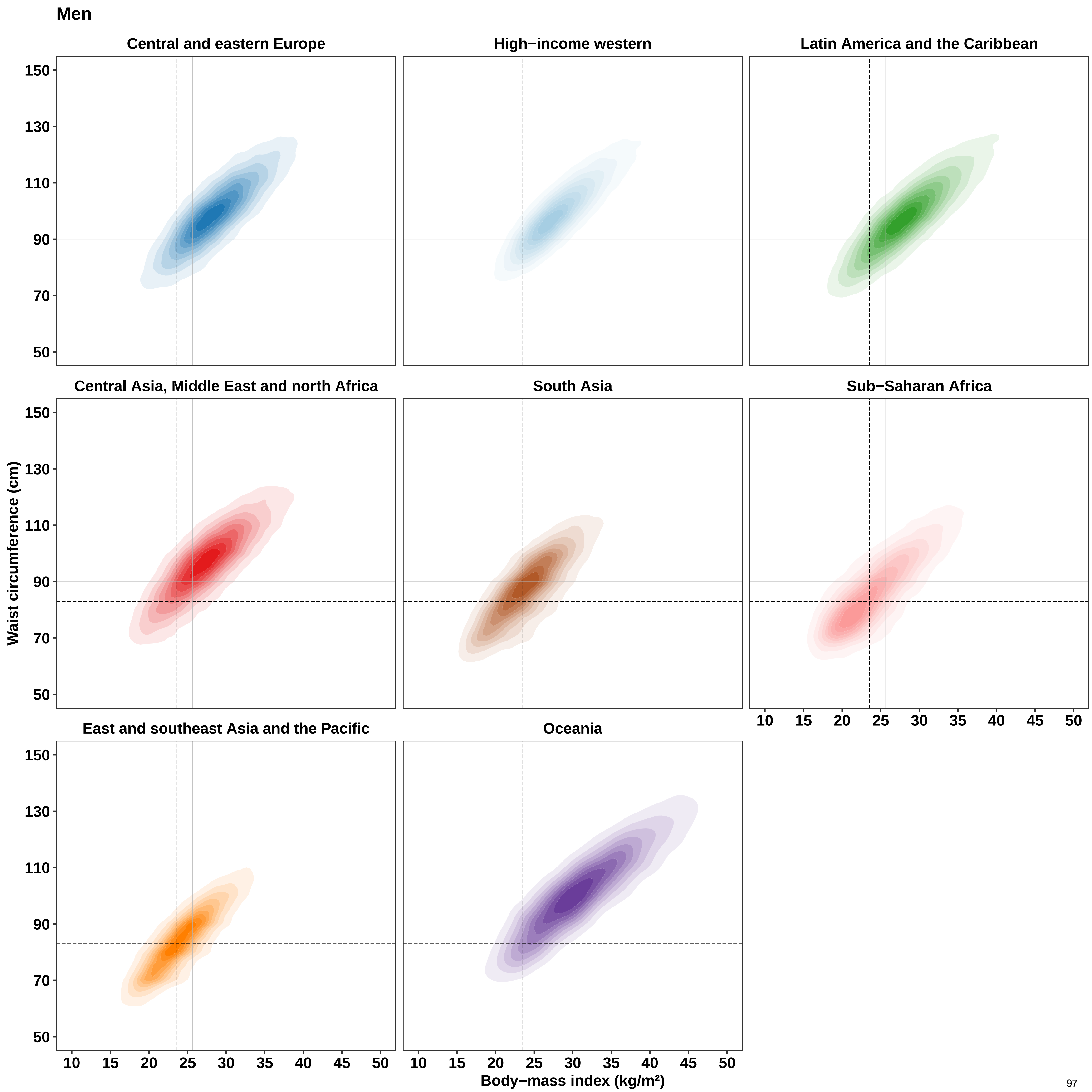

Men
Central and eastern Europe
High−income western
Latin America and the Caribbean
Central Asia, Middle East and north Africa
South Asia
Sub−Saharan Africa
East and southeast Asia and the Pacific
Oceania
Waist circumference (cm)
Body−mass index (kg/m²)
150
130
110
90
70
50
10
15
20
25
30
35
40
45
50

Appendix Figure 17: Prevalence of participants with hypertension who did not use anti-hypertensive medicines at different levels of waist-to-height ratio (WHtR) and body-mass index (BMI), by region.

Cells with ≤30 participants have been excluded from the figure because the results are less stable than at larger numbers. The number on each panel indicates the crude prevalence of participants with hypertension who did not use anti-hypertensive medicines among all participants in each region. See Appendix Figure 19 for results using waist circumference (WC).

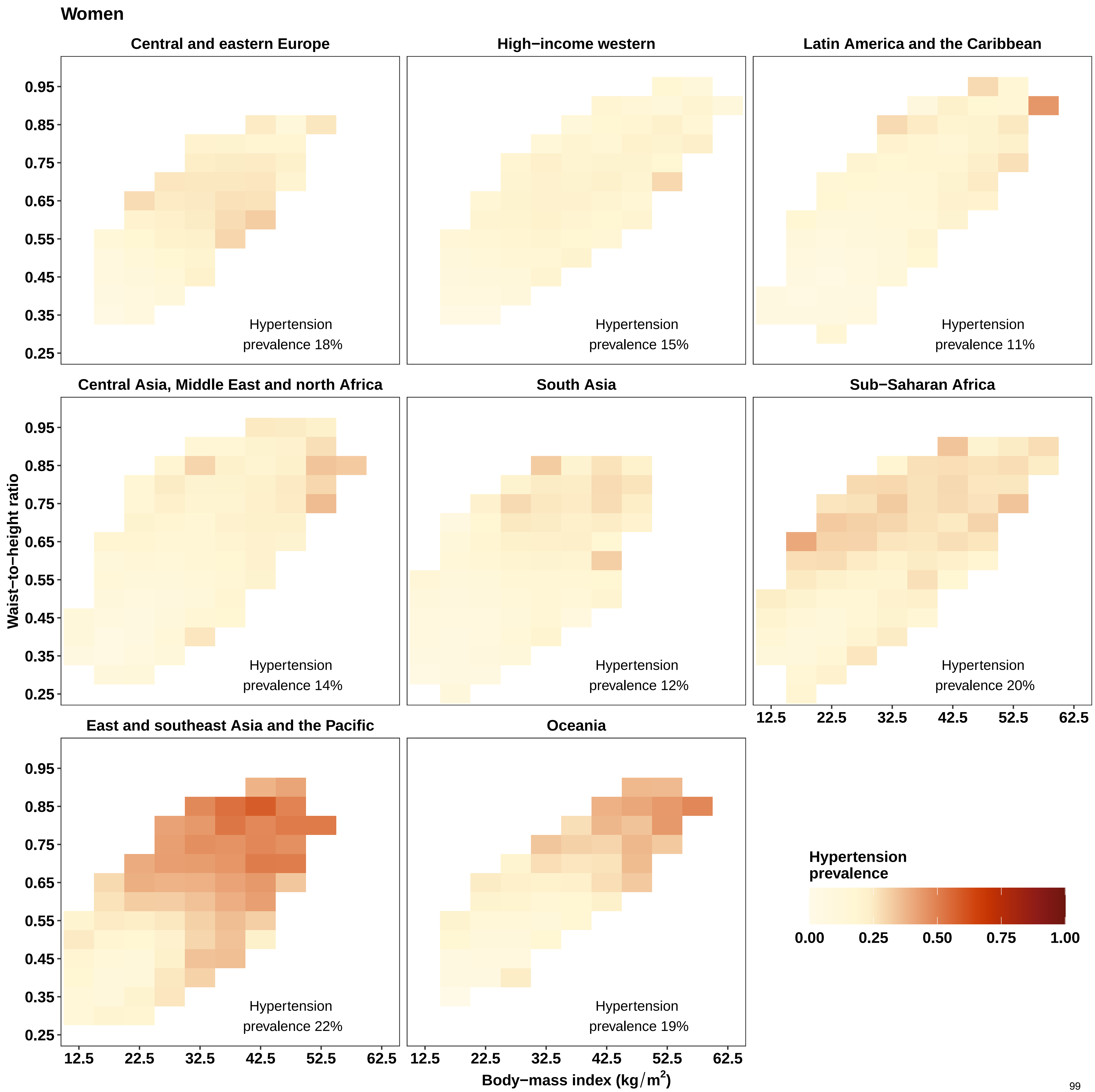

Women
Central and eastern Europe
Hypertension prevalence 18%
High−income western
Hypertension prevalence 15%
Latin America and the Caribbean
Hypertension prevalence 11%
Central Asia, Middle East and north Africa
Hypertension prevalence 14%
South Asia
Hypertension prevalence 12%
Sub−Saharan Africa
Hypertension prevalence 20%
East and southeast Asia and the Pacific
Hypertension prevalence 22%
Oceania
Hypertension prevalence 19%
Waist−to−height ratio
0.95
0.85
0.75
0.65
0.55
0.45
0.35
0.25
12.5
22.5
32.5
42.5
52.5
62.5
Body−mass index (kg/m2)
Hypertension prevalence
0.00
0.25
0.50
0.75
1.00

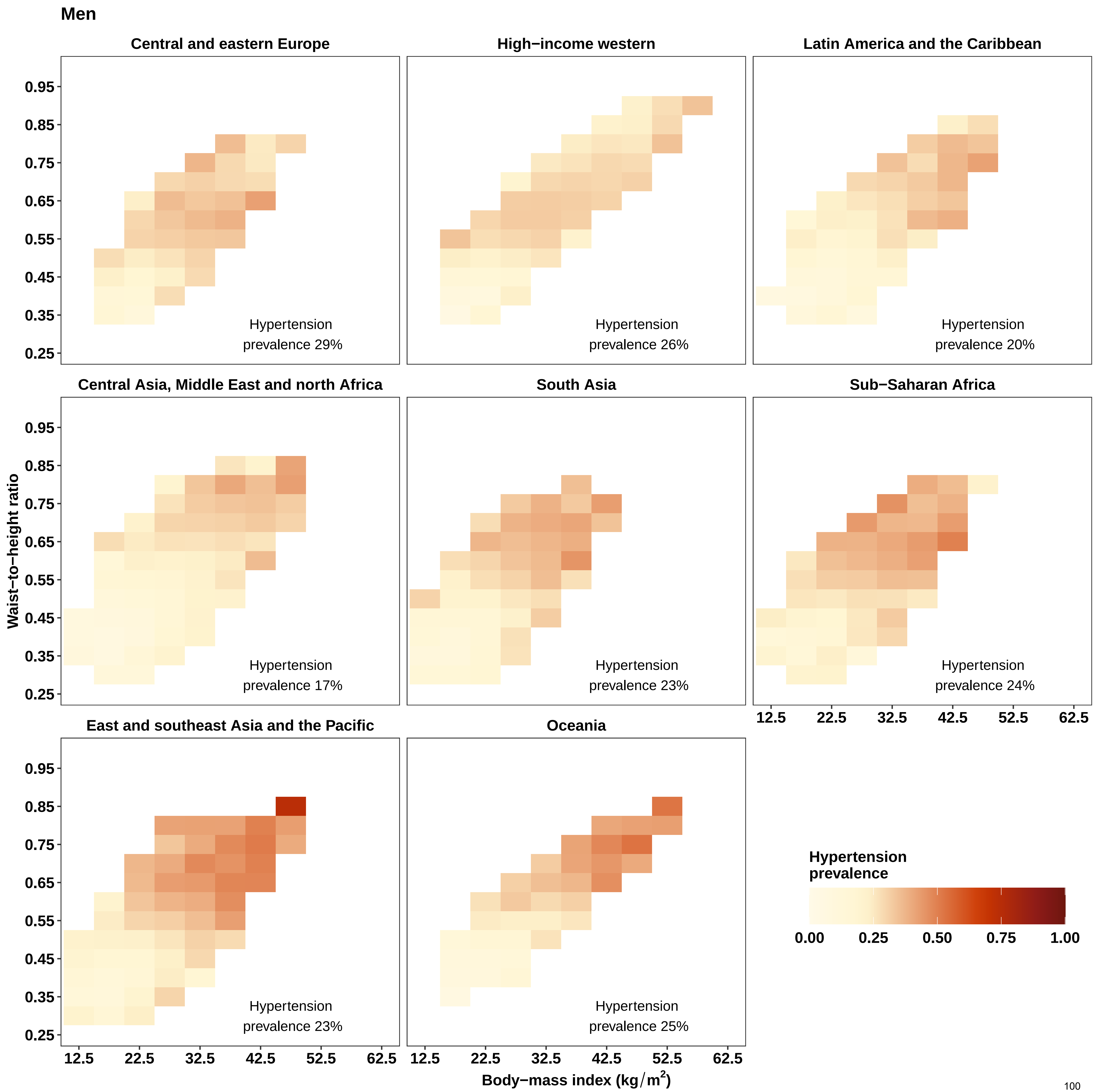
Men
Central and eastern Europe
High−income western
Latin America and the Caribbean
Central Asia, Middle East and north Africa
South Asia
Sub−Saharan Africa
East and southeast Asia and the Pacific
Oceania
Hypertension prevalence 29%
Hypertension prevalence 26%
Hypertension prevalence 20%
Hypertension prevalence 17%
Hypertension prevalence 23%
Hypertension prevalence 24%
Hypertension prevalence 23%
Hypertension prevalence 25%
Waist−to−height ratio
0.95
0.85
0.75
0.65
0.55
0.45
0.35
0.25
Body−mass index (kg/m²)
12.5
22.5
32.5
42.5
52.5
62.5
Hypertension prevalence
0.00
0.25
0.50
0.75
1.00

Appendix Figure 18: Prevalence of participants with hypertension who used anti-hypertensive medicines at different levels of waist-to-height ratio (WHtR) and body-mass index (BMI), by region.

Cells with ≤30 participants have been excluded from the figure because the results are less stable than at larger numbers. The number on each panel indicates the crude prevalence of participants with hypertension who used anti-hypertensive medicines among all participants in each region. See Appendix Figure 20 for results using waist circumference (WC).

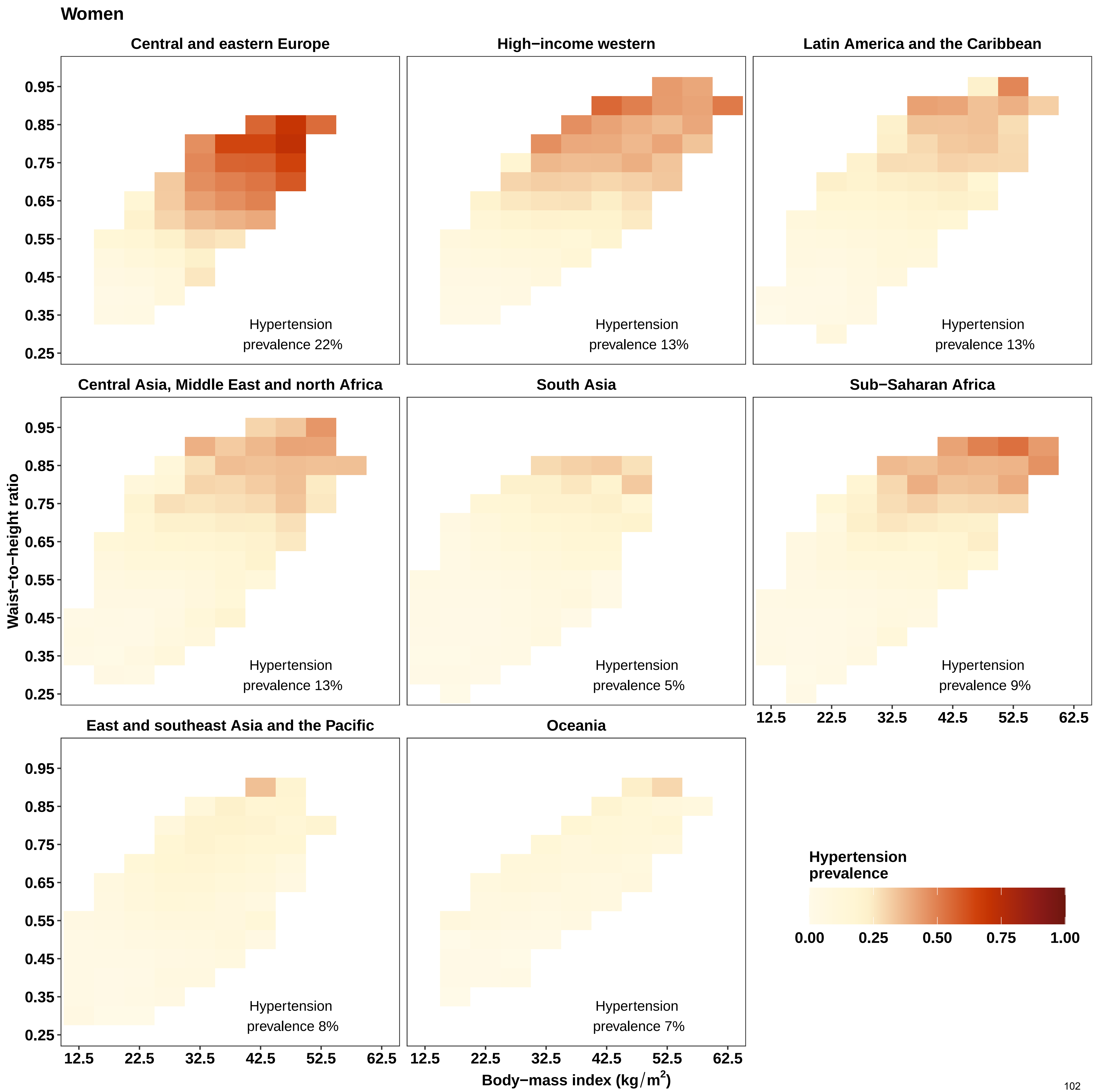
Women
Central and eastern Europe
High−income western
Latin America and the Caribbean
Central Asia, Middle East and north Africa
South Asia
Sub−Saharan Africa
East and southeast Asia and the Pacific
Oceania
Hypertension prevalence 22%
Hypertension prevalence 13%
Hypertension prevalence 13%
Hypertension prevalence 13%
Hypertension prevalence 5%
Hypertension prevalence 9%
Hypertension prevalence 8%
Hypertension prevalence 7%
Waist−to−height ratio
0.95
0.85
0.75
0.65
0.55
0.45
0.35
0.25
Body−mass index (kg/m2)
12.5
22.5
32.5
42.5
52.5
62.5
Hypertension prevalence
0.00
0.25
0.50
0.75
1.00

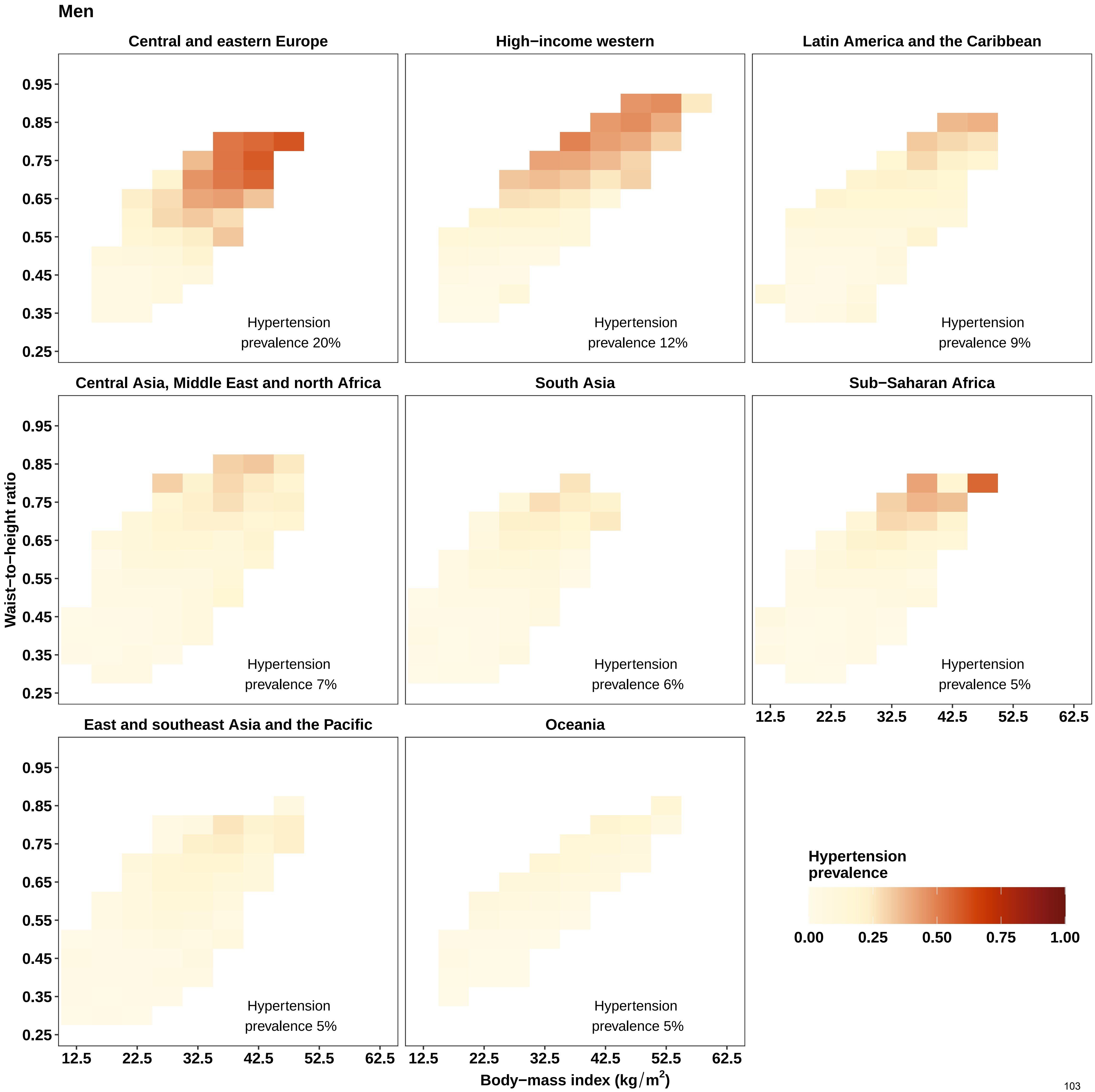
Men
Central and eastern Europe
High−income western
Latin America and the Caribbean
Central Asia, Middle East and north Africa
South Asia
Sub−Saharan Africa
East and southeast Asia and the Pacific
Oceania
Hypertension prevalence 20%
Hypertension prevalence 12%
Hypertension prevalence 9%
Hypertension prevalence 7%
Hypertension prevalence 6%
Hypertension prevalence 5%
Hypertension prevalence 5%
Hypertension prevalence 5%
Waist−to−height ratio
0.95
0.85
0.75
0.65
0.55
0.45
0.35
0.25
Body−mass index (kg/m2)
12.5
22.5
32.5
42.5
52.5
62.5
Hypertension prevalence
0.00
0.25
0.50
0.75
1.00

Appendix Figure 19: Prevalence of participants with hypertension who did not use anti-hypertensive medicines at different levels of waist circumference (WC) and body-mass index (BMI), by region.

Cells with ≤30 participants have been excluded from the figure because the results are less stable than at larger numbers. The number on each panel indicates the crude prevalence of participants with hypertension who did not use anti-hypertensive medicines among all participants in each region.

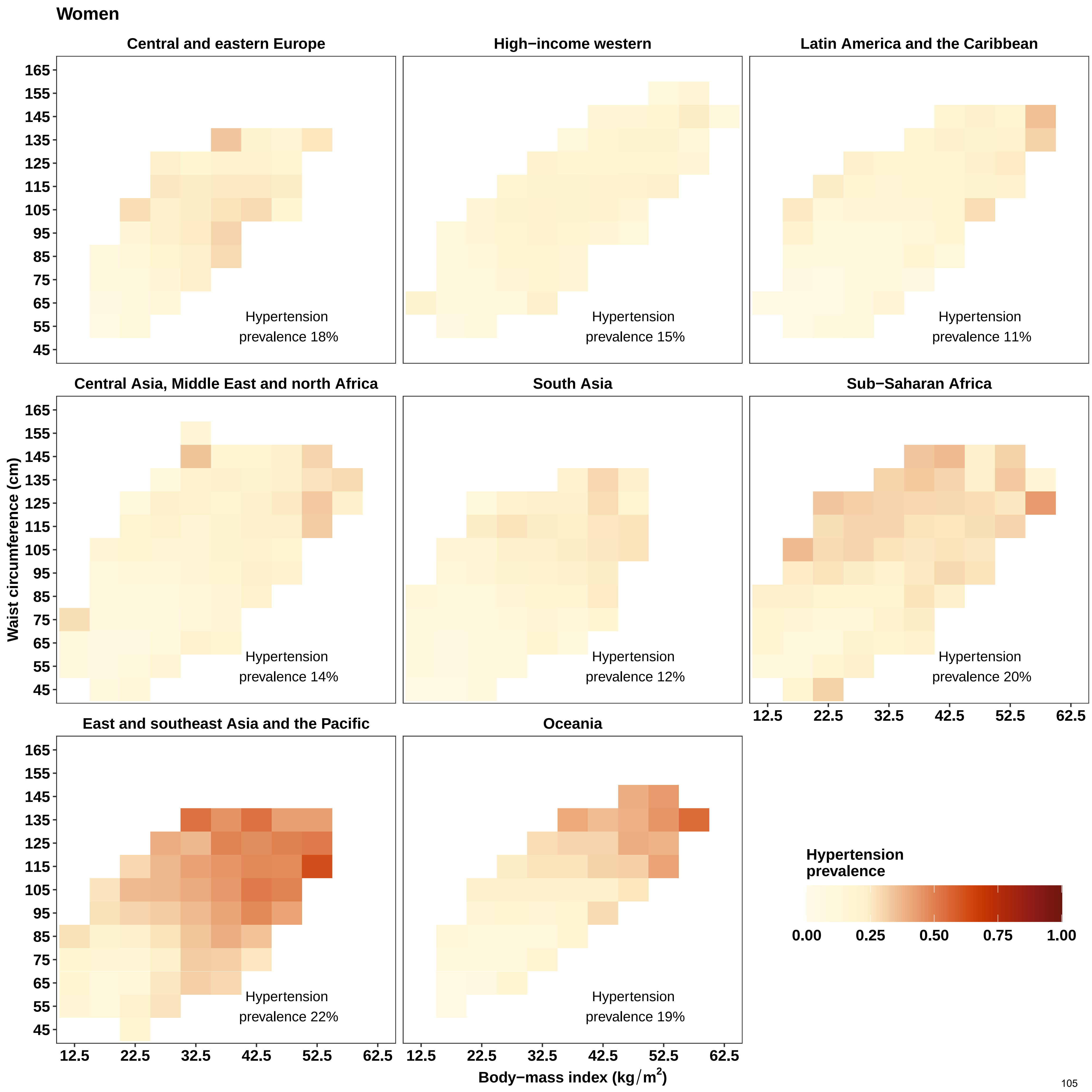
Women
Central and eastern Europe
High−income western
Latin America and the Caribbean
Central Asia, Middle East and north Africa
South Asia
Sub−Saharan Africa
East and southeast Asia and the Pacific
Oceania
Hypertension prevalence 18%
Hypertension prevalence 15%
Hypertension prevalence 11%
Hypertension prevalence 14%
Hypertension prevalence 12%
Hypertension prevalence 20%
Hypertension prevalence 22%
Hypertension prevalence 19%
Waist circumference (cm)
165
155
145
135
125
115
105
95
85
75
65
55
45
12.5
22.5
32.5
42.5
52.5
62.5
Body−mass index (kg/m2)
Hypertension prevalence
0.00
0.25
0.50
0.75
1.00

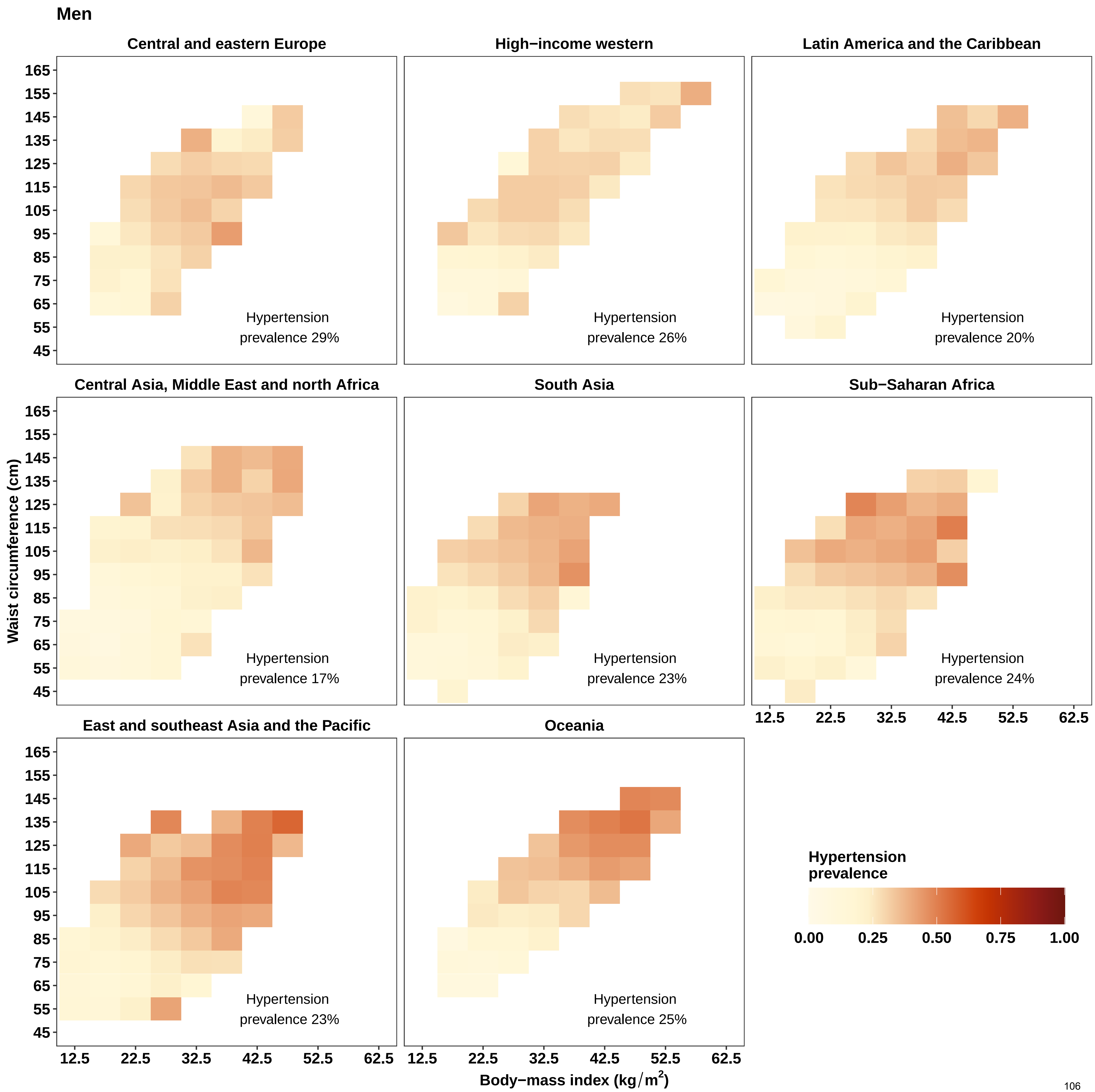

Men
Central and eastern Europe
High−income western
Latin America and the Caribbean
Central Asia, Middle East and north Africa
South Asia
Sub−Saharan Africa
East and southeast Asia and the Pacific
Oceania
Hypertension prevalence 29%
Hypertension prevalence 26%
Hypertension prevalence 20%
Hypertension prevalence 17%
Hypertension prevalence 23%
Hypertension prevalence 24%
Hypertension prevalence 23%
Hypertension prevalence 25%
Waist circumference (cm)
165
155
145
135
125
115
105
95
85
75
65
55
45
Body−mass index (kg/m^2)
12.5
22.5
32.5
42.5
52.5
62.5
Hypertension prevalence
0.00
0.25
0.50
0.75
1.00

Appendix Figure 20: Prevalence of participants with hypertension who used anti-hypertensive medicines at different levels of waist circumference (WC) and body-mass index (BMI), by region.

Cells with ≤30 participants have been excluded from the figure because the results are less stable than at larger numbers. The number on each panel indicates the crude prevalence of participants with hypertension who used anti-hypertensive medicines among all participants in each region.

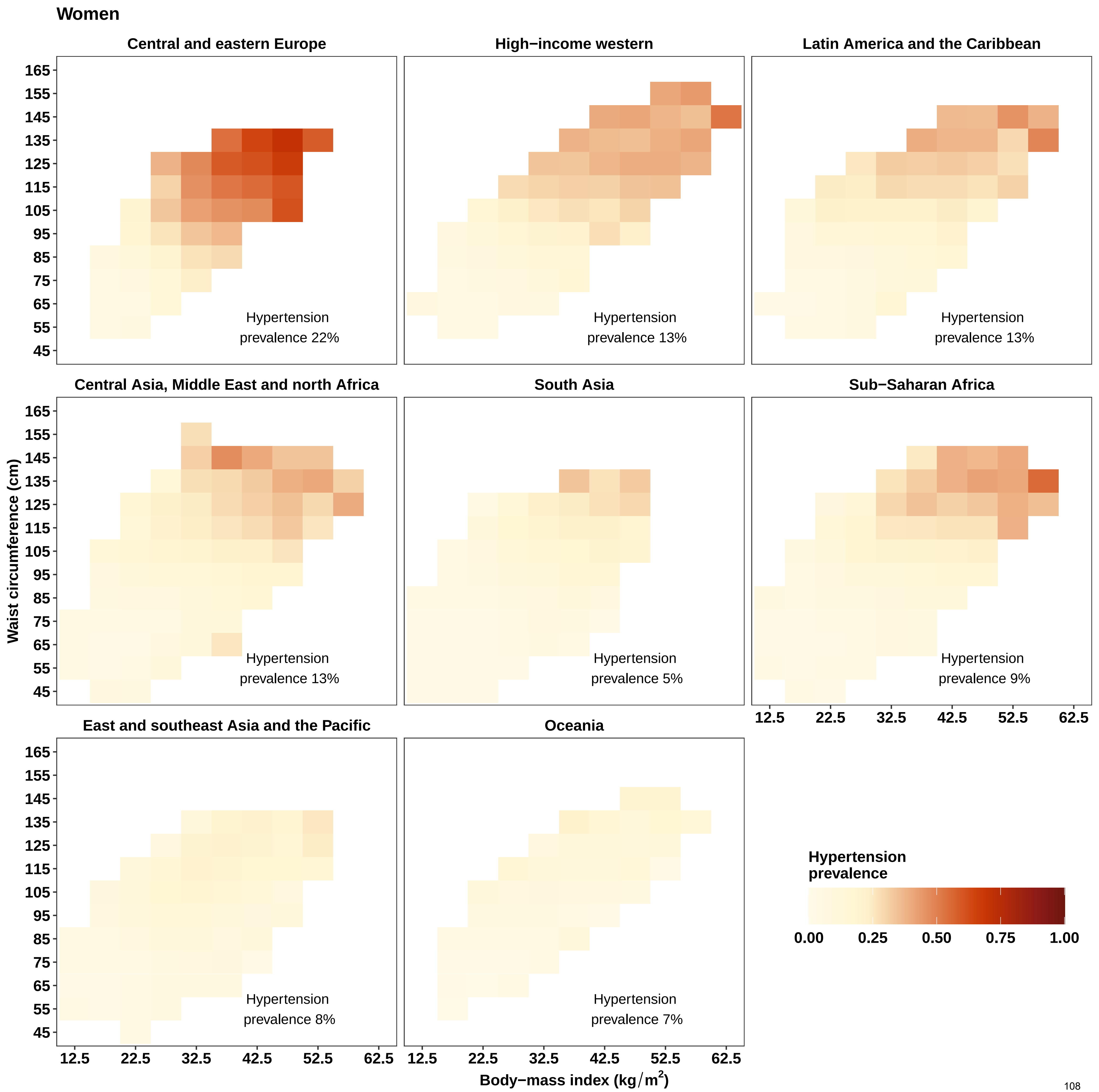
Women
Central and eastern Europe
Hypertension prevalence 22%
High−income western
Hypertension prevalence 13%
Latin America and the Caribbean
Hypertension prevalence 13%
Central Asia, Middle East and north Africa
Hypertension prevalence 13%
South Asia
Hypertension prevalence 5%
Sub−Saharan Africa
Hypertension prevalence 9%
East and southeast Asia and the Pacific
Hypertension prevalence 8%
Oceania
Hypertension prevalence 7%
Waist circumference (cm)
165 155 145 135 125 115 105 95 85 75 65 55 45
Body−mass index (kg/m2)
12.5 22.5 32.5 42.5 52.5 62.5
Hypertension prevalence
0.00 0.25 0.50 0.75 1.00

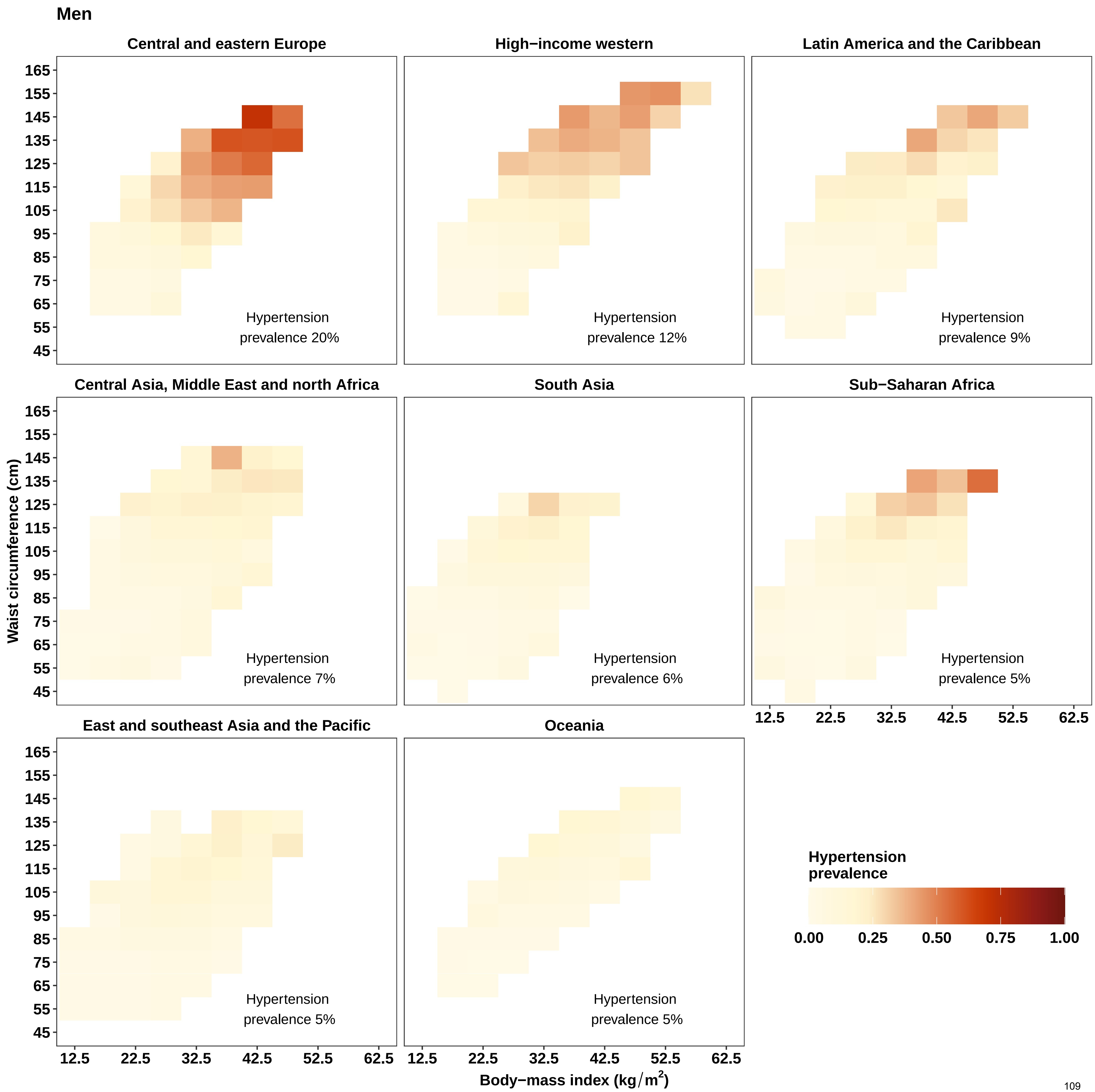

Men
Central and eastern Europe
High−income western
Latin America and the Caribbean
Central Asia, Middle East and north Africa
South Asia
Sub−Saharan Africa
East and southeast Asia and the Pacific
Oceania
Hypertension prevalence 20%
Hypertension prevalence 12%
Hypertension prevalence 9%
Hypertension prevalence 7%
Hypertension prevalence 6%
Hypertension prevalence 5%
Hypertension prevalence 5%
Hypertension prevalence 5%
Waist circumference (cm)
165
155
145
135
125
115
105
95
85
75
65
55
45
12.5
22.5
32.5
42.5
52.5
62.5
Body−mass index (kg/m2)
Hypertension prevalence
0.00
0.25
0.50
0.75
1.00

Appendix Figure 21: Distribution of participants with hypertension who did not use anti-hypertensive medicines in relation to body-mass index (BMI) and waist-to-height ratio (WHtR), by region.

The shading indicates the density of participants with hypertension who did not use anti-hypertensive medicines in each region, with darker shades corresponding to more participants. The vertical and horizontal lines show median BMI and WHtR, respectively, for all participants (black-dashed) and those with hypertension (grey-solid) globally. See Appendix Figures 23 for results using waist circumference (WC).

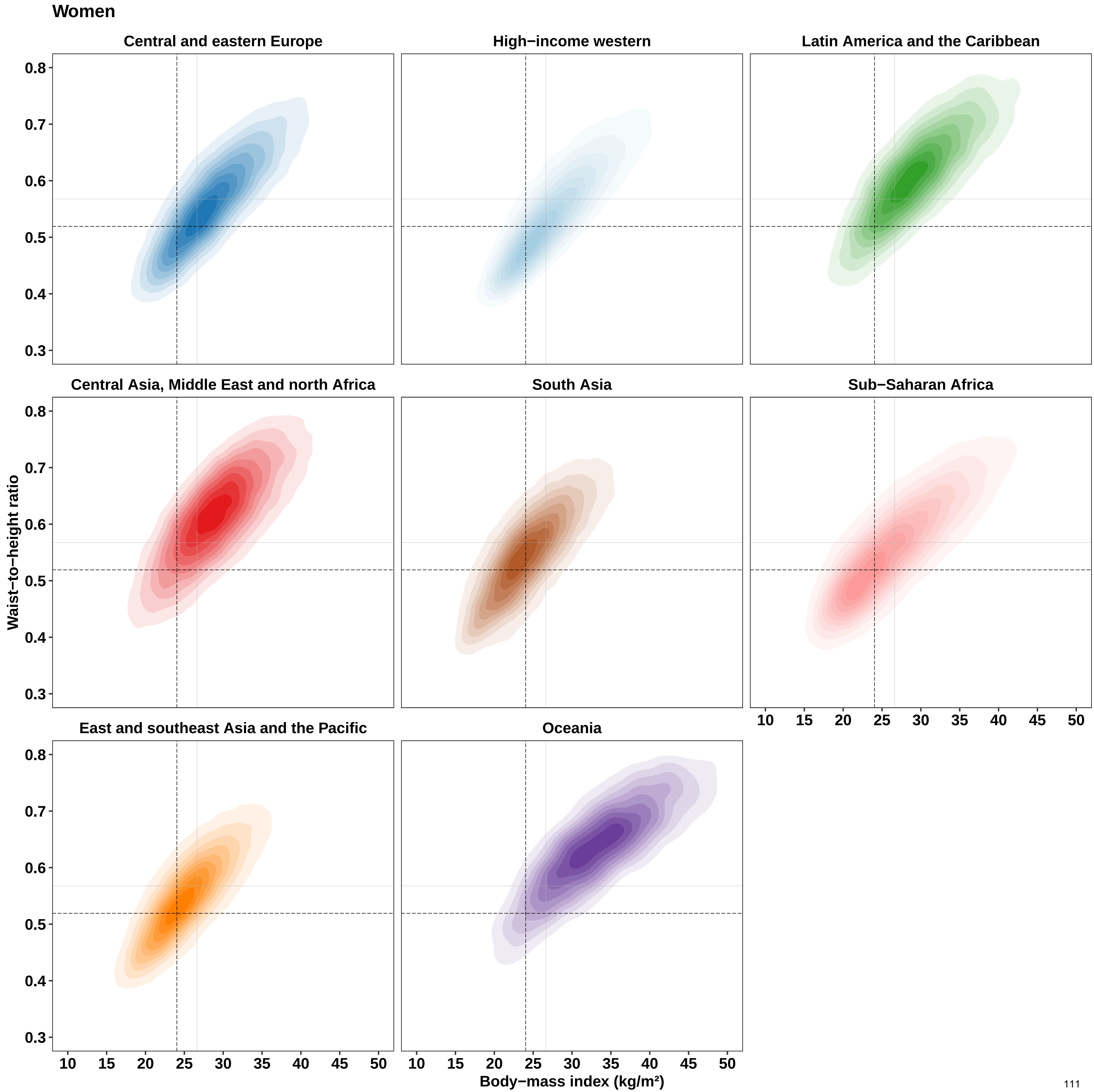
Women
Central and eastern Europe
High−income western
Latin America and the Caribbean
Central Asia, Middle East and north Africa
South Asia
Sub−Saharan Africa
East and southeast Asia and the Pacific
Oceania
Waist−to−height ratio
0.3
0.4
0.5
0.6
0.7
0.8
10
15
20
25
30
35
40
45
50
Body−mass index (kg/m²)

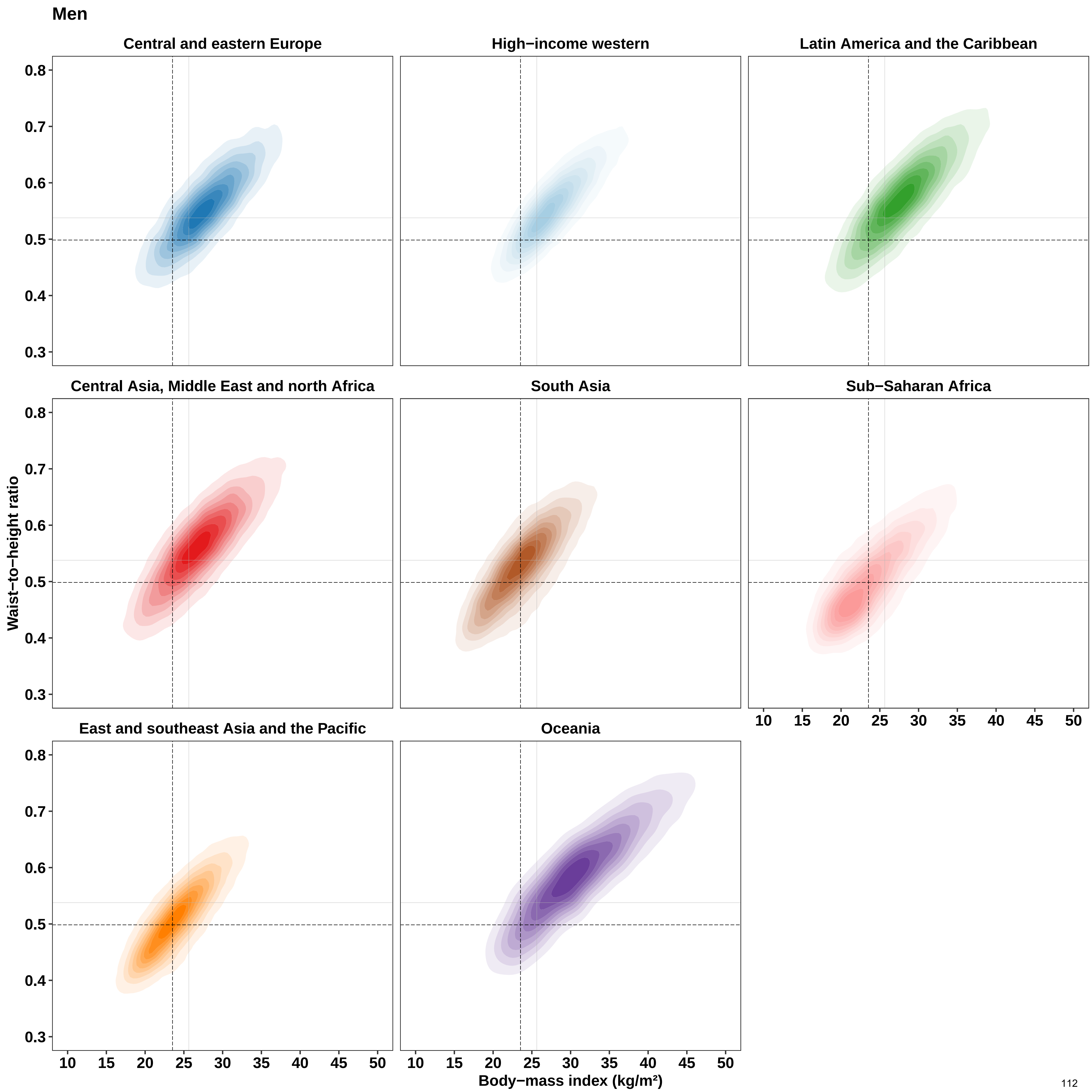
Men
Central and eastern Europe
High−income western
Latin America and the Caribbean
Central Asia, Middle East and north Africa
South Asia
Sub−Saharan Africa
East and southeast Asia and the Pacific
Oceania
Waist−to−height ratio
Body−mass index (kg/m²)
0.3
0.4
0.5
0.6
0.7
0.8
10
15
20
25
30
35
40
45
50

Appendix Figure 22: Distribution of participants with hypertension who used anti-hypertensive medicines in relation to body-mass index (BMI) and waist-to-height ratio (WHtR), by region.

The shading indicates the density of participants with hypertension who used anti-hypertensive medicines in each region, with darker shades corresponding to more participants. The vertical and horizontal lines show median BMI and WHtR, respectively, for all participants (black-dashed) and those with hypertension (grey-solid) globally. See Appendix Figures 24 for results using waist circumference (WC).

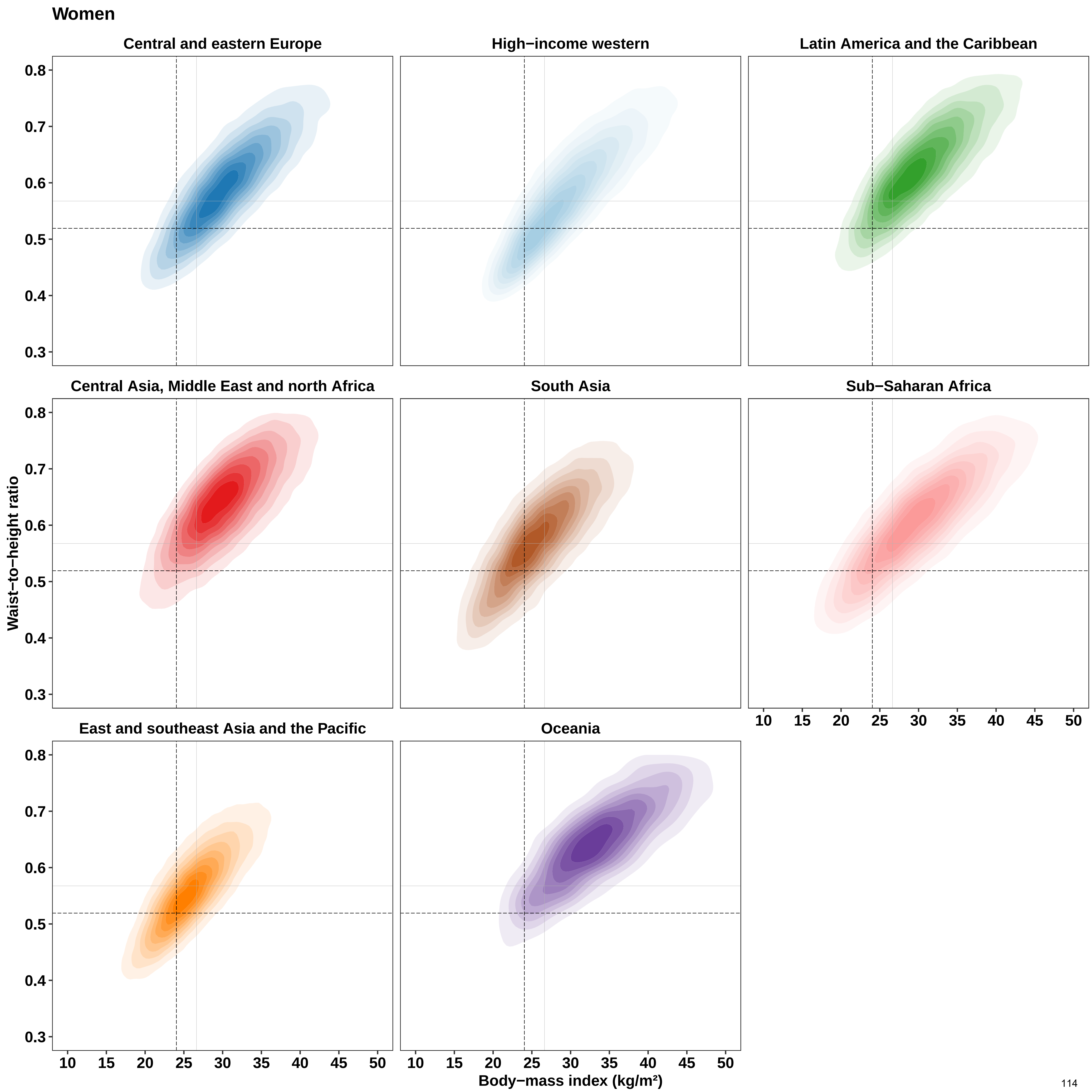

Women
Central and eastern Europe
High−income western
Latin America and the Caribbean
Central Asia, Middle East and north Africa
South Asia
Sub−Saharan Africa
East and southeast Asia and the Pacific
Oceania
Waist−to−height ratio
0.3
0.4
0.5
0.6
0.7
0.8
10
15
20
25
30
35
40
45
50
Body−mass index (kg/m²)

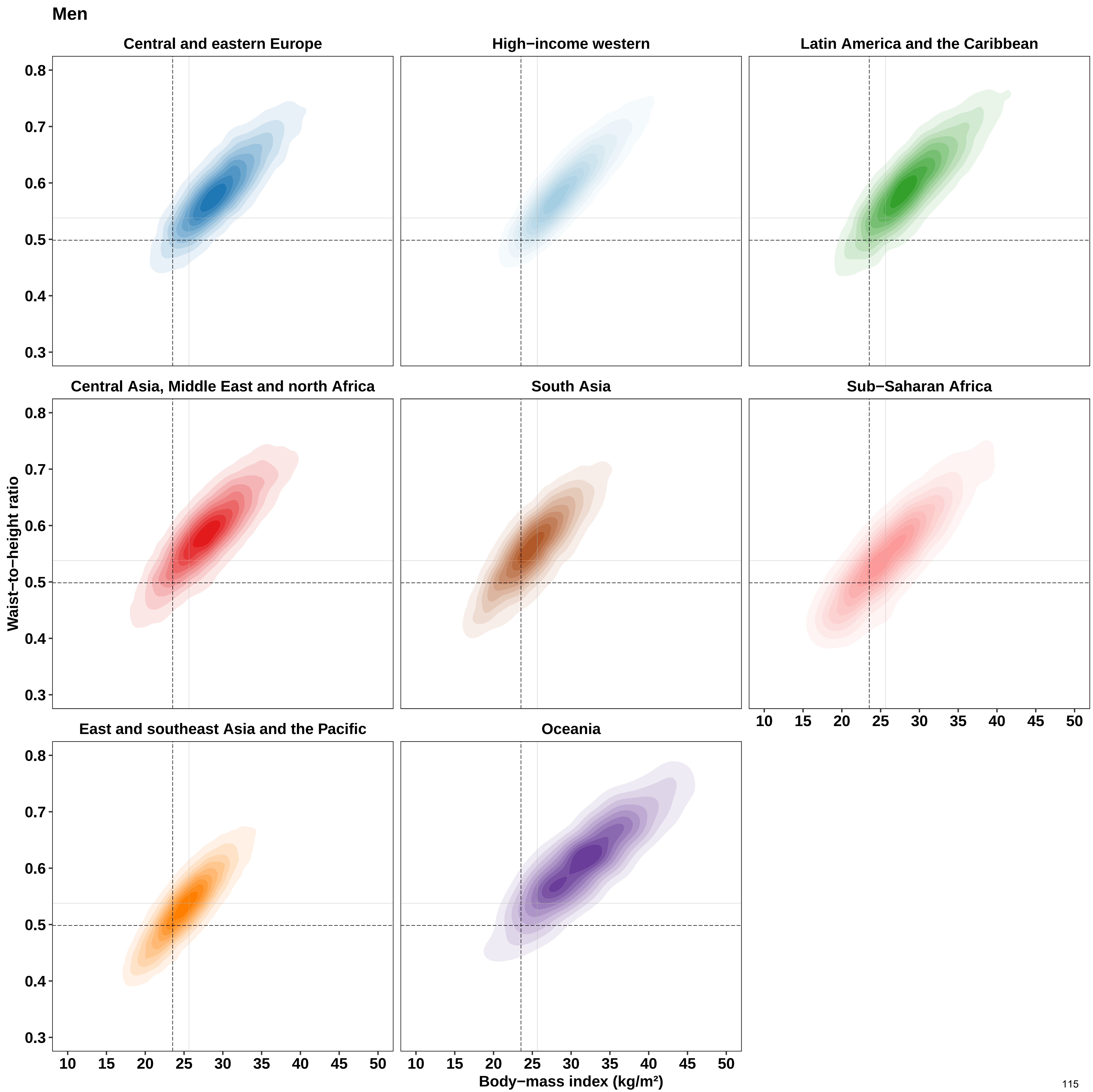
Men
Central and eastern Europe
High−income western
Latin America and the Caribbean
Central Asia, Middle East and north Africa
South Asia
Sub−Saharan Africa
East and southeast Asia and the Pacific
Oceania
Waist−to−height ratio
0.3
0.4
0.5
0.6
0.7
0.8
10
15
20
25
30
35
40
45
50
Body−mass index (kg/m²)

Appendix Figure 23: Distribution of participants with hypertension who did not use anti-hypertensive medicines in relation to body-mass index (BMI) and waist circumference (WC), by region.

The shading indicates the density of participants with hypertension who did not use anti-hypertensive medicines in each region, with darker shades corresponding to more participants. The vertical and horizontal lines show median BMI and WC, respectively, for all participants (black-dashed) and those with hypertension (grey-solid) globally.

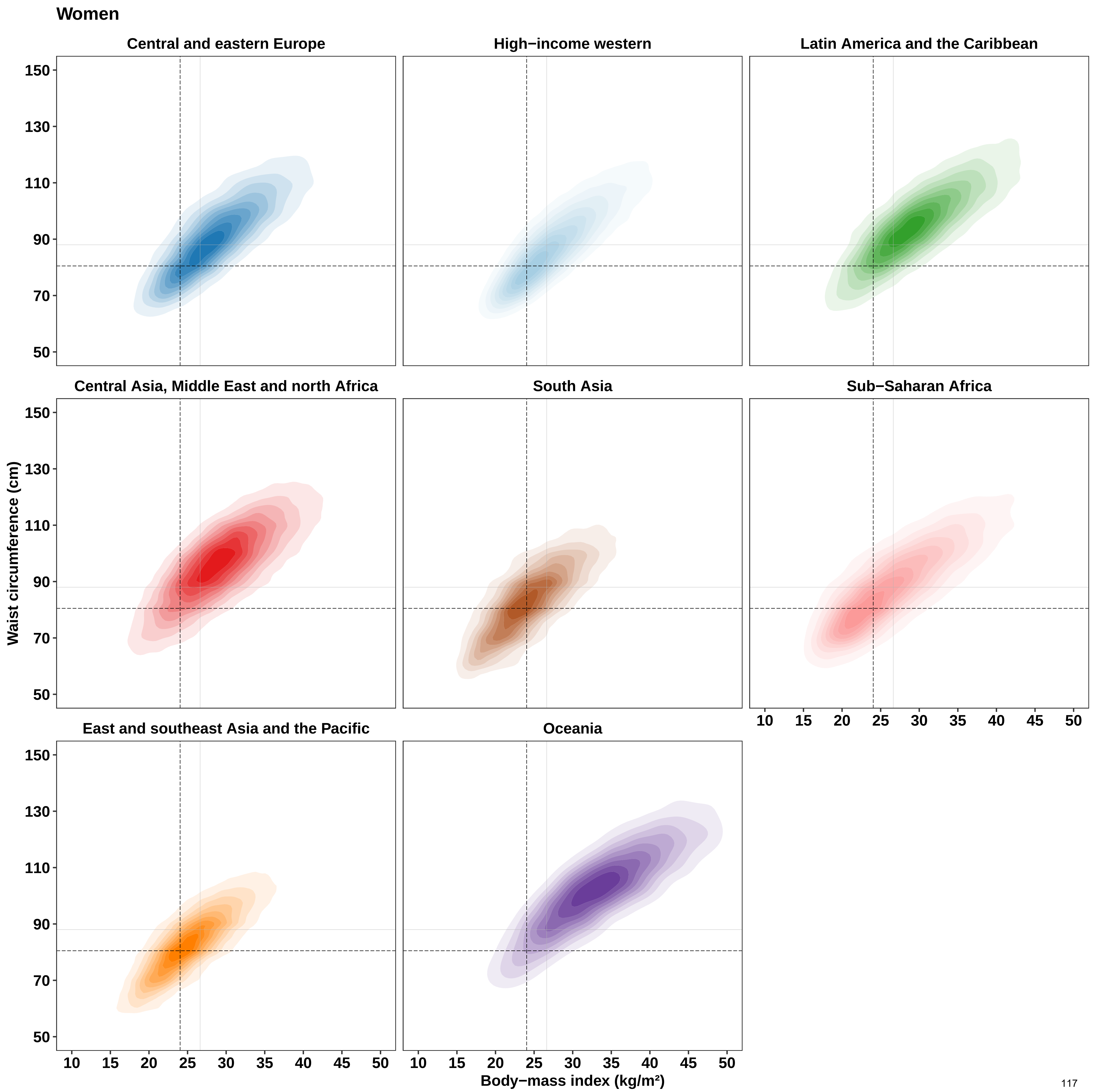

Women
Central and eastern Europe
High−income western
Latin America and the Caribbean
Central Asia, Middle East and north Africa
South Asia
Sub−Saharan Africa
East and southeast Asia and the Pacific
Oceania
Waist circumference (cm)
Body−mass index (kg/m²)
150
130
110
90
70
50
10
15
20
25
30
35
40
45
50

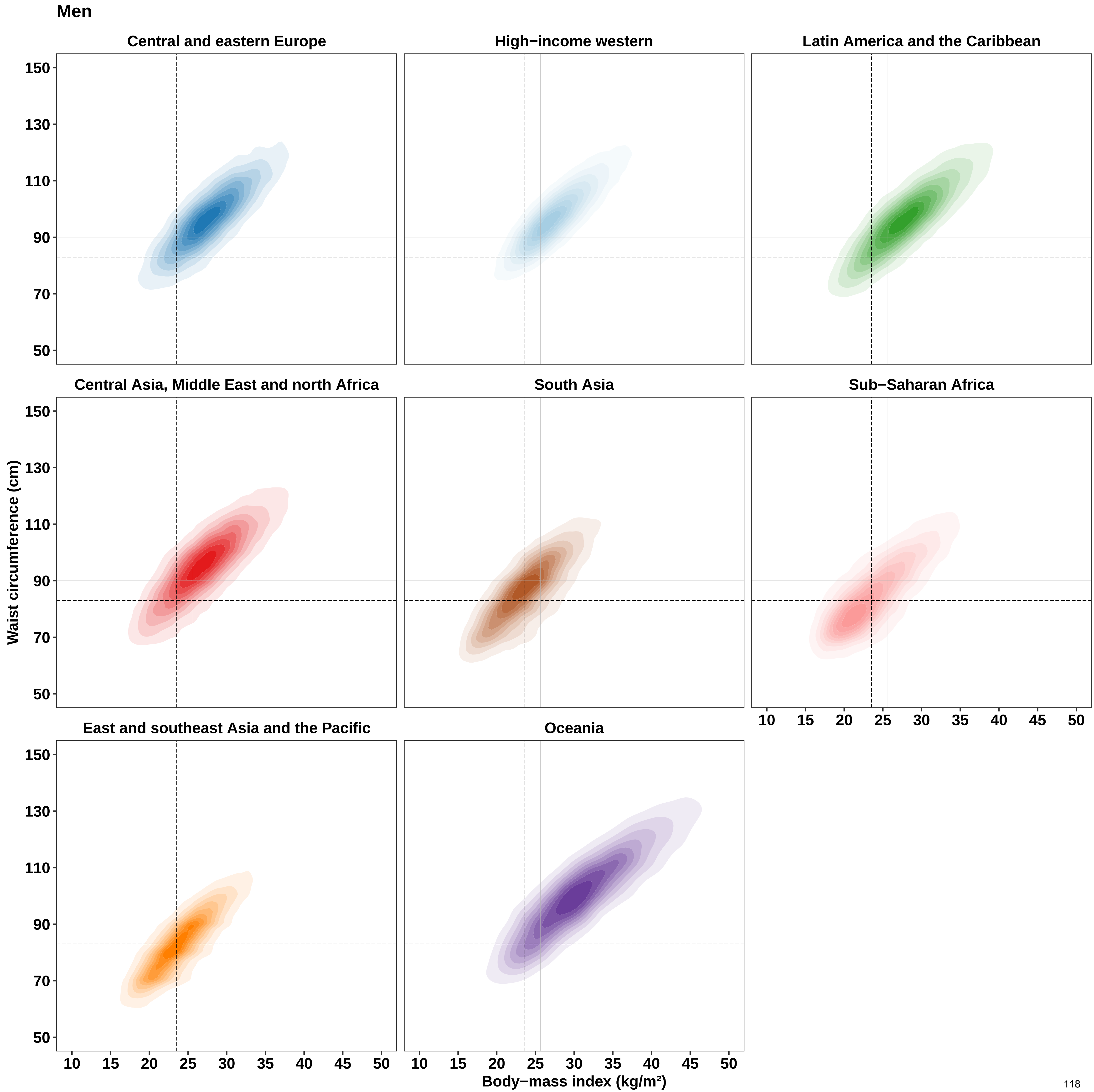

Men
Central and eastern Europe
High−income western
Latin America and the Caribbean
Central Asia, Middle East and north Africa
South Asia
Sub−Saharan Africa
East and southeast Asia and the Pacific
Oceania
Waist circumference (cm)
Body−mass index (kg/m²)
150
130
110
90
70
50
10
15
20
25
30
35
40
45
50

Appendix Figure 24: Distribution of participants with hypertension who used anti-hypertensive medicines in relation to body-mass index (BMI) and waist circumference (WC), by region.

The shading indicates the density of participants with hypertension who used anti-hypertensive medicines in each region, with darker shades corresponding to more participants. The vertical and horizontal lines show median BMI and WC, respectively, for all participants (black-dashed) and those with hypertension (grey-solid) globally.

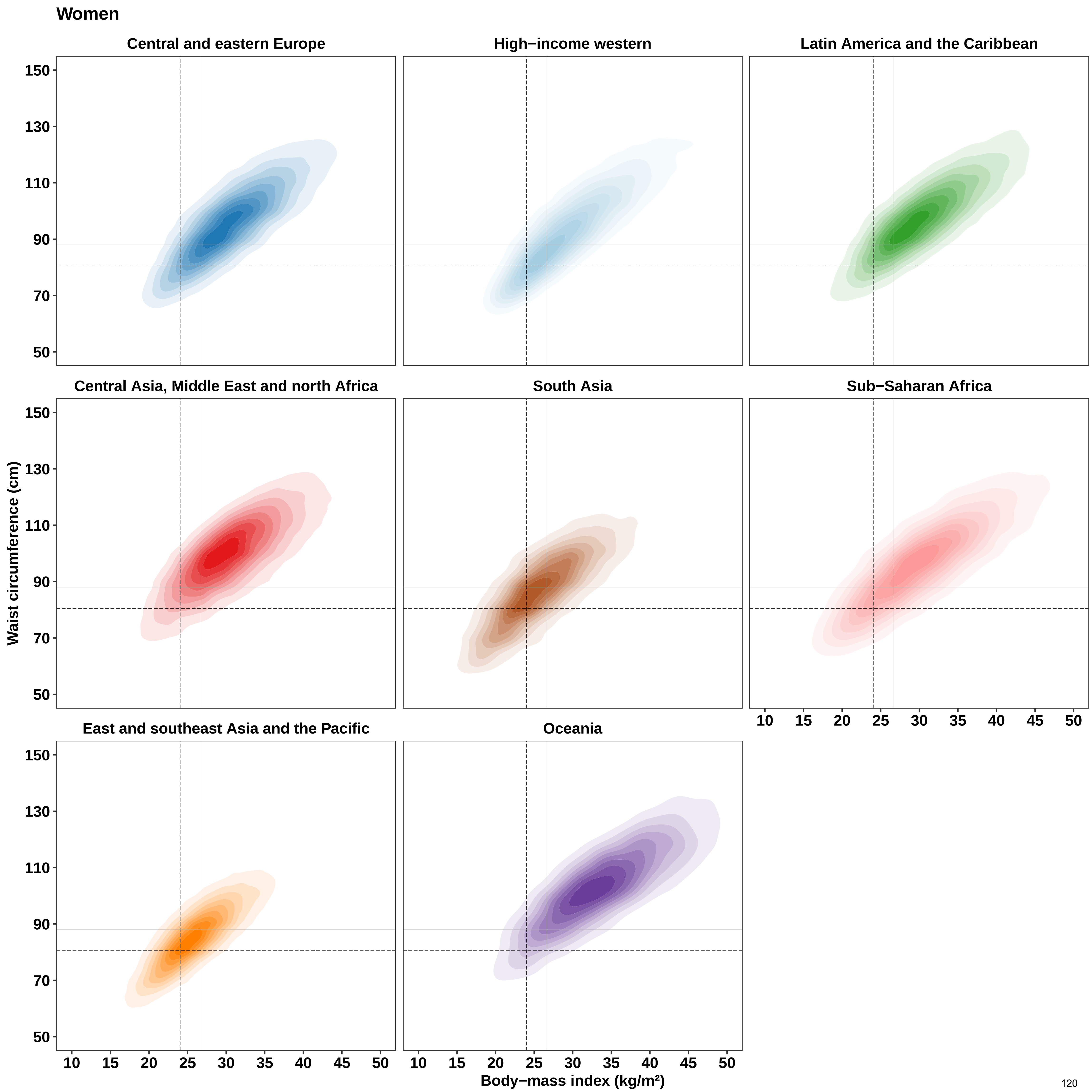

Women
Central and eastern Europe
High−income western
Latin America and the Caribbean
Central Asia, Middle East and north Africa
South Asia
Sub−Saharan Africa
East and southeast Asia and the Pacific
Oceania
Waist circumference (cm)
Body−mass index (kg/m²)
50
70
90
110
130
150
10
15
20
25
30
35
40
45
50

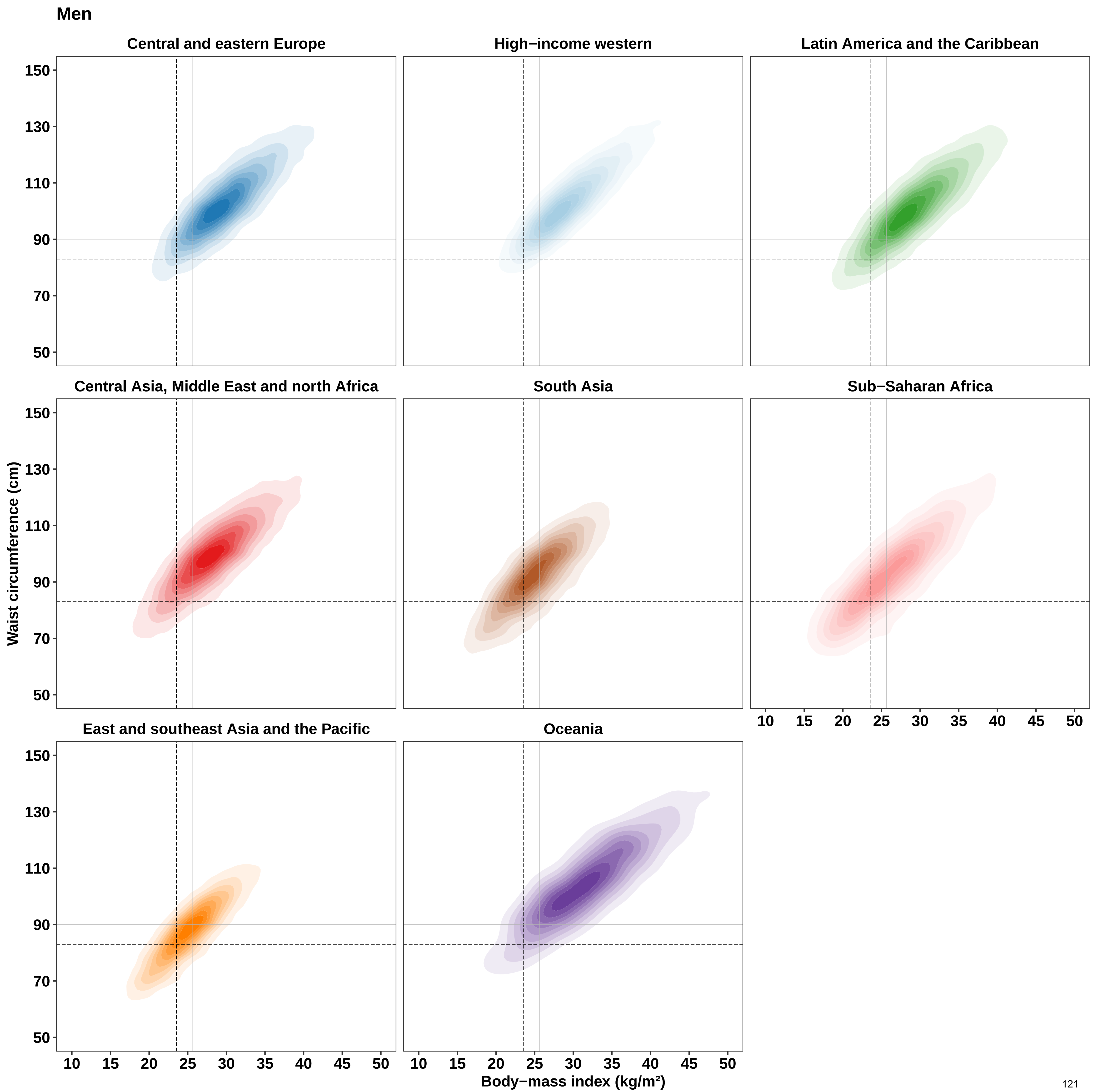

Men
Central and eastern Europe
High−income western
Latin America and the Caribbean
Central Asia, Middle East and north Africa
South Asia
Sub−Saharan Africa
East and southeast Asia and the Pacific
Oceania
Waist circumference (cm)
Body−mass index (kg/m²)
50
70
90
110
130
150
10
15
20
25
30
35
40
45
50

Appendix Figure 25: Mean systolic blood pressure (SBP) at different levels of waist circumference (WC) and body-mass index (BMI), by region.

Cells with ≤30 participants have been excluded from the figure because the results are less stable than at larger numbers. The number on each panel indicates the mean SBP among all participants in each region.

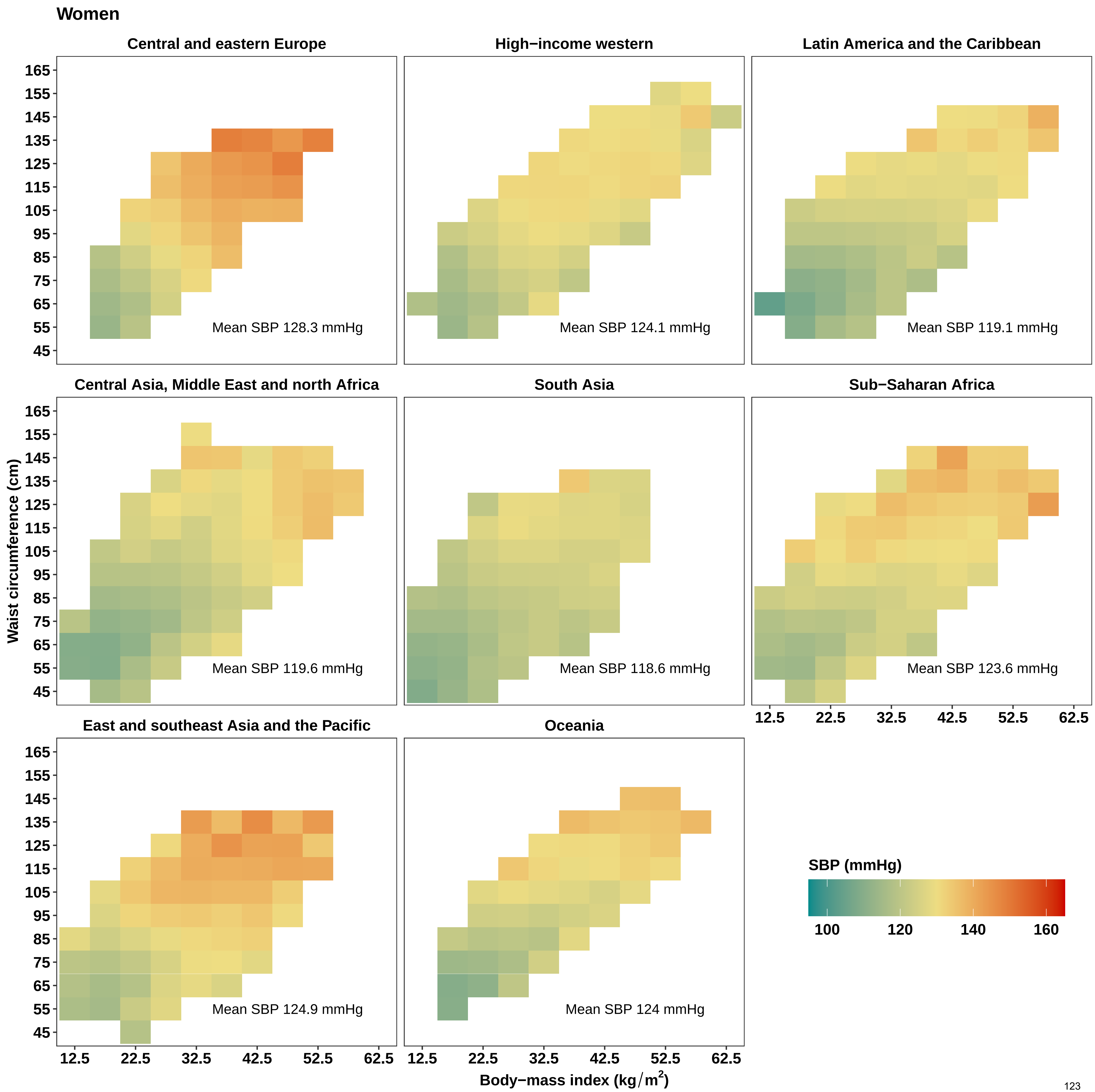

Women
Central and eastern Europe
High−income western
Latin America and the Caribbean
Central Asia, Middle East and north Africa
South Asia
Sub−Saharan Africa
East and southeast Asia and the Pacific
Oceania
Mean SBP 128.3 mmHg
Mean SBP 124.1 mmHg
Mean SBP 119.1 mmHg
Mean SBP 119.6 mmHg
Mean SBP 118.6 mmHg
Mean SBP 123.6 mmHg
Mean SBP 124.9 mmHg
Mean SBP 124 mmHg
Waist circumference (cm)
Body−mass index (kg/m²)
165 155 145 135 125 115 105 95 85 75 65 55 45
12.5 22.5 32.5 42.5 52.5 62.5
SBP (mmHg)
100 120 140 160

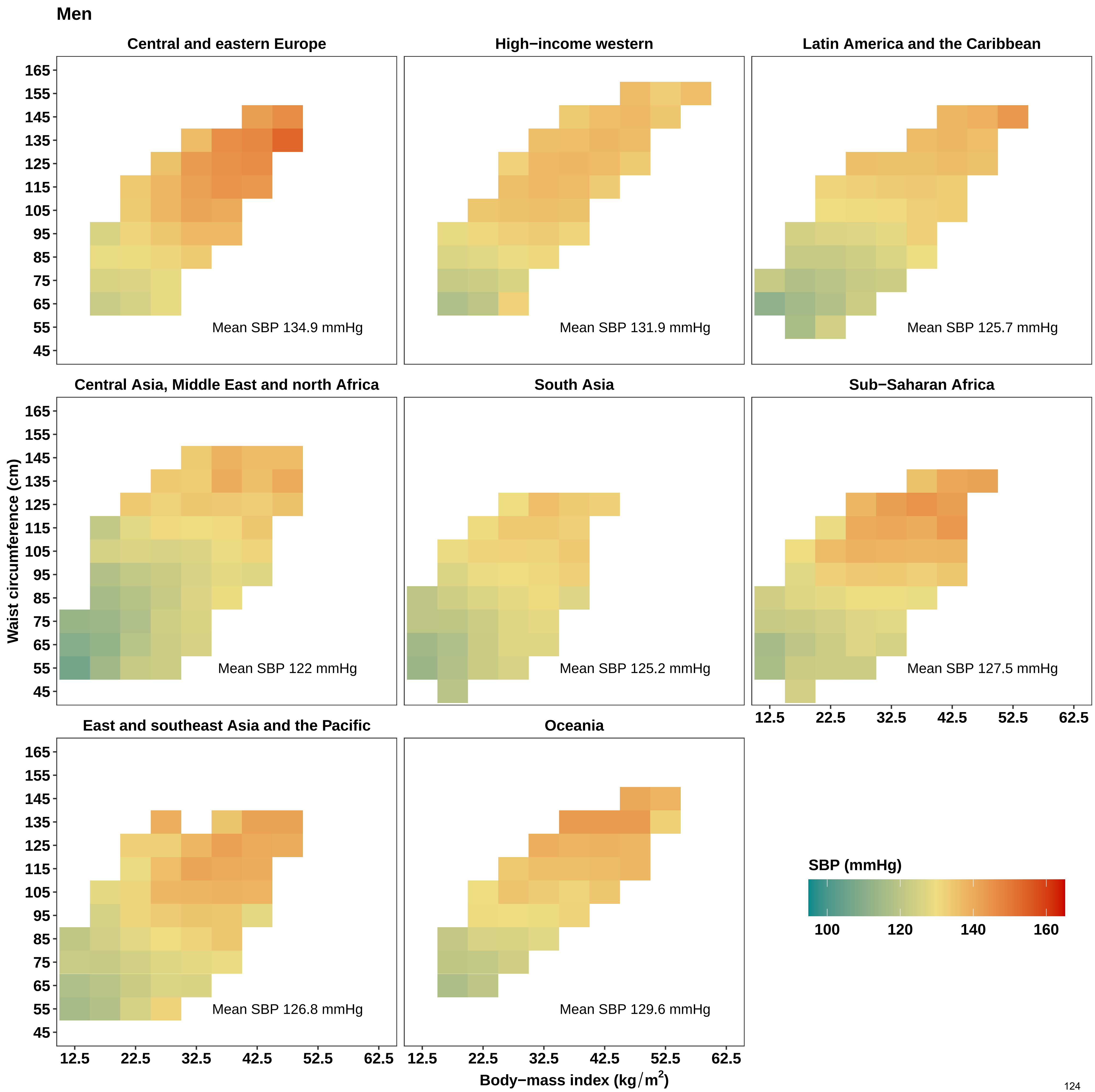

Men
Central and eastern Europe
High−income western
Latin America and the Caribbean
Central Asia, Middle East and north Africa
South Asia
Sub−Saharan Africa
East and southeast Asia and the Pacific
Oceania
Mean SBP 134.9 mmHg
Mean SBP 131.9 mmHg
Mean SBP 125.7 mmHg
Mean SBP 122 mmHg
Mean SBP 125.2 mmHg
Mean SBP 127.5 mmHg
Mean SBP 126.8 mmHg
Mean SBP 129.6 mmHg
Waist circumference (cm)
165
155
145
135
125
115
105
95
85
75
65
55
45
Body−mass index (kg/m²)
12.5
22.5
32.5
42.5
52.5
62.5
SBP (mmHg)
100
120
140
160

Appendix Figure 26: Mean diastolic blood pressure (DBP) at different levels of waist circumference (WC) and body-mass index (BMI), by region.

Cells with ≤30 participants have been excluded from the figure because the results are less stable than at larger numbers. The number on each panel indicates the mean DBP among all participants in each region.

Women

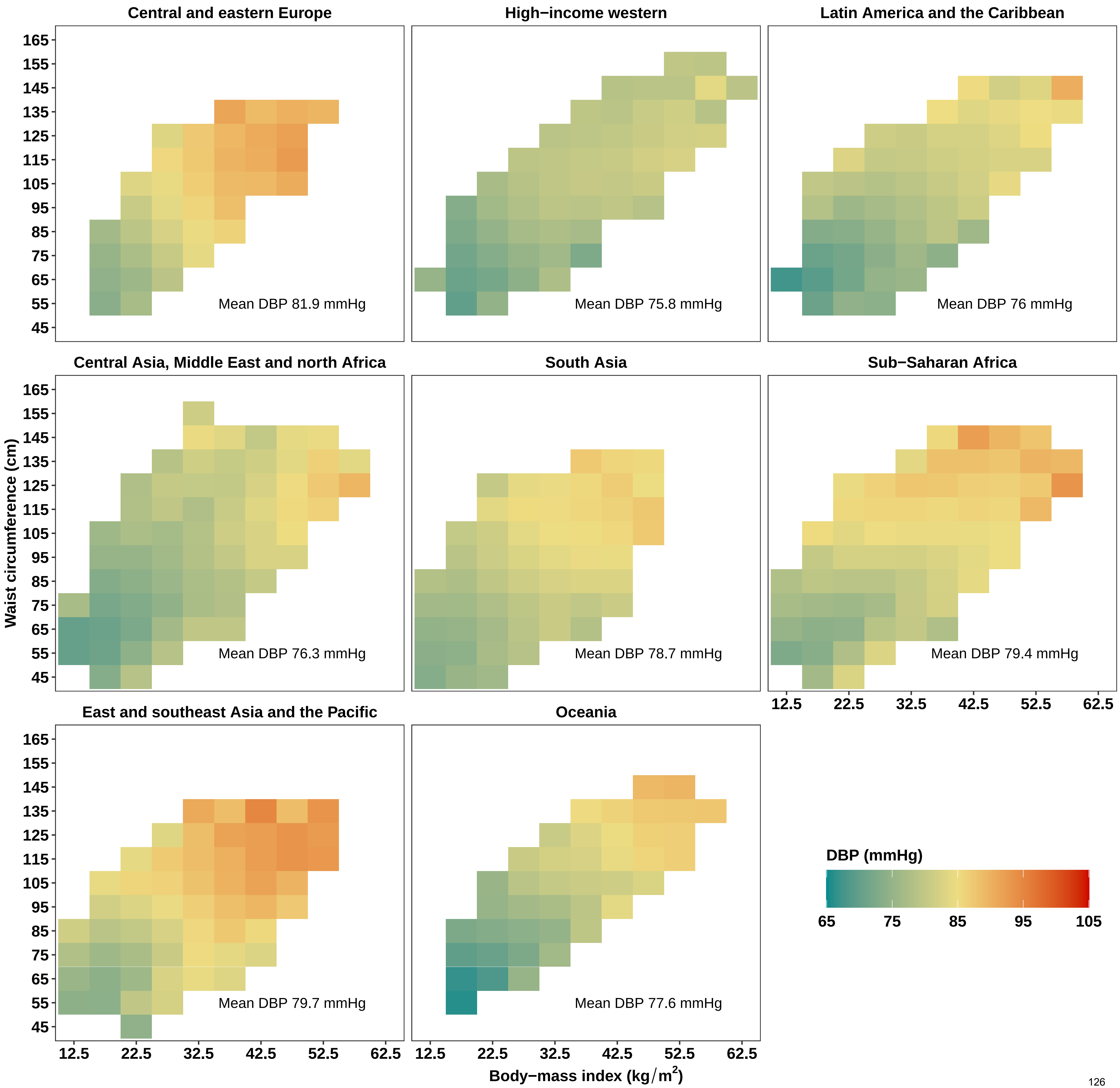

Central and eastern Europe
Mean DBP 81.9 mmHg
High−income western
Mean DBP 75.8 mmHg
Latin America and the Caribbean
Mean DBP 76 mmHg
Central Asia, Middle East and north Africa
Mean DBP 76.3 mmHg
South Asia
Mean DBP 78.7 mmHg
Sub−Saharan Africa
Mean DBP 79.4 mmHg
East and southeast Asia and the Pacific
Mean DBP 79.7 mmHg
Oceania
Mean DBP 77.6 mmHg
Waist circumference (cm)
165
155
145
135
125
115
105
95
85
75
65
55
45
Body−mass index (kg/m²)
12.5
22.5
32.5
42.5
52.5
62.5
DBP (mmHg)
65
75
85
95
105

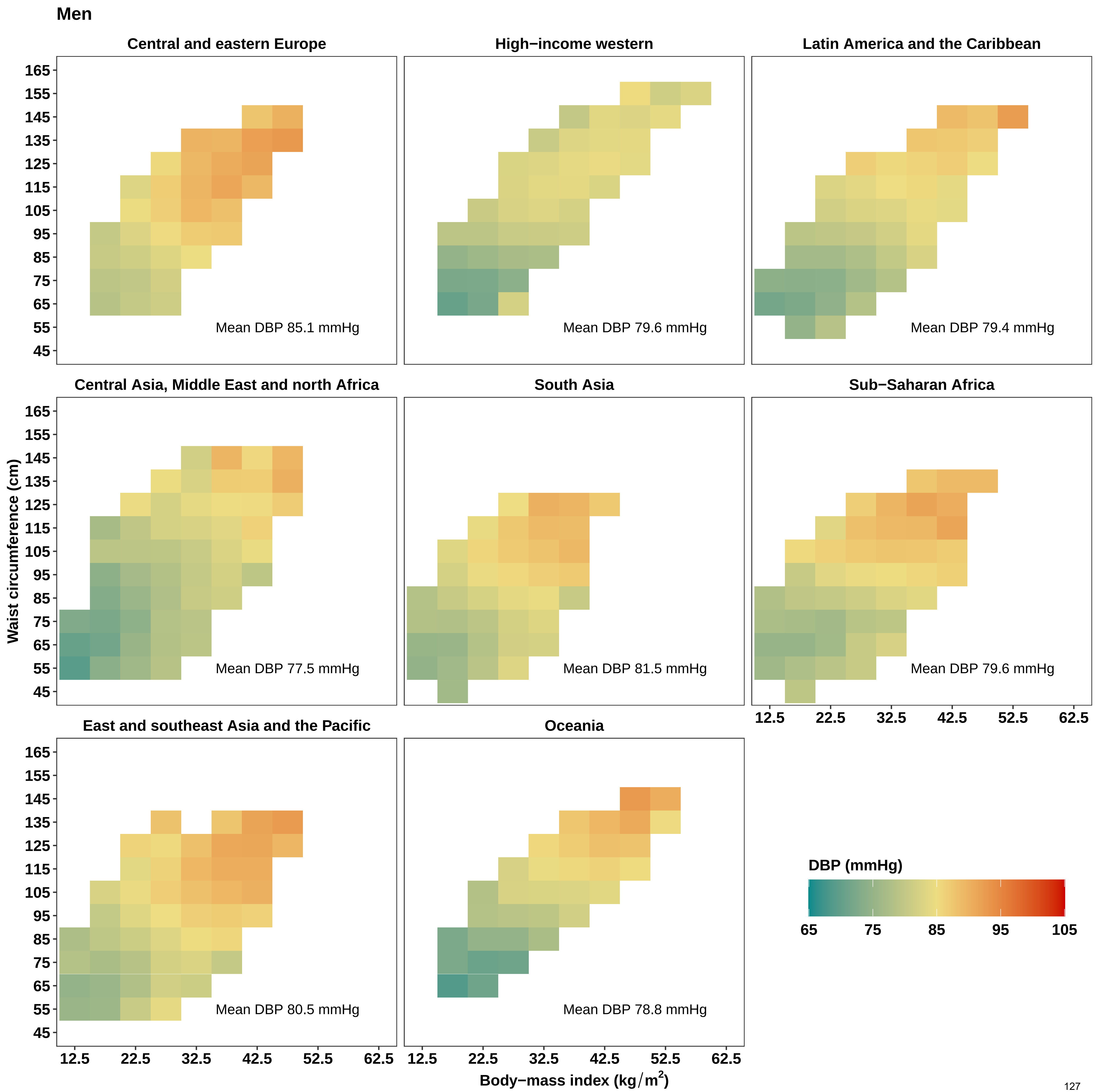

Men
Central and eastern Europe
High−income western
Latin America and the Caribbean
Central Asia, Middle East and north Africa
South Asia
Sub−Saharan Africa
East and southeast Asia and the Pacific
Oceania
Mean DBP 85.1 mmHg
Mean DBP 79.6 mmHg
Mean DBP 79.4 mmHg
Mean DBP 77.5 mmHg
Mean DBP 81.5 mmHg
Mean DBP 79.6 mmHg
Mean DBP 80.5 mmHg
Mean DBP 78.8 mmHg
Waist circumference (cm)
165 155 145 135 125 115 105 95 85 75 65 55 45
12.5 22.5 32.5 42.5 52.5 62.5
Body−mass index (kg/m^2)
DBP (mmHg)
65 75 85 95 105

Appendix Figure 27: Mean systolic blood pressure (SBP) of participants who did not use anti-hypertensive medicines at different levels of waist circumference (WC) and body-mass index (BMI), by region.

Cells with ≤30 participants have been excluded from the figure because the results are less stable than at larger numbers. The number on each panel indicates the mean SBP among all participants who did not use anti-hypertensive medicines in each region.

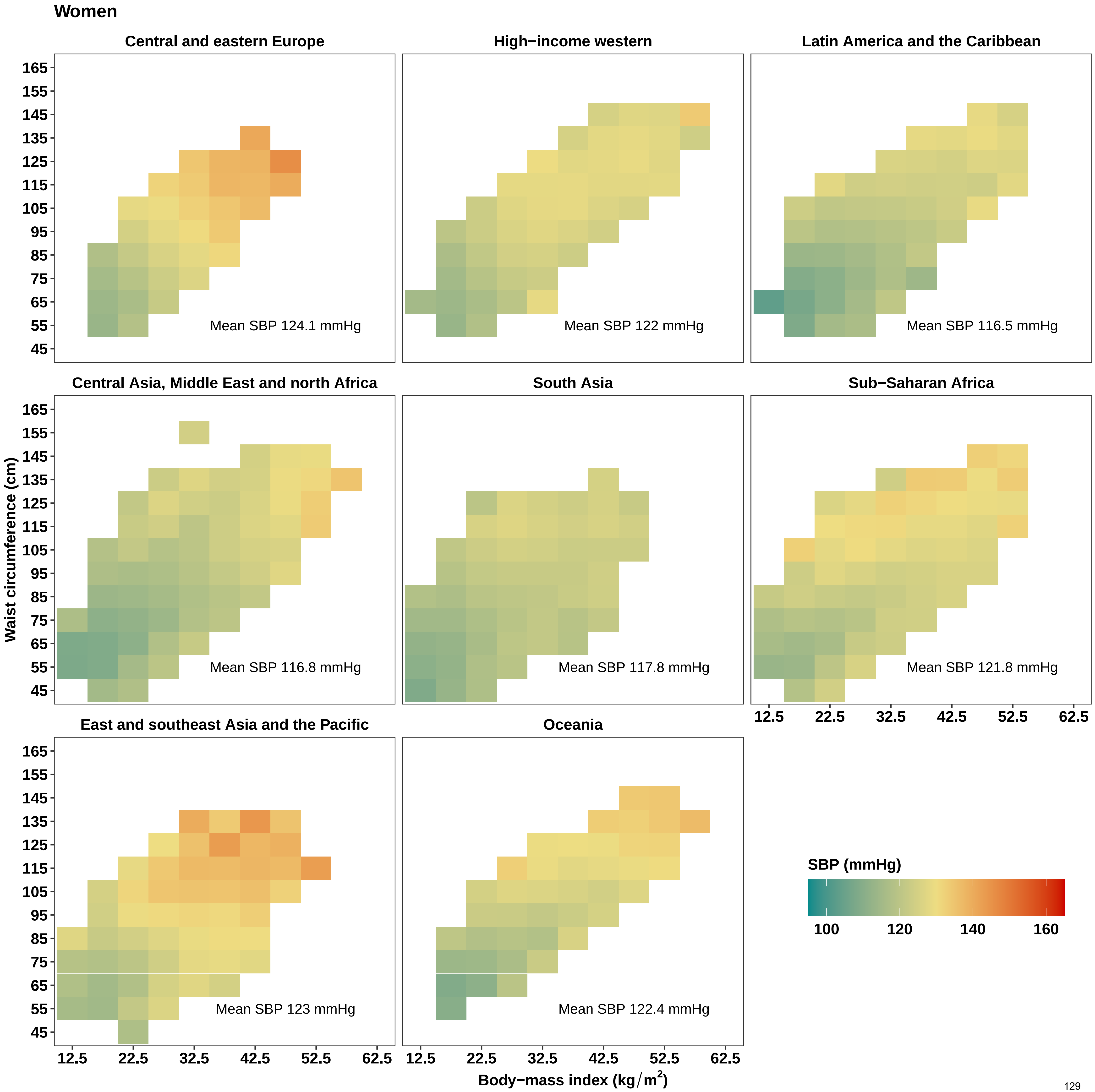

Women
Central and eastern Europe
Mean SBP 124.1 mmHg
High−income western
Mean SBP 122 mmHg
Latin America and the Caribbean
Mean SBP 116.5 mmHg
Central Asia, Middle East and north Africa
Mean SBP 116.8 mmHg
South Asia
Mean SBP 117.8 mmHg
Sub−Saharan Africa
Mean SBP 121.8 mmHg
East and southeast Asia and the Pacific
Mean SBP 123 mmHg
Oceania
Mean SBP 122.4 mmHg
Waist circumference (cm)
165
155
145
135
125
115
105
95
85
75
65
55
45
12.5
22.5
32.5
42.5
52.5
62.5
Body−mass index (kg/m²)
SBP (mmHg)
100
120
140
160

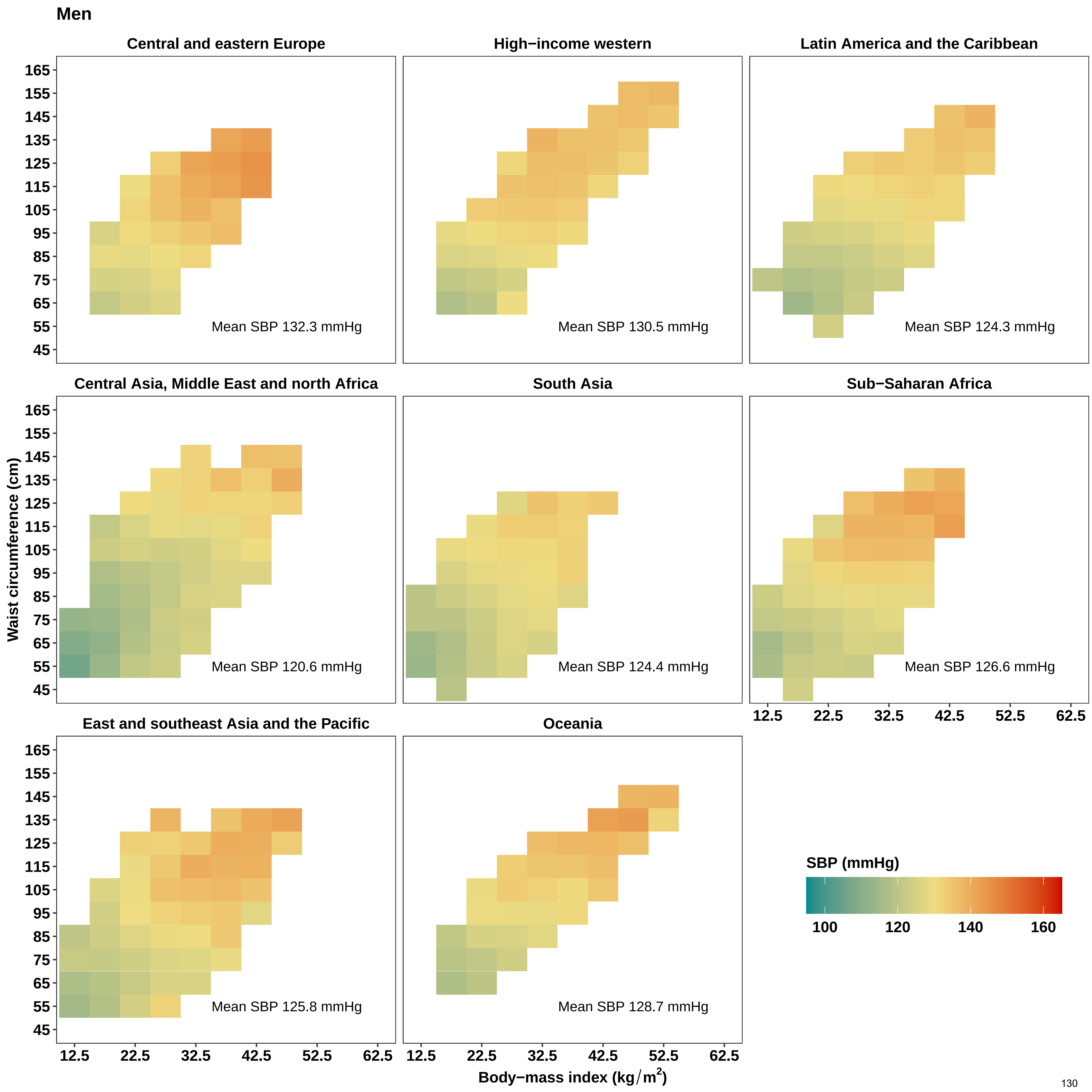

Men
Central and eastern Europe
Mean SBP 132.3 mmHg
High−income western
Mean SBP 130.5 mmHg
Latin America and the Caribbean
Mean SBP 124.3 mmHg
Central Asia, Middle East and north Africa
Mean SBP 120.6 mmHg
South Asia
Mean SBP 124.4 mmHg
Sub−Saharan Africa
Mean SBP 126.6 mmHg
East and southeast Asia and the Pacific
Mean SBP 125.8 mmHg
Oceania
Mean SBP 128.7 mmHg
Waist circumference (cm)
165
155
145
135
125
115
105
95
85
75
65
55
45
Body−mass index (kg/m2)
12.5
22.5
32.5
42.5
52.5
62.5
SBP (mmHg)
100
120
140
160

Appendix Figure 28: Mean diastolic blood pressure (DBP) of participants who did not use anti-hypertensive medicines at different levels of waist circumference (WC) and body-mass index (BMI), by region.

Cells with ≤30 participants have been excluded from the figure because the results are less stable than at larger numbers. The number on each panel indicates the mean DBP among all participants who did not use anti-hypertensive medicines in each region.

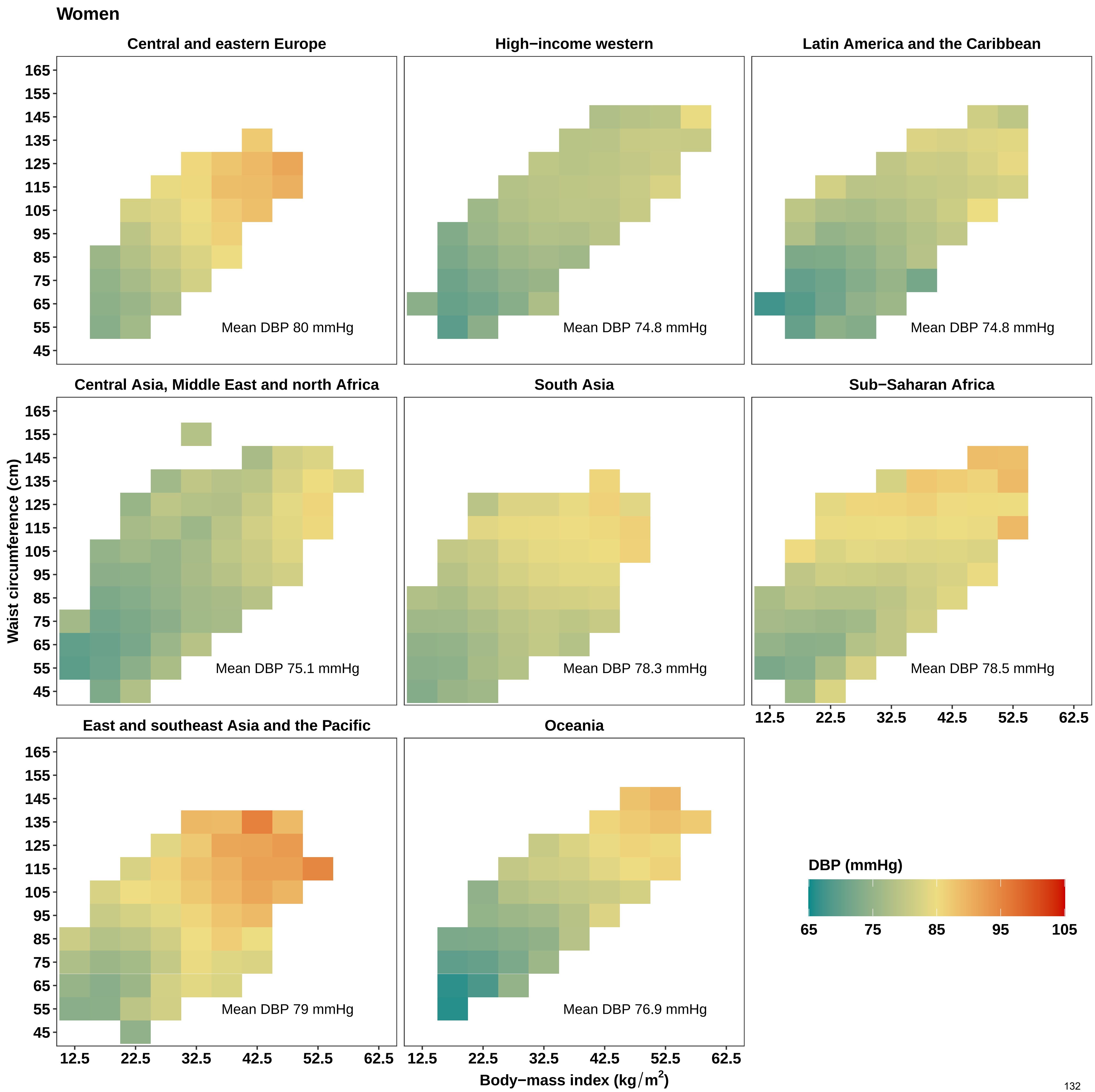
Women
Central and eastern Europe
High−income western
Latin America and the Caribbean
Central Asia, Middle East and north Africa
South Asia
Sub−Saharan Africa
East and southeast Asia and the Pacific
Oceania
Mean DBP 80 mmHg
Mean DBP 74.8 mmHg
Mean DBP 74.8 mmHg
Mean DBP 75.1 mmHg
Mean DBP 78.3 mmHg
Mean DBP 78.5 mmHg
Mean DBP 79 mmHg
Mean DBP 76.9 mmHg
Waist circumference (cm)
165
155
145
135
125
115
105
95
85
75
65
55
45
Body−mass index (kg/m2)
12.5
22.5
32.5
42.5
52.5
62.5
DBP (mmHg)
65
75
85
95
105

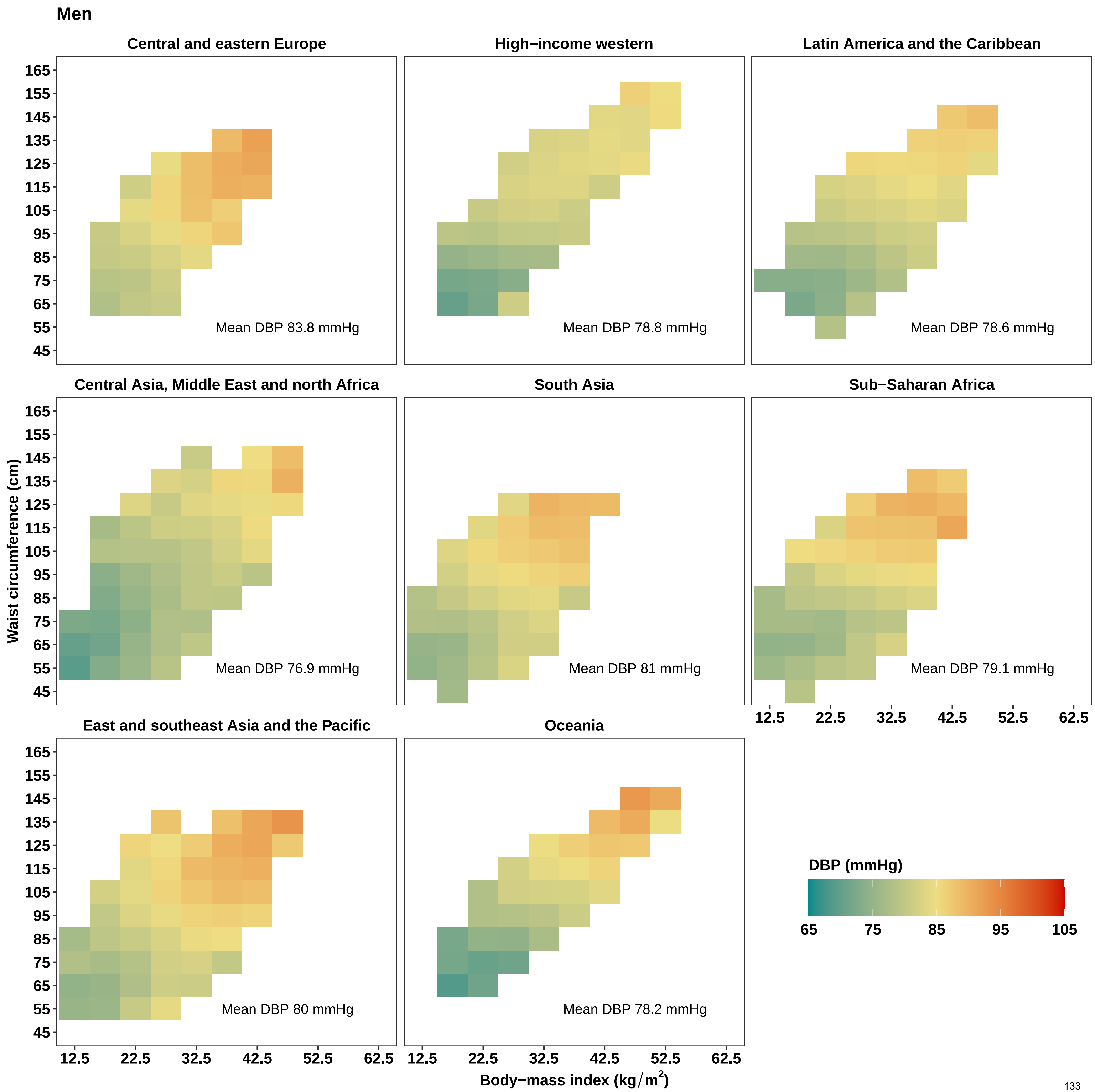
Men
Central and eastern Europe
High−income western
Latin America and the Caribbean
Central Asia, Middle East and north Africa
South Asia
Sub−Saharan Africa
East and southeast Asia and the Pacific
Oceania
Mean DBP 83.8 mmHg
Mean DBP 78.8 mmHg
Mean DBP 78.6 mmHg
Mean DBP 76.9 mmHg
Mean DBP 81 mmHg
Mean DBP 79.1 mmHg
Mean DBP 80 mmHg
Mean DBP 78.2 mmHg
Waist circumference (cm)
165
155
145
135
125
115
105
95
85
75
65
55
45
12.5
22.5
32.5
42.5
52.5
62.5
Body−mass index (kg/m2)
DBP (mmHg)
65
75
85
95
105

Appendix Figure 29: Mean systolic blood pressure (SBP) of participants who used anti-hypertensive medicines at different levels of waist circumference (WC) and body-mass index (BMI), by region.

Cells with ≤30 participants have been excluded from the figure because the results are less stable than at larger numbers. The number on each panel indicates the mean SBP among all participants who used anti-hypertensive medicines in each region.

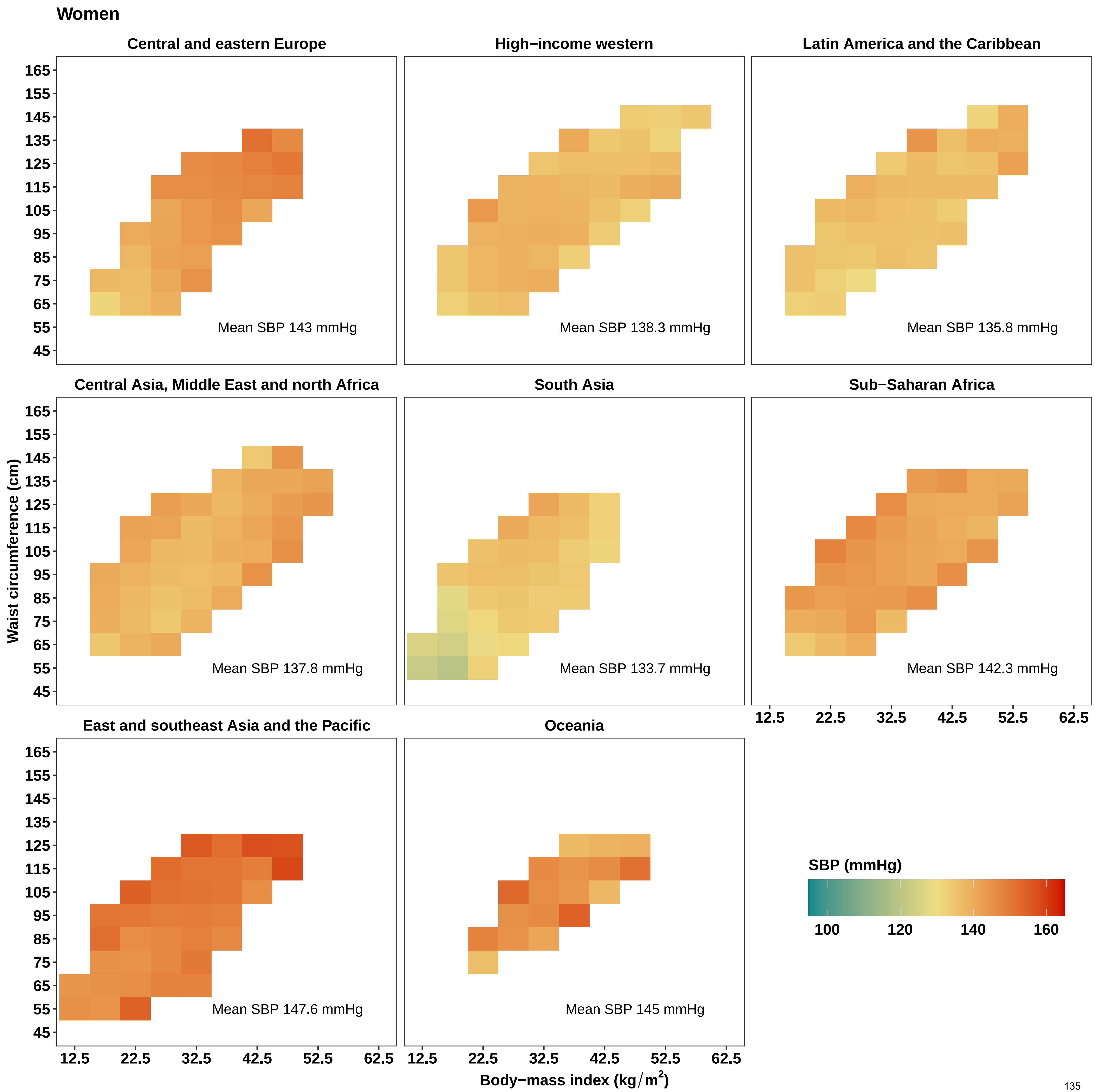

Women
Central and eastern Europe
High−income western
Latin America and the Caribbean
Central Asia, Middle East and north Africa
South Asia
Sub−Saharan Africa
East and southeast Asia and the Pacific
Oceania
Mean SBP 143 mmHg
Mean SBP 138.3 mmHg
Mean SBP 135.8 mmHg
Mean SBP 137.8 mmHg
Mean SBP 133.7 mmHg
Mean SBP 142.3 mmHg
Mean SBP 147.6 mmHg
Mean SBP 145 mmHg
Waist circumference (cm)
165
155
145
135
125
115
105
95
85
75
65
55
45
Body−mass index (kg/m2)
12.5
22.5
32.5
42.5
52.5
62.5
SBP (mmHg)
100
120
140
160

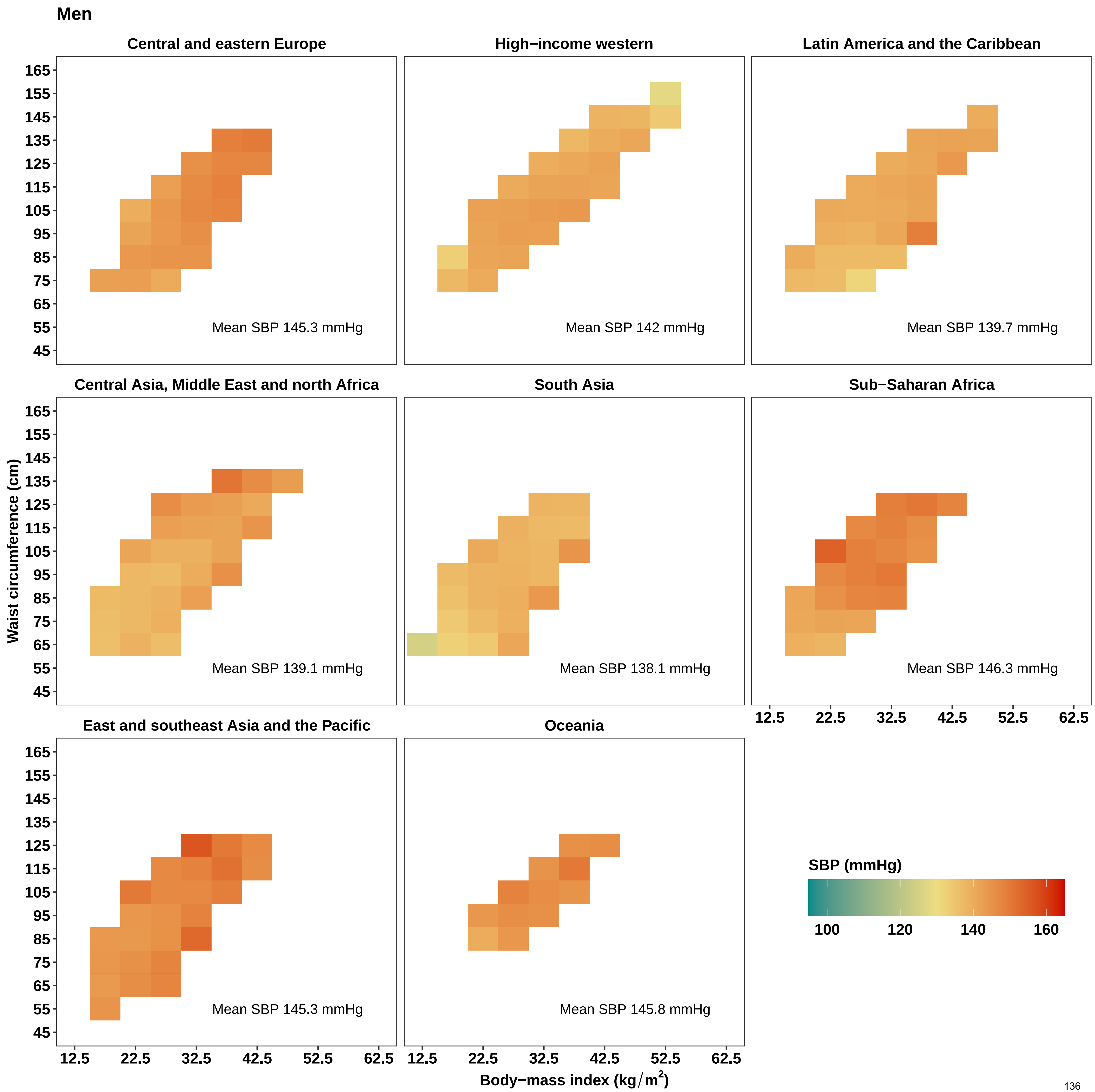

Men
Central and eastern Europe
Mean SBP 145.3 mmHg
High−income western
Mean SBP 142 mmHg
Latin America and the Caribbean
Mean SBP 139.7 mmHg
Central Asia, Middle East and north Africa
Mean SBP 139.1 mmHg
South Asia
Mean SBP 138.1 mmHg
Sub−Saharan Africa
Mean SBP 146.3 mmHg
East and southeast Asia and the Pacific
Mean SBP 145.3 mmHg
Oceania
Mean SBP 145.8 mmHg
Waist circumference (cm)
165
155
145
135
125
115
105
95
85
75
65
55
45
Body−mass index (kg/m²)
12.5
22.5
32.5
42.5
52.5
62.5
SBP (mmHg)
100
120
140
160

Appendix Figure 30: Mean diastolic blood pressure (DBP) of participants who used anti-hypertensive medicines at different levels of waist circumference (WC) and body-mass index (BMI), by region.

Cells with ≤30 participants have been excluded from the figure because the results are less stable than at larger numbers. The number on each panel indicates the mean DBP among all participants who used anti-hypertensive medicines in each region.

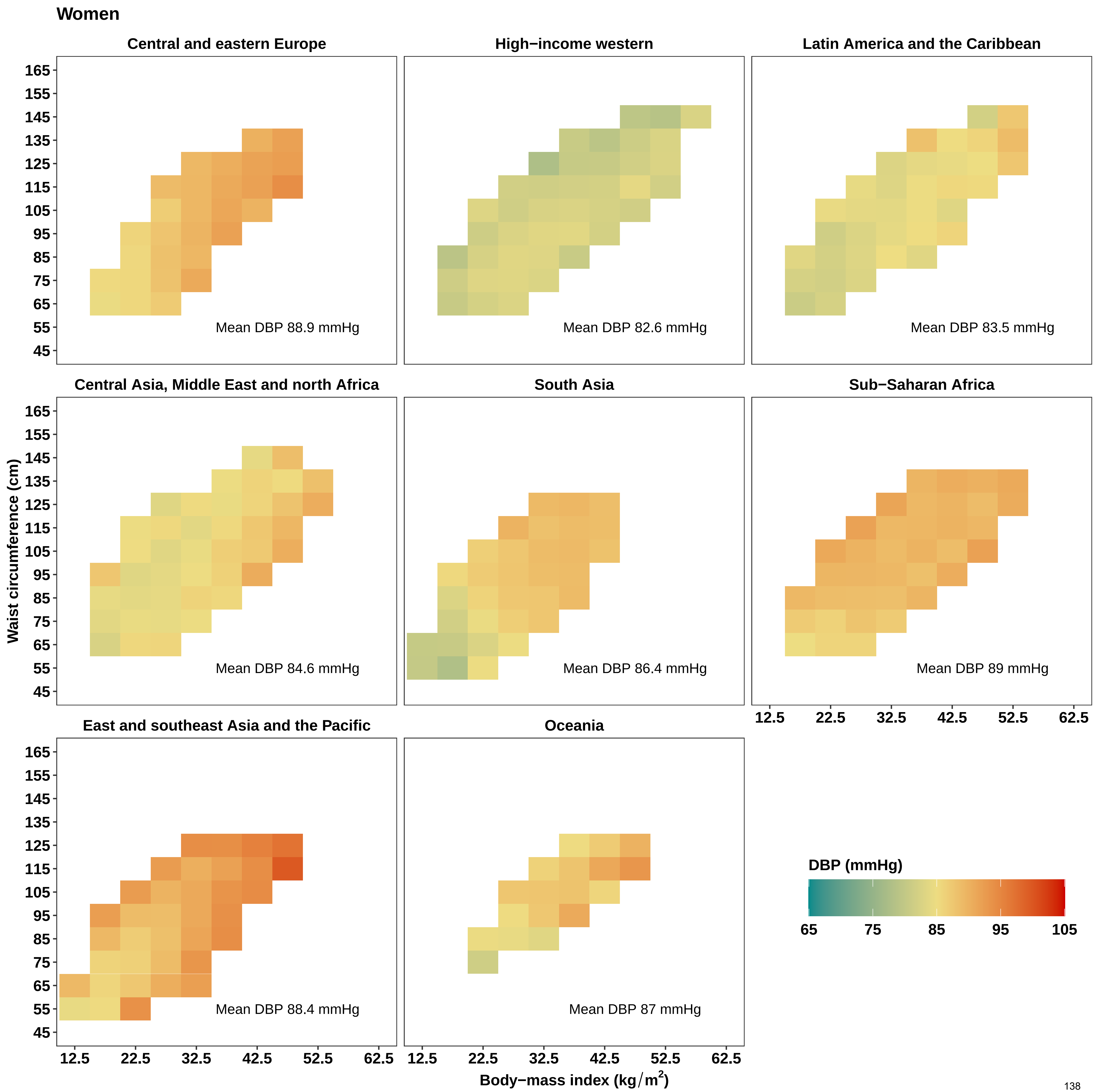

Women
Central and eastern Europe
Mean DBP 88.9 mmHg
High−income western
Mean DBP 82.6 mmHg
Latin America and the Caribbean
Mean DBP 83.5 mmHg
Central Asia, Middle East and north Africa
Mean DBP 84.6 mmHg
South Asia
Mean DBP 86.4 mmHg
Sub−Saharan Africa
Mean DBP 89 mmHg
East and southeast Asia and the Pacific
Mean DBP 88.4 mmHg
Oceania
Mean DBP 87 mmHg
Waist circumference (cm)
165
155
145
135
125
115
105
95
85
75
65
55
45
Body−mass index (kg/m²)
12.5
22.5
32.5
42.5
52.5
62.5
DBP (mmHg)
65
75
85
95
105

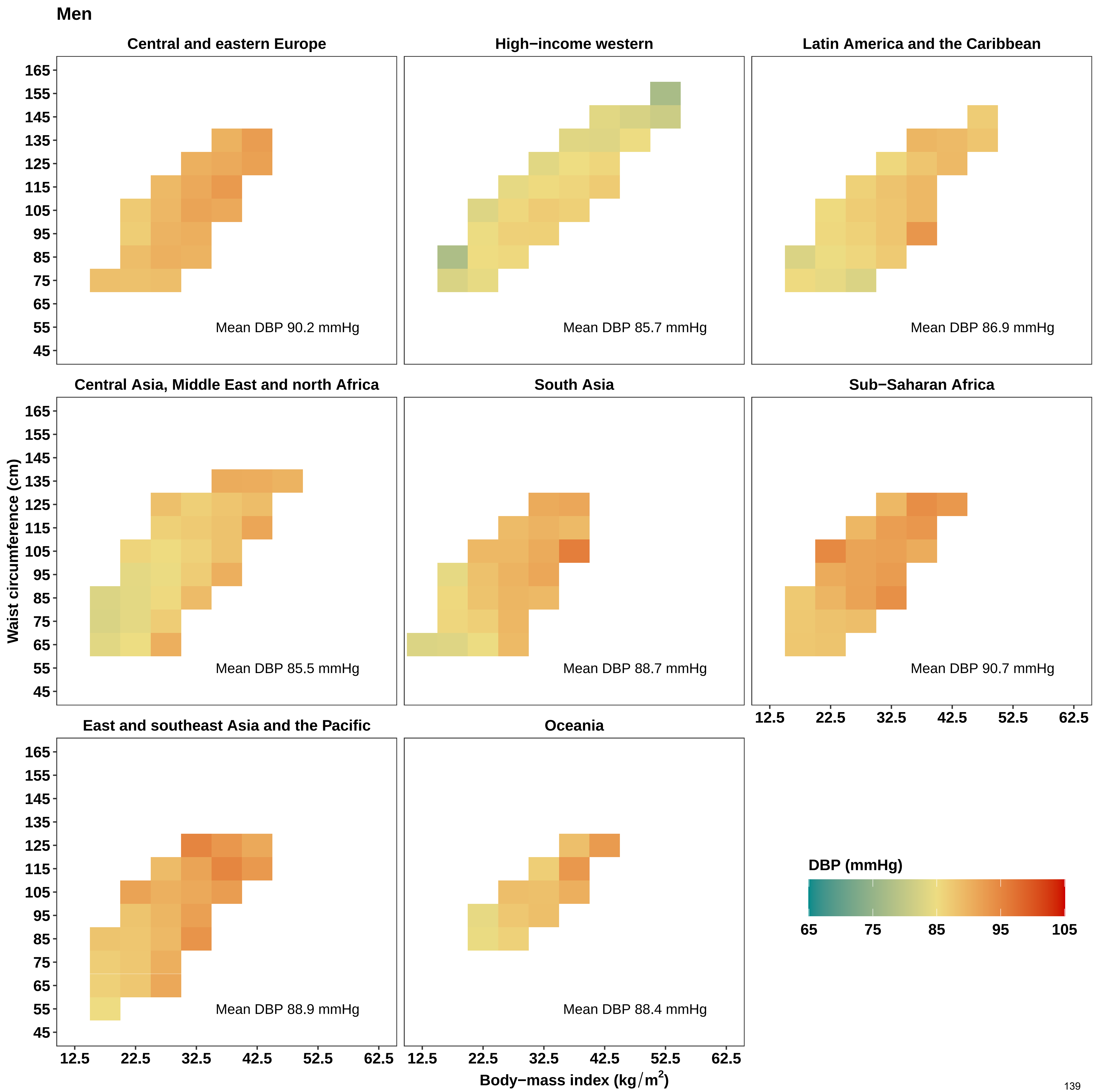

Men
Central and eastern Europe
High−income western
Latin America and the Caribbean
Central Asia, Middle East and north Africa
South Asia
Sub−Saharan Africa
East and southeast Asia and the Pacific
Oceania
Mean DBP 90.2 mmHg
Mean DBP 85.7 mmHg
Mean DBP 86.9 mmHg
Mean DBP 85.5 mmHg
Mean DBP 88.7 mmHg
Mean DBP 90.7 mmHg
Mean DBP 88.9 mmHg
Mean DBP 88.4 mmHg
Waist circumference (cm)
165
155
145
135
125
115
105
95
85
75
65
55
45
Body−mass index (kg/m²)
12.5
22.5
32.5
42.5
52.5
62.5
DBP (mmHg)
65
75
85
95
105

Appendix Figure 31: Odds ratio (OR) for prevalent hypertension per standard deviation (SD) of body-mass index (BMI) and of waist circumference (WC) with and without mutual adjustment.

In each panel, the upper point shows OR without adjustment for the 2nd adiposity index and the lower point shows OR with adjustment for the 2nd adiposity index.

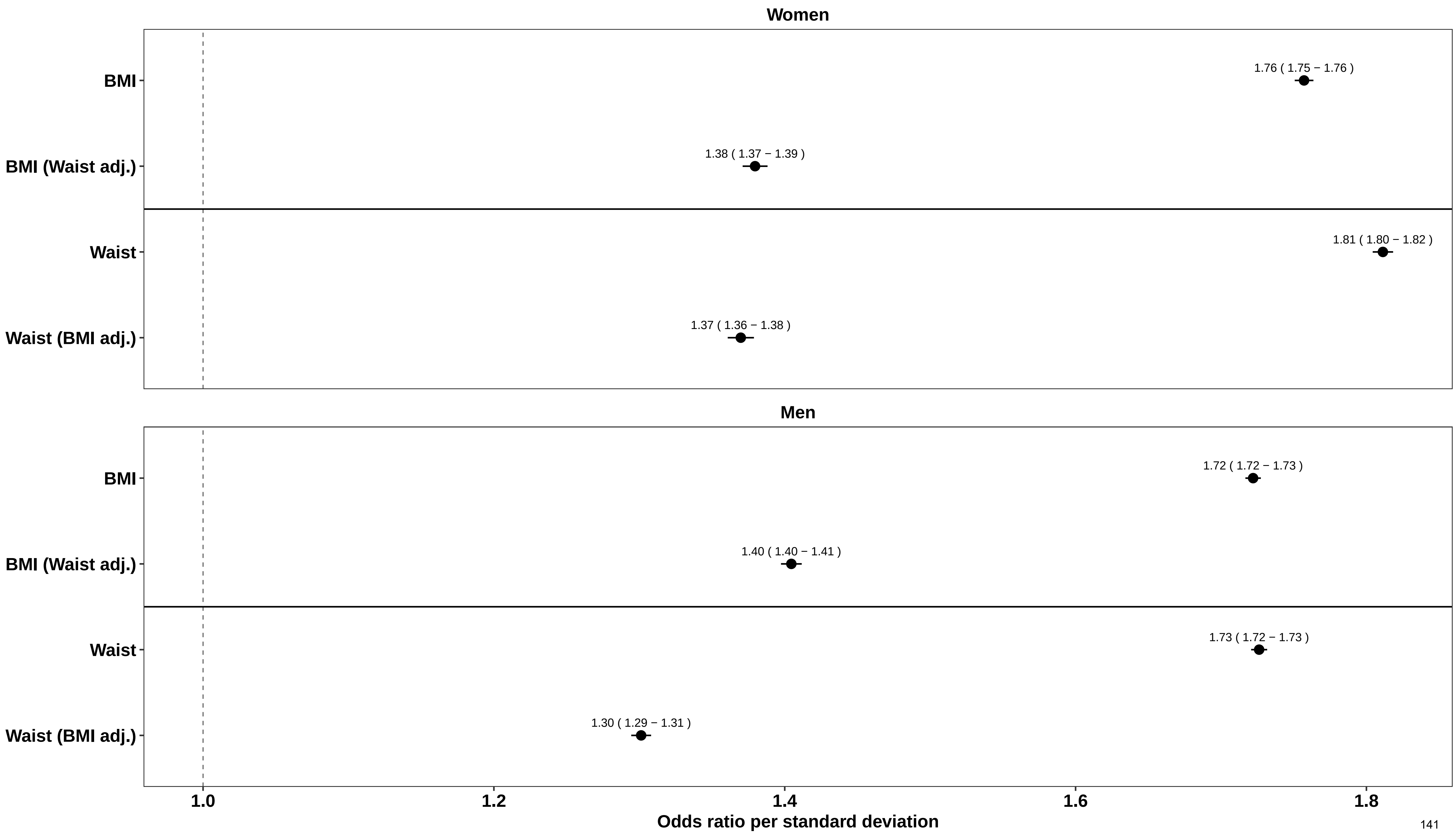
Women
BMI
1.76 (1.75 − 1.76)
BMI (Waist adj.)
1.38 (1.37 − 1.39)
Waist
1.81 (1.80 − 1.82)
Waist (BMI adj.)
1.37 (1.36 − 1.38)
Men
BMI
1.72 (1.72 − 1.73)
BMI (Waist adj.)
1.40 (1.40 − 1.41)
Waist
1.73 (1.72 − 1.73)
Waist (BMI adj.)
1.30 (1.29 − 1.31)
1.0
1.2
1.4
1.6
1.8
Odds ratio per standard deviation

References

1. NCD Risk Factor Collaboration. Worldwide trends in underweight and obesity from 1990 to 2022: a pooled analysis of 3663 population-representative studies with 222 million children, adolescents, and adults. *The Lancet* 2024; **403**(10431): 1027-50.
2. NCD Risk Factor Collaboration. Worldwide trends in hypertension prevalence and progress in treatment and control from 1990 to 2019: a pooled analysis of 1201 population-representative studies with 104 million participants. *The Lancet* 2021; **398**(10304): 957-80.
3. NCD Risk Factor Collaboration. Trends in adult body-mass index in 200 countries from 1975 to 2014: a pooled analysis of 1698 population-based measurement studies with 19·2 million participants. *Lancet* 2016; **387**(10026): 1377-96.
4. Farzadfar F, Finucane MM, Danaei G, et al. National, regional, and global trends in serum total cholesterol since 1980: systematic analysis of health examination surveys and epidemiological studies with 321 country-years and 3·0 million participants. *Lancet* 2011; **377**(9765): 578-86.
5. Finucane MM, Stevens GA, Cowan MJ, et al. National, regional, and global trends in body-mass index since 1980: systematic analysis of health examination surveys and epidemiological studies with 960 country-years and 9·1 million participants. *Lancet* 2011; **377**(9765): 557-67.
6. Danaei G, Finucane MM, Lin JK, et al. National, regional, and global trends in systolic blood pressure since 1980: systematic analysis of health examination surveys and epidemiological studies with 786 country-years and 5.4 million participants. *Lancet* 2011; **377**(9765): 568-77.
7. Danaei G, Finucane MM, Lu Y, et al. National, regional, and global trends in fasting plasma glucose and diabetes prevalence since 1980: systematic analysis of health examination surveys and epidemiological studies with 370 country-years and 2.7 million participants. *Lancet* 2011; **378**(9785): 31-40.
8. Rousseeuw PJ, Van Zomeren BC. Unmasking multivariate outliers and leverage points. *Journal of the American Statistical association* 1990; **85**(411): 633-9.
9. Hsieh SD, Yoshinaga H, Muto T. Waist-to-height ratio, a simple and practical index for assessing central fat distribution and metabolic risk in Japanese men and women. *International Journal of Obesity* 2003; **27**(5): 610-6.
10. Ho S-Y, Lam T-H, Janus ED. Waist to stature ratio is more strongly associated with cardiovascular risk factors than other simple anthropometric indices. *Annals of Epidemiology* 2003; **13**(10): 683-91.
11. Cai L, Liu A, Zhang Y, Wang P. Waist-to-height ratio and cardiovascular risk factors among Chinese adults in Beijing. *PLoS ONE* 2013; **8**(7): e69298.
12. Li WC, Chen IC, Chang YC, Loke SS, Wang SH, Hsiao KY. Waist-to-height ratio, waist circumference, and body mass index as indices of cardiometabolic risk among 36,642 Taiwanese adults. *Eur J Nutr* 2013; **52**(1): 57-65.
13. Chen Z, Smith M, Du H, et al. Blood pressure in relation to general and central adiposity among 500 000 adult Chinese men and women. *Int J Epidemiol* 2015; **44**(4): 1305-19.
14. Kuciene R, Dulskiene V. Associations between body mass index, waist circumference, waist-to-height ratio, and high blood pressure among adolescents: a cross-sectional study. *Scientific Reports* 2019; **9**(1).
15. Pasdar Y, Moradi S, Moludi J, et al. Waist-to-height ratio is a better discriminator of cardiovascular disease than other anthropometric indicators in Kurdish adults. *Scientific Reports* 2020; **10**(1).
16. Momin M, Fan F, Li J, et al. Joint effects of body mass index and waist circumference on the incidence of hypertension in a community-based Chinese population. *Obesity Facts* 2020; **13**(2): 245-55.
17. Savva S, Tornaritis M, Savva M, et al. Waist circumference and waist-to-height ratio are better predictors of cardiovascular disease risk factors in children than body mass index. *International Journal of Obesity* 2000; **24**(11): 1453-8.

18. Jayawardana R, Ranasinghe P, Sheriff MH, Matthews DR, Katulanda P. Waist to height ratio: a better anthropometric marker of diabetes and cardio-metabolic risks in South Asian adults. *Diabetes Res Clin Pract* 2013; **99**(3): 292-9.
19. Luz RH, Barbosa AR, d'Orsi E. Waist circumference, body mass index and waist-height ratio: Are two indices better than one for identifying hypertension risk in older adults? *Prev Med* 2016; **93**: 76-81.
20. Wu X, Li B, Lin WQ, et al. The association between obesity indices and hypertension: Which index is the most notable indicator of hypertension in different age groups stratified by sex? *Clin Exp Hypertens* 2019; **41**(4): 373-80.
21. Petermann-Rocha F, Ulloa N, Martinez-Sanguinetti MA, et al. Is waist-to-height ratio a better predictor of hypertension and type 2 diabetes than body mass index and waist circumference in the Chilean population? *Nutrition* 2020; **79-80**: 110932.
22. Bovet P, Arlabosse T, Viswanathan B, Myers G. Association between obesity indices and cardiovascular risk factors in late adolescence in the Seychelles. *BMC Pediatr* 2012; **12**: 176.
23. Asia Pacific Cohort Studies Collaboration. Central obesity and risk of cardiovascular disease in the Asia Pacific Region. *Asia Pacific Journal of Clinical Nutrition* 2006; **15**(3): 287-92.
24. Huxley R, James WPT, Barzi F, et al. Ethnic comparisons of the cross-sectional relationships between measures of body size with diabetes and hypertension. *Obesity Reviews* 2008; **9**(s1): 53-61.
25. Decoda Study Group, Nyamdorj R, Qiao Q, et al. BMI compared with central obesity indicators in relation to diabetes and hypertension in Asians. *Obesity (Silver Spring)* 2008; **16**(7): 1622-35.
26. Huxley R, Mendis S, Zheleznyakov E, Reddy S, Chan J. Body mass index, waist circumference and waist:hip ratio as predictors of cardiovascular risk--a review of the literature. *Eur J Clin Nutr* 2010; **64**(1): 16-22.
27. Emerging Risk Factors Collaboration. Separate and combined associations of body-mass index and abdominal adiposity with cardiovascular disease: collaborative analysis of 58 prospective studies. *Lancet* 2011; **377**(9771): 1085-95.
28. Ashwell M, Gunn P, Gibson S. Waist-to-height ratio is a better screening tool than waist circumference and BMI for adult cardiometabolic risk factors: systematic review and meta-analysis. *Obesity Reviews* 2012; **13**(3): 275-86.
29. Savva S, Lamnisos D, Kafatos A. Predicting cardiometabolic risk: waist-to-height ratio or BMI. A meta-analysis. *Diabetes, Metabolic Syndrome and Obesity: Targets and Therapy* 2013: 403.
30. Seo DC, Choe S, Torabi MR. Is waist circumference ⩾102/88cm better than body mass index ⩾30 to predict hypertension and diabetes development regardless of gender, age group, and race/ethnicity? Meta-analysis. *Prev Med* 2017; **97**: 100-8.
31. Chandramouli C, Tay WT, Bamadhaj NS, et al. Association of obesity with heart failure outcomes in 11 Asian regions: A cohort study. *PLoS Med* 2019; **16**(9): e1002916.
32. Obesity in Asia Collaboration. Is central obesity a better discriminator of the risk of hypertension than body mass index in ethnically diverse populations? *J Hypertens* 2008; **26**(2): 169-77.
33. Bajaj HS, Pereira MA, Anjana RM, et al. Comparison of relative waist circumference between Asian Indian and US adults. *Journal of obesity* 2014; **2014**.
34. Ragavan RS, Ismail J, Evans RG, et al. Combining general and central measures of adiposity to identify risk of hypertension: a cross-sectional survey in rural India. *Obesity research & clinical practice* 2023; **17**(3): 249-56.
35. Patel SA, Deepa M, Shivashankar R, et al. Comparison of multiple obesity indices for cardiovascular disease risk classification in South Asian adults: The CARRS Study. *PLoS One* 2017; **12**(4): e0174251.
36. Ware L, Rennie K, Kruger HS, et al. Evaluation of waist-to-height ratio to predict 5 year cardiometabolic risk in sub-Saharan African adults. *Nutrition, Metabolism and Cardiovascular Diseases* 2014; **24**(8): 900-7.

37. World Health Organization. Waist Circumference and Waist-Hip Ratio: Report of a WHO Expert Consultation. 2008.
38. Balkau B, Deanfield JE, Despres JP, et al. International Day for the Evaluation of Abdominal Obesity (IDEA): a study of waist circumference, cardiovascular disease, and diabetes mellitus in 168,000 primary care patients in 63 countries. *Circulation* 2007; **116**(17): 1942-51.
39. Lee CMY, Woodward M, Pandeya N, et al. Comparison of relationships between four common anthropometric measures and incident diabetes. *Diabetes research and clinical practice* 2017; **132**: 36-44.
